# Synthesis of Aryl and Heteroaryl Diazophosphonates via Palladium-Catalyzed Cross-Coupling and Their Application in Rhodium-Catalyzed Cyclopropanation

**DOI:** 10.1021/acs.joc.6c00961

**Published:** 2026-07-10

**Authors:** Yasir Naeem, Duc Ly, Huw M. L. Davies

**Affiliations:** Department of Chemistry, 1371 Emory University, 1515 Dickey Drive, Atlanta, Georgia 30322, United States

## Abstract

Aryl and heteroaryl diazophosphonates are valuable intermediates in organic synthesis and medicinal chemistry. This study presents an efficient and versatile strategy for their synthesis via palladium-catalyzed cross-coupling reactions using readily available diazophosphonate precursors and diverse aryl- and heteroaryl iodides. The method demonstrates excellent yields, broad functional group tolerance, and scalability. Notably, the synthetic utility of the resulting diazophosphonates is showcased through a rhodium-catalyzed cyclopropanation, yielding complex cyclopropane derivatives with high diastereo- and enantioselectivity. Highly enantioselective cyclopropanation was also feasible. This combined palladium- and rhodium-catalyzed approach offers a robust, scalable, and stereoselective pathway for accessing multifunctional scaffolds, thereby expanding the capabilities of modern synthetic chemistry.

## Graphical abstract

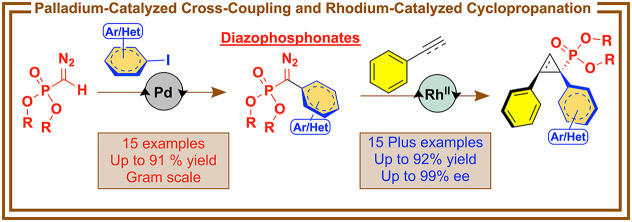

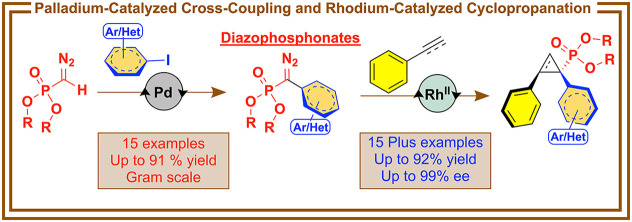

## Introduction

Aryl diazophosphonates are highly versatile compounds with broad applications in organic synthesis, pharmaceuticals, and materials science.^1–14^ The combination of a reactive diazo group and the functional flexibility of the phosphonate moiety makes them useful intermediates for building complex molecular frameworks.^1,6,11,14–18^ However, the development of efficient, scalable, and selective strategies for their synthesis remains a challenge.^19–23^ The sulfonyl azide-mediated diazo transfer reaction, which is commonly used for the synthesis of various diazo compounds,^24,25^ has not been particularly effective for the synthesis of α-aryldiazophosphonates, proceeding in relatively low yields in most cases.^11,26–28^ The most effective diazo transfer method requires a strong base, potassium *tert*-butoxide, rather than the typical amines such as triethylamine or DBU, which limits functional group compatibility (Scheme 1, Method A).^29–36^ An alternative method (Method B) involves three stages, formation of an α-ketophosphonate via an Arbuzov reaction, conversion to a tosylhydrazone, and base-induced elimination to generate the diazophosphonate.^37–46^ The third method (Method C) employs a two-step process starting with the synthesis of α-diazomethylphosphonate, followed by a Pd(0)-catalyzed cross-coupling reaction with aryl iodides to form the desired diazo compounds.^18,47^ A photoredox strategy has also been reported for the synthesis of aryl diazophosphonates through visible-light-mediated C–H functionalization (Method D). However, this photoredox approach has only been demonstrated for a single example and has not been extended to heteroaryl substrates.^48^ None of these processes have been applied to the synthesis of heteroaryl diazophosphonates.

**Scheme 1:**
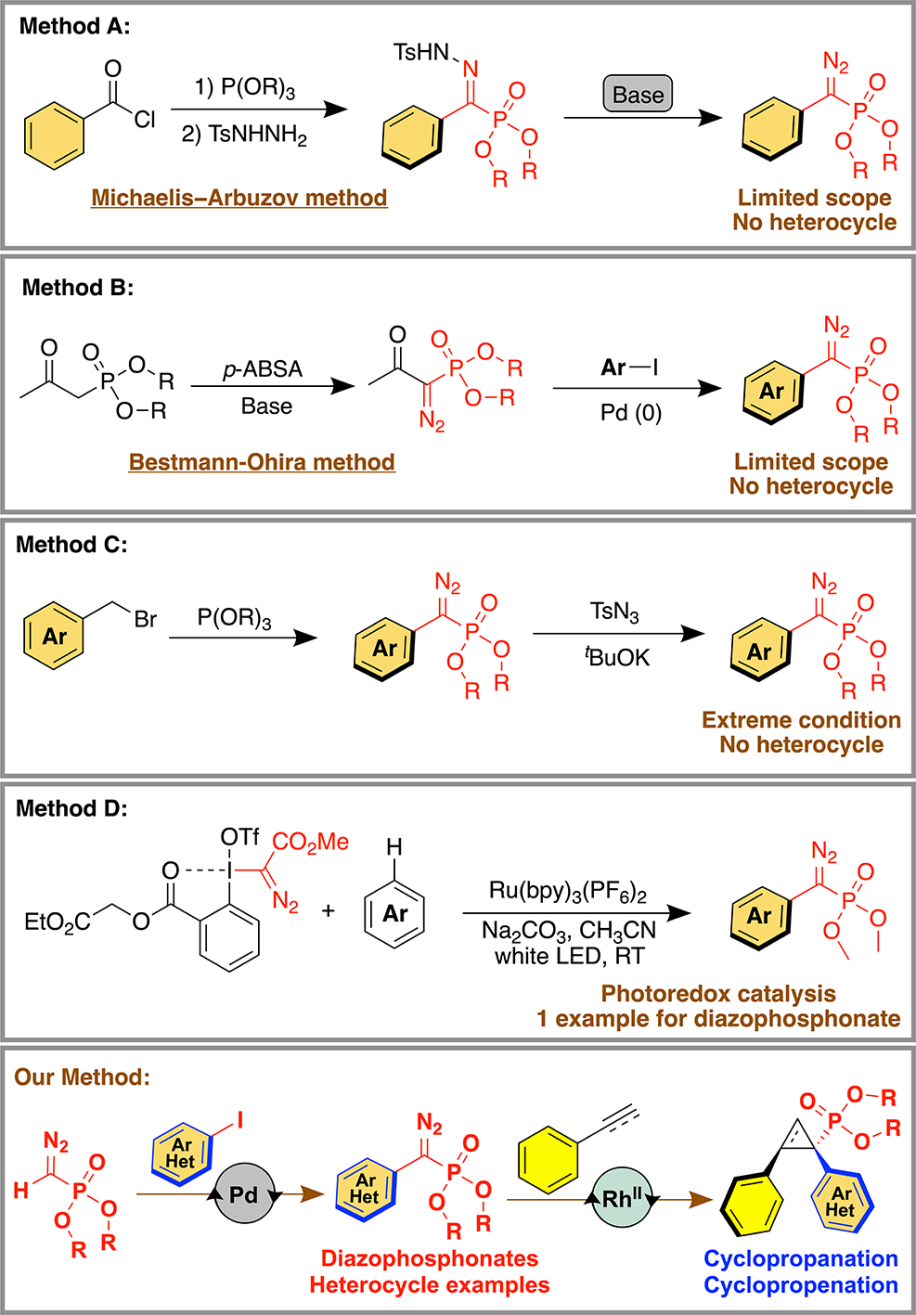
Existing Approaches for α-Aryldiazophosphonate Synthesis

An alternative approach that has proven to be generally useful for the synthesis of aryldiazoacetates has been the palladium-catalyzed cross-coupling reaction between aryl iodides and alkyl diazoacetates.^49–55^ Having found this transformation to have broad utility; we became intrigued with the possibility of a similar type of reaction with diazophosphonates as the coupling partners. In this paper, we describe the development of such a reaction, with a particular focus on introducing heteroaryl iodides as coupling partners (Scheme 1, our method). We also developed suitable conditions for applying these diazophosphonates into enantioselective cyclopropanation and cyclopropenation.

## Results and Discussion

The optimization studies for the palladium-catalyzed cross-coupling reaction were conducted using pyridyl iodide **2a** and diazophosphonate **1a** as model substrates (Table 1). Optimization of reaction conditions began using 5 mol % of the palladium catalyst, 1.0 equiv of K_2_CO_3_ base, and toluene as the solvent (entries 1–4). The reactions were generally low yielding, but the best result of 15% yield was obtained with Pd­(PPh_3_)_4_ as the catalyst. In the palladium-catalyzed cross-coupling reaction with diazoacetates, an extra phosphine ligand was found to be beneficial,^51^ and so the role of an additional phosphine ligand (10 mol %) was examined (entries 5–8). Triphenylphosphine was most effective, resulting in an improved yield of 33% (entry 8). The effect of the base selection was then explored. A combination of K_2_CO_3_ and NEt_3_ increased the yield to 40% (entry 9), while Cs_2_CO_3_ and NEt_3_ further enhanced it to 52% (entry 10). The highest yield of 58% was achieved with Ag_2_CO_3_ and NEt_3_ (entry 11).

**Table 1:**
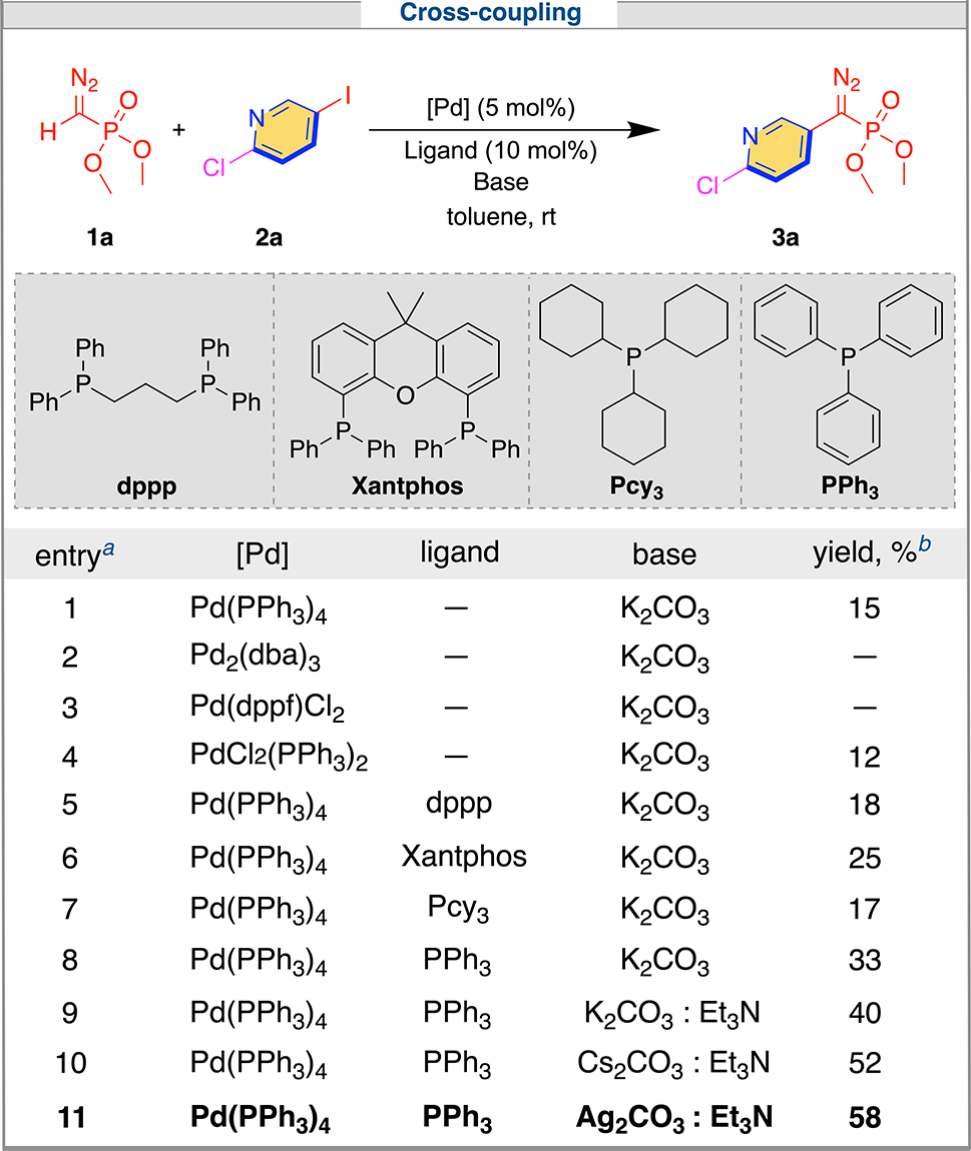
Optimization of the Reaction Conditions^a^

a Reaction condition: Diazophosphonate **1a** (1.3 equiv), iodopyridine **2a** (1.0 equiv), NEt_3_ (1.0 equiv), Ag_2_CO_3_ (0.75 equiv), Pd­(PPh_3_)_4_ (5 mol %), and ligand (10 mol %) in toluene at room temperature for 6 h (monitored by TLC).

b Isolated yield of **3a**.

With the optimized conditions in hand, the substrate scope was explored using a variety of aryl and heteroaryl iodides (Table 2). A series of aryl iodides were compatible with this reaction, resulting in the formation of the aryldiazophosphonates **3a–f**. The reaction is readily scalable as illustrated in the formation of the phenyldiazophosphonate **3b** in 86% yield on a five-gram scale. Of particular note is the formation of the boron pinacolate derivative **3g** in 85% yield, illustrating the compatibility of boron functionality with the cross-coupling conditions and offering useful functionality for further diversification. The reaction was further applied to more elaborate substrates. Thiophene compatibility was readily seen in the formation of **3h** (75% yield), which contains a major fragment present in canagliflozin (a therapeutic agent for type 2 diabetes).^56^ To highlight the broader applicability of this methodology, several diazophosphonates incorporating heterocyclic rings (**3i**–**j**, **3n**, and **3o**) were prepared in yields ranging from 40% to 60%. Aryldiazoacetates functionalized with electron-donating groups tend to be more challenging to prepare because they are more reactive to nitrogen extrusion. Hence, we explored whether an aryldiazophosphonate was accessible using the cross-coupling strategy, and we were pleased to observe the formation of the morpholine derivative **3k**. As the conditions used herein are not strongly basic, we also demonstrated that the phenylalanine derivative **3m** could be readily prepared.

**Table 2:**
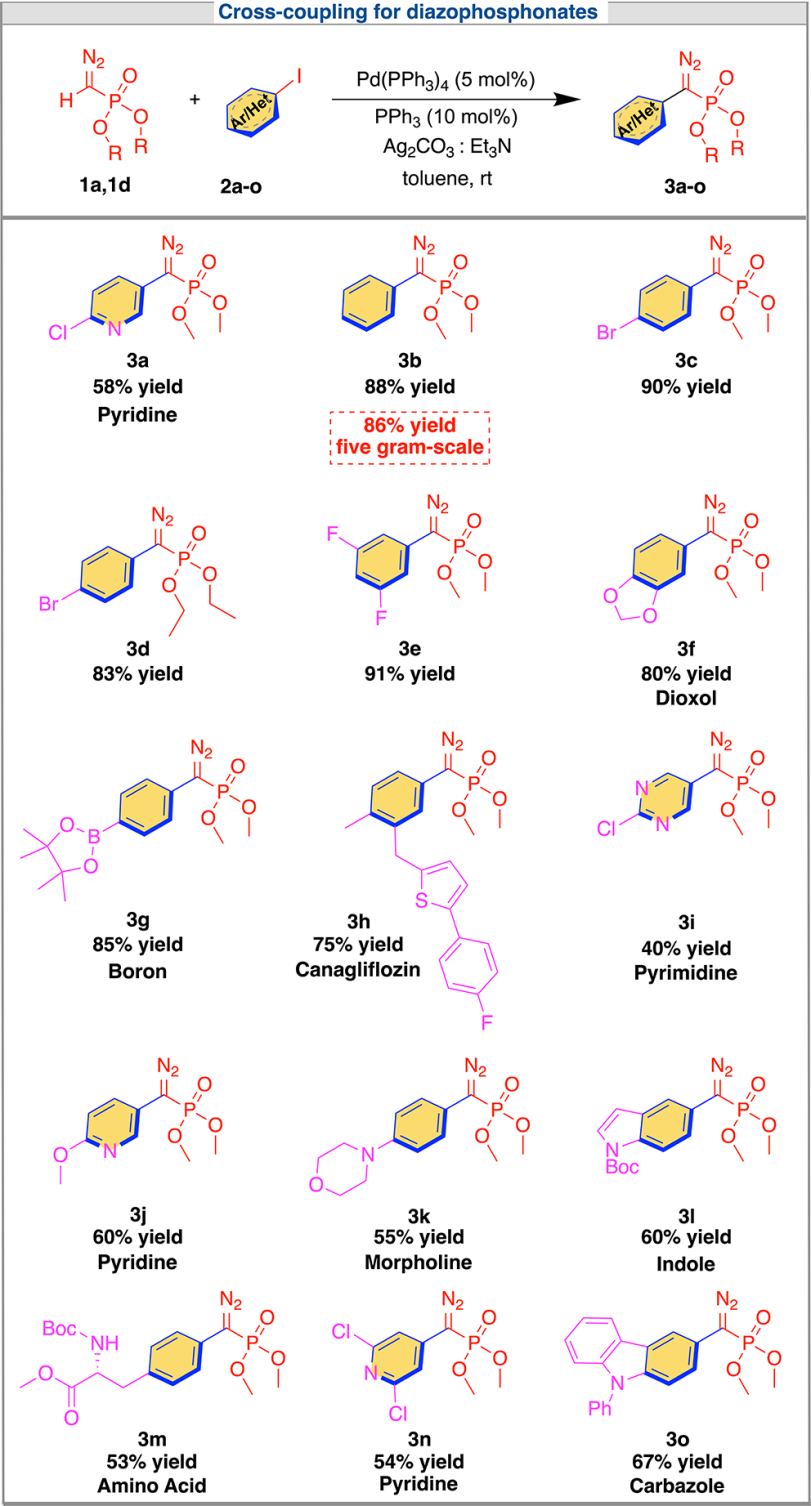
Scope of Aryl- and Heteroaryl Diazophosphonates^a^

a Reaction conditions: Diazophosphonate **1a** or **1d** (1.3 equiv), aryl or heteroaryl iodide **2a–o** (1.0 equiv), NEt_3_ (1.0 equiv), Ag_2_CO_3_ (0.75 equiv), Pd­(PPh_3_)_4_ (5 mol %), and ligand (10 mol %) in 15 mL of toluene at room temperature for 2–8 h (monitored by TLC). The reported yields are isolated yields after purification.

The cross-coupling reaction afforded several novel aryl and heteroaryl diazophosphonates. Therefore, a study was conducted to determine how effective the diazophosphonate would be in enantioselective cyclopropanation and cyclopropenation.^19,29,35,57–70,79^ The first stage involved determining the optimum dirhodium catalyst for these reactions and the results are summarized in Table 3. We first examined the reaction with our standard catalysts (entries 1–4), under refluxing conditions in dichloromethane, and as had been previously observed,^31^ Rh_2_(*S*-PTAD)_4_ gave the best result, generating **6b** in 85% yield and 94% ee (entry 2). As is typical of the reactions with donor/acceptor carbenes, all the reactions were highly diastereoselective, favoring the *E*-isomer. The naphthylimido-derived catalyst Rh_2_(*S*-NTTL)_4_ does not typically perform well with aryldiazoacetates,^71^ but performed much better with the aryl diazophosphonates, producing **6b** in 90% yield and 80% ee (entry 5). This promising result led to the evaluation of other Rh_2_(*S*-NTTL)_4_ derivatives. Our recently developed tetraphenyl derivative Rh_2_(*S*-TPNTTL)_4_ ^71^ achieved 92% yield and 90% ee (entry 6), while the diaryl derivative Rh_2_(*S*-di­(4-^*t*^BuPh)­NTTL)_4_ ^71^ gave a 90% yield and 92% ee (entry 7). The reactions were then conducted at 25 °C and the lower temperature caused improvement in the enantioselectivity as can be seen in the Rh_2_(*S*-TPNTTL)_4_, Rh_2_(*S*-di­(3,5-^*t*^BuPh)­NTTL)_4_, and Rh_2_(*S*-PTAD)_4_-catalyzed reactions (entries 8–10). The best results were obtained with Rh_2_(*S*-di­(3,5-^*t*^BuPh)­NTTL)_4_,^71^ generating **6b** in 92% yield, >30:1 dr, and 96% ee (entry 10).

**Table 3:**
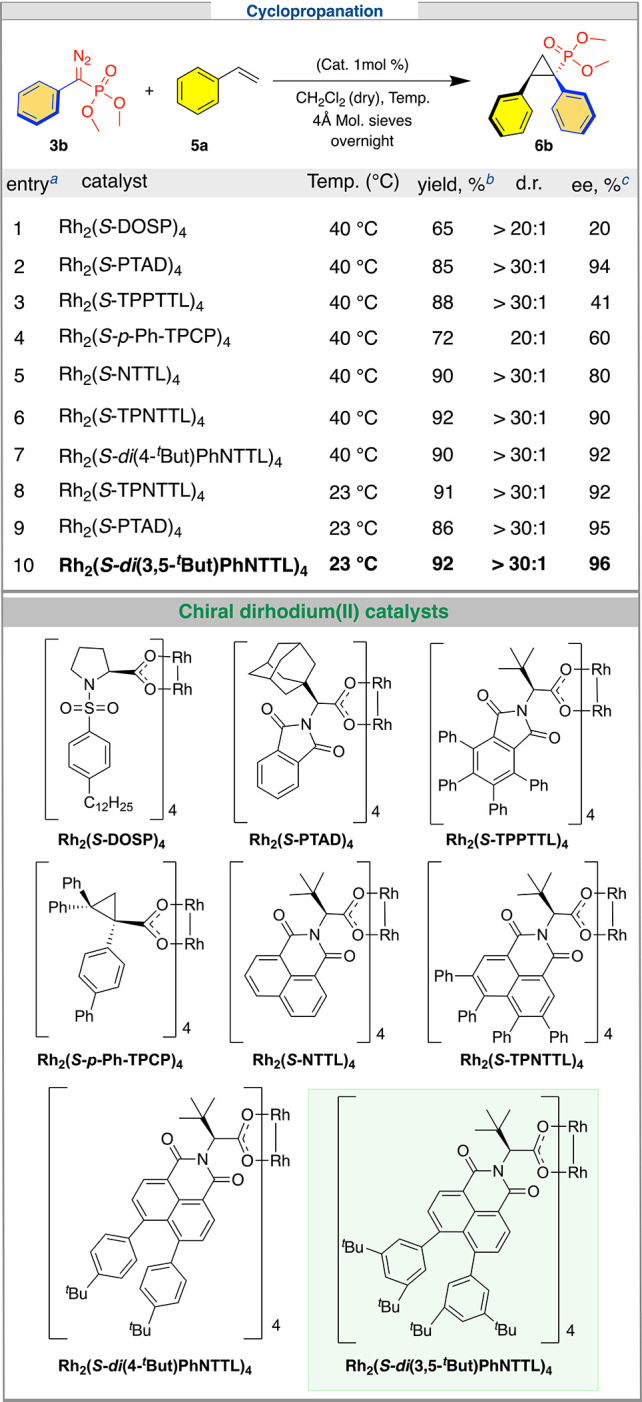
Optimization of the Reaction Conditions for Cyclopropanation Using Dirhodium Tetracarboxylate Catalysts^a^

a Reaction conditions: To a mixture of **5** (1.0 mmol) and Rh_2_L_4_ catalyst (1 mol %) in dry CH_2_Cl_2_ solvent (5.0 mL) was added a solution of **3b** (0.2 mmol) in dry CH_2_Cl_2_ solvent (10.0 mL) via an automatic syringe pump over 5 h over activated 4 Å molecular sieves and the mixture was stirred overnight.

b Combined isolated yields of **6b**.

c Enantioselectivity (ee) was determined by SFC or HPLC analysis.

Once Rh_2_(*S*-di­(3,5-^*t*^BuPh)­NTTL)_4_ was identified as the optimal catalyst, its efficacy was evaluated across a diverse range of diazo substrates **3a**–**o**, demonstrating excellent functional group tolerance and broad substrate scope, as summarized in Table 4. Cyclopropanation of styrene with a diverse set of diazo substrates bearing a broad range of functional groups proceeded efficiently, affording the corresponding cyclopropanes in good to excellent yields with high levels of enantio- and diastereoselectivity. The pyridine derivative **3a** yielded **6a** in 70% isolated yield with outstanding enantioselectivity (99% ee) and >30:1 d.r. Phenyldiazophosphonate **3b** generated **6b** in 92% yield with 96% ee and >30:1 d.r. Diazo compound **3c**, bearing a *para*-bromo substituent, provided **6c** in 80% yield with excellent enantioselectivity (98% ee) and >30:1 d.r. Similarly, substrate **3d**, containing an ethyl phosphonate, afforded **6d** in 85% yield with 90% ee and >30:1 d.r., while substrate **3e**, featuring *meta*-difluoro substituents, delivered **6e** in 78% yield with 90% ee and >30:1 d.r. The dioxolane-containing substrate **3f** furnished **6f** in 84% yield with 97% ee and >30:1 d.r. The boronate-functionalized substrate **3g** also proved effective, affording **6g** in 75% yield with 86% ee and >30:1 d.r. The more elaborate substrate **3h**, incorporating thiophene and fluorophenyl moieties, produced **6h** in 68% yield with 90% ee and >30:1 d.r. The chloropyrimidine derivative **3i** afforded **6i** in 53% yield with 97% ee and >30:1 d.r. Likewise, the 2-methoxypyridine derivative **3j** delivered **6j** in 75% yield with exceptional enantioselectivity (99% ee) and >30:1 d.r. In contrast, diazo compound **3k**, incorporating a morpholine unit, gave **6k** in 55% yield with 88% ee and >30:1 d.r. Diazo compound **3l**, featuring an indole moiety, delivered **6l** in 72% yield with moderate enantioselectivity (66% ee) and >30:1 d.r., while the amino acid-derived substrate **3m** afforded **6m** in 63% yield with high diastereomeric purity (96% de). The substitution pattern on the pyridine ring significantly influenced enantioselectivity, as the 2,6-dichloropyridine derivative **3n** furnished **6n** with only 41% ee. Finally, the carbazole-containing substrate **3o** provided **6o** in 81% yield with 96% ee and >30:1 d.r. The absolute configurations of **6a**, **6c**, **6g**, and **6j** were determined by X-ray crystallography, and the remaining products were assigned by analogy. In summary the majority of the diazophosphonates resulted in >90% ee, with 4-substituted aryl or heteroaryl derivatives achieving exceptionally high enantioselectivity, as seen with **6b**, **6k**, **6l**, and **6m** (97–99% ee).

**Table 4:**
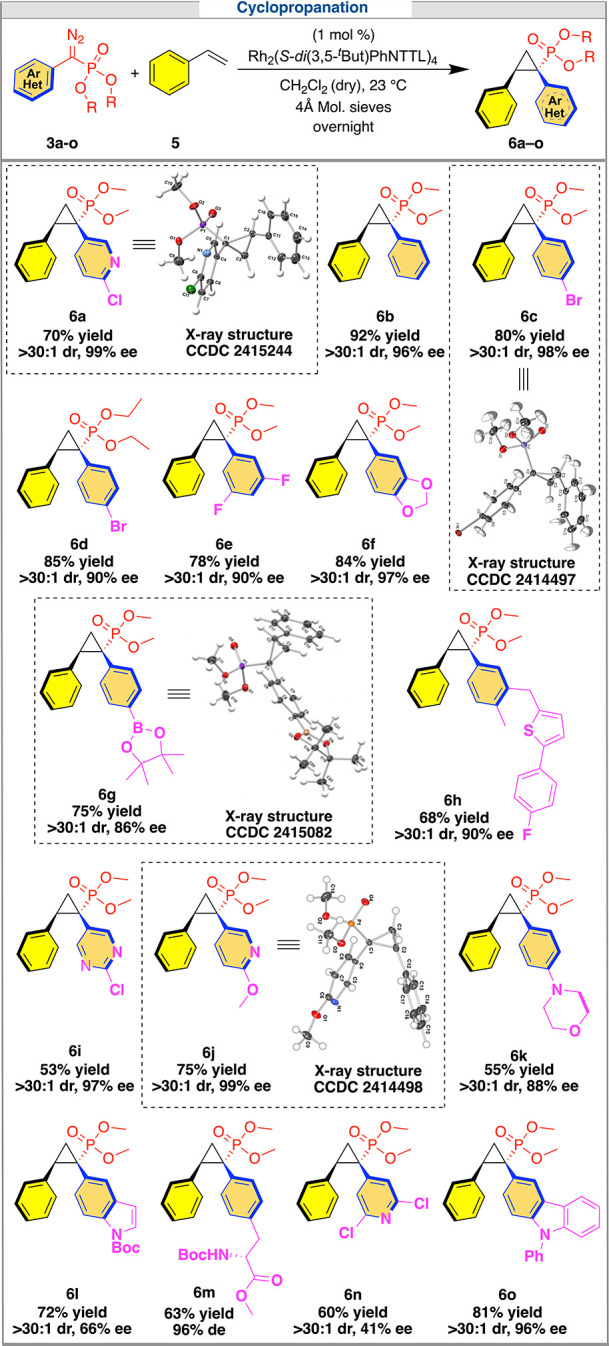
Scope of Aryl- and Heteroaryl Cyclopropanation Reactions Using Dirhodium Tetracarboxylate Catalysts^a^

a Reaction conditions: To a mixture of **5** (1.0 mmol) and Rh_2_(*S*-di­(3,5-^*t*^BuPh)­NTTL)_4_ (1 mol %) in dry CH_2_Cl_2_ solvent (5.0 mL) was added a solution of **3a**–**o** (0.2 mmol) in dry CH_2_Cl_2_ solvent (10.0 mL) via an automatic syringe pump over 5 h. The mixture was stirred overnight, and all reactions were performed with freshly distilled dry CH_2_Cl_2_ stored over activated 4 Å molecular sieves. The yields of **6a**–**o** are reported as isolated yields after purification. Enantioselectivity (ee) was determined by chiral HPLC or SFC analysis of the isolated products.

To further illustrate the synthetic potential of the diazophosphonates, an enantioselective cyclopropenation was also conducted. The Rh_2_(*S*-di­(3,5-^*t*^BuPh)­NTTL)_4_-catalyzed reaction of the diazophosphonate **3b** with phenylacetylene **7** proceeded very smoothly, generating the cyclopropene **8a** in 95% isolated yield with 95% ee (Scheme 2). This result marks a significant enhancement in both yield and enantioselectivity compared with previously reported methodologies.^72^

**Scheme 2:**
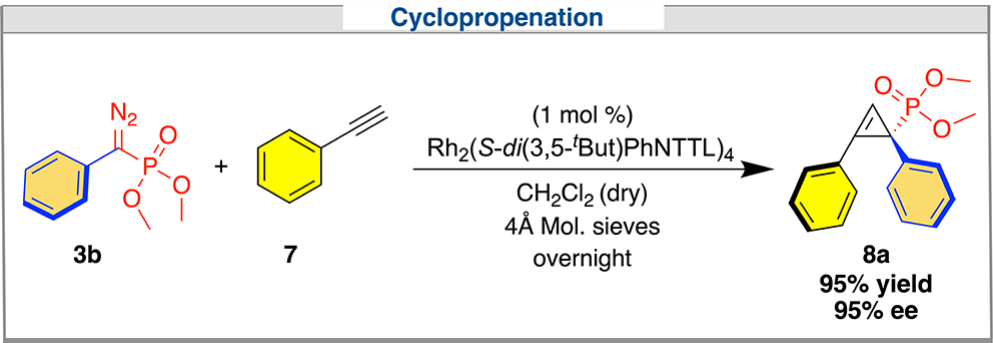
Cyclopropenation Reactions Using Dirhodium Tetracarboxylate Catalysts^a^

a Reaction conditions: To a mixture of **7** (1.0 mmol) and Rh_2_(*S*-di­(3,5-^*t*^BuPh)­NTTL)_4_ (1 mol %) in dry CH_2_Cl_2_ solvent (5.0 mL) was added a solution of **3b** (0.2 mmol) in dry CH_2_Cl_2_ solvent (10.0 mL) via an automatic syringe pump over 5 h. The mixture was stirred overnight, and all reactions were performed with freshly distilled dry CH_2_Cl_2_ stored over activated 4 Å molecular sieves. The yields of **8a** are reported as isolated yield after purification. Enantioselectivity (ee) was determined by chiral HPLC analysis of the isolated products.

## Conclusion

The palladium-catalyzed cross-coupling of aryl and heteroaryl diazophosphonates represents a significant advancement in the synthesis of elaborate diazo compounds. By systematically optimizing reaction conditions, including the precise selection of palladium catalysts, ligands, and bases—this methodology delivers a streamlined, high-yielding, and mild approach for the generation of these valuable diazo compounds. Notably, the developed strategy exhibits broad substrate compatibility, tolerating a wide range of functional groups and enabling scalability, which underscores its practicality for synthetic applications. Furthermore, the synthetic utility of the aryl- and heteroaryl diazophosphonates was showcased through their successful downstream transformation via rhodium-catalyzed cyclopropanation. This transformation efficiently generates donor/acceptor-substituted cyclopropane derivatives with exceptional regio- and stereoselectivity, demonstrating the versatility of diazophosphonates as robust precursors for accessing structurally complex and functionally rich molecules.

## Experimental Section

### General Information

All chemicals were used as purchased or purified as necessary following the procedures outlined in *Purification of Laboratory Chemicals*. Unless otherwise stated, all reagents were obtained from commercial sources and used without further purification. Preparation of 4 Å Molecular Sieves was activated at 220 °C for 4 h under vacuum and stored in an oven maintained at over 100 °C until use. Thin-Layer Chromatography (TLC) analyses were performed using aluminum-backed silica gel plates, and visualization was achieved using UV light or staining with aqueous potassium permanganate (KMnO_4_), ceric ammonium molybdate (CAM), or phosphomolybdic acid (PMA). Flash Column Chromatography was conducted on silica gel (SilicaFlash P60, 40–63 μm) or Merck silica gel 60 Å (230–400 mesh). ^1^H, ^13^C­{^1^H}, ^19^F, and ^31^P NMR spectra were recorded at either 400, 500, 600, or 800 MHz on Bruker spectrometer or Varian spectrometer, (^13^C­{^1^H} at 101, 151, or 201 MHz), 400, 600, or 800 MHz on Bruker spectrometers, (^19^F at 376 or 565) on Bruker-400 or Bruker-600 spectrometers, and (^31^P at 162 or 243 MHz) on Bruker-400 or Bruker-600 spectrometers, and all were reported in parts per million (ppm). All NMR samples were prepared using deuterated chloroform (CDCl_3_) solvent unless otherwise noted. Coupling constant (*J*) values were recorded in Hertz (Hz). Abbreviations for ^1^H NMR signals coupling are as follows: s = singlet, d = doublet, t = triplet, m = multiplet, dd = doublet of a doublet, ddd = doublet of a doublet of a doublet, dtd doublet of a triplet of a doublet, and br = broad. Mass spectra were recorded using Fourier-transform mass spectrometry (FTMS) with either positive nanospray ionization (NSI) or atmospheric pressure chemical ionization (APCI) as the ionization source unless otherwise specified. All reactions were conducted under an argon atmosphere in flame-dried or oven-dried glassware unless otherwise stated. Dichloromethane (CH_2_Cl_2_) used for C–H functionalization reactions was dried over calcium hydride (CaH_2_), stored under argon overnight, and treated with 4 Å molecular sieves. Solvent degassing was performed by sparging with argon for one hour. Solvents, including tetrahydrofuran (THF), diethyl ether (Et_2_O), acetonitrile (CH_3_CN), dichloromethane (CH_2_Cl_2_), trifluorotoluene, and toluene, were dried using a solvent purification system before use. Infrared Spectroscopy (IR) spectra were recorded on a Nicolet iS10 FT-IR spectrometer. Mass spectrometric determinations were carried out on a Thermo Finnigan Exactive Plus Mass spectrometer with electrospray ionization (ESI) or atmospheric pressure chemical ionization (APCI) Quadrupole-Orbitrap Mass spectrometer with ESI and APCI. Melting points (m.p.) were determined in open capillary tubes using a Mel-Temp Electrothermal melting point apparatus and are uncorrected. Enantiomeric Excess (ee) was determined by high-performance liquid chromatography (HPLC) or supercritical fluid chromatography (SFC). HPLC was conducted on Agilent 1100 and Agilent 1290 Infinity UHPLC systems. Traces are based on racemic retention times. Isopropanol/hexane gradients were used with commercial Daicel ChiralPak and ChiralCel columns, including AD-H, OZ-H, OD-H, AS-H, and OJ-H (all 5 μm, 4.6 × 250 mm), and Regis (*S*,*S*)-Whelk-O1 Kromasil columns. SFC was performed using a Waters Acquity UPC2 system with supercritical CO_2_ and cosolvents such as HPLC-grade methanol, acetonitrile, ethanol, isopropanol, or their mixtures. Additives included 0.2% formic acid or 20 mM ammonium formate. SFC columns included Trefoil AMY1, CEL1, CEL2 (all 2.5 μm, 3.0 × 150 mm), Regis (*S*,*S*)-Whelk-O1 Kromasil (3.5 μm, 3.0 × 150 mm), ChiralPak AD-3, OZ-3, OD-3, OX-3, OJ-3, and AS-3 (all 3.0 μm, 3.0 × 150 mm). Coupling constants are uncorrected: When it is stated that “coupling constants are uncorrected,” it typically means that the reported values of coupling constants (*J* values) are directly taken from the spectral data without applying any corrections or refinements. The values are raw and have not undergone advanced processing or theoretical adjustments to refine their accuracy. Rh_2_(*S*-TPNTTL)_4_, Rh_2_(*S*-di­(4-^*t*^BuPhNTTL))_4_, and Rh_2_(*S*-di-(3,5-^*t*^BuPhNTTL))_4_ catalysts were prepared according to the reported literature.^71^

**Caution**: While we never experienced any problems, diazo compounds are presumed to be potentially explosive.

#### *tert*-Butyl 5-Iodo-1*H*-indole-1-carboxylate (**2l**)

A solution of 5-iodoindole **S2l** (2.00 g, 8.229 mmol) in dry methylene chloride (40 mL) was treated with di-*tert*-butyl dicarbonate (2.155 g, 9.875 mmol) and 4-dimethylaminopyridine (201 mg, 1.646 mmol) was added at room temperature and the solution was stirred at room temperature for 16 h using a heating mantle. The crude product was purified by flash column chromatography on silica gel (petroleum ether:EtOAc, 1:1) giving product **2l** as a colorless oil (2.57 g, 91%). ^1^H NMR (600 MHz, CDCl_3_) δ 7.90 (s, 1H), 7.57 (d, *J* = 9.1 Hz, 1H), 7.55 (s, 1H), 6.49 (d, *J* = 3.7 Hz, 1H), 1.67 (d, J = 1.4 Hz, 1H). (Coupling constants are uncorrected). ^13^C­{^1^H} NMR (151 MHz, CDCl_3_) δ 149.8, 134.8, 133.2, 133.0, 130.1, 127.0, 117.4, 106.6, 87.0, 84.5, 28.5. Spectroscopic data are in agreement with those reported in the literature.^73^

#### (*R*)-2-((*tert*-Butoxycarbonyl)­amino)-3-(4-iodophenyl)­propanoic Acid (**2m**)

A solution of halogenated amino acid **S2m** (5.00 g, 12.8 mmol) in DMF (35 mL) was treated with NaHCO_3_ (1.61 g, 19.2 mmol) and iodomethane (2.72 g, 19.2 mmol) was added and the solution was stirred at room temperature for 4 h (monitored by TLC) using a heating mantle. The organic layer was separated, and the aqueous layer was extracted with DCM. The combined organic layers were dried (Na_2_SO_4_), concentrated, and the residue was purified by flash column chromatography on silica gel (hexane:EtOAc, 6:1) to give compound **2m** as a white solid (4.7 g, 90% yield). ^1^H NMR (600 MHz, CDCl_3_) δ 7.64 (d, *J* = 7.1 Hz, 2H), 6.90 (d, *J* = 7.8 Hz, 2H), 5.00 (d, *J* = 8.2 Hz, 1H), 4.59 (d, *J* = 7.0 Hz, 1H), 3.80–3.71 (m, 3H), 3.10 (dd, *J* = 5.7, 14.1 Hz, 1H), 3.00 (dd, *J* = 6.1, 14.0 Hz, 1H), 1.44 (s, 9H). (Coupling constants are uncorrected). ^13^C­{^1^H} NMR (151 MHz, CDCl_3_) δ 172.4, 155.3, 137.9, 136.1, 131.7, 92.9, 80.4, 54.5, 52.7, 38.2, 28.6. Spectroscopic data are in agreement with those reported in the literature.^74^

### Bestmann–Ohira Method^75–77^

To a round-bottom flask, a solution of dimethyl (2-oxopropyl)­phosphonate or diethyl (2-oxopropyl)­phosphonate (1 equiv) in dry toluene (60 mL for a 5-g scale) was added and stirred at 0 °C for 30 min using a heating mantle. Sodium hydride (60% dispersion in mineral oil, 1.2 equiv) was slowly added to the flask, and the mixture was stirred at 0 °C for 1 h under a nitrogen atmosphere. 4-Acetamidobenzenesulfonyl azide *p*-ABSA (1.1 equiv) in dry THF (20 mL) was then added slowly, and the reaction mixture was allowed to warm to room temperature with stirring overnight, monitored by TLC analysis. Upon completion, petroleum ether (25 mL) was added, and the precipitate was filtered through Celite. The filter cake was washed with ether (3 × 25 mL), and the filtrate was concentrated under reduced pressure to remove volatile components. The resulting crude diazo product was purified by flash column chromatography on silica gel (petroleum ether:EtOAc, 1:1), affording the product dimethyl (1-diazo-2-oxopropyl)­phosphonate or diethyl (1-diazo-2-oxopropyl)­phosphonate as a yellow liquid in 84–90% yield. *Dimethyl (1-diazo-2-oxopropyl)­phosphonate.* ^1^H NMR (600 MHz, CDCl_3_) δ 3.83 (d, *J* = 11.8 Hz, 6H), 2.26 (s, 3H). (Coupling constants are uncorrected). ^13^C­{^1^H} NMR (151 MHz, CDCl_3_) δ 190.3 (d, *J* = 13.1 Hz), 63.9 (d, *J* = 221.1 Hz), 53.9 (d, *J* = 5.5 Hz), 27.5. ^31^P NMR (243 MHz, CDCl_3_) δ 14.30. Spectroscopic data are in agreement with those reported in the literature.^18,78,79^

#### Diethyl (1-Diazo-2-oxopropyl)­phosphonate

^1^H NMR (400 MHz, CDCl_3_) δ 4.33–4.08 (m, 4H), 2.28 (s, 3H), 1.38 (t, *J* = 7.1 Hz, 6H). (Coupling constants are uncorrected). ^31^P NMR (162 MHz, CDCl_3_) δ 11.3. Spectroscopic data are in agreement with those reported in the literature.^18,78,79^

A solution of Dimethyl (1-diazo-2-oxopropyl)­phosphonate **S1a** or Diethyl (1-diazo-2-oxopropyl)­phosphonate **S1d** (1 equiv) in methanol (15 mL) was stirred with sodium carbonate (1.2 equiv) at room temperature for 15 min using a heating mantle, with progress monitored by TLC. The reaction mixture was filtered, and the filtrate was concentrated under reduced pressure to remove volatile components. The resulting crude diazo product was purified by flash column chromatography on silica gel (petroleum ether:EtOAc, 1:1), yielding product **1a** or **1d** as a yellow liquid in 84–90% yield.^80^

### General Procedure: Palladium-Catalyzed Cross-Coupling Reaction

Pd­(PPh_3_)_4_ (142 mg, 0.12 mmol, 5 mol %), PPh_3_ (64.3 mg, 0.25 mmol, 10 mol %), aryl iodide (**2a–o**) (500 mg, 2.45 mmol, 1.0 equiv), and Ag_2_CO_3_ (507 mg, 1.84 mmol, 0.75 equiv) were suspended in toluene (15 mL) under an argon atmosphere. NEt_3_ (0.34 mL, 2.45 mmol, 1.0 equiv) and dimethyl (diazomethyl)­phosphonate **1a** (478 mg, 3.19 mmol, 1.3 equiv) or for **1d** diethyl (diazomethyl)­phosphonate (568 mg, 3.19 mmol, 1.3 equiv) were then added to the mixture. The reaction was stirred at room temperature for 4–6 h (monitored by TLC) using a heating mantle, after which it was filtered through a short silica gel column using ethyl acetate as the eluent. The volatiles were removed under reduced pressure, and the residue was purified by column chromatography to yield the desired products (**3a–o**).

### Palladium-Catalyzed Cross-Coupling Reaction for Five-Gram Scale

Pd­(PPh_3_)_4_ (1.42 g, 1.23 mmol, 5 mol %), PPh_3_ (643 mg, 2.45 mmol, 10 mol %), aryl iodide (**2b**) (5.00 g, 24.51 mmol, 1.0 equiv), and Ag_2_CO_3_ (5.07 g, 18.38 mmol, 0.75 equiv) were suspended in toluene (150 mL) under an argon atmosphere. NEt_3_ (3.41 mL, 24.51 mmol, 1.0 equiv) and dimethyl (diazomethyl)­phosphonate **1a** (4.78 g, 31.86 mmol, 1.3 equiv) were then added to the mixture. The reaction was stirred at room temperature for 4–6 h (monitored by TLC) using a heating mantle, after which it was filtered through a short silica gel column using ethyl acetate as the eluent. The volatiles were removed under reduced pressure, and the residue was purified by flash chromatography (SiO_2_; hexanes/EtOAc, 9:1 to 1:1 gradient; *R*_*f*_ = 0.35 in 1:1 hexanes/EtOAc), which afforded the major product **3b** as a yellow oil (4.76 g, 86% yield).

#### Dimethyl ((6-Chloropyridin-3-yl)­(diazo)­methyl)­phosphonate (**3a**)

The material was purified by flash chromatography (SiO_2_; hexanes/EtOAc, 9:1 to 1:1 gradient; *R*_*f*_ = 0.20 in 1:1 hexanes/EtOAc), which afforded major product **3a** as a yellow oil (318 mg, 58% yield). ^1^H NMR (800 MHz, CDCl_3_) δ 8.20 (d, *J* = 2.7 Hz, 1H), 7.45 (dd, *J* = 8.5, 2.8 Hz, 1H), 7.29 (d, *J* = 8.5 Hz, 1H), 3.82 (d, *J*_HP_ = 11.9 Hz, 6H). (Coupling constants are uncorrected). ^13^C­{^1^H} NMR (201 MHz, CDCl_3_) δ 148.4, 143.8 (d, *J*_CP_ = 4.5 Hz), 132.8 (d, *J*_CP_ = 3.8 Hz), 124.9, 123.2 (d, *J*_CP_ = 10.1 Hz), 53.8 (d, *J*_CP_ = 5.1 Hz), 48.2 (d, *J*_CP_ = 229.7 Hz). ^31^P NMR (162 MHz, CDCl_3_) δ 18.51. IR (neat): 2955, 2083, 1549, 1515 cm^–1^. HRMS (+p ESI) calcd. for C_8_H_9_ClN_3_O_3_P [M + H]^+^ 262.0142, found 262.0148.

#### Dimethyl (Diazo­(phenyl)­methyl)­phosphonate (**3b**)

The material was purified by flash chromatography (SiO_2_; hexanes/EtOAc, 9:1 to 1:1 gradient; *R*_*f*_ = 0.35 in 1:1 hexanes/EtOAc), which afforded the major product **3b** as a yellow oil (486 mg, 88% yield). For a 5-g scale (4.76 g, 86% yield). ^1^H NMR (600 MHz, CDCl_3_) δ 7.35 (t, *J* = 7.7 Hz, 2H), 7.15 (d, *J* = 7.8 Hz, 3H), 3.80 (d, *J*_HP_ = 11.9 Hz, 6H). (Coupling constants are uncorrected). ^13^C­{^1^H} NMR (151 MHz, CDCl_3_) δ 129.6, 126.6 (d, *J*_CP_ = 9.4 Hz), 125.6, 122.9 (d, *J*_CP_ = 4.4 Hz), 53.4 (d, *J*_CP_ = 5.0 Hz), 50.1 (d, *J*_CP_ = 228.6 Hz). ^31^P NMR (243 MHz, CDCl_3_) δ 20.89. Spectroscopic data are in agreement with those reported in the literature.^18,30,31,81^

#### Dimethyl ((4-Bromophenyl)­(diazo)­methyl)­phosphonate (**3c**)^18,30,31,81^

The material was purified by flash chromatography (SiO_2_; hexanes/EtOAc, 9:1 to 1:1 gradient; *R*_*f*_ = 0.35 in 1:1 hexanes/EtOAc), which afforded the major product **3c** as a yellow oil (485 mg, 90% yield). ^1^H NMR (600 MHz, CDCl_3_) δ 7.46 (d, *J* = 8.6 Hz, 2H), 7.02 (d, *J* = 8.7 Hz, 2H), 3.80 (d, *J*_HP_ = 11.9 Hz, 6H). (Coupling constants are uncorrected). ^13^C­{^1^H} NMR (151 MHz, CDCl_3_) δ 132.7, 126.0 (d, *J*_CP_ = 9.9 Hz), 124.4 (d, *J*_CP_ = 4.6 Hz), 119.0, 53.6 (d, *J*_CP_ = 5.1 Hz), 50.2 (d, *J*_CP_ = 228.9 Hz). ^31^P NMR (243 MHz, CDCl_3_) δ 20.00.

#### Diethyl (Diazo­(phenyl)­methyl)­phosphonate (**3d**)

The material was purified by flash chromatography (SiO_2_; hexanes/EtOAc, 9:1 to 1:1 gradient; *R*_*f*_ = 0.35 in 1:1 hexanes/EtOAc), which afforded the major product **3d** as a yellow oil (487 mg, 83% yield). ^1^H NMR (400 MHz, CDCl_3_) δ 7.41 (d, *J* = 8.6 Hz, 2H), 7.01 (d, *J* = 8.7 Hz, 2H), 4.31–3.96 (m, 4H), 1.29 (t, *J*_HP_ = 7.1 Hz, 6H). (Coupling constants are uncorrected). ^13^C­{^1^H} NMR (101 MHz, CDCl_3_) δ 132.5, 126.2 (d, *J*_CP_ = 9.8 Hz), 124.4 (d, *J*_CP_ = 4.5 Hz), 118.7, 63.3 (d, *J*_CP_ = 5.1 Hz), 50.9 (d, *J*_CP_ = 226.3 Hz), 16.4 (d, *J*_CP_ = 6.8 Hz). ^31^P NMR (162 MHz, CDCl_3_) δ 16.40. Spectroscopic data are in agreement with those reported in the literature.^82^

#### Dimethyl (Diazo­(3,5-difluorophenyl)­methyl)­phosphonate (**3e**)

The material was purified by flash chromatography (SiO_2_; hexanes/EtOAc, 9:1 to 1:1 gradient; *R*_*f*_ = 0.35 in 1:1 hexanes/EtOAc), which afforded major product **3e** as a yellow oil (495 mg, 91% yield). ^1^H NMR (400 MHz, CDCl_3_) δ 6.70–6.63 (m, 2H), 6.55 (tt, *J*_HF_ = 2.2, 8.8 Hz, 1H), 3.81 (d, *J*_HP_ = 12.0 Hz, 6H). (Coupling constants are uncorrected). ^13^C­{^1^H} NMR (101 MHz, CDCl_3_) δ 163.9 (dd, *J*_CF_ = 13.8, 248.4 Hz), 131.2 (q, *J*_CF_ = 11.1 Hz), 105.9–105.42 (m), 100.8 (t, *J*_CF_ = 25.6 Hz), 53.7 (d, *J*_CP_ = 5.2 Hz), 51.4 (d, *J*_CP_ = 229.1 Hz). ^31^P NMR (162 MHz, CDCl_3_) δ 18.65. ^19^F NMR (376 MHz, CDCl_3_) δ −108.19. IR (neat): 2983, 2907, 2075, 1621, 1585 cm^–1^. HRMS (+p ESI) calcd. for C_9_H_9_F_2_N_2_O_3_P [M + H]^+^ 263.0392, found 263.0391.

#### Dimethyl (Benzo­[*d*]­[1,3]­dioxol-5-yl­(diazo)­methyl)­phosphonate (**3f**)

The material was purified by flash chromatography (SiO_2_; hexanes/EtOAc, 9:1 to 1:1 gradient; *R*_*f*_ = 0.30 in 1:1 hexanes/EtOAc), which afforded the major product **3f** as a yellow oil (437 mg, 80% yield). ^1^H NMR (600 MHz, CDCl_3_) δ 6.82 (d, *J*_HP_ = 8.3 Hz, 1H), 6.69 (s, 1H), 6.63 (d, *J*_HP_ = 8.2 Hz, 1H), 5.96 (s, 2H), 3.80 (d, *J*_HP_ = 12.0 Hz, 6H). (Coupling constants are uncorrected). ^13^C­{^1^H} NMR (151 MHz, CDCl_3_) δ 149.1, 146.1, 119.6 (d, *J*_CP_ = 9.8 Hz), 116.8 (d, *J*_CP_ = 4.7 Hz), 109.6, 104.4 (d, *J*_CP_ = 4.4 Hz), 101.7, 53.5 (d, *J*_CP_ = 5.0 Hz), 49.7 (d, *J*_CP_ = 230.0 Hz). ^31^P NMR (243 MHz, CDCl_3_) δ 21.22. IR (neat): 2953, 2077, 1608, 1504 cm^–1^. HRMS (+p ESI) calcd. for C_10_H_11_N_2_O_5_P [M + H – N_2_]^+^, 243.0415, found 243.0412.

#### Dimethyl (Diazo­(4-(4,4,5,5-tetramethyl-1,3,2-dioxaborolan-2-yl)­phenyl)­methyl)­phosphonate (**3g**)

The material was purified by flash chromatography (SiO_2_; hexanes/EtOAc, 9:1 to 1:1 gradient; *R*_*f*_ = 0.35 in 1:1 hexanes/EtOAc), which afforded the major product **3g** as an orange solid (465 mg, 85% yield). mp 103–106 °C. ^1^H NMR (400 MHz, CDCl_3_) δ 7.78 (d, *J* = 8.2 Hz, 2H), 7.14 (d, *J* = 8.5 Hz, 2H), 3.80 (d, *J*_HP_ = 12.0 Hz, 6H), 1.33 (s, 12H). (Coupling constants are uncorrected). ^13^C­{^1^H} NMR (101 MHz, CDCl_3_) δ 136.0, 130.0 (d, *J*_CP_ = 9.5 Hz), 129.7, 121.9 (d, *J*_CP_ = 4.5 Hz), 84.17, 53.5 (d, *J*_CP_ = 5.0 Hz), 51.0 (d, *J*_CP_ = 227.6 Hz), 25.2. ^31^P NMR (162 MHz, CDCl_3_) δ 20.39. IR (neat): 2977, 2852, 2080, 1605, 1548 cm^–1^. HRMS (+p ESI) calcd. for C_15_H_22_BN_2_O_5_P [M + H]^+^ 352.1469, found 352.1473.

#### Dimethyl (Diazo­(3-((5-(4-fluorophenyl)­thiophen-2-yl)­methyl)-4-methylphenyl)­methyl)­phosphonate (**3h**)

The material was purified by flash chromatography (SiO_2_; hexanes/EtOAc, 9:1 to 1:1 gradient; *R*_*f*_ = 0.30 in 1:1 hexanes/EtOAc), which afforded the major product **3h** as a yellow oil (397 mg, 75% yield). ^1^H NMR (600 MHz, CDCl_3_) δ 7.47 (d, *J* = 7.0 Hz, 2H), 7.17 (d, *J* = 7.8 Hz, 1H), 7.06–6.97 (m, 5H), 6.68 (d, *J* = 3.7 Hz, 1H), 4.11 (s, 2H), 3.79 (d, *J*_HP_ = 11.8 Hz, 6H), 2.30 (s, 3H). (Coupling constants are uncorrected). ^13^C­{^1^H} NMR (151 MHz, CDCl_3_) δ 162.4 (d, *J*_CF_ = 246.9 Hz), 143.1, 142.0, 139.7, 134.0, 131.8, 131.1 (d, *J*_CP_ = 3.5 Hz), 127.4 (d, *J*_CP_ = 8.0 Hz), 126.4, 124.1, 124.0 (d, *J*_CP_ = 4.4 Hz), 123.0, 121.6 (d, *J*_CP_ = 4.4 Hz), 116.1 (d, *J*_CF_ = 21.7 Hz), 53.4 (d, *J*_CP_ = 5.0 Hz), 49.7 (d, *J*_CP_ = 229.1 Hz), 34.5, 19.3. ^31^P NMR (243 MHz, CDCl_3_) δ 21.16. ^19^F NMR (565 MHz, CDCl_3_) δ −114.99. IR (neat): 2952, 2849, 2241, 2072, 1595, 1500 cm^–1^. HRMS (+p ESI) calcd. for C_21_H_20_FN_2_O_3_PS [M + Na]^+^ 453.0809, found 453.0804.

#### Dimethyl ((2-Chloropyrimidin-5-yl)­(diazo)­methyl)­phosphonate (**3i**)

The material was purified by flash chromatography (SiO_2_; hexanes/EtOAc, 9:1 to 1:1 gradient; *R*_*f*_ = 0.20 in 1:1 hexanes/EtOAc), which afforded the major product **3i** as a yellow oil (218 mg, 40% yield). ^1^H NMR (400 MHz, CDCl_3_) δ 8.45 (s, 2H), 3.86 (d, *J*_HP_ = 11.9 Hz, 6H). (Coupling constants are uncorrected). ^13^C­{^1^H} NMR (101 MHz, CDCl_3_) δ 158.1, 153.1 (d, *J*_CP_ = 3.9 Hz), 122.3 (d, *J*_CP_ = 10.2 Hz), 55.7 (dd, *J*_CP_ = 6.0, 186.4 Hz), 54.0 (d, *J*_CP_ = 5.5 Hz). ^31^P NMR (162 MHz, CDCl_3_) δ 16.98. IR (neat): 2956, 1085, 1667, 1549 cm^–1^. HRMS (+p APCI) calcd. for C_7_H_8_ClN_4_O_3_P [M + H]^+^ 263.0095, found 263.0102.

#### Dimethyl (Diazo­(6-methoxypyridin-3-yl)­methyl)­phosphonate (**3j**)

The material was purified by flash chromatography (SiO_2_; hexanes/EtOAc, 9:1 to 1:1 gradient; *R*_*f*_ = 0.15 in 1:1 hexanes/EtOAc), which afforded the major product **3j** as a yellow oil (327 mg, 60% yield). ^1^H NMR (600 MHz, CDCl_3_) δ 8.02 (s, 1H), 7.40 (dd, *J* = 2.6, 8.8 Hz, 1H), 6.77 (d, *J* = 8.7 Hz, 1H), 3.92 (d, *J* = 1.7 Hz, 3H), 3.81 (d, *J*_HP_ = 11.9 Hz, 6H). (Coupling constants are uncorrected). ^13^C­{^1^H} NMR (151 MHz, CDCl_3_) δ 162.9, 142.0 (d, *J*_CP_ = 4.3 Hz), 134.3 (d, *J*_CP_ = 4.3 Hz), 115.6 (d, *J*_CP_ = 9.9 Hz), 112.0, 53.9, 53.6 (d, *J*_CP_ = 5.4 Hz), 46.8 (d, *J*_CP_ = 232.0 Hz). ^31^P NMR (243 MHz, CDCl_3_) δ 20.50. IR (neat): 2952, 2849, 2241, 2072, 1595, 1500 cm^–1^. HRMS (+p APCI) calcd. for C_9_H_12_N_3_O_4_P [M + H]^+^ 258.0638, found 258.0644.

#### Dimethyl (Diazo­(4-morpholinophenyl)­methyl)­phosphonate (**3k**)

The material was purified by flash chromatography (SiO_2_; hexanes/EtOAc, 9:1 to 1:1 gradient; *R*_*f*_ = 0.35 in 1:1 hexanes/EtOAc), which afforded the major product **3k** as a yellow oil (297 mg, 55% yield). ^1^H NMR (400 MHz, CDCl_3_) δ 7.09 (d, *J* = 8.7 Hz, 2H), 6.93 (d, *J* = 8.9 Hz, 2H), 3.86 (t, *J* = 4.9 Hz, 4H), 3.80 (d, *J*_HP_ = 11.9 Hz, 6H), 3.14 (t, *J* = 4.9 Hz, 4H). (Coupling constants are uncorrected). ^13^C­{^1^H} NMR (101 MHz, CDCl_3_) δ 149.6, 124.5 (d, *J*_CP_ = 4.4 Hz), 117.1, 116.7 (d, *J*_CP_ = 9.6 Hz), 67.2, 53.4 (d, *J*_CP_ = 5.1 Hz), 49.6. ^31^P NMR (162 MHz, CDCl_3_) δ 21.82. IR (neat): 2955, 2852, 2080, 1608, 1515 cm^–1^. HRMS (+p ESI) calcd. for C_13_H_18_N_3_O_4_P [M + H]^+^ 312.1108, found 312.1115.

#### *tert*-Butyl 5-(Diazo­(dimethoxyphosphoryl)­methyl)-1*H*-indole-1-carboxylate (**3l**)

The material was purified by flash chromatography (SiO_2_; hexanes/EtOAc, 9:1 to 1:1 gradient; *R*_*f*_ = 0.30 in 1:1 hexanes/EtOAc), which afforded the major product **3l** as an orange solid (319 mg, 60% yield). mp 101–104 °C. ^1^H NMR (600 MHz, CDCl_3_) δ 8.12 (s, 1H), 7.59 (s, 1H), 7.38 (d, *J* = 2.0 Hz, 1H), 7.10 (dd, *J* = 2.0, 8.8 Hz, 1H), 6.52 (dd, *J* = 0.8, 3.7 Hz, 1H), 3.82 (d, *J*_HP_ = 11.9 Hz, 6H), 1.67 (s, 9H). (Coupling constants are uncorrected). ^13^C­{^1^H} NMR (151 MHz, CDCl_3_) δ 149.9, 133.6, 131.9, 127.2, 120.4 (d, *J* = 9.5 Hz), 119.7 (d, *J* = 4.8 Hz), 116.5, 115.7 (d, *J*_CP_ = 4.2 Hz), 107.3, 84.3, 53.5 (d, *J*_CP_ = 5.0 Hz), 49.6 (d, *J*_CP_ = 228.6 Hz), 28.5. ^31^P NMR (243 MHz, CDCl_3_) δ 21.67. IR (neat): 2978, 2074, 1732, 1575 cm^–1^. HRMS (+p APCI) calcd. for C_16_H_20_N_3_O_5_P [M + H]^+^ 366.1213, found 366.1212.

#### Methyl (*R*)-2-((*tert*-Butoxycarbonyl)­amino)-3-(4-(diazo­(dimethoxyphosphoryl)­methyl)­phenyl)­propanoate (**3m**)

The material was purified by flash chromatography (SiO_2_; hexanes/EtOAc, 9:1 to 1:1 gradient; *R*_*f*_ = 0.25 in 1:1 hexanes/EtOAc), which afforded the major product **3m** as a yellow oil (279 mg, 53% yield). ^1^H NMR (600 MHz, CDCl_3_) δ 7.13 (d, *J* = 8.0 Hz, 2H), 7.10 (d, *J* = 8.8 Hz, 2H), 5.01 (d, *J* = 8.3 Hz, 1H), 4.59 (d, *J* = 7.1 Hz, 1H), 3.83 (d, *J*_HP_ = 11.9 Hz, 6H), 3.74 (s, 3H), 3.12 (dd, *J* = 5.6, 14.0 Hz, 1H), 3.03 (dd, *J* = 6.1, 14.0 Hz, 1H), 1.43 (s, 9H). (Coupling constants are uncorrected). ^13^C­{^1^H} NMR (151 MHz, CDCl_3_) δ 172.5, 155.4, 133.5, 130.6, 125.3 (d, *J*_CP_ = 9.6 Hz), 123.1 (d, *J*_CP_ = 4.4 Hz), 80.3, 54.7, 53.5 (d, *J*_CP_ = 5.0 Hz), 52.6, 50.0 (d, *J*_CP_ = 228.8 Hz), 38.1, 28.6. ^31^P NMR (243 MHz, CDCl_3_) δ 20.81. IR (neat): 3301, 2954, 2077, 1745, 1711, 1610, 1513 cm^–1^. HRMS (+p APCI) calcd. for C_18_H_26_N_3_O_7_P [M + H]^+^ 428.1581, found 428.1583.

#### Dimethyl (Diazo­(2,6-dichloropyridin-4-yl)­methyl)­phosphonate (**3n**)

The material was purified by flash chromatography (SiO_2_; hexanes/EtOAc, 9:1 to 1:1 gradient; *R*_*f*_ = 0.20 in 1:1 hexanes/EtOAc), which afforded major product **3n** as a yellow oil (292 mg, 54% yield). ^1^H NMR (600 MHz, CDCl_3_) δ 6.98 (s, 2H), 3.84 (d, *J*_HP_ = 11.9 Hz, 6H). (Coupling constants are uncorrected). ^13^C­{^1^H} NMR (151 MHz, CDCl_3_) δ 151.5, 143.3 (d, *J*_CP_ = 10.3 Hz), 115.2 (d, *J*_CP_ = 4.5 Hz), 54.0 (d, *J*_CP_ = 5.1 Hz), 52.4 (d, *J*_CP_ = 227.4 Hz). ^31^P NMR (243 MHz, CDCl_3_) δ 15.87. IR (neat): 2955, 2097, 1573, 1521 cm^–1^. HRMS (+p APCI) calcd. for C_8_H_8_Cl_2_N_3_O_3_P [M + H]^+^ 295.9753, found 295.9753.

#### Dimethyl (Diazo­(9-phenyl-9*H*-carbazol-3-yl)­methyl)­phosphonate (**3o**)

The material was purified by flash chromatography (SiO_2_; hexanes/EtOAc, 9:1 to 1:1 gradient; *R*_*f*_ = 0.30 in 1:1 hexanes/EtOAc), which afforded the major product **3o** as a yellow oil (354 mg, 67% yield). ^1^H NMR (800 MHz, CDCl_3_) δ 8.15 (d, *J* = 7.8 Hz, 1H), 7.95 (d, *J* = 1.9 Hz, 1H), 7.61 (t, *J* = 7.7 Hz, 2H), 7.55 (d, *J* = 8.2 Hz, 2H), 7.48 (t, *J* = 7.5 Hz, 1H), 7.45–7.38 (m, 3H), 7.30 (t, *J* = 7.3 Hz, 1H), 7.25 (dd, *J* = 8.6, 2.0 Hz, 1H), 3.89 (d, *J*_HP_ = 11.9 Hz, 6H). (Coupling constants are uncorrected). ^13^C­{^1^H} NMR (201 MHz, CDCl_3_) δ 141.5, 139.5, 137.7, 130.3, 127.9, 127.3, 126.8, 124.7, 122.9, 121.9 (d, *J*_CP_ = 3.9 Hz), 120.8, 120.5, 117.1 (d, *J*_CP_ = 9.6 Hz), 115.4 (d, *J*_CP_ = 4.4 Hz), 111.2, 110.2, 53.5 (d, *J*_CP_ = 5.1 Hz), 49.5 (d, *J*_CP_ = 229.3 Hz). ^31^P NMR (243 MHz, CDCl_3_) δ. 22.11. IR (neat): 3061, 2951, 2849, 2072, 1595, 1501 cm^–1^. HRMS (+p ESI) calcd. for C_21_H_18_N_3_O_3_P [M + H – N_2_]^+^ 364.1097, found 364.1097.

### General Procedure for Cyclopropanation with Diazophosphonates

A clean, oven-dried, and flame-dried 25.0 mL round-bottom flask (flask-A) equipped with activated 4 Å molecular sieves and a magnetic stir bar was evacuated and purged with argon (2–3 times). After cooling down to room temperature, styrene (5 equiv, 1.00 mmol) followed by Rh_2_(Cat.) (0.01 equiv) was then added. The flask was once again evacuated and purged with argon (3–5 times) and anhydrous CH_2_Cl_2_ (3.0 mL) was added. The flask and its contents were then set to stir at room temperature using a hot plate or using a heating mantle under an argon atmosphere. To a second oven-dried round-bottom flask (flask-B), which was evacuated and purged with argon, diazophosphonates (0.20 mmol) were added. Flask-B and its contents were evacuated and purged with argon (2–3 times) and anhydrous CH_2_Cl_2_ (10.0 mL) was then added to obtain a solution of the diazo compound. The solution was transferred into a 12.0 mL plastic syringe. Using a well-calibrated syringe pump, a slow addition of the diazo solution into the stirring solution of flask-A under an inert atmosphere was initiated. After the complete addition of the solution (5 h), the residual diazo compound in the 12.0 mL plastic syringe was rinsed with anhydrous CH_2_Cl_2_ (1.0 mL) and transferred dropwise into the stirring reaction mixture of flask-A. The resulting solution was stirred for 5 to 7 h before concentrating the solution under reduced pressure. Purification by flash column chromatography on silica gel (hexanes:EtOAc) was used to afford the final products (**6a–o**).

#### Dimethyl ((1*S*,2*R*)-1,2-Diphenylcyclopropyl)­phosphonate (**6a**)

The material was purified by flash chromatography (SiO_2_; hexanes/EtOAc, 9:1 to 1:1 gradient; *R*_*f*_ = 0.20 in 1:1 hexanes/EtOAc), which afforded the major product **6a** as a colorless oil (55 mg, 92% yield). Spectroscopic data are in agreement with those reported in the literature. ^1^H NMR (600 MHz, CDCl_3_) δ 7.12 (dd, *J* = 2.0, 5.2 Hz, 3H), 7.10–7.02 (m, 5H), 6.78–6.72 (m, 2H), 3.74 (d, *J*_HP_ = 10.6 Hz, 3H), 3.69 (d, *J*_HP_ = 10.6 Hz, 3H), 3.02 (ddd, *J*_HP_ = 6.6, 9.0, 16.6 Hz, 1H), 2.07 (ddd, *J*_HP_ = 5.2, 9.0, 17.4 Hz, 1H), 1.74 (ddd, *J*_HP_ = 5.2, 6.6, 12.5 Hz, 1H). (Coupling constants are uncorrected). ^13^C­{^1^H} NMR (151 MHz, CDCl_3_) δ 136.3 (d, *J*_CP_ = 2.7 Hz), 133.9 (d, *J*_CP_ = 2.0 Hz), 132.5 (d, *J*_CP_ = 4.0 Hz), 128.3, 128.2 (d, *J*_CP_ = 2.5 Hz), 128.0, 127.5 (d, *J*_CP_ = 2.8 Hz), 126.6, 53.5, 29.7 (d, *J*_CP_ = 186.6 Hz), 27.3 (d, *J*_CP_ = 1.9 Hz), 16.9 (d, *J*_CP_ = 3.3 Hz). ^31^P NMR (243 MHz, CDCl_3_) δ 29.13. Spectroscopic data are in agreement with those reported in the literature.^30,31^ SFC analysis: (OJ-3, 3 μm particle size, 150 mm × 3 mm, 20.0% IPA/Heptane, with 0.2% Formic Acid, 2.5 mL/min, 254 nm) indicated 96% ee: *t*_R_ (minor enantiomer) = 0.79 min, *t*_R_ (major enantiomer) = 0.87 min. [α]^20^ D: −50.1° (c 0.60, CHCl_3_).

#### Dimethyl ((1*S*,2*R*)-1-(4-Bromophenyl)-2-phenylcyclopropyl)­phosphonate (**6b**)

The material was purified by flash chromatography (SiO_2_; hexanes/EtOAc, 9:1 to 1:1 gradient; *R*_*f*_ = 0.24 in 1:1 hexanes/EtOAc), which afforded the major product **6b** as a colorless oil (61 mg, 80% yield). Spectroscopic data are in agreement with those reported in the literature. ^1^H NMR (600 MHz, CDCl_3_) δ 7.25 (d, *J* = 12.4 Hz, 2H), 7.09 (m, 2H), 6.92 (d, *J* = 8.3 Hz, 2H), 6.76 (d, *J* = 7.3 Hz, 2H), 3.75 (dd, *J*_HP_ = 1.7, 10.6 Hz, 3H), 3.70 (dd, *J*_HP_ = 1.7, 10.5 Hz, 3H), 3.03 (ddd, *J*_HP_ = 6.6, 9.0, 16.2 Hz, 1H), 2.06 (ddd, *J*_HP_ = 5.3, 8.9, 16.6 Hz, 1H), 1.70 (ddd, *J*_HP_ = 6.0, 8.8, 12.2 Hz, 1H). (Coupling constants are uncorrected). ^13^C­{^1^H} NMR (151 MHz, CDCl_3_) δ 135.8 (d, *J*_CP_ = 2.6 Hz), 134.2 (d, *J*_CP_ = 3.8 Hz), 133.2, 131.4 (d, *J*_CP_ = 2.3 Hz), 128.3 (d, *J*_CP_ = 7.2 Hz), 126.9, 121.8 (d, *J*_CP_ = 3.4 Hz), 53.6 (dd, *J*_CP_ = 6.5, 10.1 Hz), 29.2 (d, *J*_CP_ = 187.8 Hz), 27.4, 16.7 (d, *J*_CP_ = 3.0 Hz). ^31^P NMR (243 MHz, CDCl_3_) 28.46. Spectroscopic data are in agreement with those reported in the literature.^30,31^ SFC analysis: (OJ-3, 3 μm particle size, 150 mm × 3 mm, 20.0% IPA/Heptane, with 0.2% Formic Acid, 2.5 mL/min, 230 nm) indicated 98% ee: *t*_R_ (minor enantiomer) = 1.07 min, *t*_R_ (major enantiomer) = 1.23 min. The crystal structure information for compound (4.39b) is located in the crystallography section. [α]^20^ D: −65.7° (c 2.00, CHCl_3_).

#### Diethyl ((1*S*,2*R*)-1-(4-Bromophenyl)-2-phenylcyclopropyl)­phosphonate (**6c**)

The material was purified by flash chromatography (SiO_2_; hexanes/EtOAc, 9:1 to 1:1 gradient; *R*_*f*_ = 0.24 in 1:1 hexanes/EtOAc), which afforded the major product **6c** as a colorless oil (70 mg, 85% yield). ^1^H NMR (600 MHz, CDCl_3_) δ 7.24 (d, *J* = 8.1 Hz, 2H), 7.12–7.06 (m, 3H), 6.93 (d, *J* = 6.9 Hz, 2H), 6.79–6.72 (m, 2H), 4.14–3.99 (m, 4H), 3.01 (ddd, *J*_HP_ = 6.6, 8.8, 16.1 Hz, 1H), 2.05 (ddd, *J*_HP_ = 5.2, 8.9, 17.2 Hz, 1H), 1.72–1.60 (m, 1H), 1.29 (t, J = 7.1 Hz, 3H), 1.25 (t, J = 7.0 Hz, 3H). (Coupling constants are uncorrected). ^13^C­{^1^H} NMR (151 MHz, CDCl_3_) δ 136.1 (d, *J*_CP_ = 2.5 Hz), 134.3 (d, *J*_CP_ = 4.0 Hz), 133.5, 131.3 (d, *J*_CP_ = 2.2 Hz), 128.3, 128.2, 126.8, 121.6 (d, *J*_CP_ = 3.5 Hz), 62.9 (t, *J*_CP_ = 6.0 Hz), 30.0 (d, *J*_CP_ = 187.0 Hz), 27.5, 16.9 (d, *J*_CP_ = 3.3 Hz), 16.8 (t, *J*_CP_ = 6.1 Hz). ^31^P NMR (243 MHz, CDCl_3_) δ 25.60. Spectroscopic data are in agreement with those reported in the literature.^30,31^ SFC analysis: (OJ-3, 3 μm particle size, 150 mm × 3 mm, 20.0% IPA/Heptane, with 0.2% Formic Acid, 2.5 mL/min, 230 nm) indicated 90% ee: *t*_R_ (major enantiomer) = 1.93 min, *t*_R_ (minor enantiomer) = 3.96 min. [α]^20^ D: −48.8° (c 0.60, CHCl_3_).

#### Dimethyl ((1*S*,2*R*)-1-(3,5-Difluorophenyl)-2-phenylcyclopropyl)­phosphonate (**6d**)

The material was purified by flash chromatography (SiO_2_; hexanes/EtOAc, 9:1 to 1:1 gradient; *R*_*f*_ = 0.20 in 1:1 hexanes/EtOAc), which afforded major product **6d** as a colorless oil (53 mg, 78% yield). ^1^H NMR (400 MHz, CDCl_3_) δ 7.12 (dd, *J* = 1.9, 5.0 Hz, 3H), 6.79 (dd, *J* = 4.3, 7.6 Hz, 2H), 6.65–6.53 (m, 3H), 3.79 (d, *J*_HP_ = 10.7 Hz, 3H), 3.73 (d, *J*_HP_ = 10.7 Hz, 3H), 3.04 (ddd, *J*_HP_ = 6.7, 9.0, 16.1 Hz, 1H), 2.06 (ddd, *J*_HP_ = 5.4, 9.0, 17.1 Hz, 1H), 1.74 (ddd, *J*_HP_ = 5.4, 6.7, 12.2 Hz, 1H). (Coupling constants are uncorrected). ^13^C­{^1^H} NMR (101 MHz, CDCl_3_) δ 162.6 (ddd, *J*_CF_ = 2.6, 13.1, 248.2 Hz), 138.3–138.0 (m), 135.2 (d, *J*_CP_ = 2.6 Hz), 128.4, 128.2, 127.2, 115.8–115.0 (m), 103.4 (td, *J*_CF_ = 2.7, 25.2 Hz), 53.7 (t, *J*_CP_ = 6.4 Hz), 29.4 (d, *J*_CP_ = 188.2 Hz), 27.8 (d, *J*_CP_ = 2.0 Hz), 16.5 (d, *J*_CP_ = 3.2 Hz). ^19^F NMR (565 MHz, CDCl_3_) δ −110.39. ^31^P NMR (243 MHz, CDCl_3_) δ 27.80. IR (neat): 3468, 2954, 2851, 2239, 2151, 1622, 1595 cm^–1^. HRMS (+p APCl) calcd. for C_17_H_17_F_2_O_3_P [M + H]^+^ 339.0956, found 339.0957. HPLC analysis: (Chiralcel AS-H, 25 cm × 4.6 mm, 3.0% i-PrOH/hexanes, 1.0 mL/min, 210 nm) indicated 90% ee: *t*_R_ (major enantiomer) = 21.37 min, *t*_R_ (minor enantiomer) = 53.41 min. [α]^20^ D: −26.6° (c 0.72, CHCl_3_).

#### Dimethyl ((1*S*,2*R*)-1-(Benzo­[*d*]­[1,3]­dioxol-5-yl)-2-phenylcyclopropyl)­phosphonate (**6e**)

The material was purified by flash chromatography (SiO_2_; hexanes/EtOAc, 9:1 to 1:1 gradient; *R*_*f*_ = 0.16 in 1:1 hexanes/EtOAc), which afforded the major product **6e** as a colorless oil (58 mg, 84% yield). ^1^H NMR (600 MHz, CDCl_3_) δ 7.15–7.05 (m, 3H), 6.79 (dd, *J* = 2.6, 7.1 Hz, 2H), 6.64–6.46 (m, 3H), 5.87 (d, *J* = 5.2 Hz, 2H), 3.76 (d, *J*_HP_ = 10.6 Hz, 3H), 3.72 (d, *J*_HP_ = 10.6 Hz, 3H), 2.97 (ddd, *J*_HP_ = 6.6, 9.0, 16.1 Hz, 1H), 2.03 (ddd, *J*_HP_ = 5.2, 9.0, 17.3 Hz, 1H), 1.68 (ddd, *J*_HP_ = 5.9, 9.0, 12.1 Hz, 1H). (Coupling constants are uncorrected). ^13^C­{^1^H} NMR (151 MHz, CDCl_3_) δ 147.4 (d, *J*_CP_ = 2.4 Hz), 147.1 (d, *J*_CP_ = 3.0 Hz), 136.2, 128.4, 128.2, 126.8, 126.1 (d, *J*_CP_ = 4.9 Hz), 112.7 (d, *J*_CP_ = 3.8 Hz), 108.1 (d, *J*_CP_ = 2.7 Hz), 101.3, 53.7 (dd, *J*_CP_ = 6.5, 16.4 Hz), 31.3 (d, *J*_CP_ = 285.8 Hz), 30.1, 27.5, 23.1. ^31^P NMR (243 MHz, CDCl_3_) δ 29.27. IR (neat): 3459, 2952, 2922, 2852, 1605, 1502 cm^–1^. HRMS (+p APCl) calcd. for C_18_H_19_O_5_P [M + H]^+^ 347.1043, found 347.1042. SFC analysis: (AS-3, 3 μm particle size, 150 mm × 3 mm, 20.0% IPA/Heptane, with 0.2% Formic Acid, 2.5 mL/min, 230 nm) indicated 97% ee: *t*_R_ (major enantiomer) = 1.73 min, *t*_R_ (minor enantiomer) = 2.93 min. [α]^20^ D: −44.3° (c 0.60, CHCl_3_).

#### Dimethyl ((1*S*,2*R*)-2-Phenyl-1-(4-(4,4,5,5-tetramethyl-1,3,2-dioxaborolan-2-yl)­phenyl)­cyclopropyl)­phosphonate (**6f**)

The material was purified by flash chromatography (SiO_2_; hexanes/EtOAc, 9:1 to 1:1 gradient; *R*_*f*_ = 0.20 in 1:1 hexanes/EtOAc), which afforded the major product **6f** as a colorless oil (64 mg, 75% yield). ^1^H NMR (600 MHz, CDCl_3_) δ 7.56 (d, *J* = 7.6 Hz, 2H), 7.06 (dt, *J* = 2.3, 6.2 Hz, 5H), 6.78–6.74 (m, 2H), 3.73 (d, *J*_HP_ = 10.5 Hz, 3H), 3.67 (d, *J*_HP_ = 10.4 Hz, 3H), 3.03 (ddd, *J*_HP_ = 6.6, 8.9, 16.2 Hz, 1H), 2.08 (ddd, *J*_HP_ = 5.1, 8.9, 17.4 Hz, 1H), 1.74 (ddd, *J*_HP_ = 5.8, 8.9, 12.3 Hz, 1H), 1.30 (s, 12H). (Coupling constants are uncorrected). ^13^C­{^1^H} NMR (151 MHz, CDCl_3_) δ 137.2, 136.1 (d, *J*_CP_ = 2.4 Hz), 134.6 (d, *J*_CP_ = 2.2 Hz), 131.9 (d, *J*_CP_ = 3.7 Hz), 128.3, 128.1, 126.7, 84.1, 53.7 (d, *J*_CP_ = 6.5 Hz), 53.6 (d, *J*_CP_ = 6.5 Hz), 29.9 (d, *J*_CP_ = 186.0 Hz), 27.4, 25.2 (d, *J*_CP_ = 20.3 Hz), 16.9 (d, *J*_CP_ = 3.3 Hz). ^31^P NMR (243 MHz, CDCl_3_) δ 28.91. IR (neat): 2978, 1609, 1499, 1457 cm^–1^. HRMS (+p APCl) calcd. for C_23_H_30_BO_5_P [M + H]^+^ 428.2033, found 428.2035. SFC analysis: (OJ-3, 3 μm particle size, 150 mm × 3 mm, 20.0% IPA/Heptane, with 0.2% Formic Acid, 2.5 mL/min, 230 nm) indicated 86% ee: *t*_R_ (major enantiomer) = 1.37 min, *t*_R_ (minor enantiomer) = 2.21 min. [α]^20^ D: −56.8° (c 1.00, CHCl_3_).

#### Dimethyl ((1*S*,2*R*)-1-(3-((5-(4-Fluorophenyl)­thiophen-2-yl)­methyl)-4-methylphenyl)-2-phenylcyclopropyl)­phosphonate (**6g**)

The material was purified by flash chromatography (SiO_2_; hexanes/EtOAc, 9:1 to 1:1 gradient; *R*_*f*_ = 0.18 in 1:1 hexanes/EtOAc), which afforded the major product **6g** as a colorless oil (69 mg, 68% yield). ^1^H NMR (600 MHz, CDCl_3_) δ 7.47 (dd, J = 5.3, 8.8 Hz, 2H), 7.09–7.01 (m, 5H), 6.98–6.92 (m, 3H), 6.90 (s, 1H), 6.79 (d, *J* = 8.1 Hz, 2H), 6.29 (d, *J* = 3.5 Hz, 1H), 3.94 (s, 2H), 3.73 (d, *J* = 10.5 Hz, 3H), 3.69 (d, *J* = 10.3 Hz, 3H), 3.00 (ddd, *J* = 6.6, 8.9, 16.1 Hz, 1H), 2.19 (s, 3H), 2.05 (ddd, *J* = 5.2, 9.0, 17.3 Hz, 1H), 1.74 (ddd, *J* = 5.2, 6.6, 12.2 Hz, 1H). (Coupling constants are uncorrected). ^13^C­{^1^H} NMR (151 MHz, CDCl_3_) δ 162.1 (d, *J*_CF_ = 246.6 Hz), 143.3, 141.3, 137.6 (d, *J*_CP_ = 2.6 Hz), 136.0 (d, *J*_CP_ = 2.4 Hz), 135.5 (d, *J*_CP_ = 3.0 Hz), 133.4 (d, *J*_CP_ = 3.9 Hz), 131.2 (d, *J*_CP_ = 2.1 Hz), 130.9 (d, *J*_CP_ = 3.4 Hz), 130.6 (d, *J*_CP_ = 4.2 Hz), 130.2 (d, *J*_CP_ = 2.4 Hz), 128.1, 127.8, 127.1 (d, *J*_CF_ = 7.9 Hz), 126.3, 125.9, 122.6, 115.8 (d, *J*_CF_ = 22.0 Hz), 53.2 (dd, *J*_CP_ = 6.6, 18.8 Hz), 33.9, 29.6, 28.4, 27.0, 19.1, 16.4 (d, *J*_CP_ = 3.2 Hz). ^31^P NMR (243 MHz, CDCl_3_) δ 29.41. IR (neat): 2985, 1746, 1612, 1508 cm^–1^. HRMS (+p APCl) calcd. for C_29_H_28_FO_3_PS [M + H]^+^ 507.1554, found 507.1556. SFC analysis: (SSWhelk, 3 μm particle size, 150 mm × 3 mm, 20.0% IPA/Heptane, with 0.2% Formic Acid, 2.5 mL/min, 230 nm) indicated 90% ee: *t*_R_ (minor enantiomer) = 7.92 min, *t*_R_ (major enantiomer) = 8.39 min. [α]^20^ D: −28.8° (c 1.00, CHCl_3_).

#### Dimethyl ((1*S*,2*R*)-1-(4-Morpholinophenyl)-2-phenylcyclopropyl)­phosphonate (**6h**)

The material was purified by flash chromatography (SiO_2_; hexanes/EtOAc, 9:1 to 1:1 gradient; *R*_*f*_ = 0.30 in 1:1 hexanes/EtOAc), which afforded major product **6h** as a colorless oil (43 mg, 55% yield). ^1^H NMR (600 MHz, CDCl_3_) δ 7.12–7.03 (m, 3H), 6.94 (dd, *J* = 2.2, 8.8 Hz, 2H), 6.77 (dd, *J* = 2.2, 5.2 Hz, 2H), 6.66 (dd, *J* = 2.1, 8.8 Hz, 2H), 3.80 (t, *J* = 4.8 Hz, 4H), 3.73 (dd, *J*_HP_ = 2.0, 10.5 Hz, 3H), 3.69 (dd, *J*_HP_ = 2.0, 10.5 Hz, 3H), 3.07 (t, *J* = 4.8 Hz, 4H), 2.97 (ddd, *J*_HP_ = 6.7, 9.0, 16.2 Hz, 1H), 2.03 (ddd, *J*_HP_ = 5.6, 9.0, 17.3 Hz, 1H), 1.66 (ddd, *J*_HP_ = 5.8, 9.0, 12.1 Hz, 1H). (Coupling constants are uncorrected). ^13^C­{^1^H} NMR (151 MHz, CDCl_3_) δ 150.4, 136.5, 133.2 (d, *J*_CP_ = 3.9 Hz), 128.4, 128.0, 126.5, 124.6, 115.2, 67.2, 53.6 (dd, *J*_CP_ = 6.6, 13.5 Hz), 49.3, 28.9 (d, *J*_CP_ = 188.2 Hz), 27.3, 17.1 (d, *J*_CP_ = 3.1 Hz). ^31^P NMR (243 MHz, CDCl_3_) δ 29.71. IR (neat): 3460, 2953, 2850, 1610, 1517 cm^–1^. HRMS (+p APCl) calcd. for C_23_H_30_BO_5_P [M + H]^+^ 428.2033, found 428.2035. SFC analysis: (AS-3, 3 μm particle size, 150 mm × 3 mm, 20.0% IPA/Heptane, with 0.2% Formic Acid, 2.5 mL/min, 230 nm) indicated 88% ee: *t*_R_ (major enantiomer) = 3.78 min, *t*_R_ (minor enantiomer) = 5.31 min. [α]^20^ D = −54.2° (c 1.00, CHCl_3_).

#### *tert*-Butyl 5-((1*S*,2*R*)-1-(dimethoxyphosphoryl)-2-phenylcyclopropyl)-1*H*-indole-1-carboxylate (**6i**)

The material was purified by flash chromatography (SiO_2_; hexanes/EtOAc, 9:1 to 1:1 gradient; *R*_*f*_ = 0.35 in 1:1 hexanes/EtOAc), which afforded the major product **6i** as a yellow oil (64 mg, 72% yield). ^1^H NMR (400 MHz, CDCl_3_) δ 7.87 (s, 1H), 7.51 (d, *J* = 3.5 Hz, 1H), 7.28 (t, *J* = 2.1 Hz, 1H), 7.06–7.01 (m, 3H), 7.00 (d, *J* = 8.6 Hz, 1H), 6.76 (dd, *J* = 2.9, 6.7 Hz, 2H), 6.41 (d, *J* = 3.7 Hz, 1H), 3.73 (d, *J*_HP_ = 10.6 Hz, 3H), 3.67 (d, *J*_HP_ = 10.5 Hz, 3H), 3.04 (ddd, *J*_HP_ = 6.6, 9.0, 16.1 Hz, 1H), 2.11 (ddd, *J*_HP_ = 5.1, 9.0, 17.5 Hz, 1H), 1.78 (ddd, *J*_HP_ = 5.1, 6.6, 12.1 Hz, 1H), 1.63 (s, 9H). (Coupling constants are uncorrected). ^13^C­{^1^H} NMR (151 MHz, CDCl_3_) δ 150.0, 136.5 (d, *J*_CP_ = 2.3 Hz), 130.7, 128.7 (d, *J*_CP_ = 3.5 Hz), 128.4, 128.1, 128.0, 126.6, 126.4, 125.1 (d, *J*_CP_ = 4.4 Hz), 114.9 (d, *J*_CP_ = 2.2 Hz), 107.5, 84.0, 53.6 (dd, *J*_CP_ = 6.7, 11.8 Hz), 29.7 (d, *J*_CP_ = 187.2 Hz), 28.5, 27.5, 17.4 (d, *J*_CP_ = 3.1 Hz). ^31^P NMR (243 MHz, CDCl_3_) δ 29.51. IR (neat): 2979, 2951, 1732, 1499 cm^–1^. HRMS (+p APCl) calcd. for C_24_H_28_NO_5_P [M + H]^+^ 442.1778, found 442.1769. SFC analysis: (AS-3, 3 μm particle size, 150 mm × 3 mm, 20.0% IPA/Heptane, with 0.2% Formic Acid, 2.5 mL/min, 230 nm) indicated 66% ee: *t*_R_ (major enantiomer) = 2.07 min, *t*_R_ (minor enantiomer) = 3.11 min. [α]^20^ D = −42.2° (c 1.00, CHCl_3_).

#### Methyl (*R*)-2-((*tert*-Butoxycarbonyl)­amino)-3-(4-((1*S*,2*R*)-1-(dimethoxyphosphoryl)-2-phenylcyclopropyl)­phenyl)­propanoate (**6j**)

The material was purified by flash chromatography (SiO_2_; hexanes/EtOAc, 9:1 to 1:1 gradient; *R*_*f*_ = 0.18 in 1:1 hexanes/EtOAc), which afforded the major product **6j** as a yellow oil (63 mg, 63% yield). ^1^H NMR (400 MHz, CDCl_3_) δ 7.06 (dd, *J* = 1.9, 5.1 Hz, 3H), 6.98 (dd, *J* = 2.1, 8.2 Hz, 2H), 6.88 (d, *J* = 8.0 Hz, 2H), 6.70 (dd, *J* = 1.7, 4.9 Hz, 2H), 4.92 (d, *J* = 8.1 Hz, 1H), 4.50 (q, *J* = 6.5 Hz, 1H), 3.73 (d, *J*_HP_ = 10.6 Hz, 3H), 3.68 (d, *J*_HP_ = 10.6 Hz, 3H), 3.58 (s, 3H), 3.06–2.88 (m, 3H), 2.06 (ddd, *J*_HP_ = 5.2, 9.0, 17.4 Hz, 1H), 1.69 (ddd, *J*_HP_ = 5.2, 6.6, 12.1 Hz, 1H), 1.43 (s, 9H). (Coupling constants are uncorrected). ^13^C­{^1^H} NMR (151 MHz, CDCl_3_) δ 172.6, 172.4, 155.4 (d, *J*_CP_ = 7.3 Hz), 136.2, 135.3 (d, *J*_CP_ = 17.1 Hz), 132.7 (d, *J*_CP_ = 4.0 Hz), 129.1 (d, *J*_CP_ = 2.4 Hz), 128.3 (d, *J*_CP_ = 4.0 Hz), 128.0 (d, *J*_CP_ = 7.1 Hz), 126.7 (d, *J*_CP_ = 11.6 Hz), 80.3 (d, *J*_CP_ = 13.0 Hz), 54.6 (d, *J*_CP_ = 9.7 Hz), 53.7–53.5 (m), 52.4 (d, *J*_CP_ = 5.5 Hz), 38.3 (d, *J*_CP_ = 14.4 Hz), 29.4 (d, *J*_CP_ = 187.4 Hz), 28.7, 27.3, 17.0 (d, *J*_CP_ = 10.6 Hz). ^31^P NMR (243 MHz, CDCl_3_) δ 28.92. IR (neat): 3270, 2953, 2850, 1746, 1711, 1513, 1500 cm^–1^. HRMS (+p APCl) calcd. for C_26_H_34_NO_7_P [M + H]^+^ 503.2067, found 503.2072. [α]^20^ D: −22.9° (c 0.60, CHCl_3_).

#### Dimethyl ((1*S*,2*R*)-1-(6-Chloropyridin-3-yl)-2-phenylcyclopropyl)­phosphonate (**6k**)

The material was purified by flash chromatography (SiO_2_; hexanes/EtOAc, 9:1 to 1:1 gradient; *R*_*f*_ = 0.20 in 1:1 hexanes/EtOAc), which afforded the major product **6k** as a colorless oil (47 mg, 70% yield). ^1^H NMR (600 MHz, CDCl_3_) δ 8.09 (t, *J* = 2.4 Hz, 1H), 7.23 (dt, *J* = 2.2, 8.2 Hz, 1H), 7.12 (dd, *J* = 1.9, 5.0 Hz, 3H), 7.04 (d, *J* = 8.2 Hz, 1H), 6.79 (dd, *J* = 3.2, 6.4 Hz, 2H), 3.75 (dd, *J*_HP_ = 10.7, 13.2 Hz, 6H), 3.09 (ddd, *J*_HP_ = 6.6, 9.0, 16.0 Hz, 1H), 2.11 (ddd, *J*_HP_ = 5.5, 9.0, 17.1 Hz, 1H), 1.76 (ddd, *J*_HP_ = 5.5, 6.7, 12.1 Hz, 1H). (Coupling constants are uncorrected). ^13^C­{^1^H} NMR (151 MHz, CDCl_3_) δ 153.1 (d, *J*_CP_ = 4.5 Hz), 150.5 (d, *J*_CP_ = 3.2 Hz), 142.7 (d, *J*_CP_ = 3.5 Hz), 134.9 (d, *J*_CP_ = 2.5 Hz), 129.7 (d, *J*_CP_ = 2.1 Hz), 128.6, 128.4, 127.4, 123.8 (d, *J*_CP_ = 2.2 Hz), 53.8 (d, *J*_CP_ = 6.5 Hz), 53.7 (d, *J*_CP_ = 6.7 Hz), 27.1 (d, *J*_CP_ = 1.8 Hz), 26.5 (d, *J*_CP_ = 189.9 Hz), 16.0 (d, *J*_CP_ = 3.1 Hz). ^31^P NMR (243 MHz, CDCl_3_) δ 27.54. IR (neat): 2960, 1744, 1508, 1433 cm^–1^. HRMS (+p APCl) calcd. for C16H17ClNO3P [M + H]^+^ 338.0707, found 338.0708. SFC analysis: (AS-3, 3 μm particle size, 150 mm × 3 mm, 20.0% IPA/Heptane, with 0.2% Formic Acid, 2.5 mL/min, 230 nm) indicated 99% ee: *t*_R_ (major enantiomer) = 1.39 min, *t*_R_ (minor enantiomer) = 1.65 min. [α]^20^ D = −39.1° (c 3.31, CHCl_3_).

#### Dimethyl ((1*S*,2*R*)-1-(6-Methoxypyridin-3-yl)-2-phenylcyclopropyl)­phosphonate (**6m**)

The material was purified by flash chromatography (SiO_2_; hexanes/EtOAc, 9:1 to 1:1 gradient; *R*_*f*_ = 0.20 in 1:1 hexanes/EtOAc), which afforded major product 6m as a colorless oil (50 mg, 75% yield). ^1^H NMR (400 MHz, CDCl_3_) δ 7.89 (t, *J* = 2.5 Hz, 1H), 7.19–7.07 (m, 4H), 6.83–6.77 (m, 2H), 6.46 (d, *J* = 8.6 Hz, 1H), 3.85 (s, 3H), 3.76 (d, *J*_HP_ = 10.6 Hz, 3H), 3.72 (d, *J*_HP_ = 10.6 Hz, 3H), 3.03 (ddd, *J*_HP_ = 6.6, 9.0, 15.9 Hz, 1H), 2.07 (ddd, *J*_HP_ = 5.3, 9.0, 17.2 Hz, 1H), 1.72 (ddd, *J*_HP_ = 5.3, 6.6, 12.0 Hz, 1H). (Coupling constants are uncorrected). ^13^C­{^1^H} NMR (101 MHz, CDCl_3_) δ 163.5 (d, *J*_CP_ = 2.2 Hz), 150.3 (d, *J*_CP_ = 4.9 Hz), 142.6 (d, *J*_CP_ = 3.1 Hz), 135.6 (d, *J*_CP_ = 2.5 Hz), 128.5, 128.4, 127.0, 122.8, 110.4 (d, *J*_CP_ = 2.1 Hz), 53.7, 53.6 (d, *J*_CP_ = 6.6 Hz), 26.9 (d, *J*_CP_ = 1.5 Hz), 16.3 (d, *J*_CP_ = 3.0 Hz). ^31^P NMR (162 MHz, CDCl_3_) δ 28.85. IR (neat): 3461, 2952, 2849, 1605, 1567 cm^–1^. HRMS (+p APCl) calcd. for C_17_H_20_NO_4_P [M + H]^+^ 334.1203, found 334.1201. SFC analysis: (AS-3, 3 μm particle size, 150 mm × 3 mm, 20.0% IPA/Heptane, with 0.2% Formic Acid, 2.5 mL/min, 230 nm) indicated 99% ee: *t*_R_ (major enantiomer) = 1.47 min, *t*_R_ (minor enantiomer) = 2.49 min. [α]^20^ D = −42.2° (c 1.00, CHCl_3_).

#### Dimethyl ((1*S*,2*R*)-1-(2,6-Dichloropyridin-4-yl)-2-phenylcyclopropyl)­phosphonate (**6n**)

The material was purified by flash chromatography (SiO_2_; hexanes/EtOAc, 9:1 to 1:1 gradient; *R*_*f*_ = 0.20 in 1:1 hexanes/EtOAc), which afforded the major product **6n** as a colorless oil (45 mg, 60% yield). ^1^H NMR (600 MHz, CDCl_3_) δ 7.20–7.12 (m, 3H), 6.92 (s, 2H), 6.82 (d, *J* = 7.7 Hz, 2H), 3.82 (d, *J*_HP_ = 10.8 Hz, 3H), 3.76 (d, *J*_HP_ = 10.8 Hz, 3H), 3.11 (ddd, *J*_HP_ = 6.8, 8.9, 16.2 Hz, 1H), 2.09 (ddd, *J*_HP_ = 5.7, 9.0, 16.7 Hz, 1H), 1.77 (ddd, *J*_HP_ = 5.7, 6.2, 12.3 Hz, 1H). (Coupling constants are uncorrected). ^13^C­{^1^H} NMR (151 MHz, CDCl_3_) δ 150.3 (d, *J*_CP_ = 2.2 Hz), 150.0 (d, *J*_CP_ = 3.1 Hz), 134.2 (d, *J*_CP_ = 3.3 Hz), 128.8, 128.1, 127.8, 126.3 (d, *J*_CP_ = 3.8 Hz), 53.9 (dd, *J*_CP_ = 2.4, 6.7 Hz), 28.6 (d, *J*_CP_ = 187.9 Hz), 28.0 (d, *J*_CP_ = 2.4 Hz), 15.7 (d, *J*_CP_ = 3.2 Hz). ^31^P NMR (243 MHz, CDCl_3_) δ 26.13. IR (neat): 2953, 1578, 1532, 1500 cm^–1^. HRMS (+p APCl) calcd. for C_16_H_16_Cl_2_NO_3_P [M + H]^+^ 372.0329, found 372.0320. SFC analysis: (AS-3, 3 μm particle size, 150 mm × 3 mm, 20.0% IPA/Heptane, with 0.2% Formic Acid, 2.5 mL/min, 230 nm) indicated 41% ee: *t*_R_ (major enantiomer) = 2.81 min, *t*_R_ (minor enantiomer) = 4.59 min. [α]^20^ D = −41.6° (c 2.50, CHCl_3_).

#### Dimethyl ((1*S*,2*R*)-2-Phenyl-1-(9-phenyl-9*H*-carbazol-3-yl)­cyclopropyl)­phosphonate (**6o**)

The material was purified by flash chromatography (SiO_2_; hexanes/EtOAc, 9:1 to 1:1 gradient; *R*_*f*_ = 0.20 in 1:1 hexanes/EtOAc), which afforded the major product **6o** as a yellow oil (63 mg, 81% yield). ^1^H NMR (600 MHz, CDCl_3_) δ 8.04 (dt, *J* = 1.0, 7.8 Hz, 1H), 7.91 (t, *J* = 1.8 Hz, 1H), 7.58–7.54 (m, 2H), 7.52–7.49 (m, 2H), 7.44–7.41 (m, 1H), 7.39–7.37 (m, 2H), 7.30–7.22 (m, 1H), 7.13 (dt, *J* = 0.7, 8.5 Hz, 1H), 7.06–6.98 (m, 4H), 6.83–6.78 (m, 2H), 3.75 (d, *J*_HP_ = 10.5 Hz, 3H), 3.71 (d, *J*_HP_ = 10.5 Hz, 3H), 3.09 (ddd, *J*_HP_ = 6.6, 9.0, 16.0 Hz, 1H), 2.18 (ddd, *J*_HP_ = 5.1, 8.9, 17.5 Hz, 1H), 1.87 (ddd, *J*_HP_ = 5.1, 6.6, 12.5 Hz, 1H). (Coupling constants are uncorrected). ^13^C­{^1^H} NMR (151 MHz, CDCl_3_) δ 141.3, 140.3 (d, *J*_CP_ = 2.1 Hz), 137.9, 136.5 (d, *J*_CP_ = 2.4 Hz), 130.6 (d, *J*_CP_ = 3.3 Hz), 130.1, 128.4, 128.1, 127.7, 127.3, 126.6, 126.3, 125.2, 124.3 (d, *J*_CP_ = 4.8 Hz), 123.5, 123.4 (d, *J*_CP_ = 1.5 Hz), 120.6, 120.3, 110.2, 109.5 (d, *J*_CP_ = 2.2 Hz), 53.7 (dd, *J*_CP_ = 6.7, 13.3 Hz), 29.9 (d, *J*_CP_ = 188.3 Hz), 27.6, 17.7 (d, *J*_CP_ = 3.2 Hz). ^31^P NMR (243 MHz, CDCl_3_) δ 29.67. IR (neat): 3470, 2957, 2853, 2104, 1589, 1536 cm^–1^. HRMS (+p APCl) calcd. for C_29_H_26_NO_3_P [M + H]^+^ 468.1723, found 468.1718. HPLC analysis: (Chiralcel AD-H, 25 cm × 4.6 mm, 3.0% i-PrOH/hexanes, 1.0 mL/min, 254 nm) indicated 96% ee: *t*_R_ (minor enantiomer) = 19.84 min, *t*_R_ (major enantiomer) = 23.78 min. [α]^20^ D = −46.6° (c 2.00, CHCl_3_).

### General Procedure for Cyclopropenation with Diazophosphonates

A clean, oven-dried, and flame-dried 25.0 mL round-bottom flask (flask-A) equipped with activated 4 Å molecular sieves and a magnetic stir bar was evacuated and purged with argon (2–3 times). After cooling down to room temperature, ethynylbenzene 7a (5 equiv, 1.00 mmol) followed by Rh_2_(Cat.) (0.01 equiv) was then added. The flask was once again evacuated and purged with argon (3–5 times) and anhydrous CH_2_Cl_2_ (3.0 mL) was added. The flask and its contents were then set to stir at room temperature using a hot plate or using a heating mantle under an argon atmosphere. To a second oven-dried round-bottom flask (flask-B), which was evacuated and purged with argon, diazophosphonates (0.20 mmol) were added. Flask-B and its contents were evacuated and purged with argon (2–3 times) and anhydrous CH_2_Cl_2_ (10.0 mL) was then added to obtain a solution of the diazo compound. The solution was transferred into a 12.0 mL plastic syringe. Using a well-calibrated syringe pump, a slow addition of the diazo solution into the stirring solution of flask-A under an inert atmosphere was initiated. After the complete addition of the solution (5 h), the residual diazo compound in the 12.0 mL plastic syringe was rinsed with anhydrous CH_2_Cl_2_ (1.0 mL) and transferred dropwise into the stirring reaction mixture of flask-A. The resulting solution was stirred for 5 h before concentrating the solution under reduced pressure. Purification by flash column chromatography on silica gel (hexanes:EtOAc) was used to afford the final products.

#### Dimethyl (*R*)-(1,2-Diphenylcycloprop-2-en-1-yl)­phosphonate (**8a**)

The material was purified by flash chromatography (SiO_2_; hexanes/EtOAc, 9:1 to 1:1 gradient; *R*_*f*_ = 0.33 in 1:1 hexanes/EtOAc), which afforded the major product **8a** as a yellow oil (57 mg, 95% yield). Spectroscopic data are in agreement with those reported in the literature. ^1^H NMR (600 MHz, CDCl_3_) δ 7.70 (dd, *J* = 1.6, 8.0 Hz, 2H), 7.51 (dd, *J* = 1.0, 7.3 Hz, 2H), 7.48–7.36 (m, 3H), 7.31–7.25 (m, 3H), 7.21 (t, *J* = 7.4 Hz, 1H), 3.72 (d, *J* = 10.6 Hz, 3H), 3.68 (d, *J* = 10.5 Hz, 3H). (Coupling constants are uncorrected). Spectroscopic data are in agreement with those reported in the literature.^72^ HPLC analysis: (Chiralcel ODH, 25 cm × 4.6 mm, 5.0% i-PrOH/hexanes, 1.0 mL/min, 230 nm) indicated 95% ee: *t*_R_ (major enantiomer) = 13.02 min, *t*_R_ (minor enantiomer) = 16.29 min. (The enantiomeric excess was determined by HPLC using the published procedure). [α]^20^ D: −38.5° (c 0.29, CHCl_3_).

## Supplementary Material



## Data Availability

The data underlying this study are available in the published article and its Supporting Information.

## Notes

Experimental procedures, full characterization data, and copies of NMR spectra (^1^H, ^13^C­{^1^H}, ^19^F, and ^31^P­{^1^H}) for all new compounds, as well as single-crystal X-ray diffraction data (PDF)
