# Supplementary material for: Synthesis of Aryl and Heteroaryl Diazophosphonates via Palladium-Catalyzed Cross-Coupling and Their Application in Rhodium-Catalyzed Cyclopropanation
Source: J Org Chem. 2026 Jul 10;91(29):10171–82. doi: 10.1021/acs.joc.6c00961 (PMC13411051; doi:10.1021/acs.joc.6c00961)
Supplement: Supplementary file 1 [file jo6c00961_si_001.pdf]

Supporting Information

Synthesis of Aryl and Heteroaryl Diazophosphonates via Palladium-Catalyzed Cross-Coupling and their Application in Rhodium-Catalyzed Cyclopropanation

Yasir Naeem, Duc Ly, Huw M. L. Davies*

Department of Chemistry, Emory University, 1515 Dickey Drive, Atlanta, Georgia 30322, United States.

Corresponding author email: hmdavie@emory.edu

Table of Contents

1. **Catalyst and ligand structures…………………………………………...…………S2**

2. **Dirhodium tetracarboxylate catalysts…………………………...………………S3**

3. **Purchased and synthesized substrates along with their corresponding CAS numbers……………………………………………………………...………....….S4**

4. **Diazo compounds…………………………………………………...…......….S5**

5. **Cyclopropanation and cyclopropenation……………………..……….......…...S6**

6. **Palladium-coupling ^{1}H NMR, ^{13}C{^{1}H} NMR, ^{19}F NMR, and ^{31}P NMR Spectroscopic Data…………………………………………...………………...……….S7**

7. **Cyclopropanation ^{1}H NMR, ^{13}C{^{1}H} NMR, ^{19}F NMR, and ^{31}P NMR Spectroscopic Data……………………………………………………………...…...……….S59**

8. **Enantioselectivity Determination by HPLC or SFC…………………………..….S100**

9. **X-Ray Crystallographic Data for Compound 6b, 6f, 6k, and 6m…………..……..S109**

10. **Citations for X-ray Crystallography…………………………………………….S138**

1. Catalyst and ligand structures.

Palladium Catalyst.

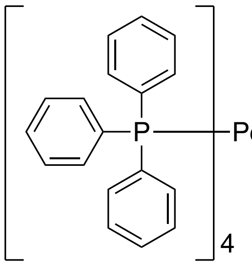

$Pd(PPh_3)_4$
CAS No. 14221-01-3

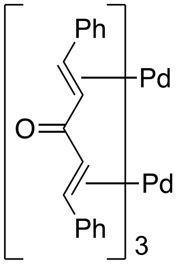

$Pd_2(dba)_3$
CAS No. 51364-51-3

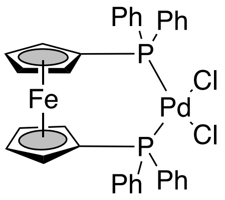

$Pd(dppf)Cl_2$
CAS No. 72287-26-4

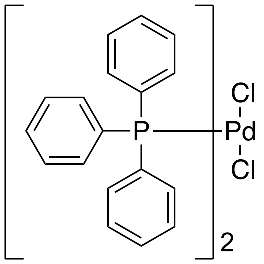

$PdCl_2(PPh_3)_2$
CAS No. 13965-03-2

Ligand for palladium Catalyst.

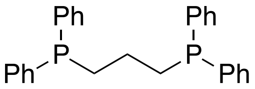

dppp
CAS No. 6737-42-4

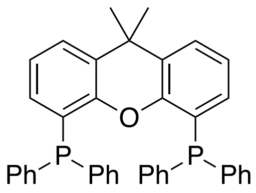

Xantphos
CAS No. 161265-03-8

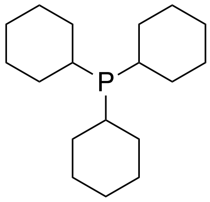

Pcy_3
CAS No. 2622-14-2

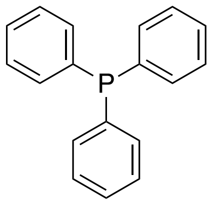

PPh_3
CAS No. 603-35-0

2. Dirhodium tetracarboxylate catalysts.

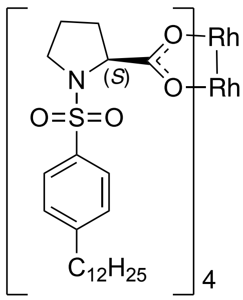

Generation: Prolinato
Rh$_2$(*S*-DOSP)$_4$

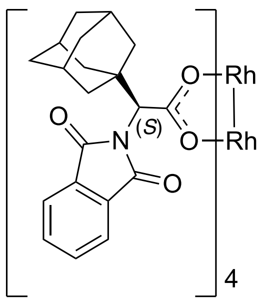

Generation: Phthalimido
Rh$_2$(*S*-PTAD)$_4$

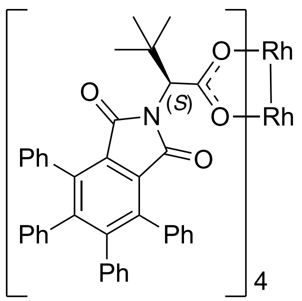

Generation: Phthalimido
Rh$_2$(*S*-TPPTTL)$_4$

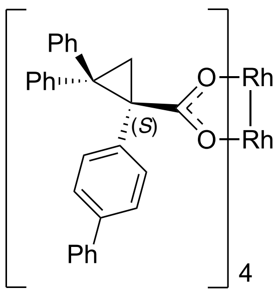

Generation: TPCP
Rh$_2$(*S*-*p*-Ph-TPCP)$_4$

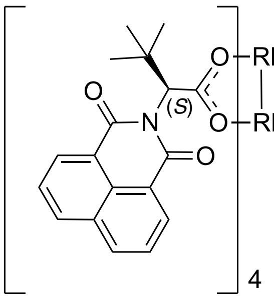

Generation: Napthalimido
Rh$_2$(*S*-NTTL)$_4$

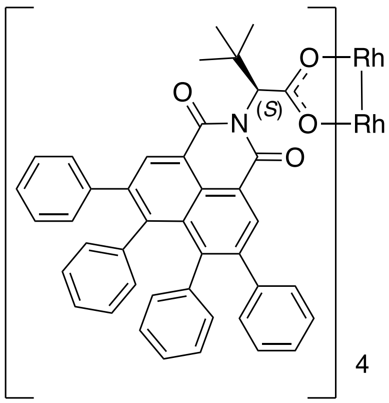

Generation: Napthalimido
Rh$_2$(*S*-TPNTTL)$_4$

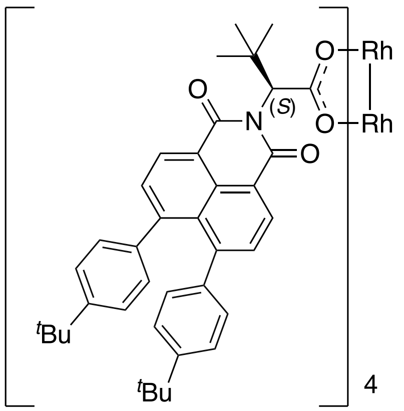

Generation: Napthalimido
Rh$_2$(*S*-*di*(4-^{t}But)PhNTTL)$_4$

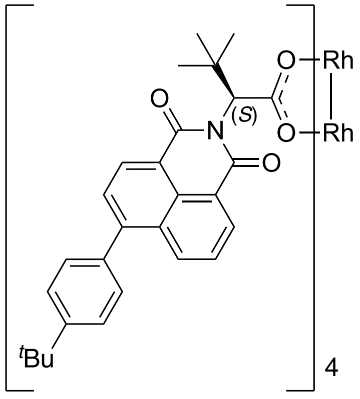

Generation: Napthalimido
Rh$_2$(*S*-Mono(4-^{t}But)PhNTTL)$_4$

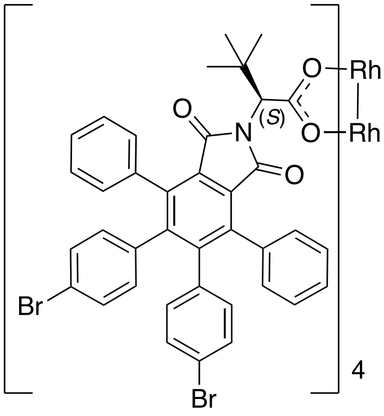

Generation: Phthalimido
Rh$_2$(*S*-*di*-(4-Br)TPPTTL)$_4$

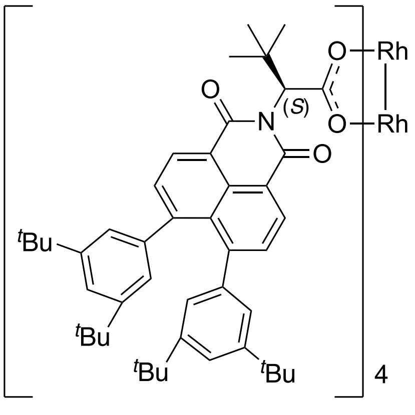

Generation: Napthalimido
Rh$_2$(*S*-*di*(3,5-^{t}But)PhNTTL)$_4$

3. Purchased and synthesized substrates along with their corresponding CAS numbers.

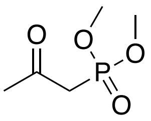<br>**SM for S1a**<br>CAS No. 4202-14-6	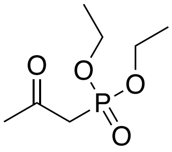<br>**SM for S1d**<br>CAS No. 1067-71-6	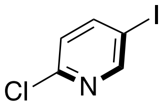<br>**2a**<br>CAS No. 69045-79-0	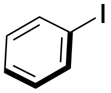<br>**2b**<br>CAS No. 591-50-4
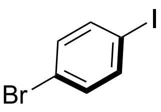<br>**2c**<br>CAS No. 589-87-7	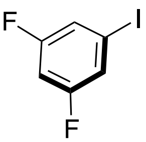<br>**2e**<br>CAS No. 2265-91-0	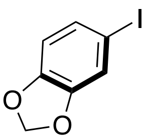<br>**2f**<br>CAS No. 5876-51-7	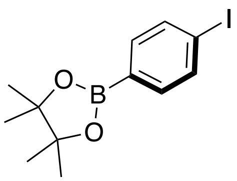<br>**2g**<br>CAS No. 73852-88-7
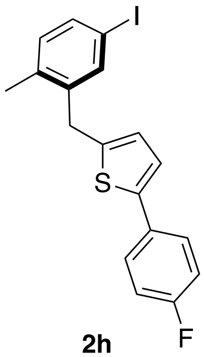<br>**2h**<br>CAS No. 898566-17-1	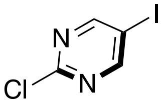<br>**2i**<br>CAS No. 32779-38-7	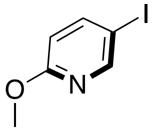<br>**2j**<br>CAS No. 13472-61-2	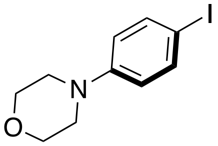<br>**2k**<br>CAS No. 87350-77-4
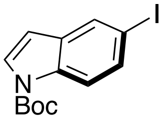<br>**2l**<br>Added protecting group	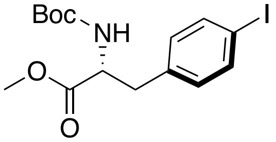<br>**2m**<br>Added protecting group	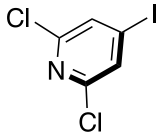<br>**2n**<br>CAS No. 98027-84-0	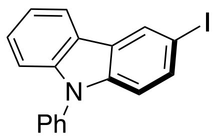<br>**2o**<br>CAS No. 502161-03-7
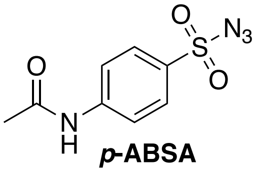<br>***p*-ABSA**<br>CAS No. 2158-14-7	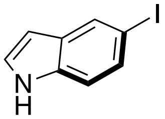<br>**S2l**<br>CAS No. 16066-91-4	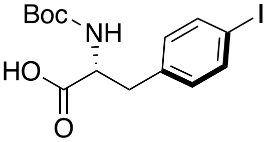<br>**S2m**<br>CAS No. 176199-35-2	**SM = Starting Material**

4. Diazo compounds.

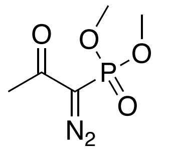

S1a

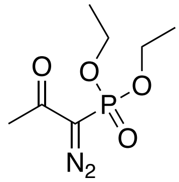

S1d

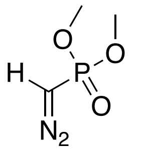

1a

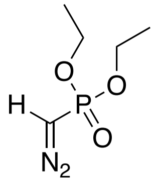

1d

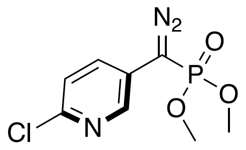

3a

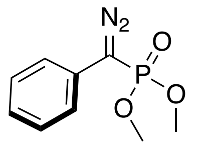

3b

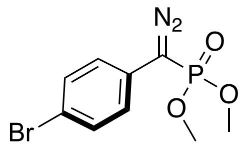

3c

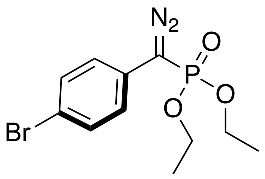

3d

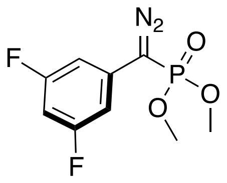

3e

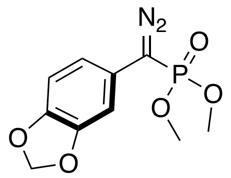

3f

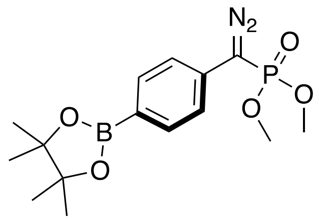

3g

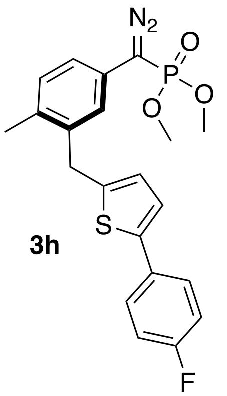

3h

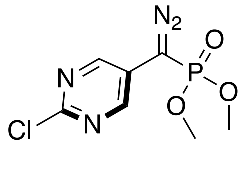

3i

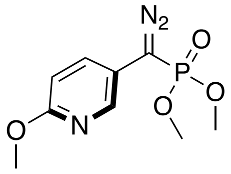

3j

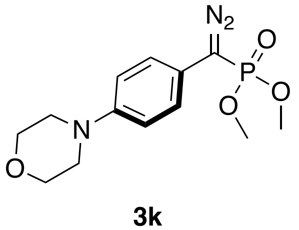

3k

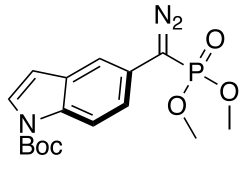

3l

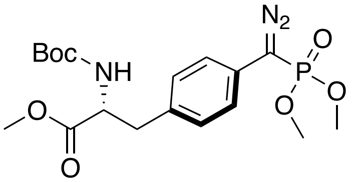

3m

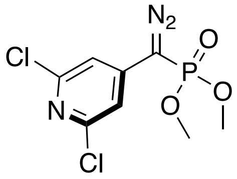

3n

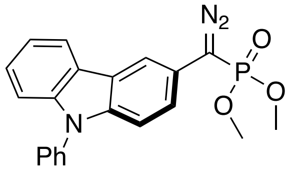

3o

5. Cyclopropanation and cyclopropane.

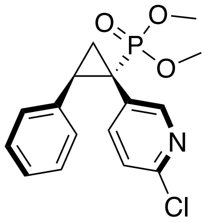

6a

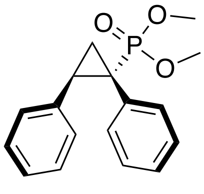

6b

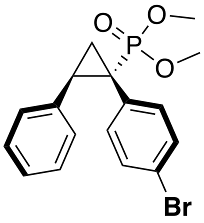

6c

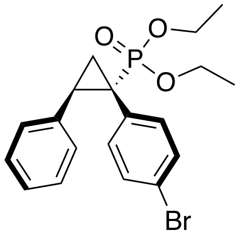

6d

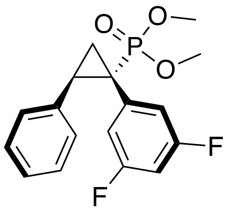

6e

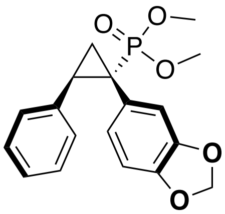

6f

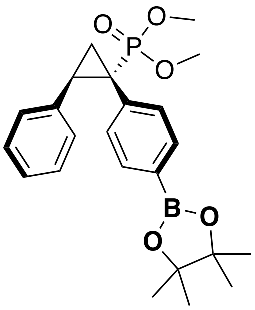

6g

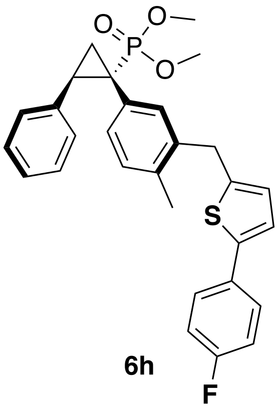

6h

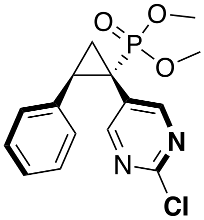

6i

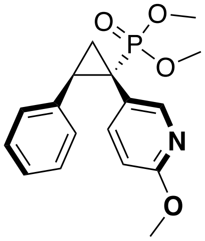

6j

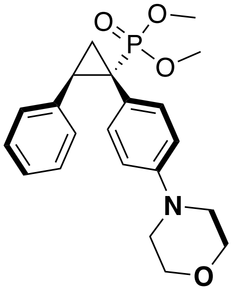

6k

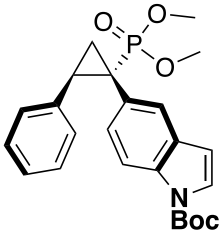

6l

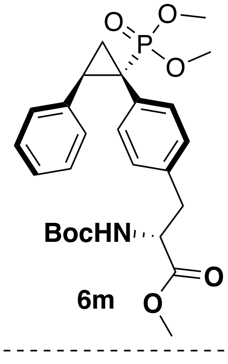

6m

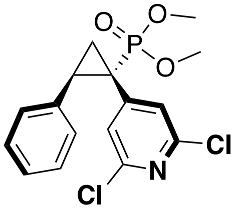

6n

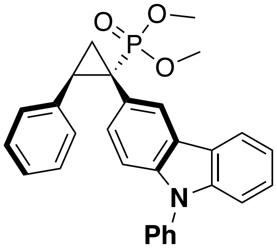

6o

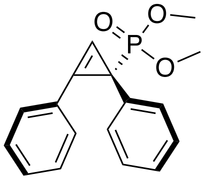

8a

6. Palladium-coupling ^{1}H NMR, ^{13}C{H} NMR, ^{19}F NMR, and ^{31}P NMR Spectroscopic Data.

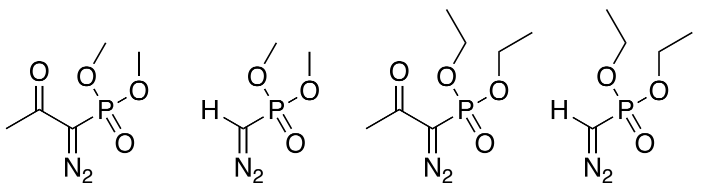

S1a **1a** **S1d** **1d**

The NMR spectra for these compounds have been reported in previous publications.[1-3]

(1) Brown, D. G.; Velthuisen, E. J.; Commerford, J. R.; Brisbois, R. G. A Convenient Synthesis of Dimethyl (Diazomethyl)phosphonate (Seyferth/Gilbert Reagent). *J. Org. Chem.* **1996**, 61, 2540-2541.

(2) Du, T.; Du, F.; Ning, Y.; Peng, Y. Organocatalytic Enantioselective 1,3-Dipolar Cycloadditions between Seyferth–Gilbert Reagent and Isatylidene Malononitriles: Synthesis of Chiral Spiro-phosphonylpyrazoline-oxindoles. *Org. Lett.* **2015**, 17, 1308-1311.

(3) Du, F.; Zhou, J.; Peng, Y. Asymmetric Reaction of α-Diazomethylphosphonates with *α*-Ketoesters To Access Optically Active *α*-Diazo-*β*-hydroxyphosphonate Derivatives. *Org. Lett.* **2017**, 19, 1310-1313.

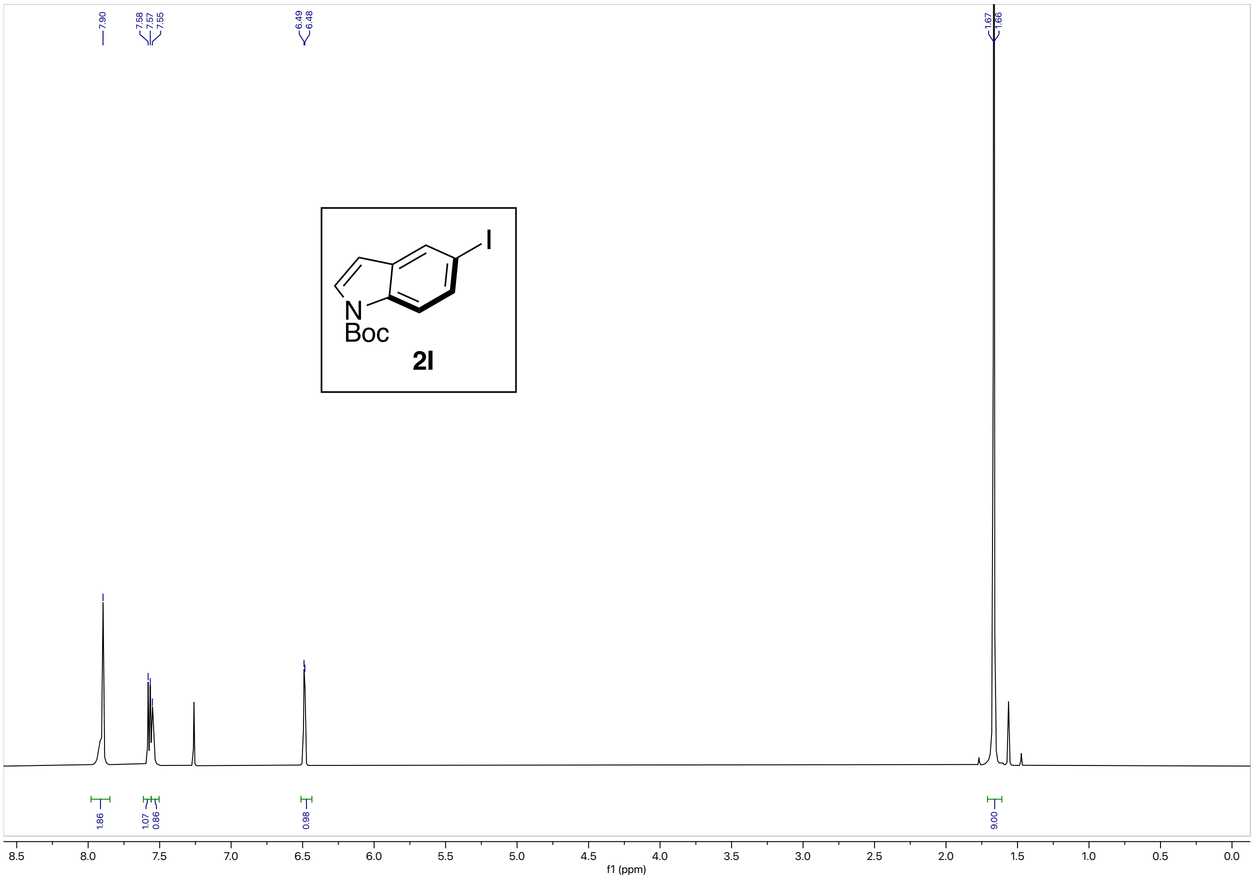

^{1}H NMR spectrum (600 MHz, Chloroform-*d*) (s, 7.26 ppm) of **2l**.

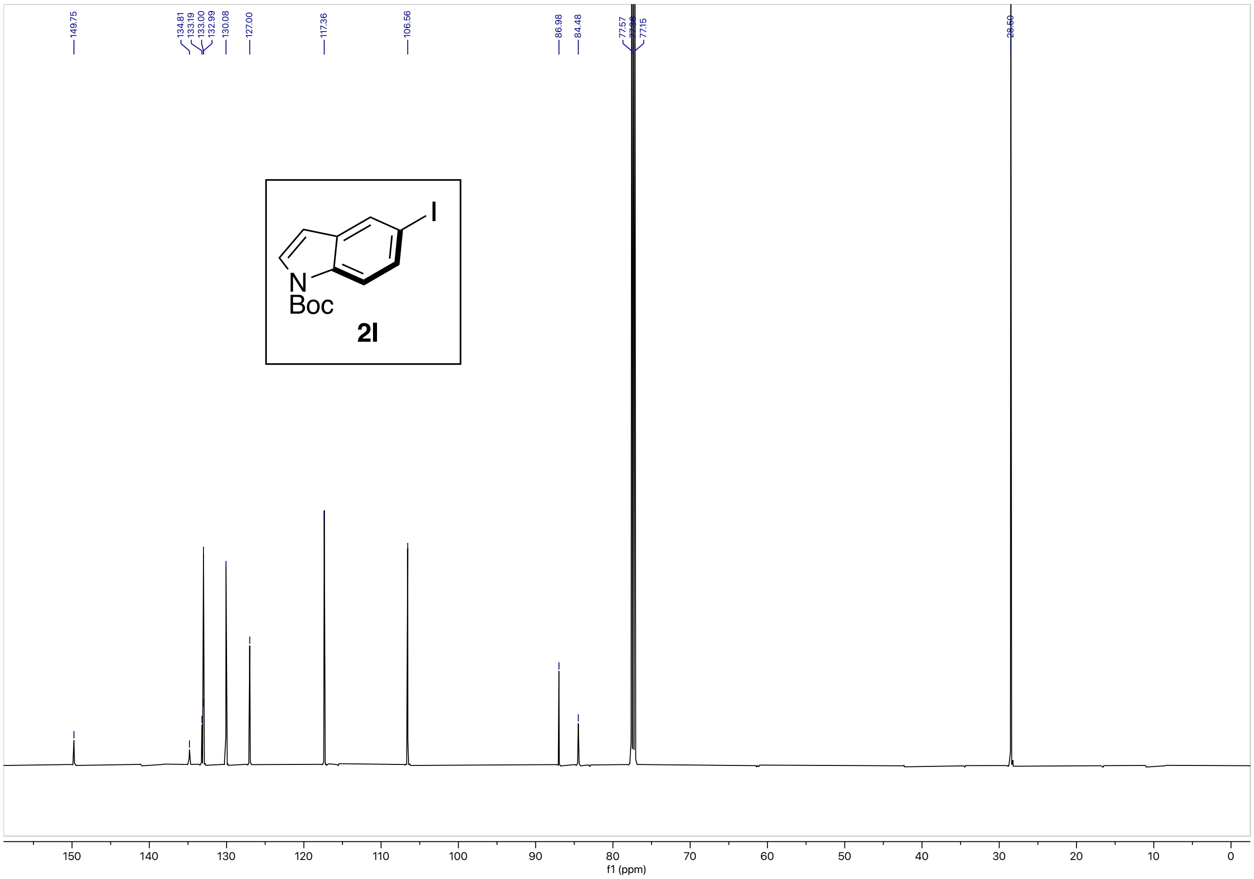

$^{13}C\{^1H\}$ NMR spectrum (151 MHz, Chloroform-*d*) (t, 77.36 ppm) of **2l.**

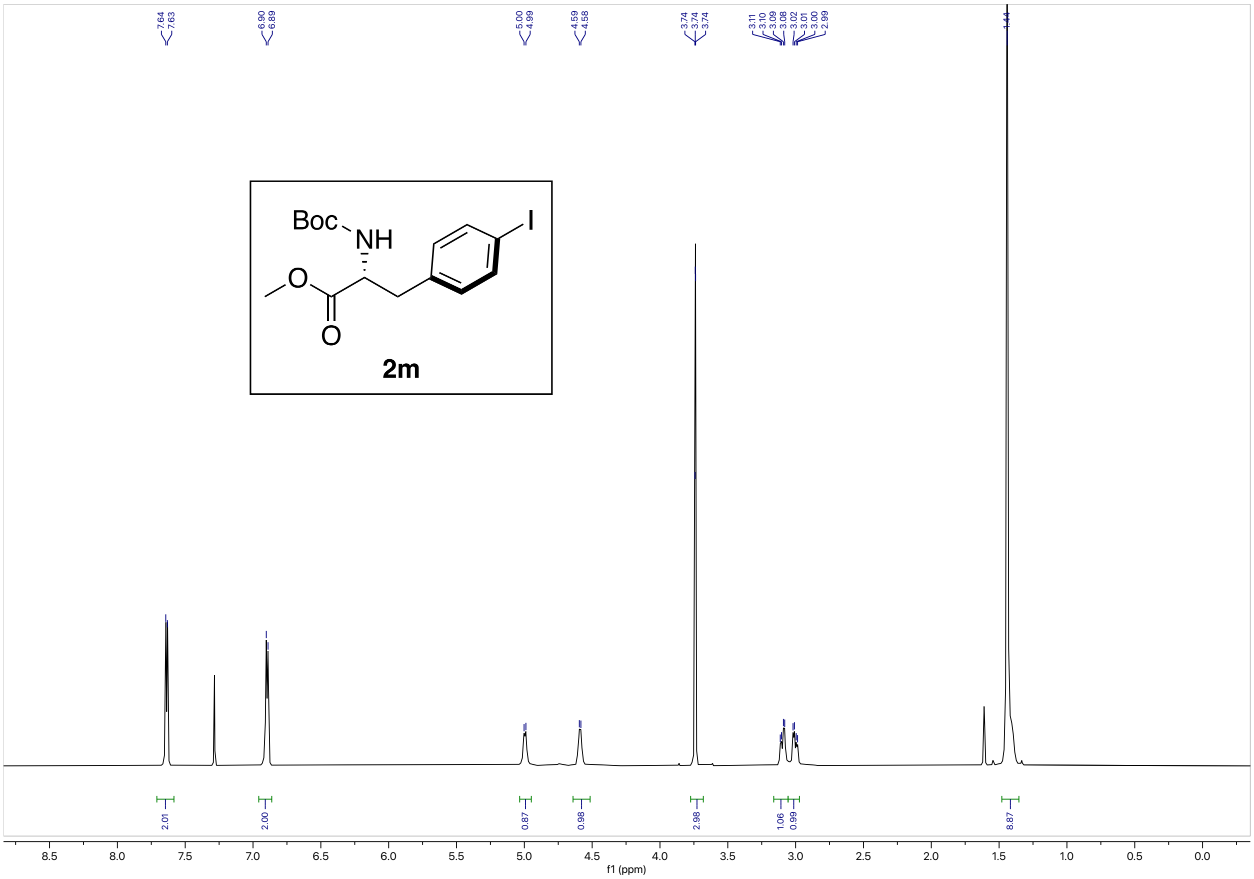

^{1}H NMR spectrum (600 MHz, Chloroform-*d*) (s, 7.26 ppm) of **2m**.

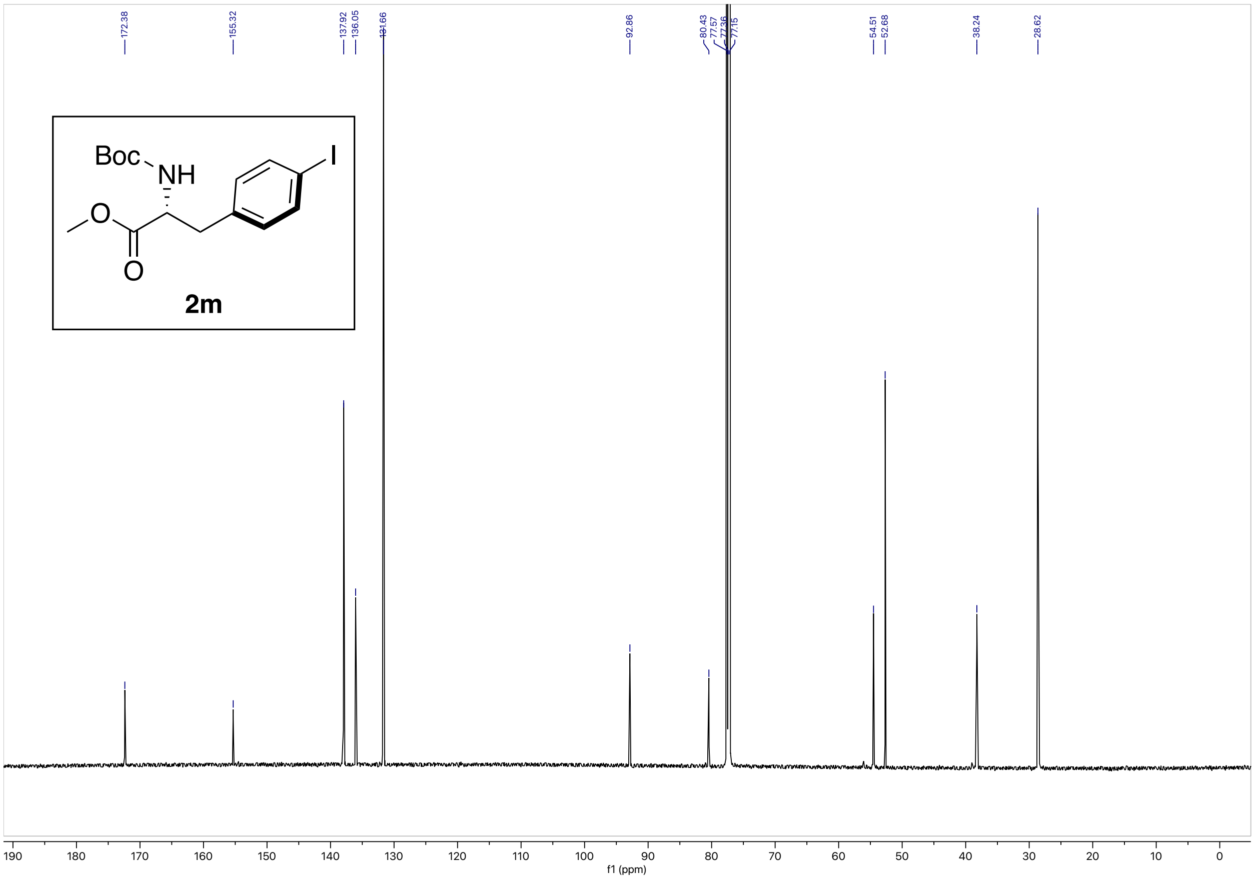

$^{13}C\{^{1}H\}$ NMR spectrum (151 MHz, Chloroform-*d*) (t, 77.36 ppm) of **2m**.

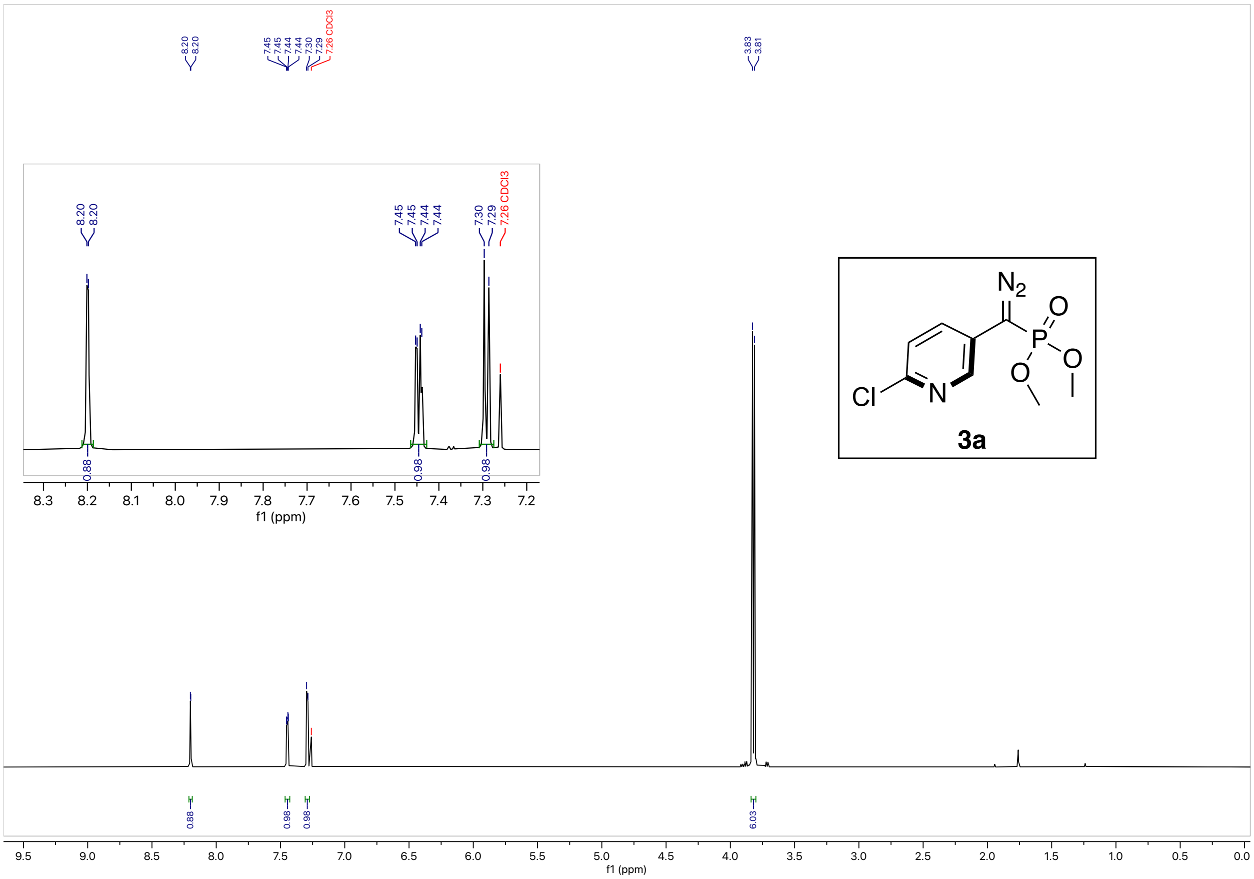

^{1}H NMR spectrum (800 MHz, Chloroform-*d*) (s, 7.26 ppm) of **3a.**

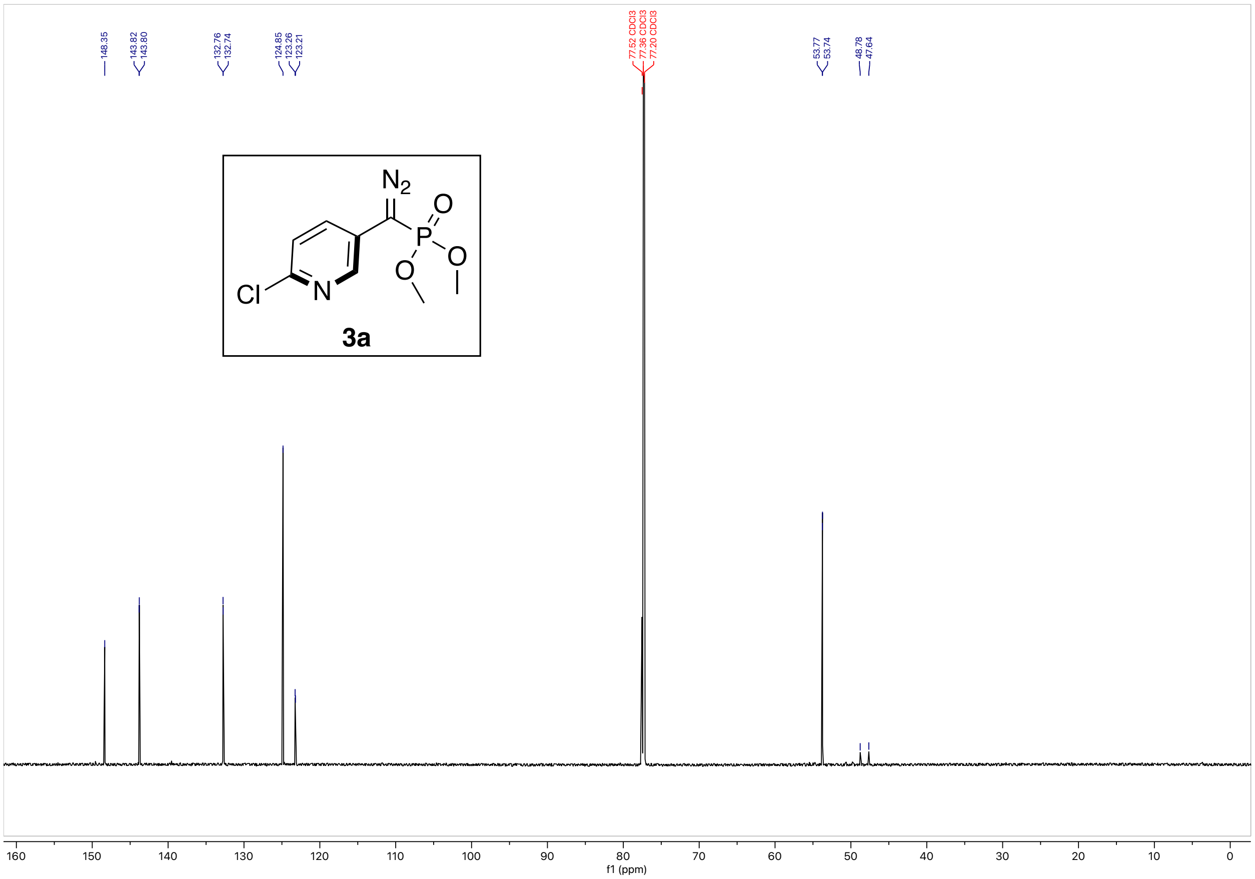

$^{13}C\{^{1}H\}$ NMR spectrum (201 MHz, Chloroform-*d*) (t, 77.36 ppm) of **3a.**

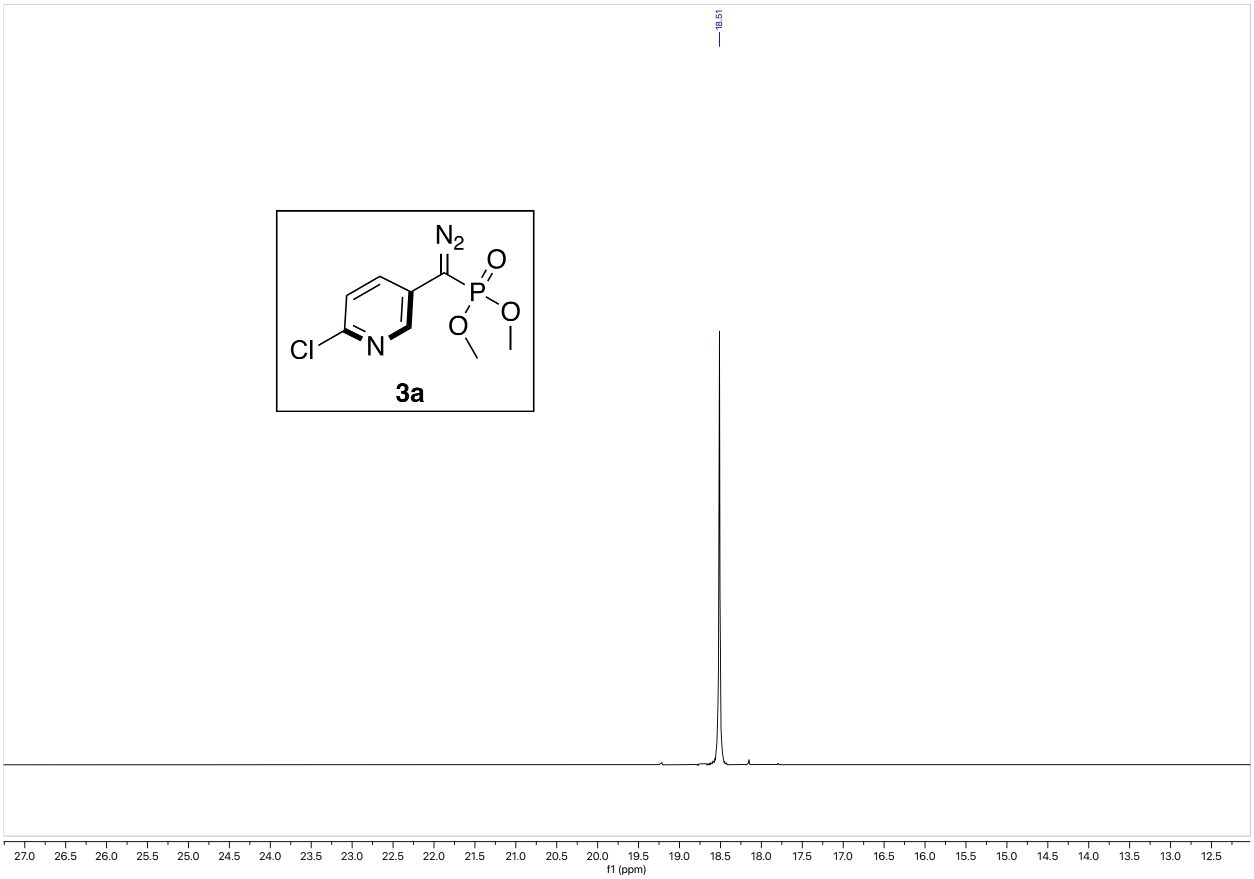

^{31}P NMR spectrum (162 MHz, Chloroform-d) of **3a.**

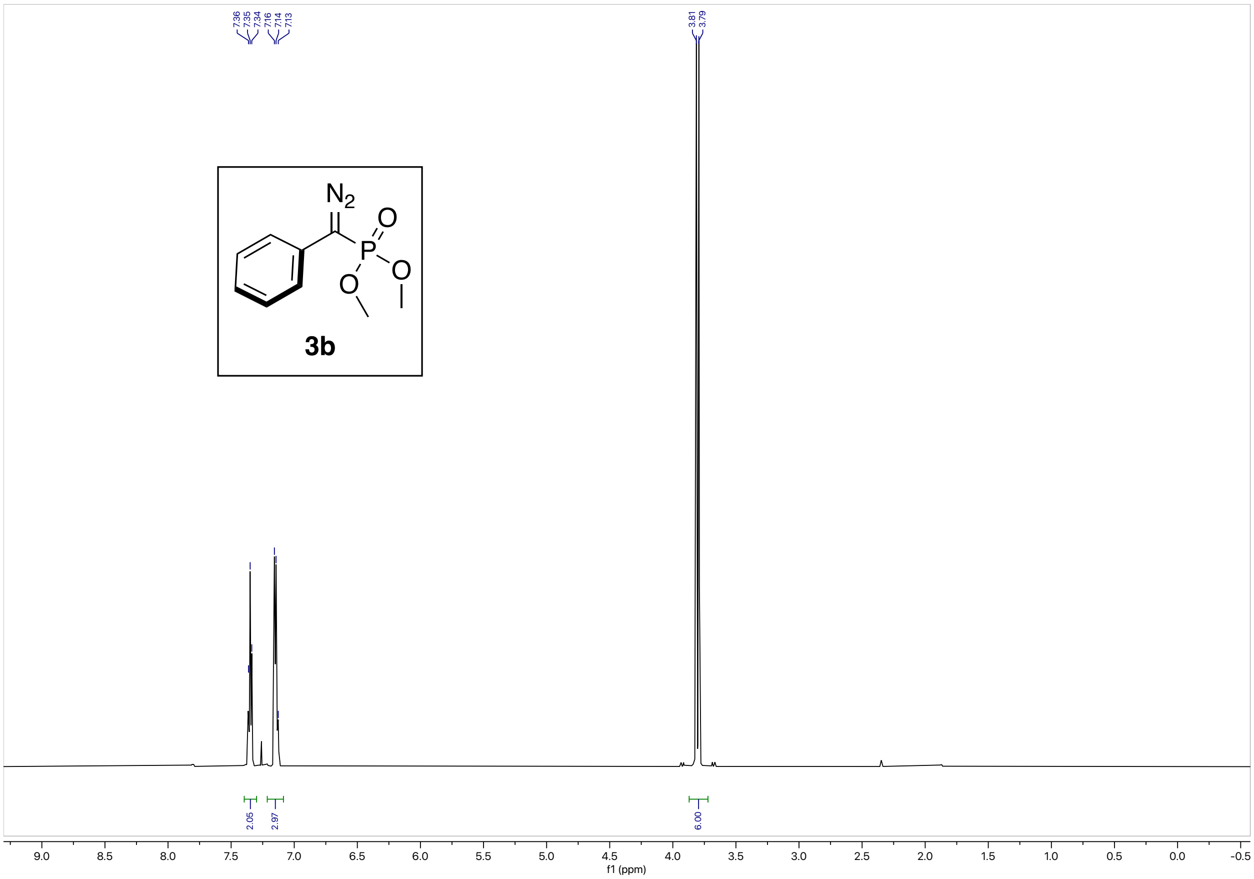

^{1}H NMR spectrum (600 MHz, Chloroform-*d*) (s, 7.26 ppm) of **3b**.

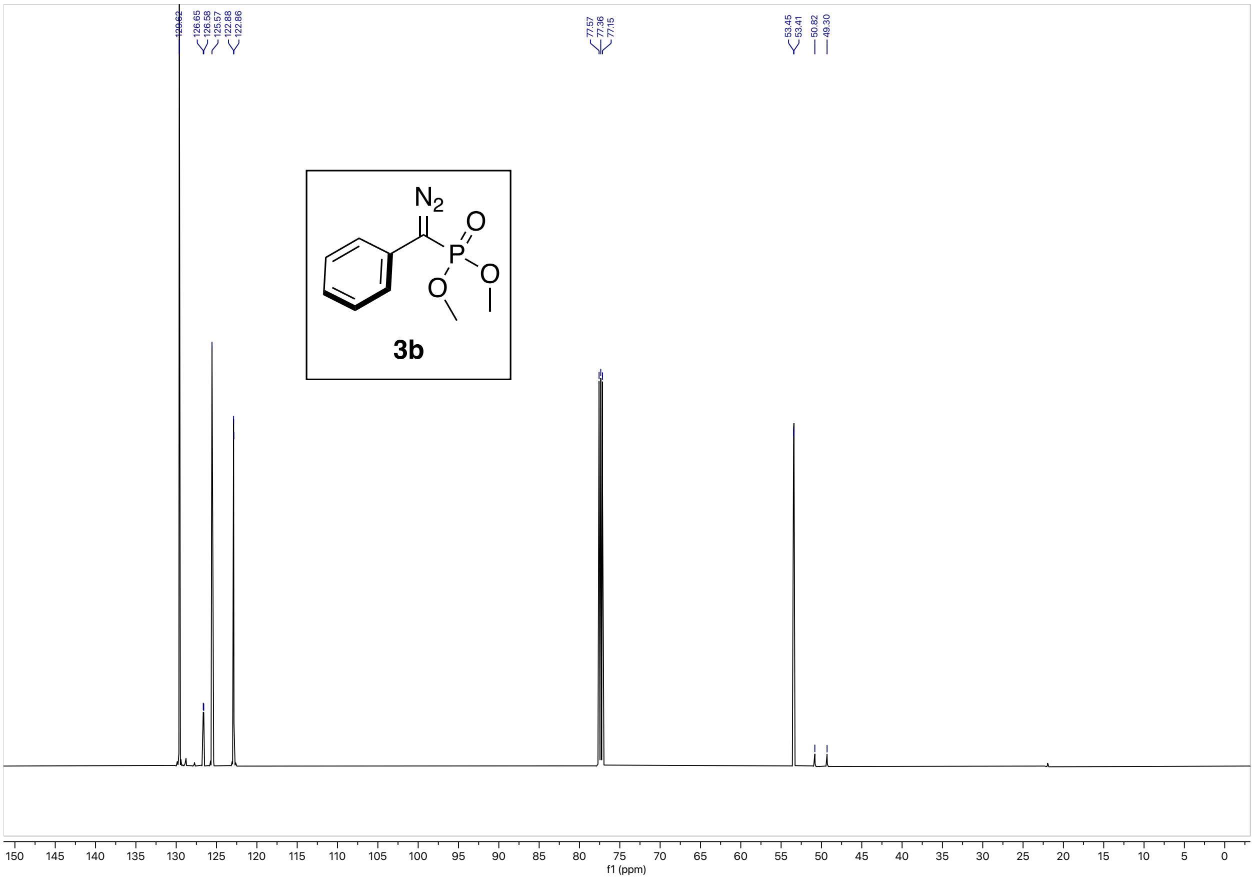

$^{13}C\{^{1}H\}$ NMR spectrum (151 MHz, Chloroform-*d*) (t, 77.36 ppm) of **3b.**

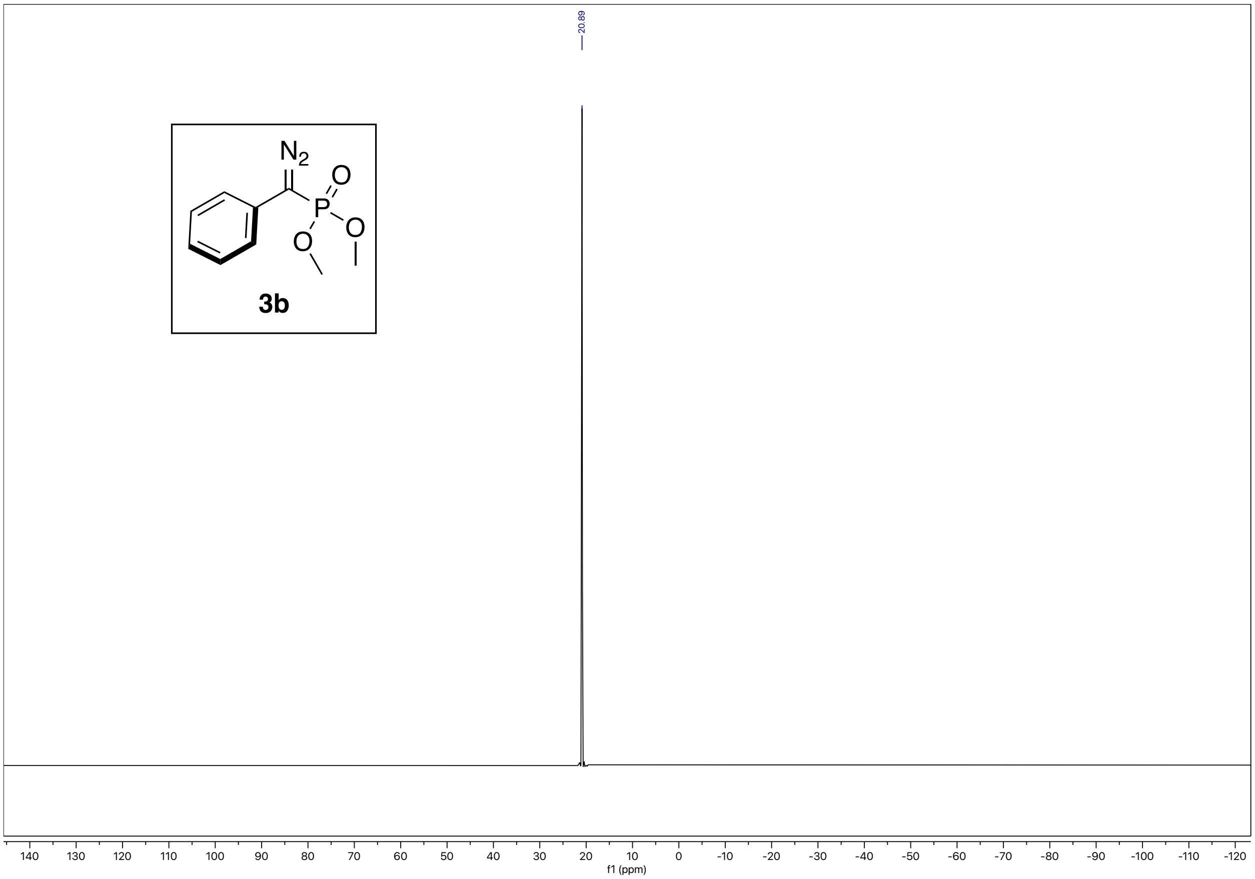

^{31}P NMR spectrum (243 MHz, Chloroform-d) of **3b.**

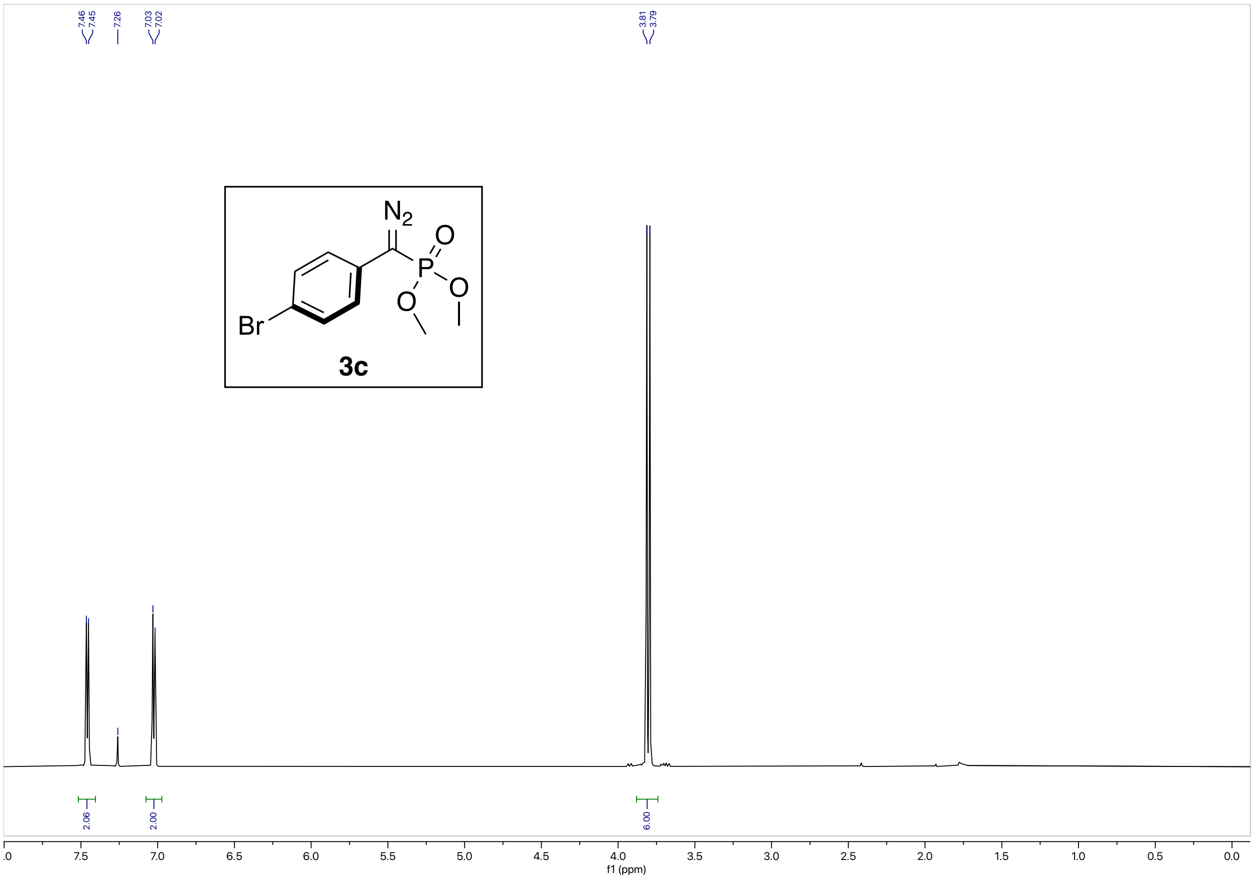

^{1}H NMR spectrum (600 MHz, Chloroform-*d*) (s, 7.26 ppm) of **3c**.

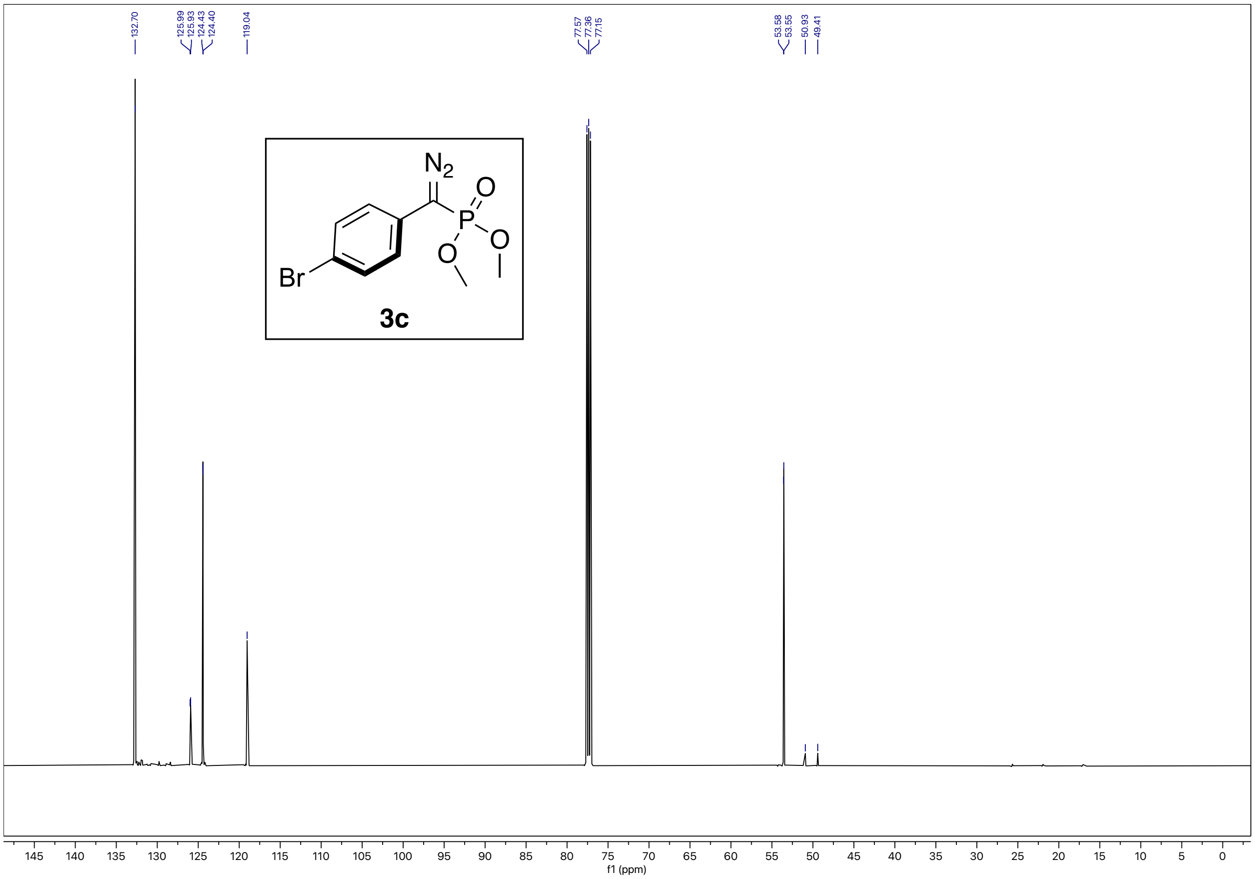

$^{13}C\{^{1}H\}$ NMR spectrum (151 MHz, Chloroform-*d*) (t, 77.36 ppm) of **3c.**

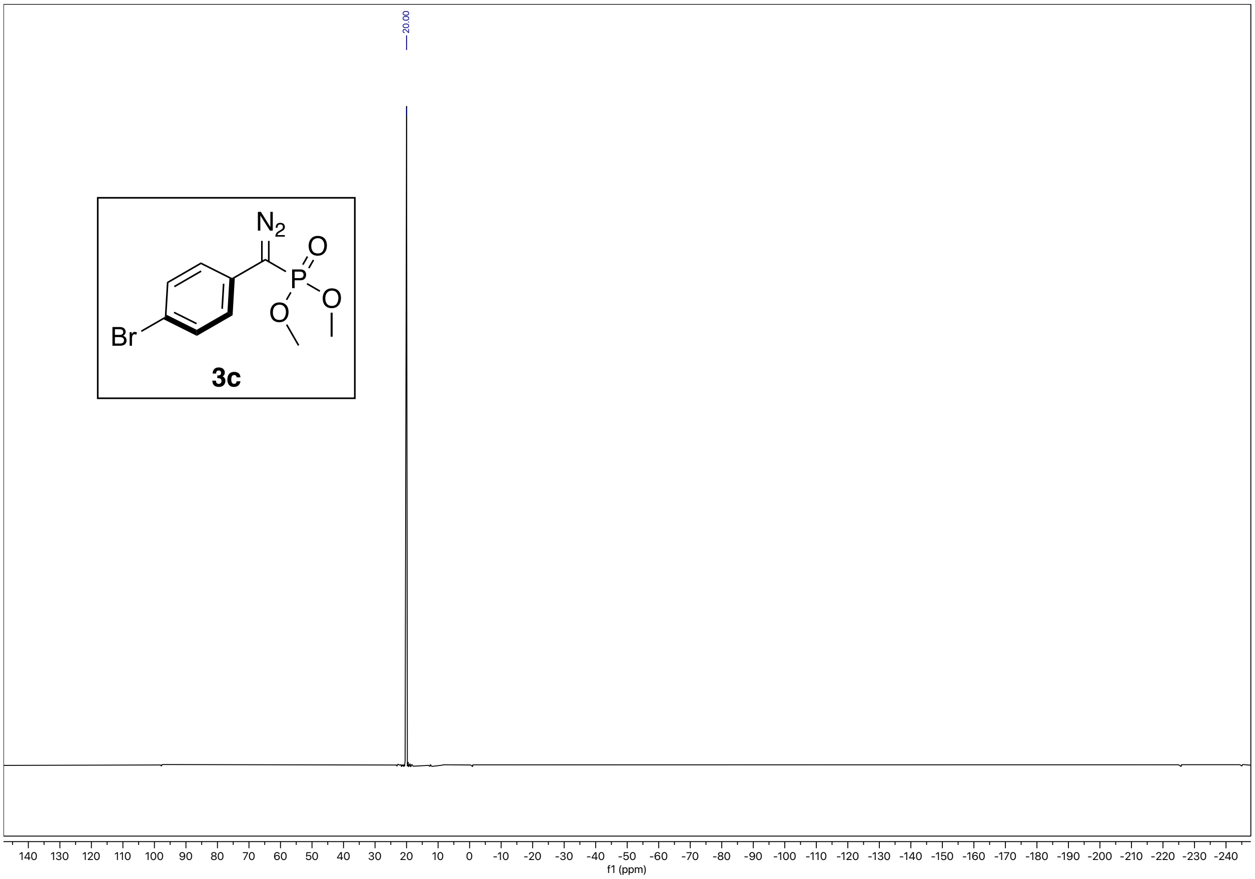

^{31}P NMR spectrum (243 MHz, Chloroform-d) of **3c.**

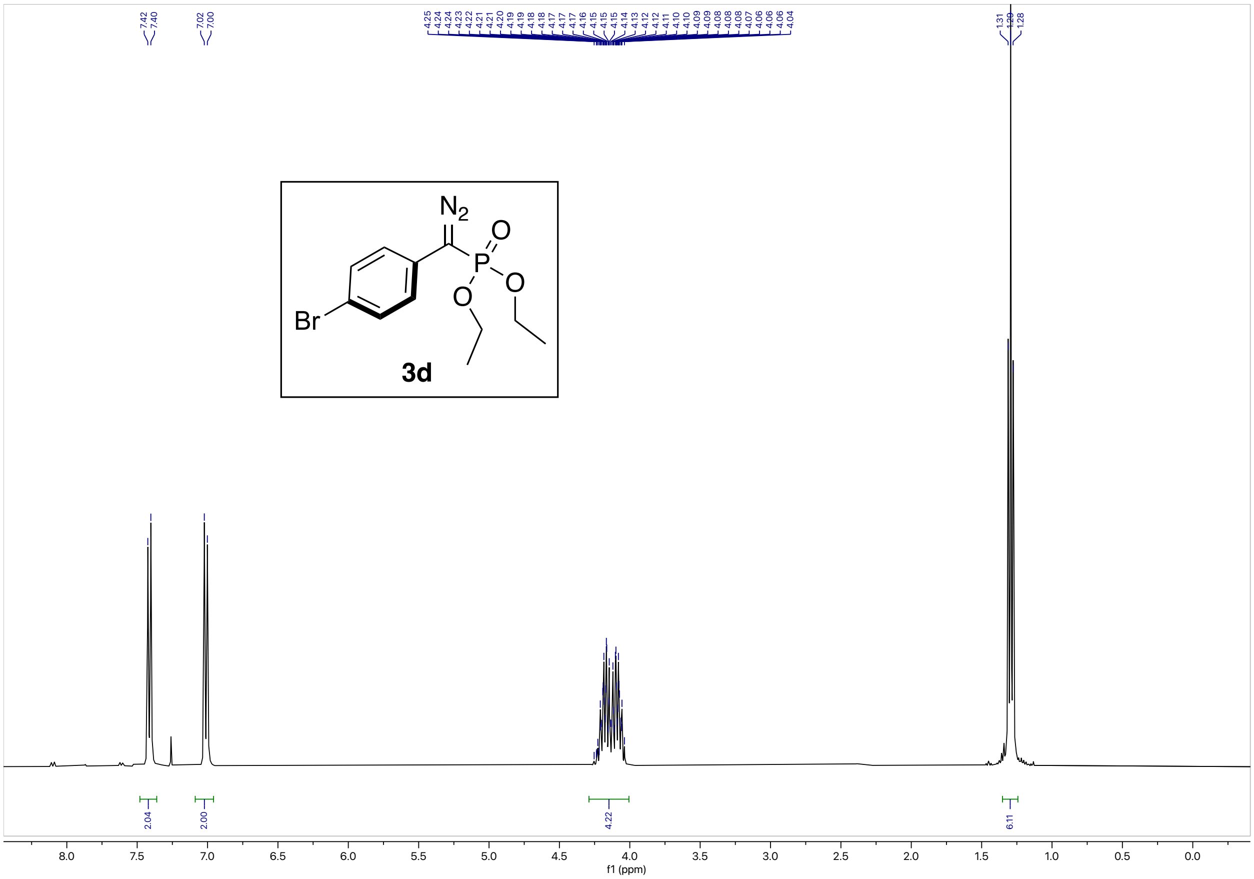

^{1}H NMR spectrum (400 MHz, Chloroform-*d*) (s, 7.26 ppm) of **3d.**

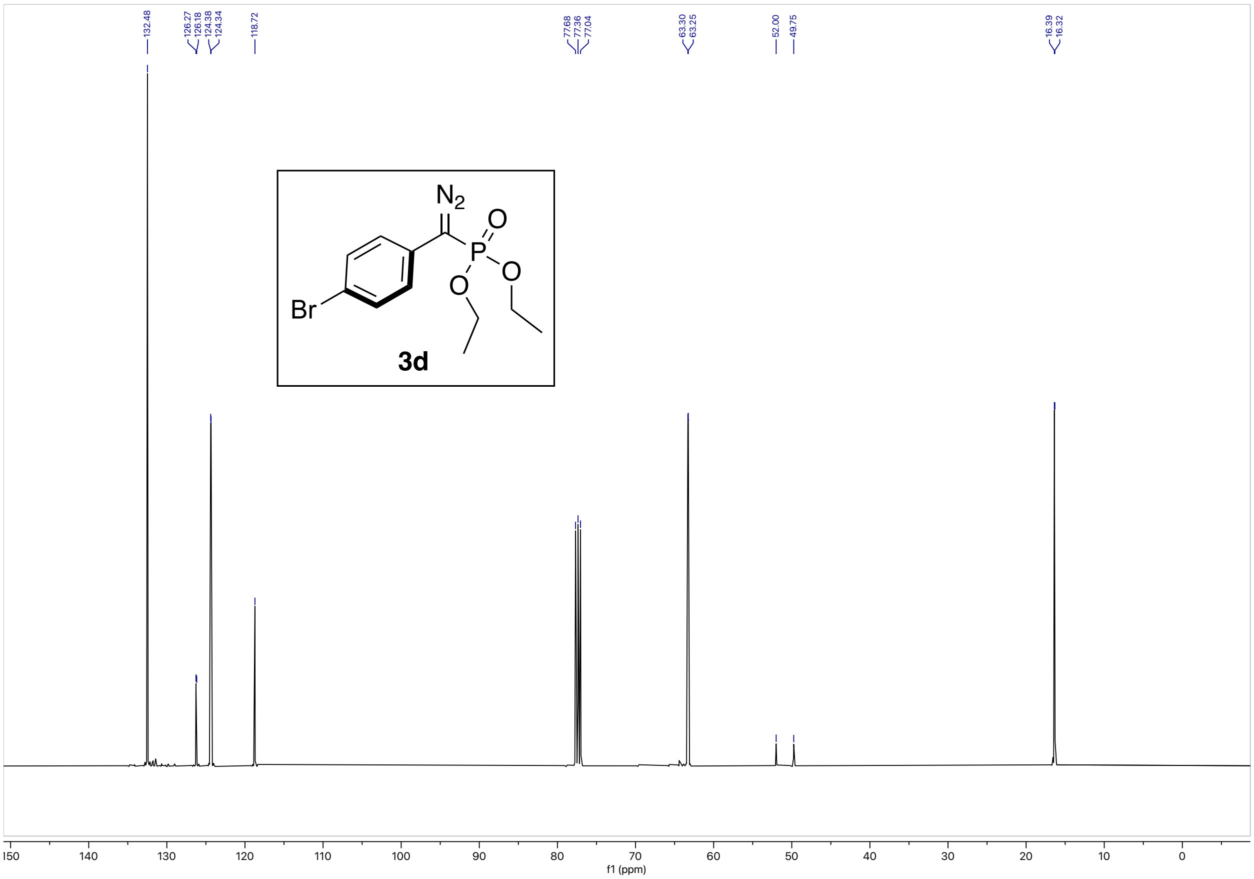

$^{13}C\{^{1}H\}$ NMR spectrum (101 MHz, Chloroform-*d*) (t, 77.36 ppm) of **3d.**

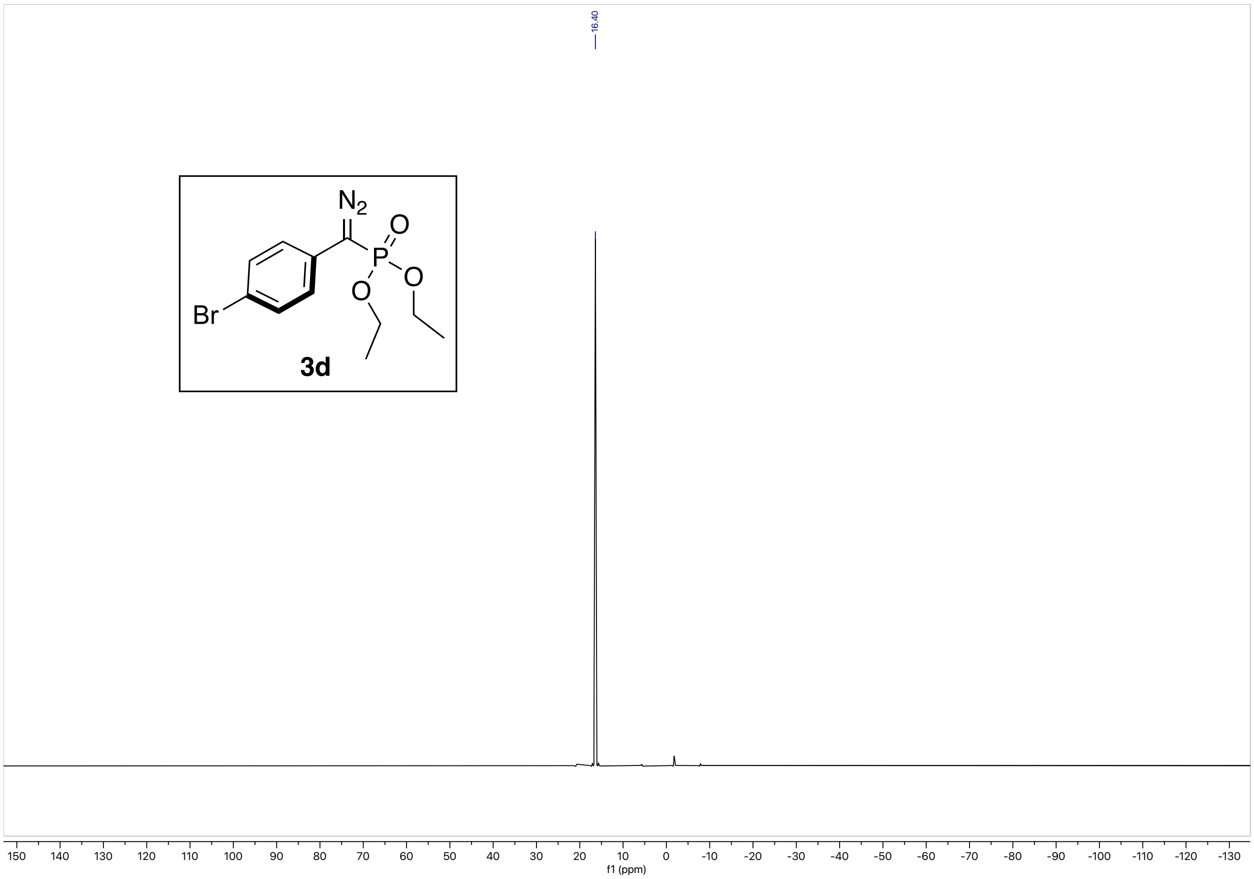

^{31}P NMR spectrum (162 MHz, Chloroform-d) of **3d.**

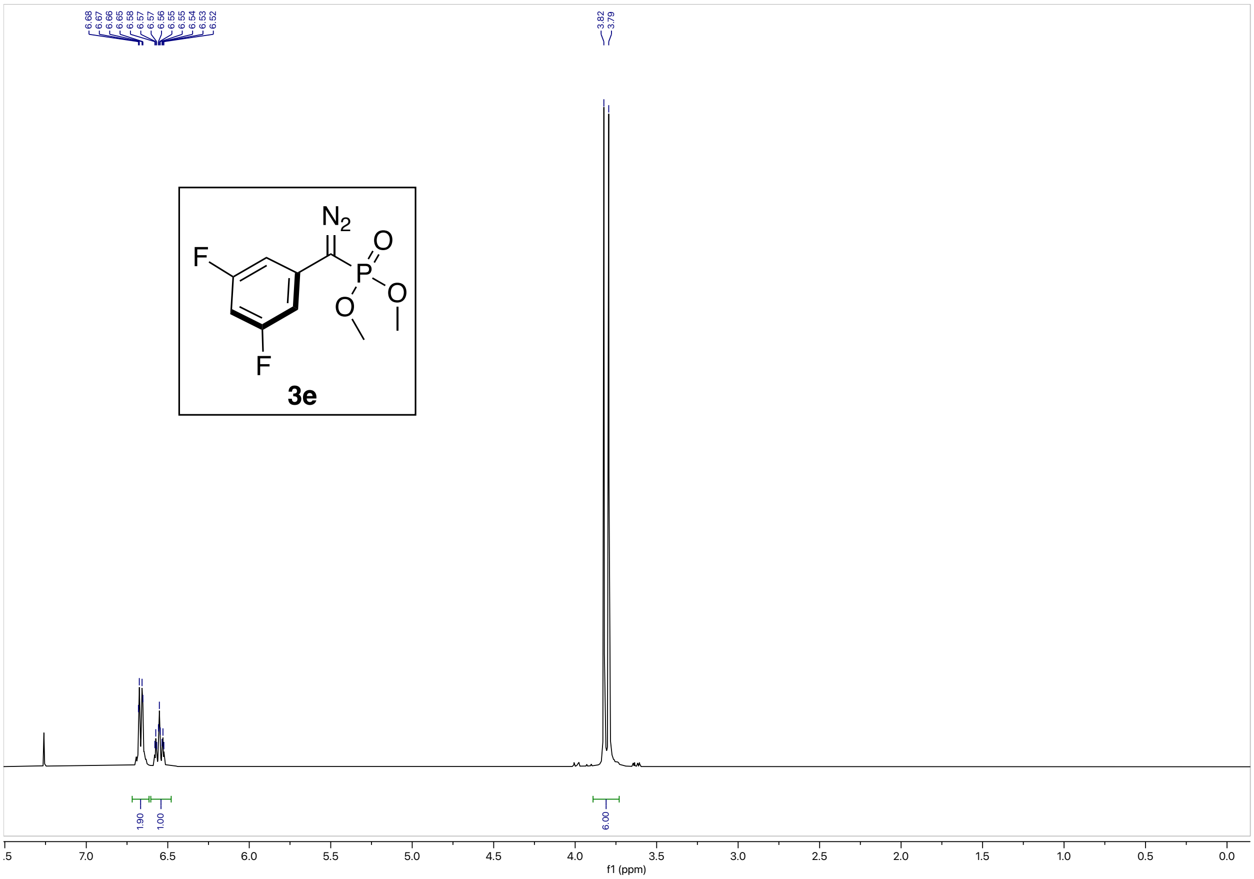

^{1}H NMR spectrum (400 MHz, Chloroform-*d*) (s, 7.26 ppm) of **3e.**

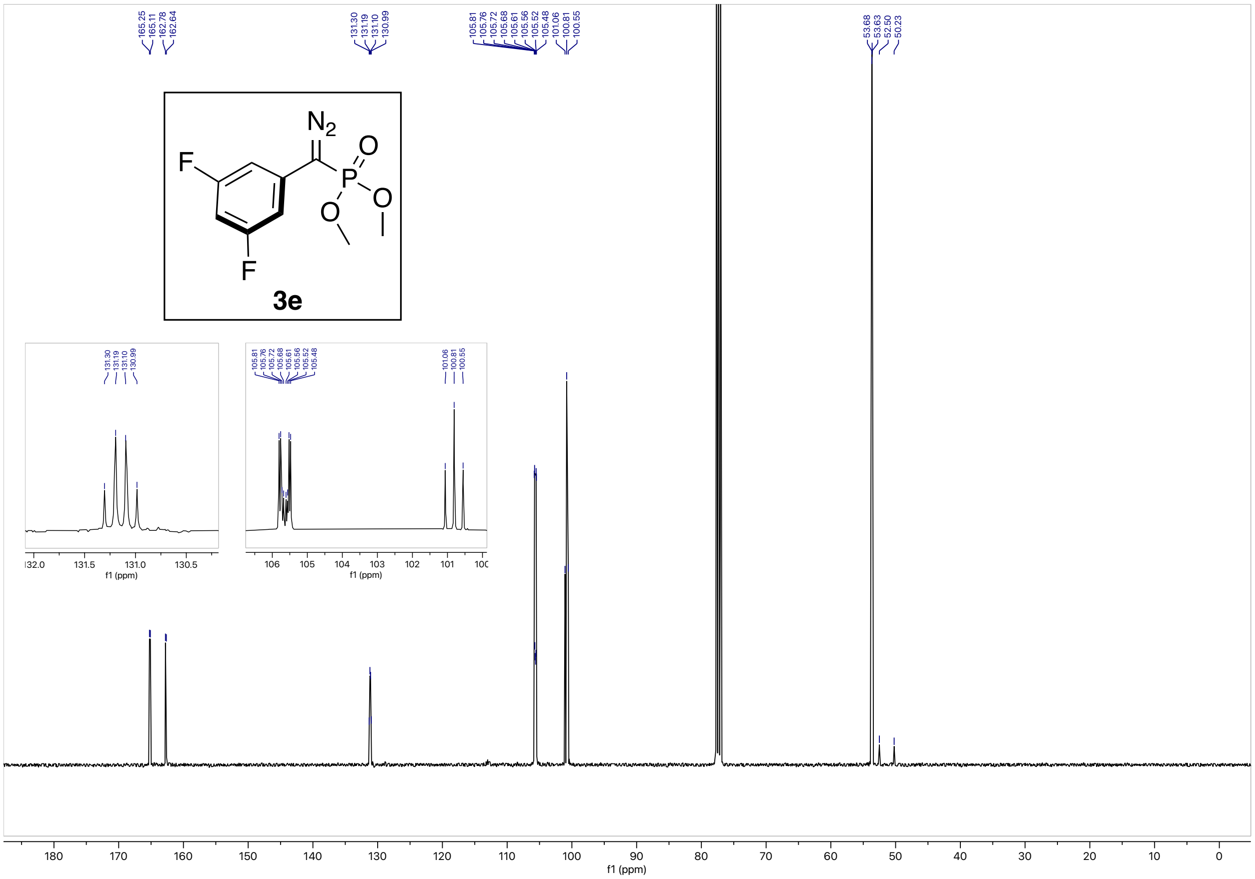

$^{13}C\{^1H\}$ NMR spectrum (101 MHz, Chloroform-*d*) (t, 77.36 ppm) of **3e.**

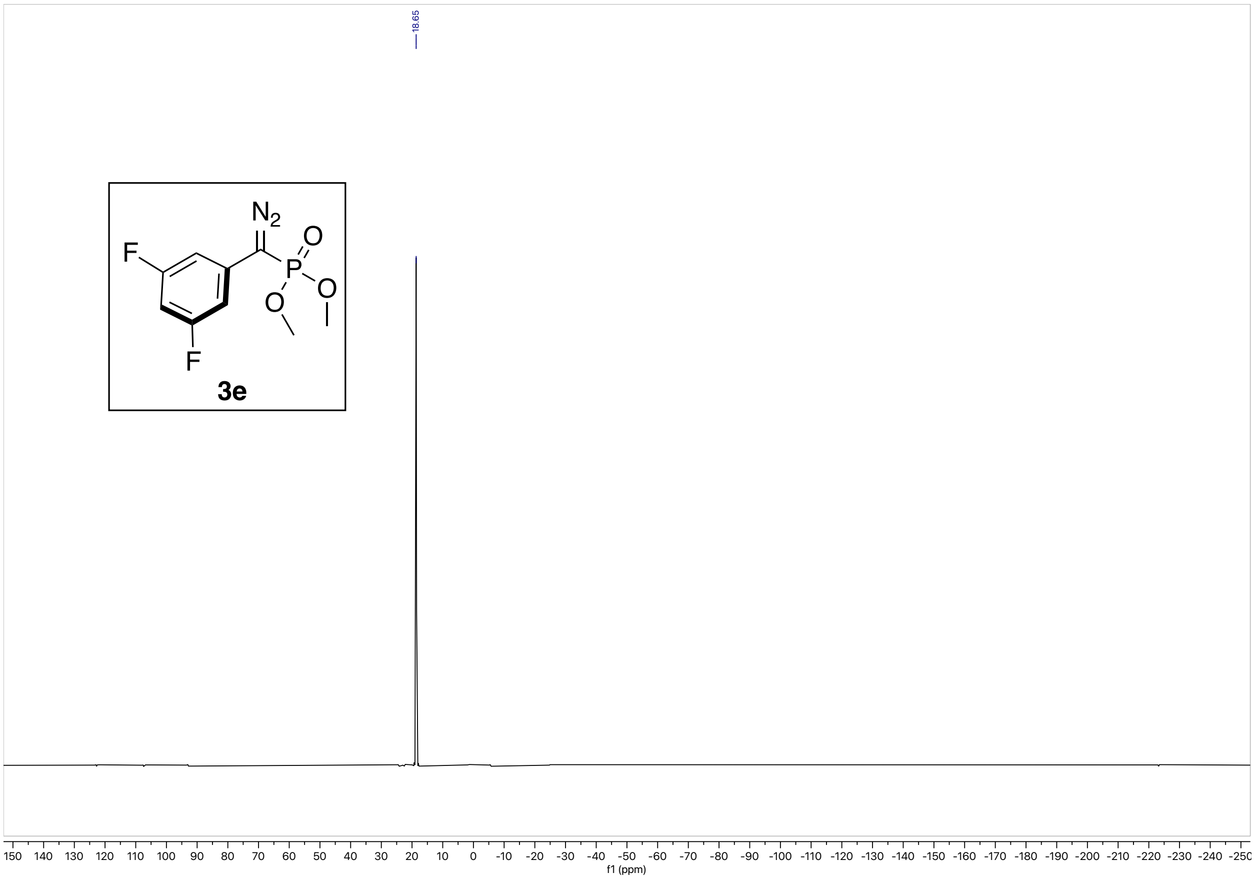

^{31}P NMR spectrum (162 MHz, Chloroform-d) of **3e.**

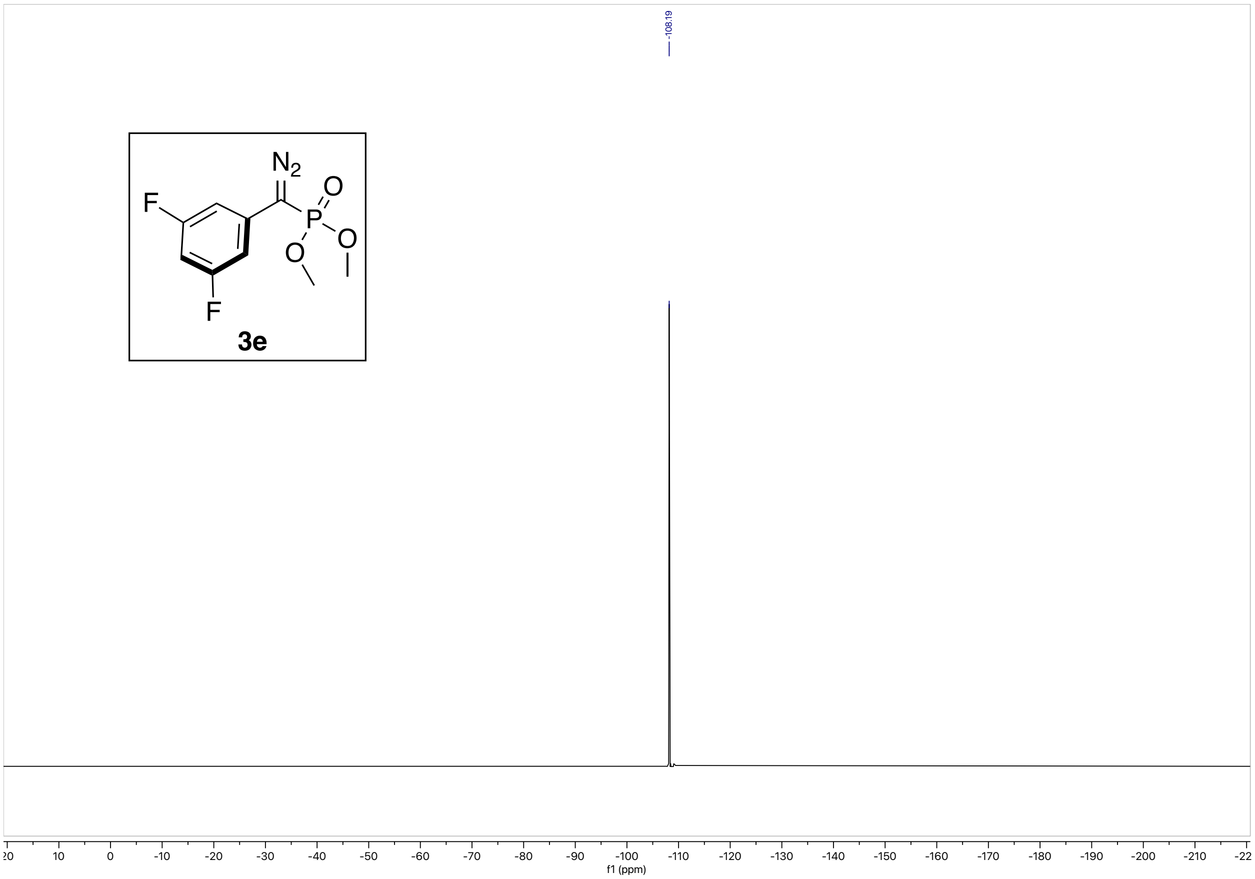

^{19}F NMR spectrum (376 MHz, Chloroform-d) of **3e.**

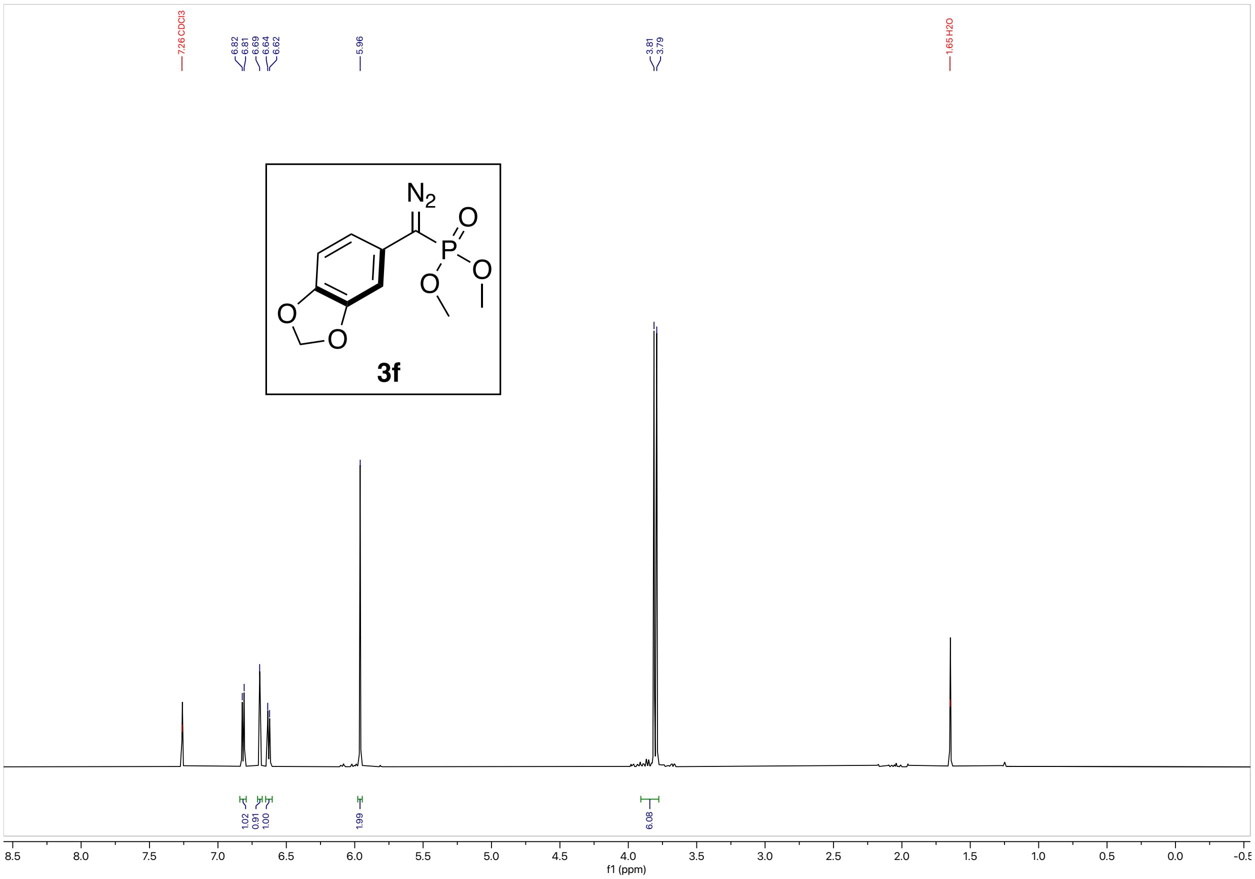

^{1}H NMR spectrum (600 MHz, Chloroform-*d*) (s, 7.26 ppm) of **3f.**

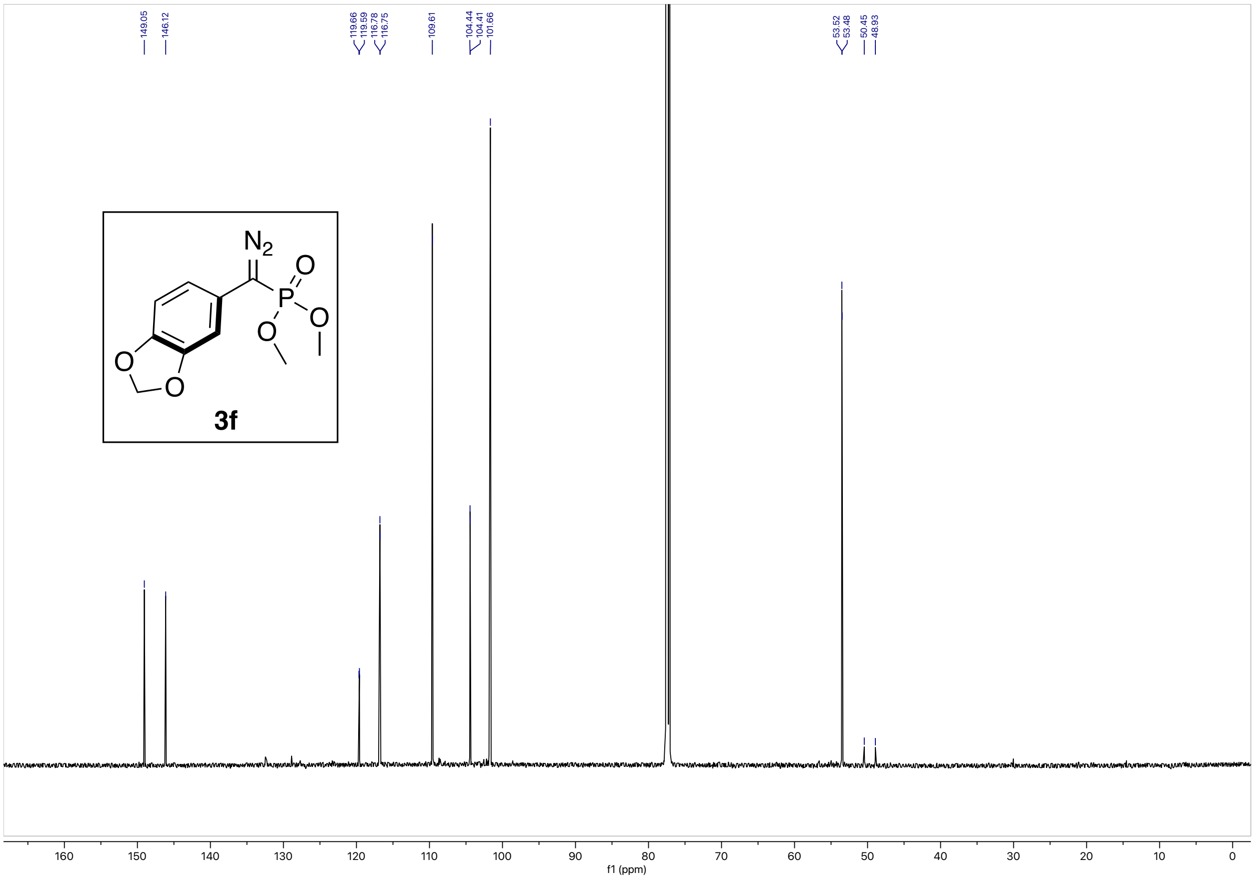

$^{13}C\{^{1}H\}$ NMR spectrum (151 MHz, Chloroform-*d*) (t, 77.36 ppm) of **3f.**

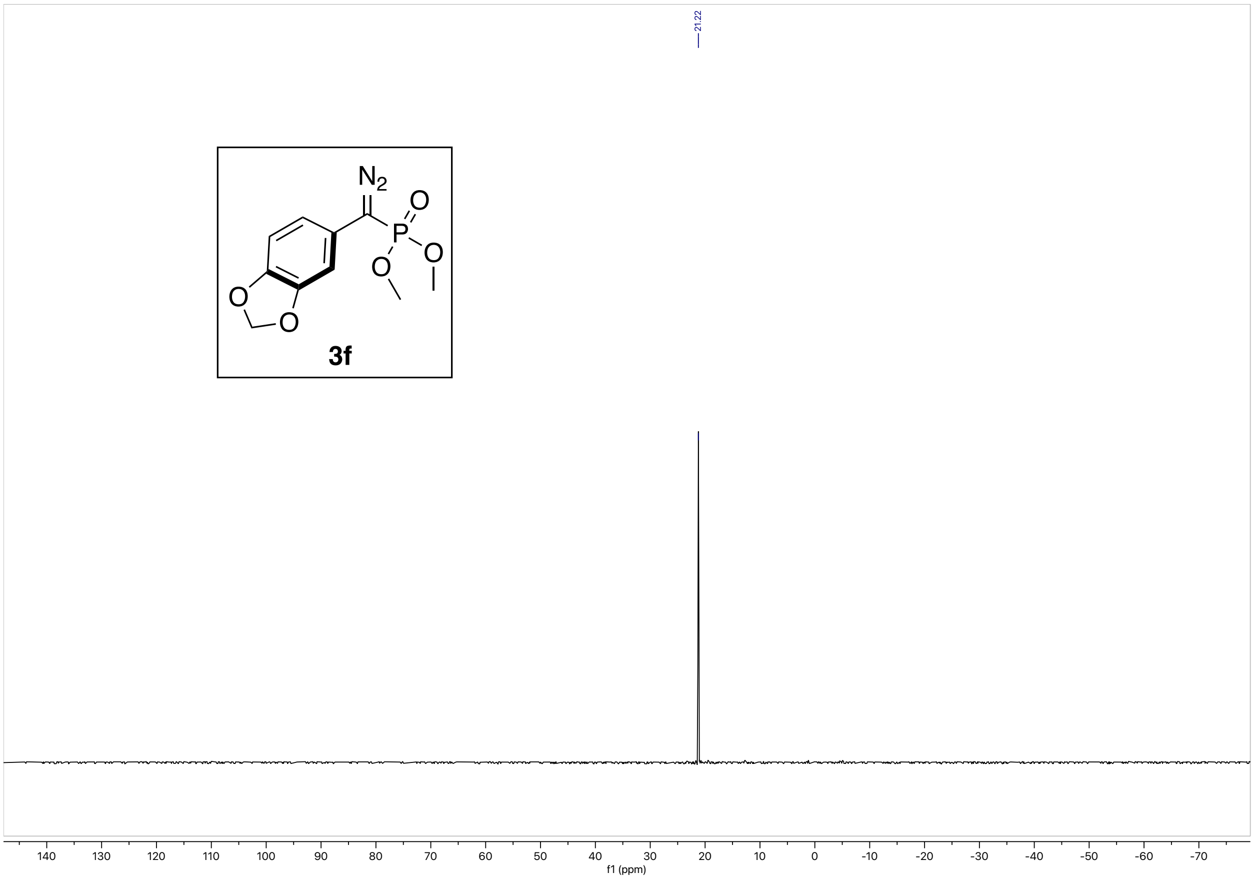

^{31}P NMR spectrum (243 MHz, Chloroform-d) of **3f.**

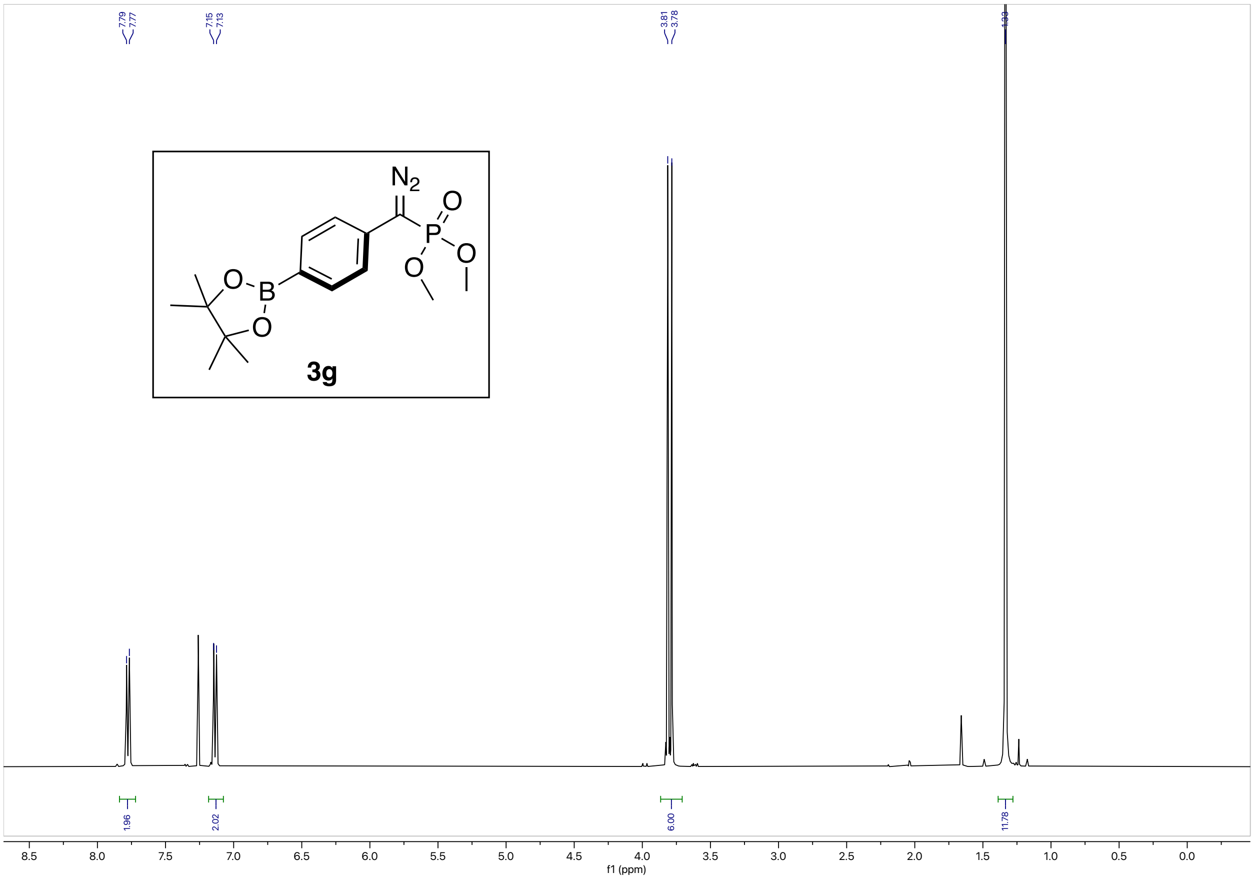

^{1}H NMR spectrum (400 MHz, Chloroform-*d*) (s, 7.26 ppm) of **3g.**

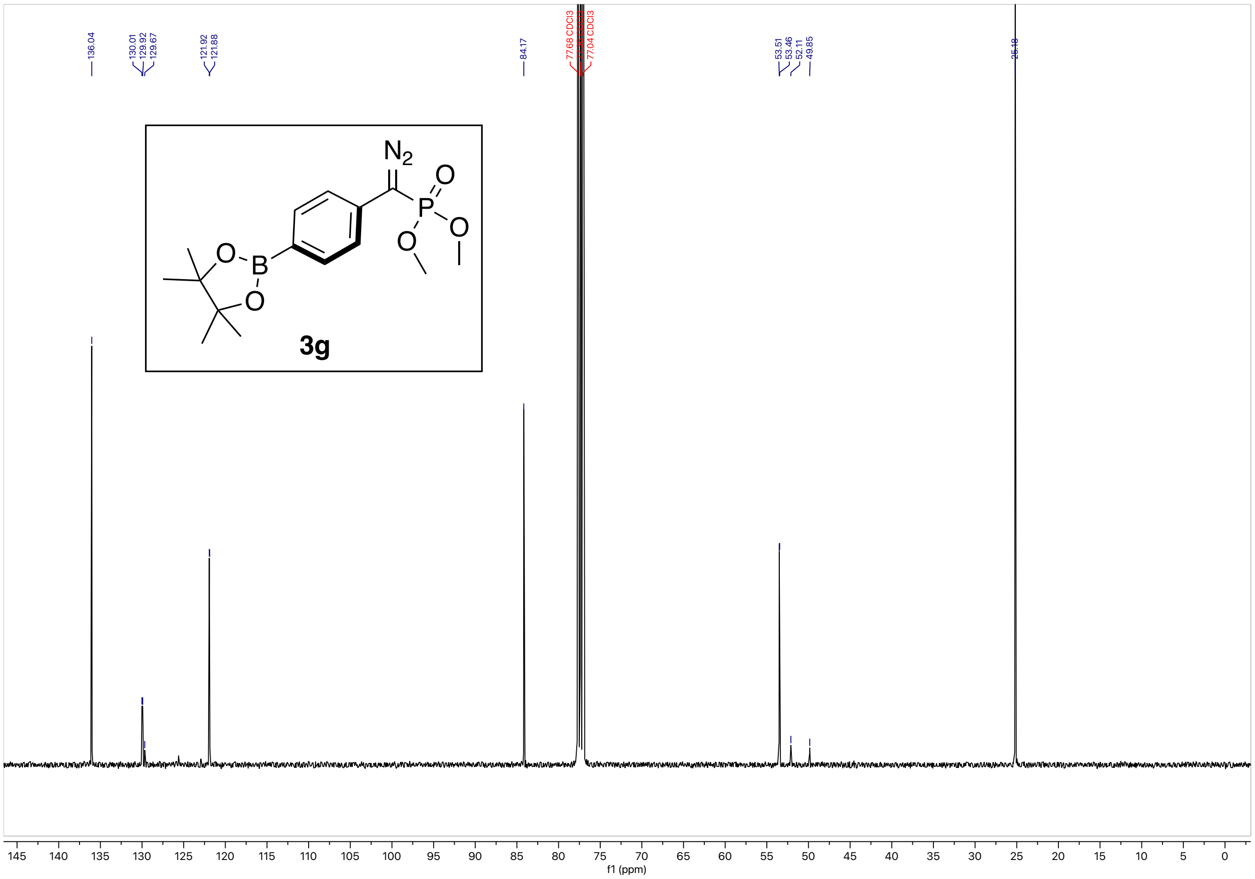

$^{13}C\{^{1}H\}$ NMR spectrum (101 MHz, Chloroform-*d*) (t, 77.36 ppm) of **3g.**

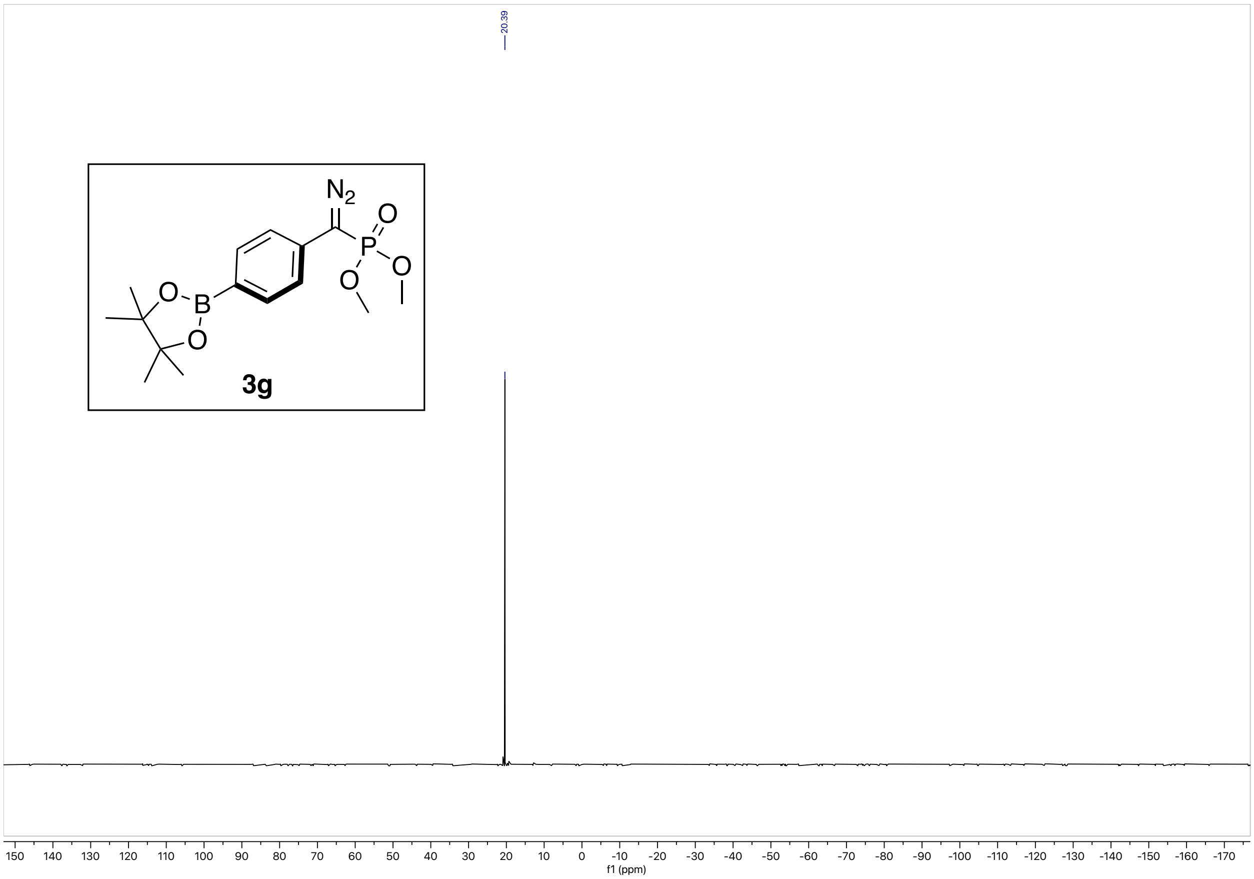

^{31}P NMR spectrum (162 MHz, Chloroform-d) of **3g.**

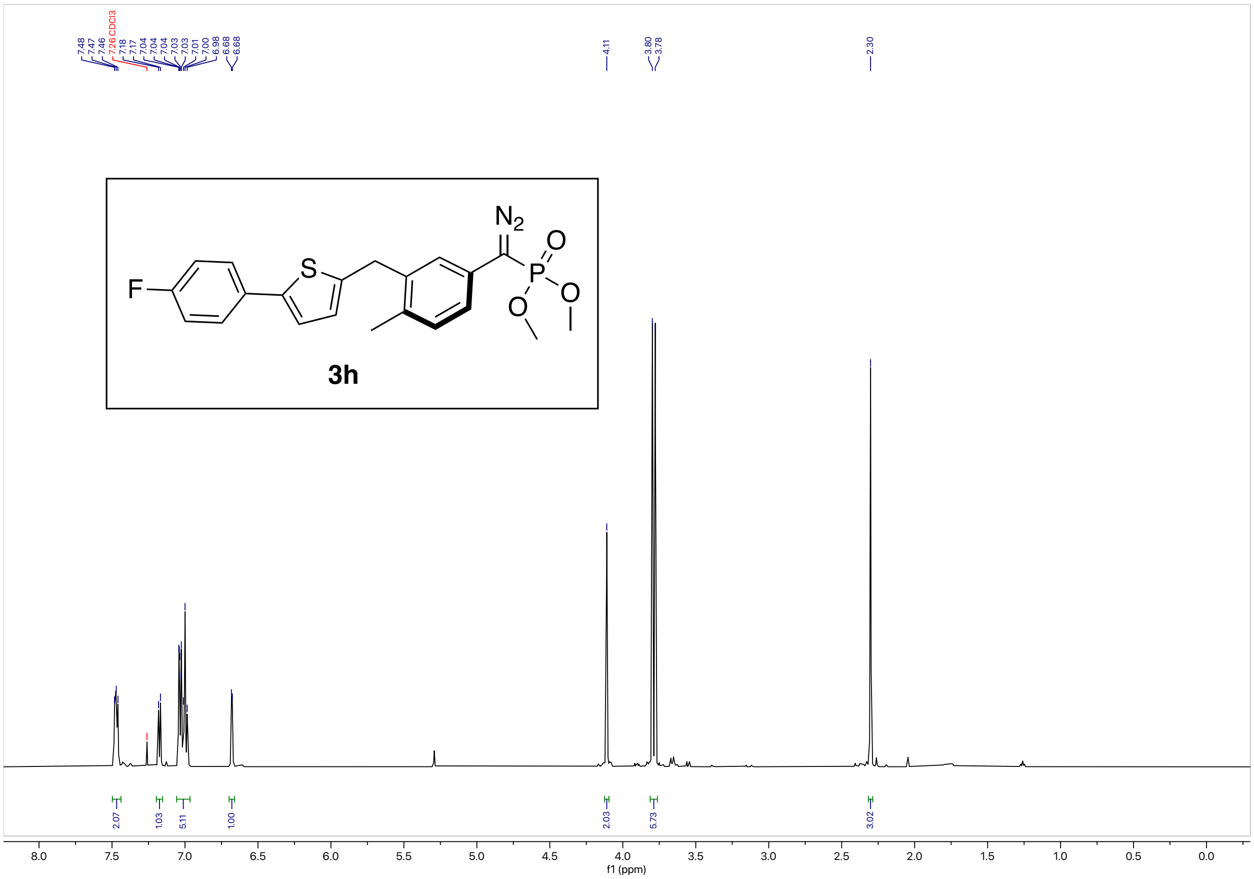

^{1}H NMR spectrum (600 MHz, Chloroform-*d*) (s, 7.26 ppm) of **3h.**

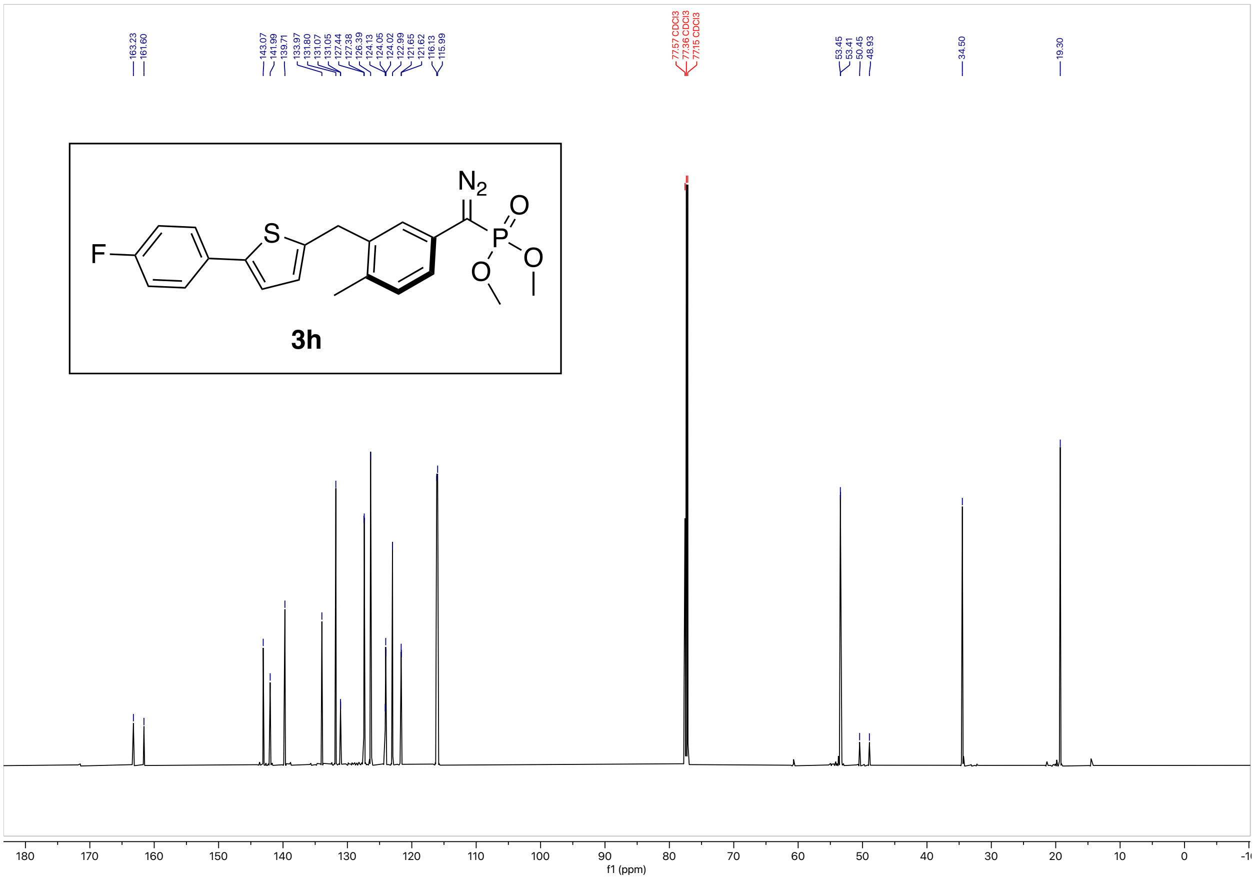

$^{13}C\{^1H\}$ NMR spectrum (151 MHz, Chloroform-*d*) (t, 77.36 ppm) of **3h.**

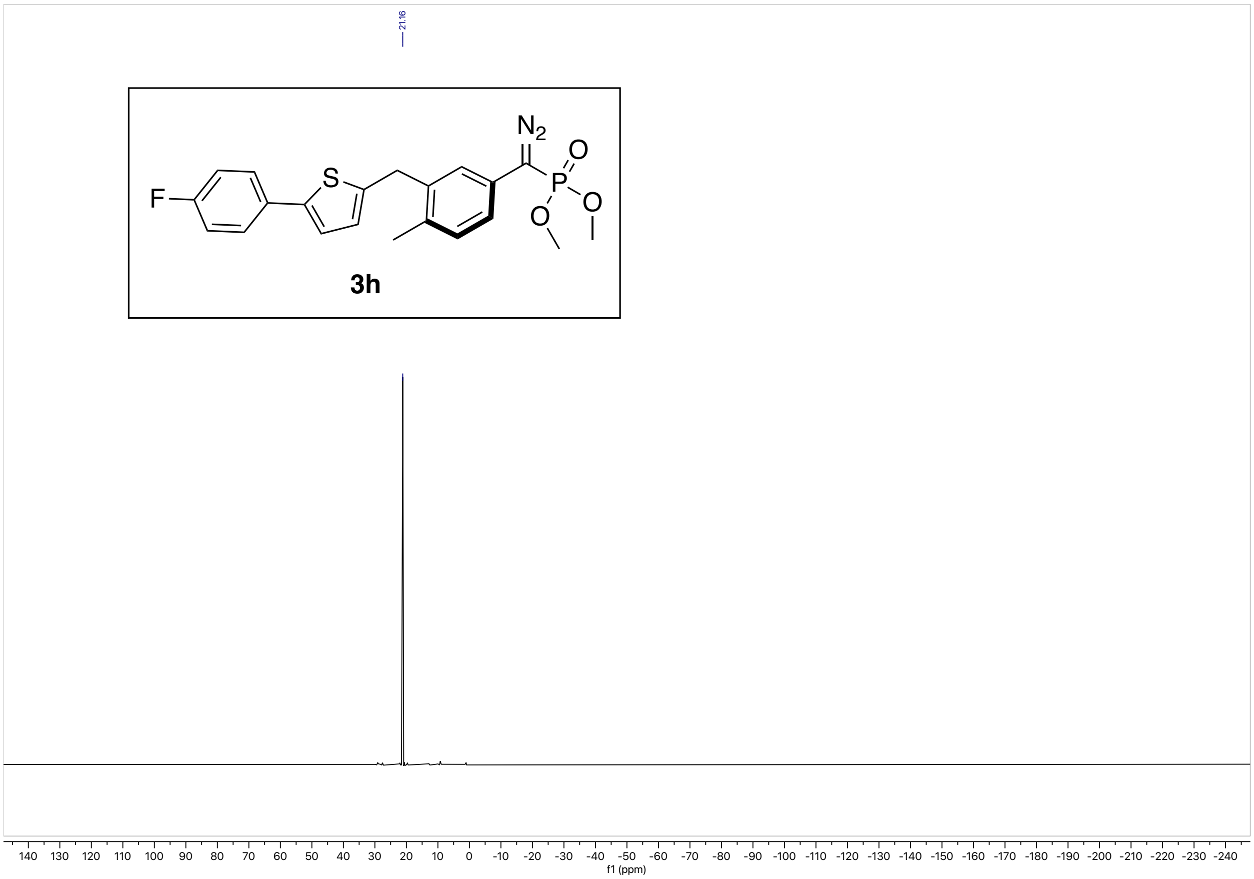

^{31}P NMR spectrum (243 MHz, Chloroform-d) of **3h.**

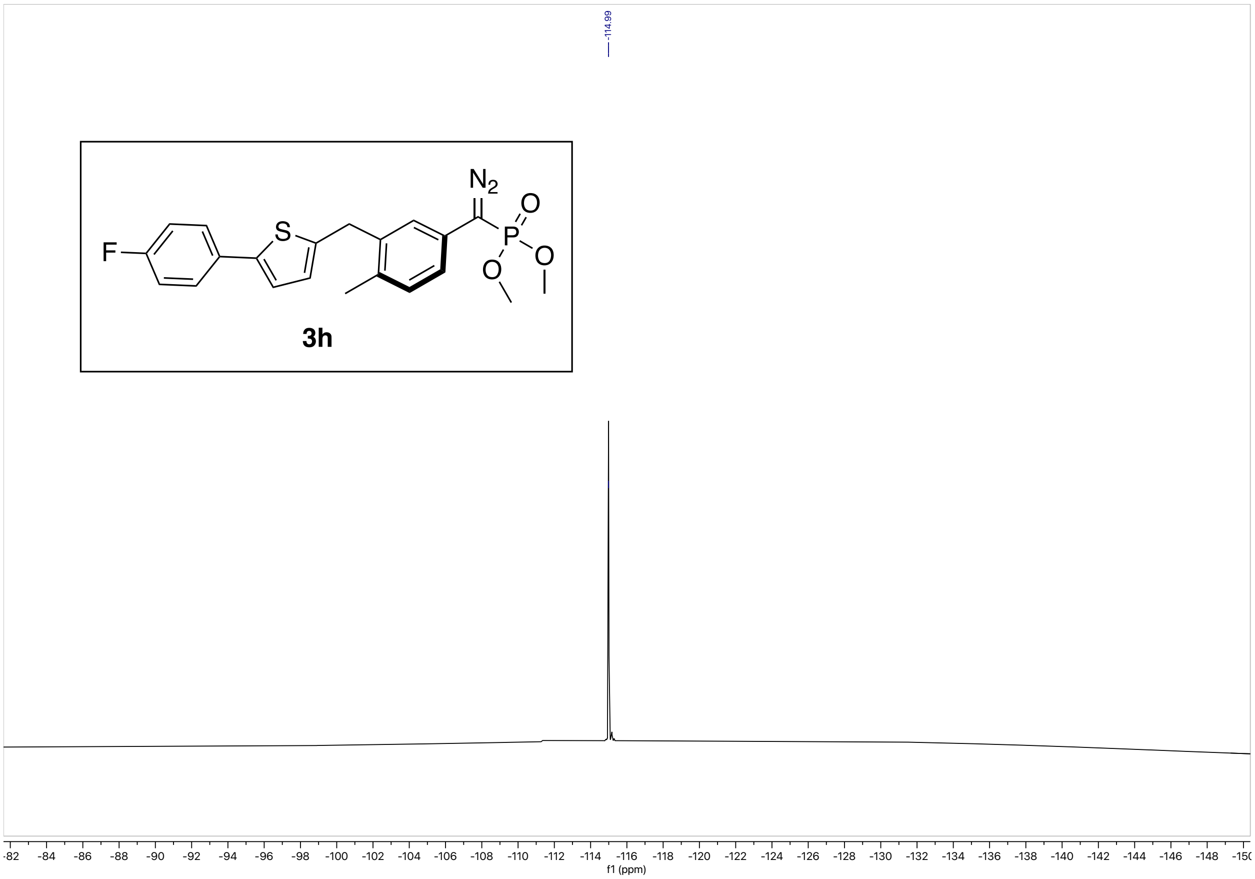

^{19}F NMR spectrum (565 MHz, Chloroform-d) of **3h.**

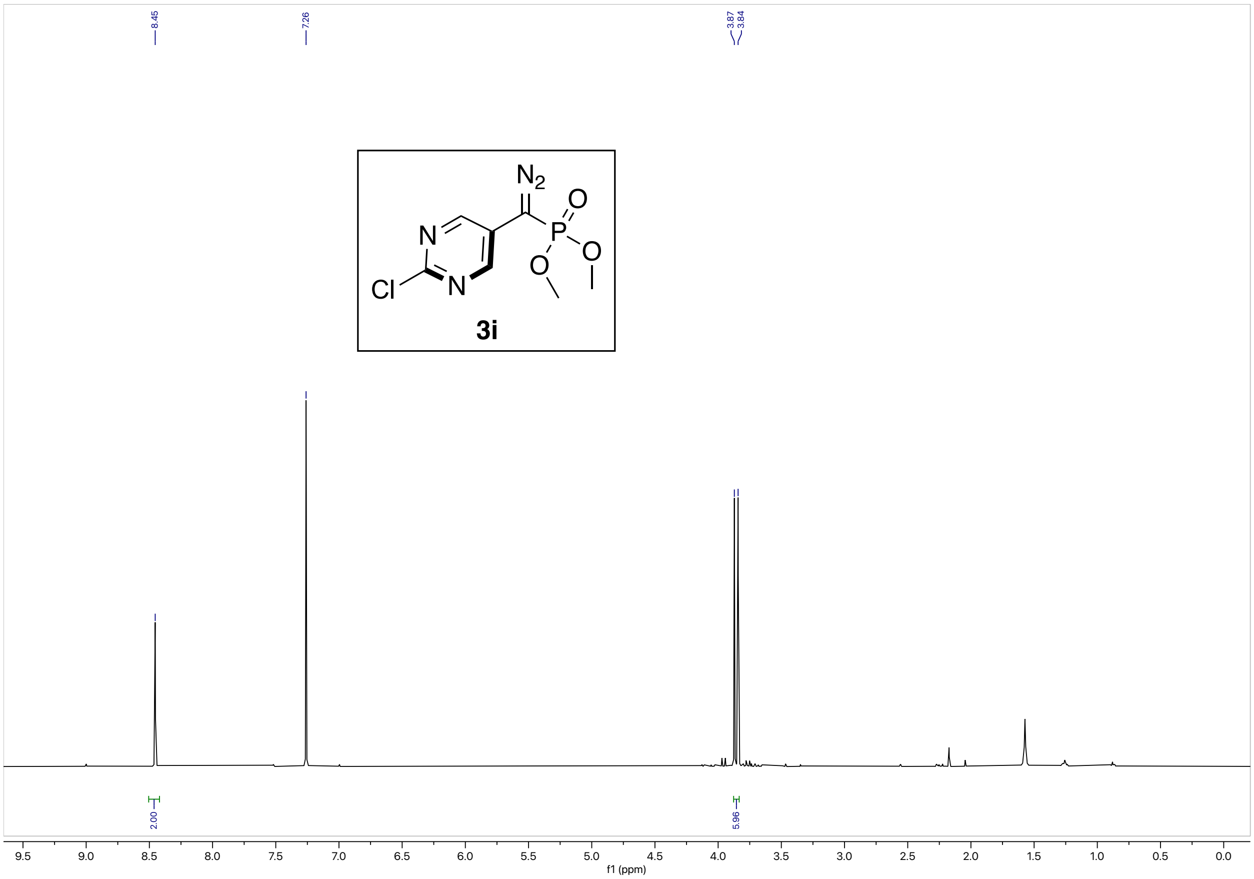

^{1}H NMR spectrum (600 MHz, Chloroform-*d*) (s, 7.26 ppm) of **3i.**

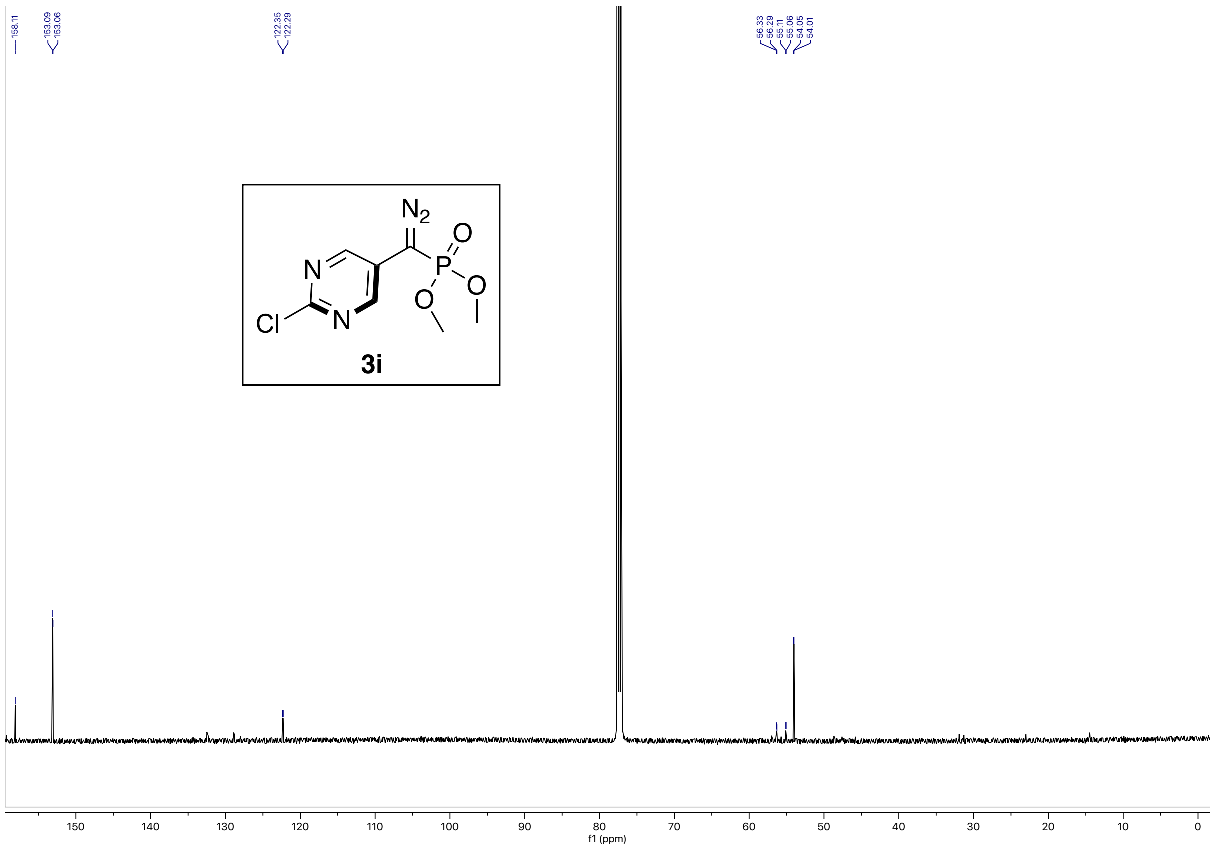

$^{13}C\{^{1}H\}$ NMR spectrum (151 MHz, Chloroform-*d*) (t, 77.36 ppm) of **3i.**

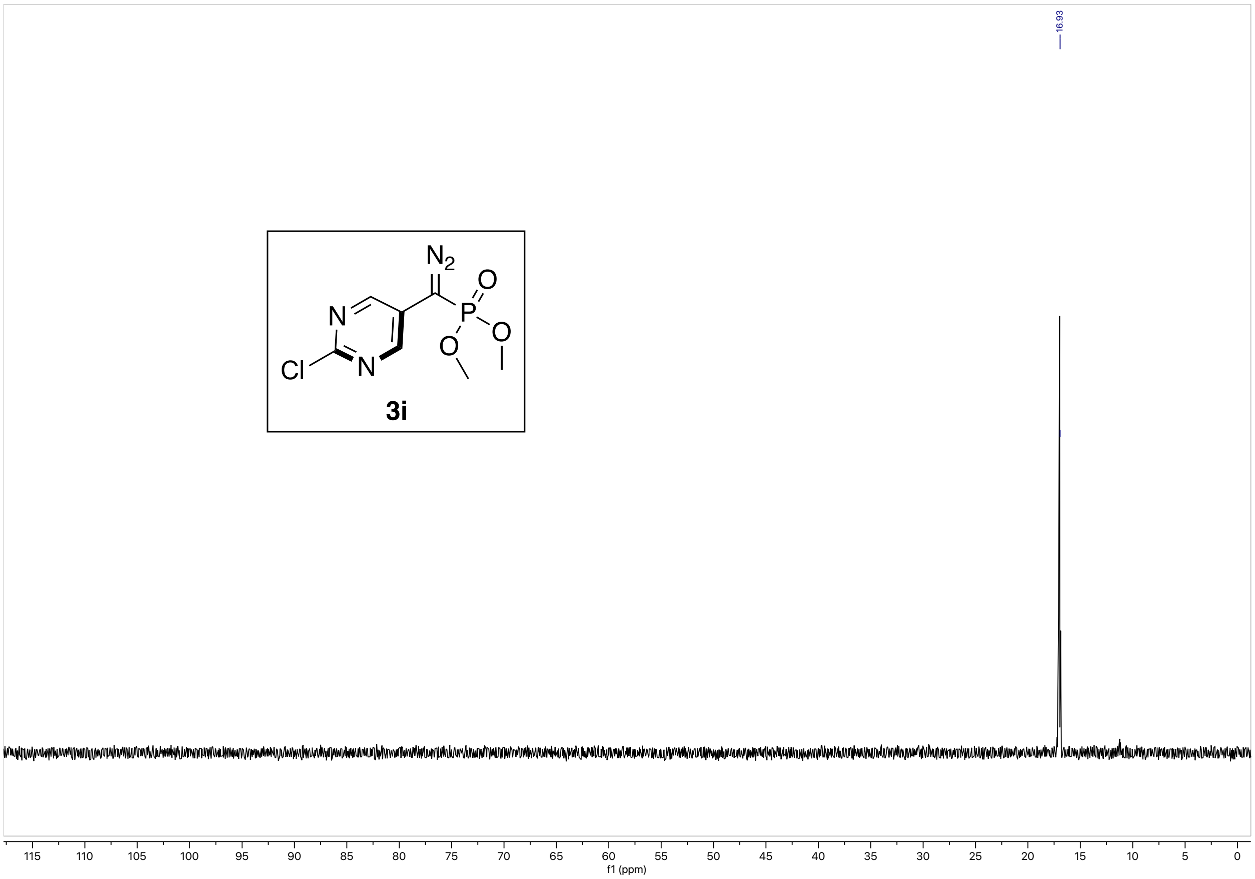

^{31}P NMR spectrum (243 MHz, Chloroform-d) of **3i.**

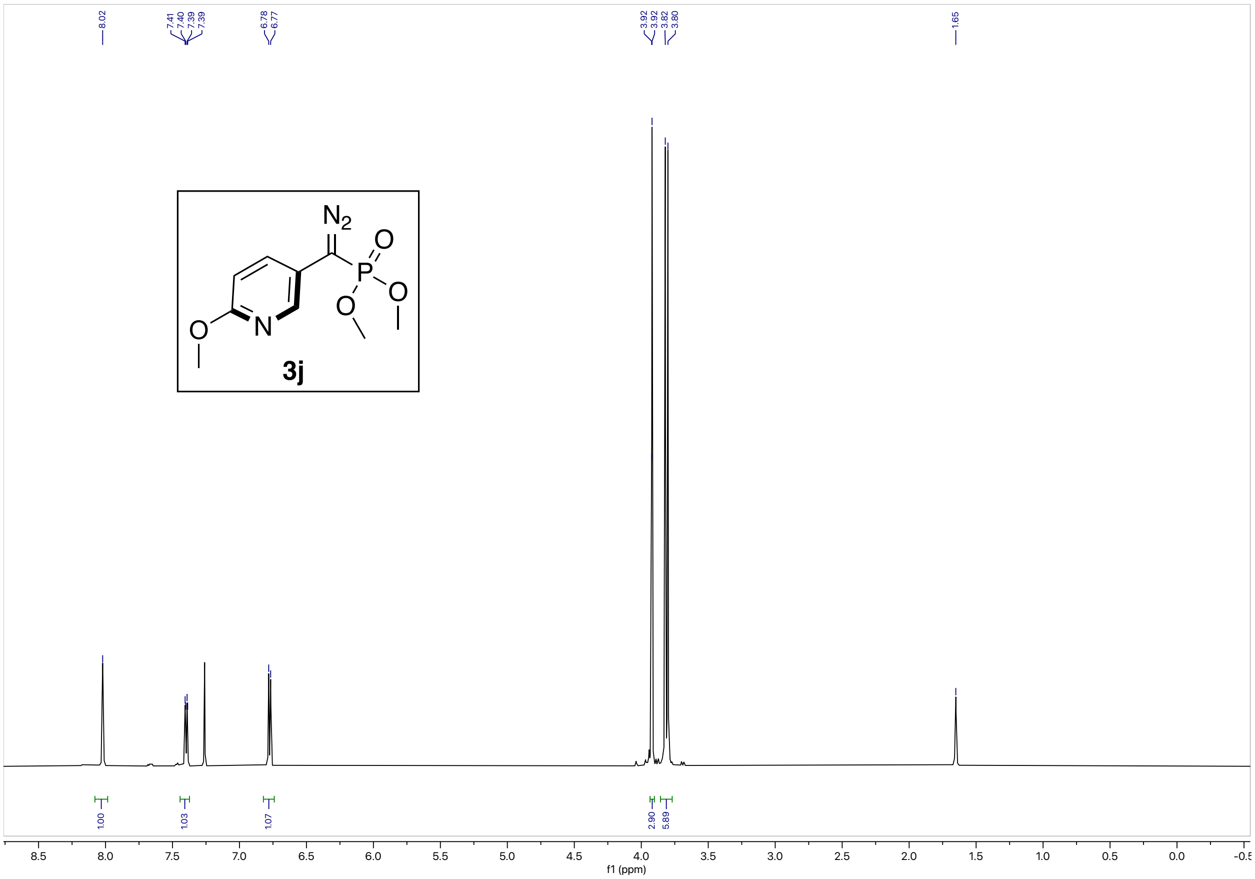

^{1}H NMR spectrum (600 MHz, Chloroform-*d*) (s, 7.26 ppm) of **3j.**

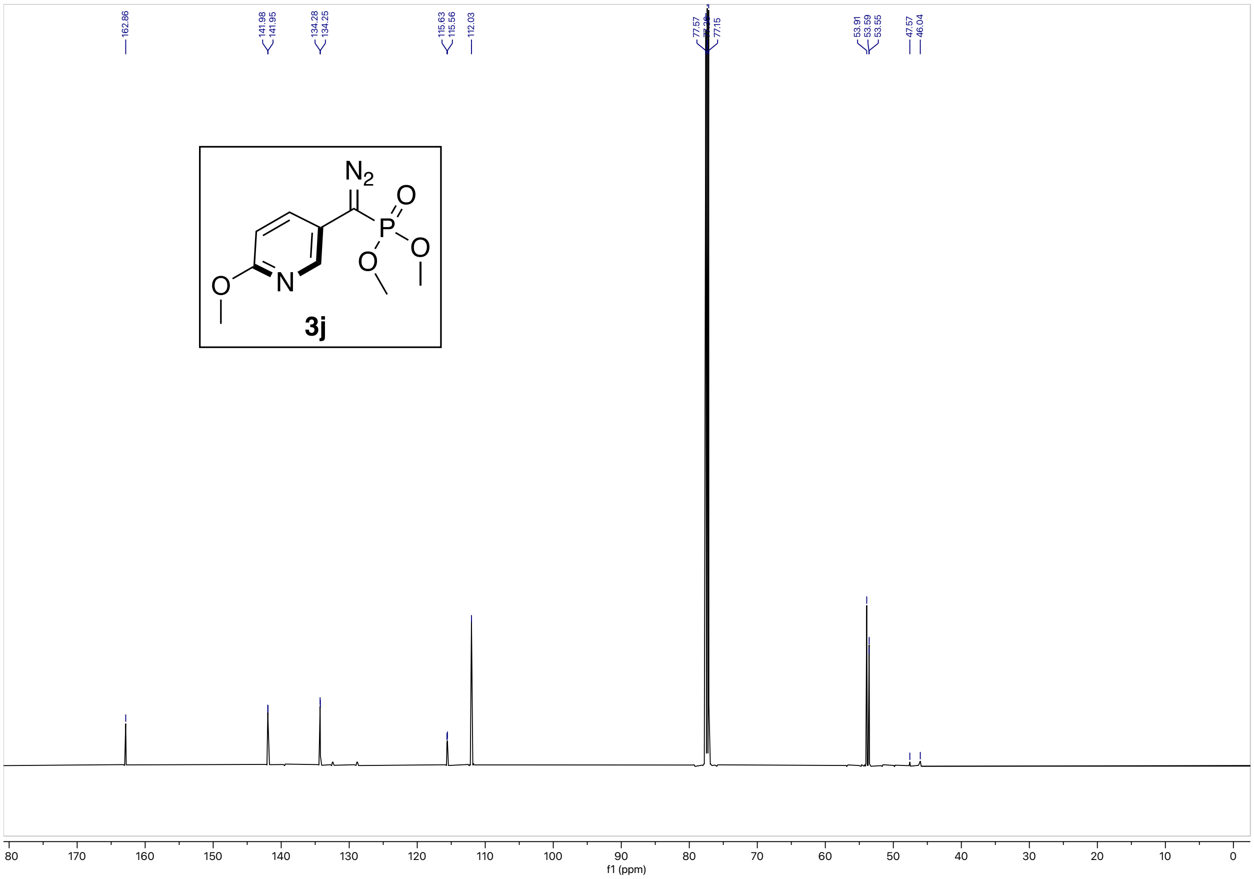

$^{13}C\{^{1}H\}$ NMR spectrum (151 MHz, Chloroform-*d*) (t, 77.36 ppm) of **3j.**

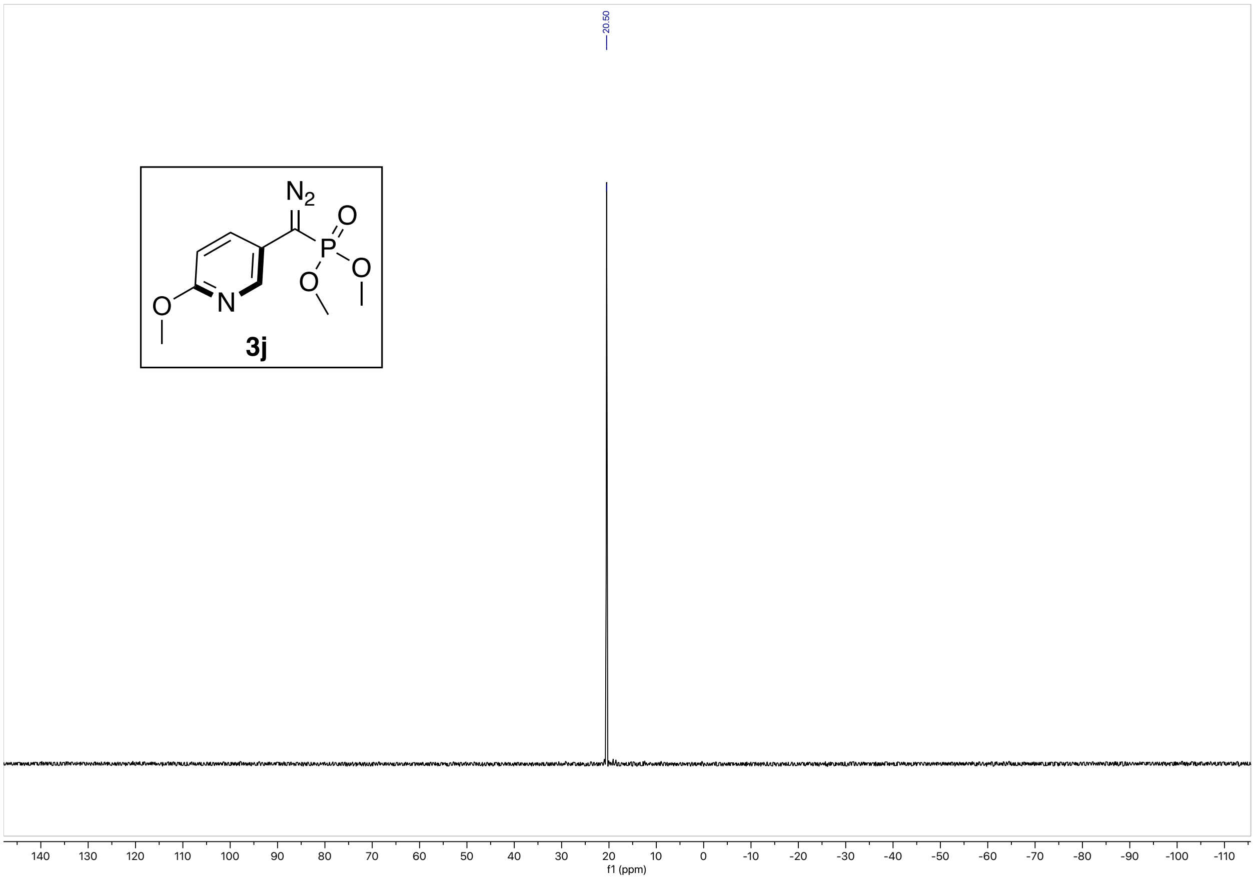

^{31}P NMR spectrum (243 MHz, Chloroform-d) of **3j.**

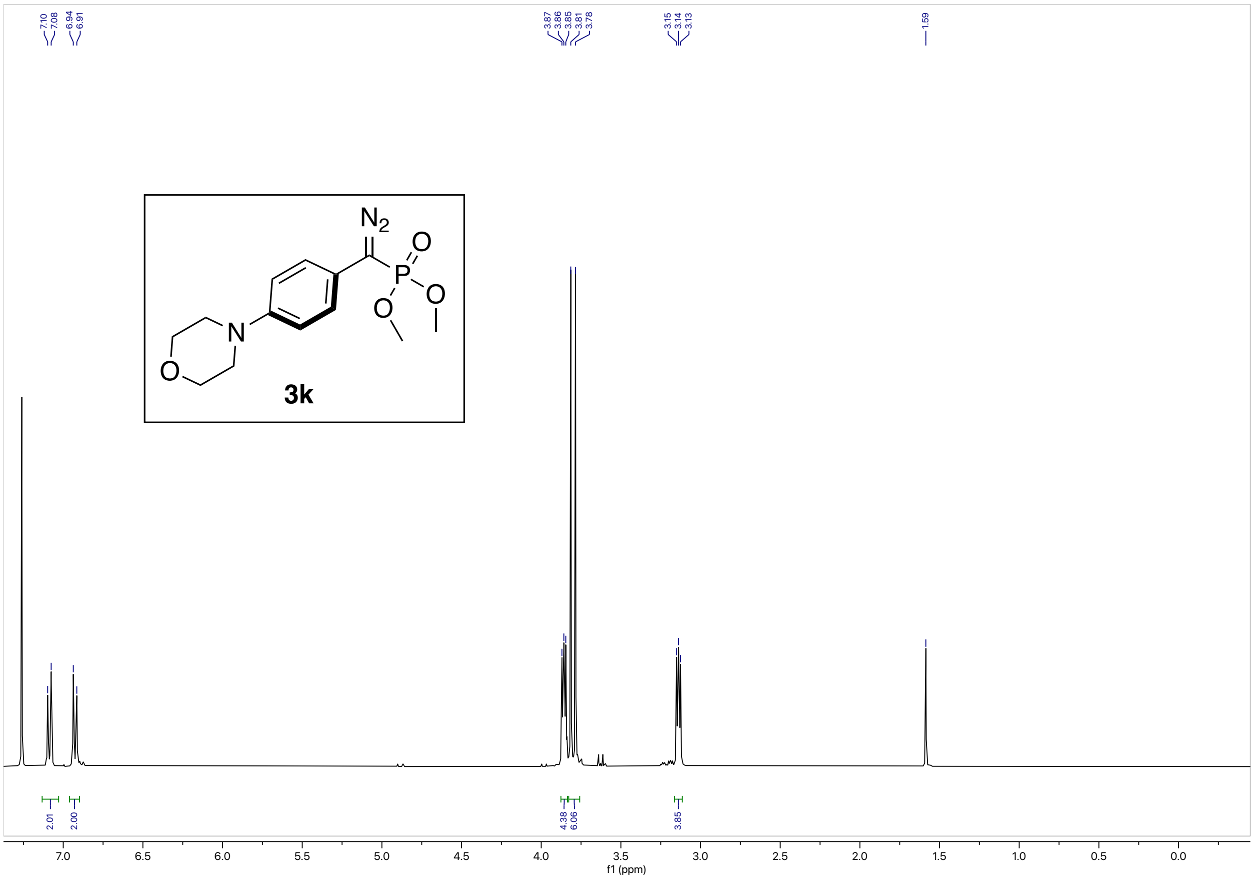

^{1}H NMR spectrum (400 MHz, Chloroform-*d*) (s, 7.26 ppm) of **3k.**

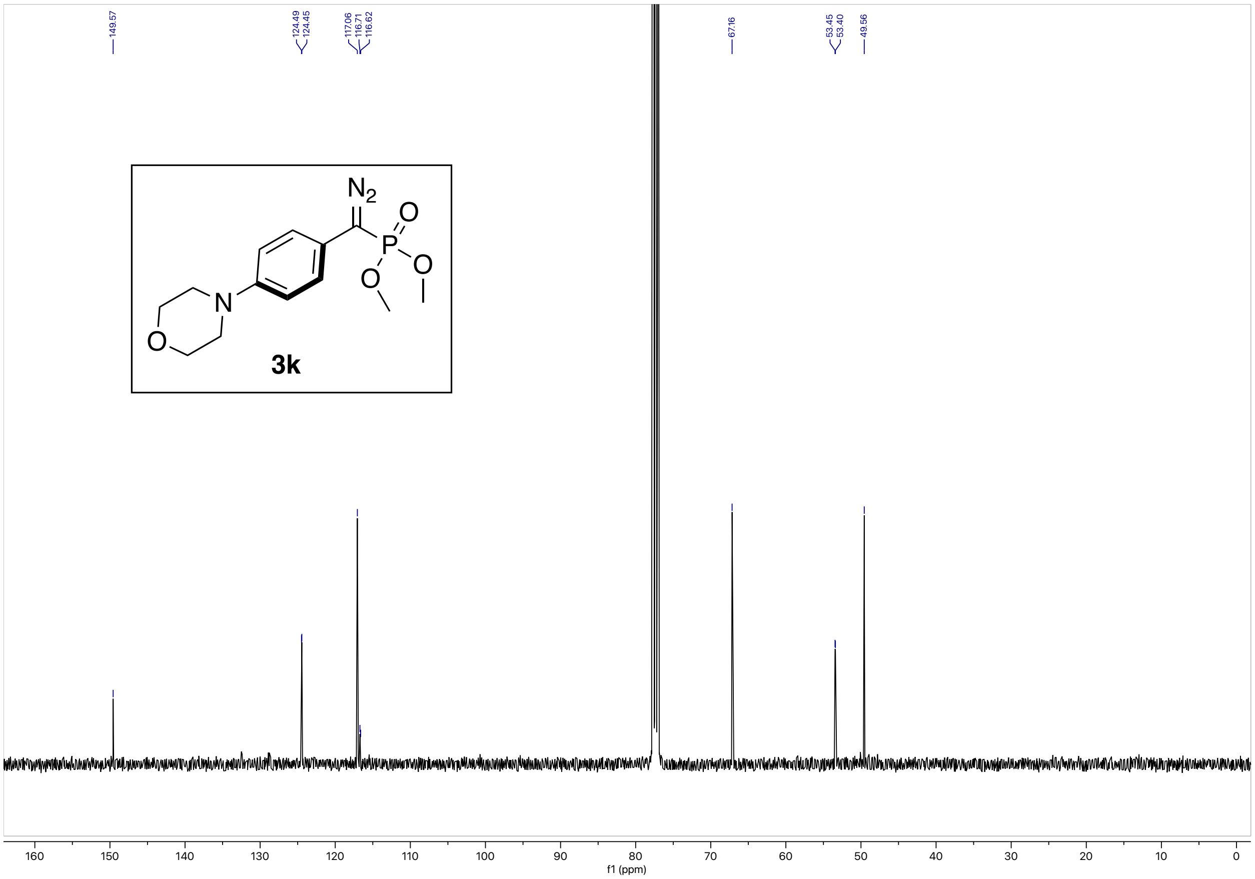

$^{13}C\{^{1}H\}$ NMR spectrum (101 MHz, Chloroform-*d*) (t, 77.36 ppm) of **3k.**

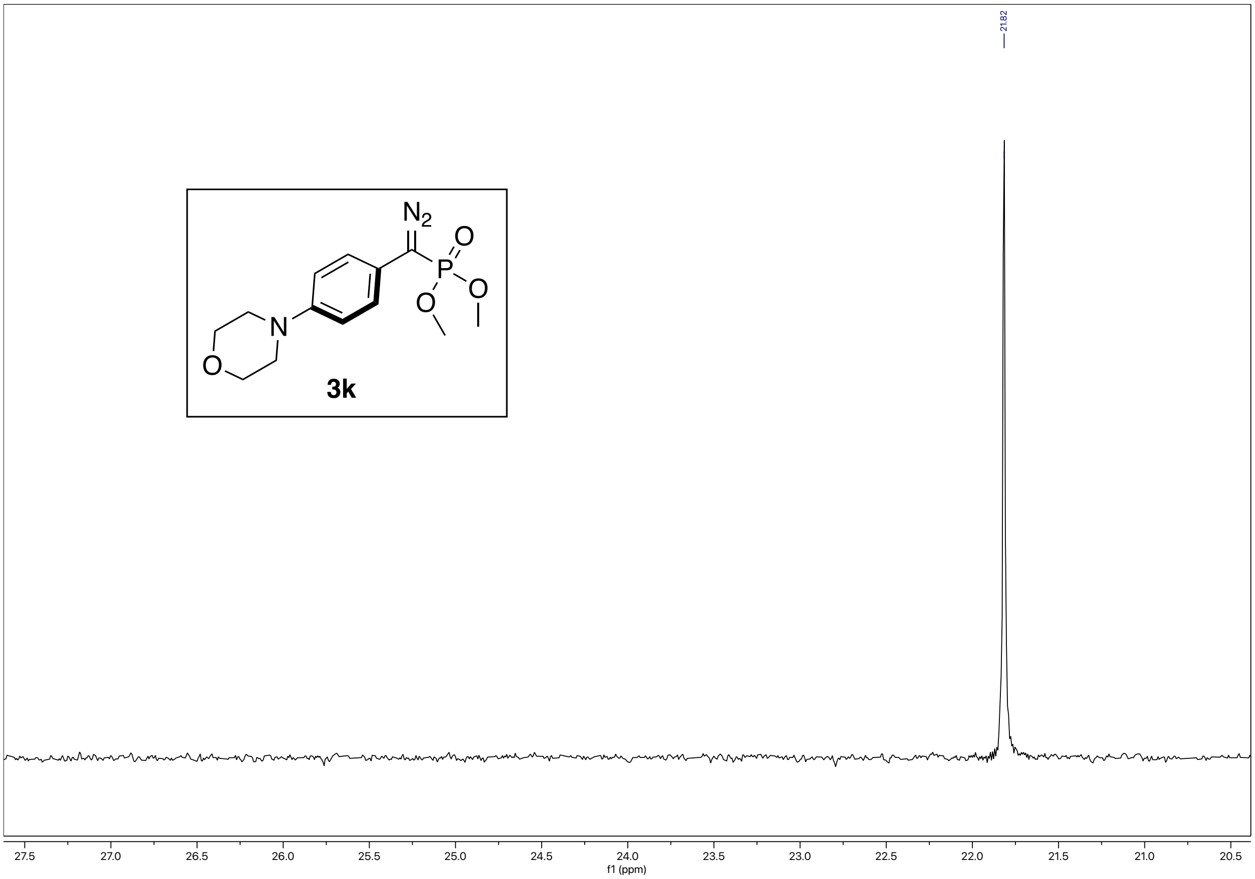

^{31}P NMR spectrum (162 MHz, Chloroform-d) of **3k.**

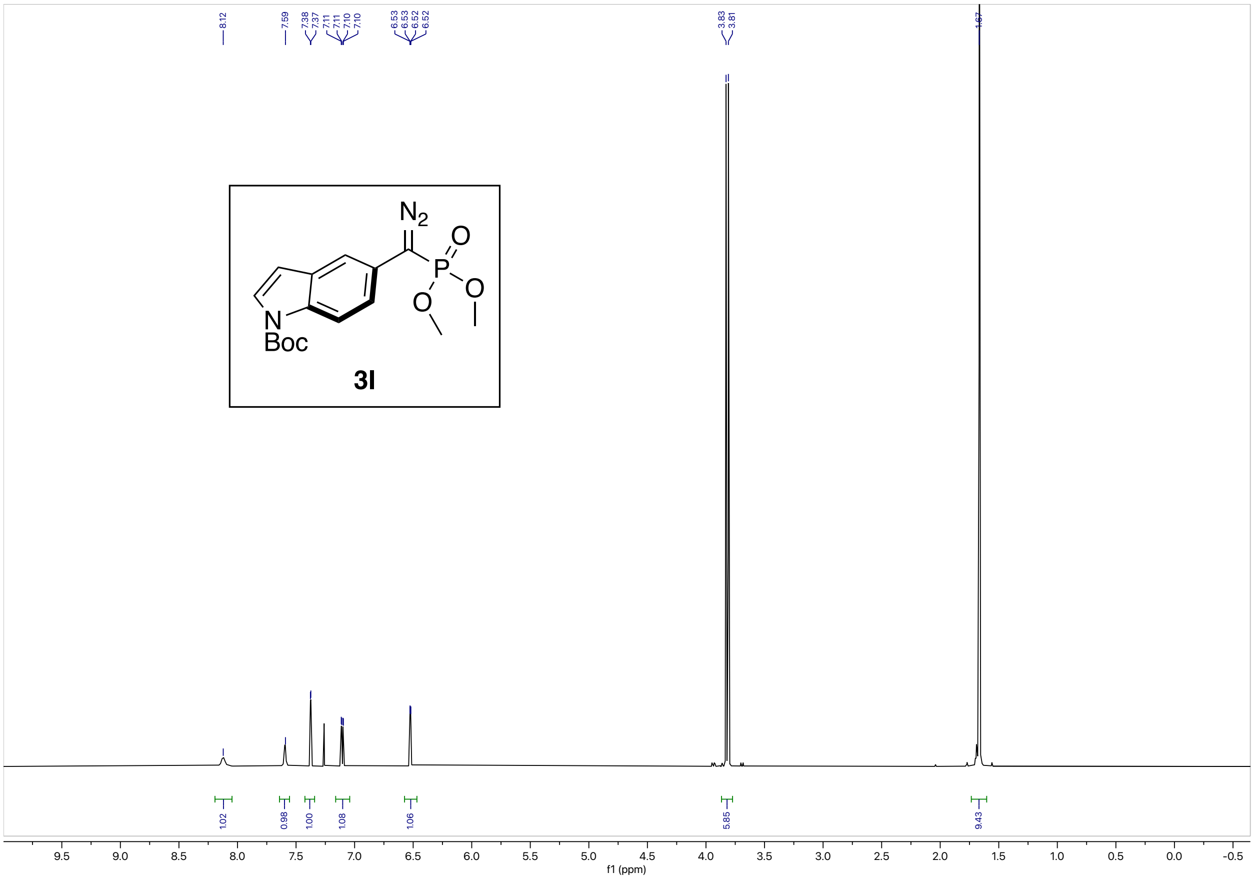

^{1}H NMR spectrum (600 MHz, Chloroform-*d*) (s, 7.26 ppm) of **3l.**

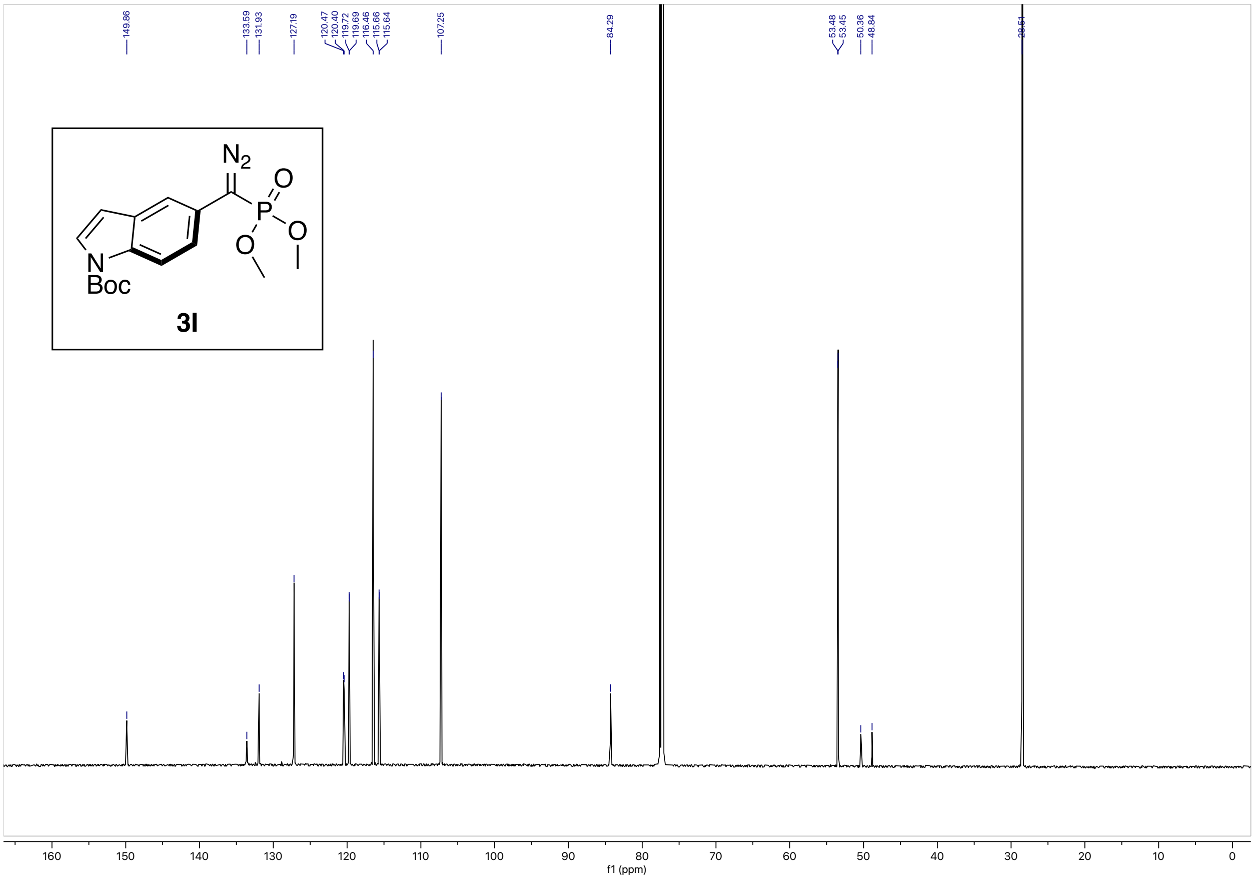

$^{13}C\{^{1}H\}$ NMR spectrum (151 MHz, Chloroform-*d*) (t, 77.36 ppm) of **3l.**

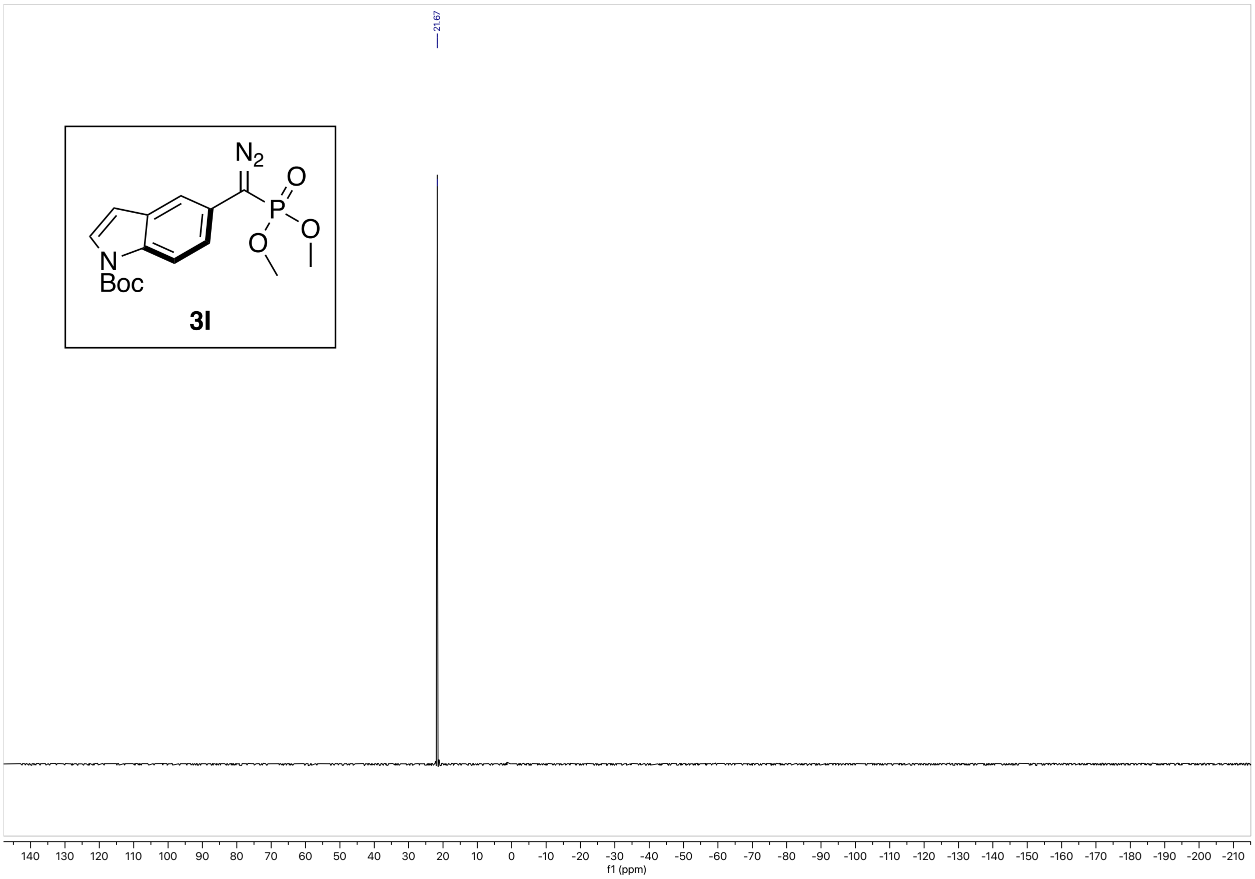

^{31}P NMR spectrum (243 MHz, Chloroform-d) of **3l.**

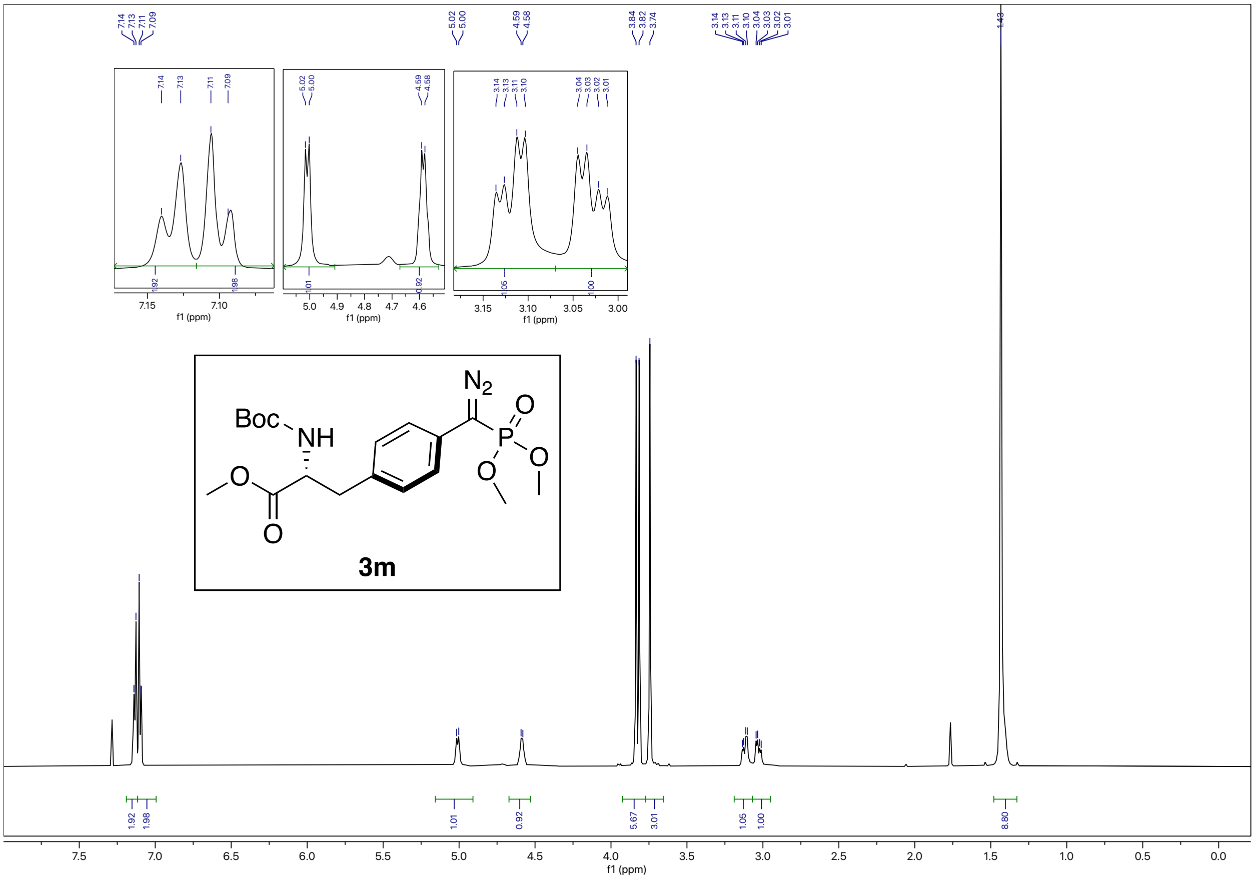

^{1}H NMR spectrum (600 MHz, Chloroform-*d*) (s, 7.26 ppm) of **3m.**

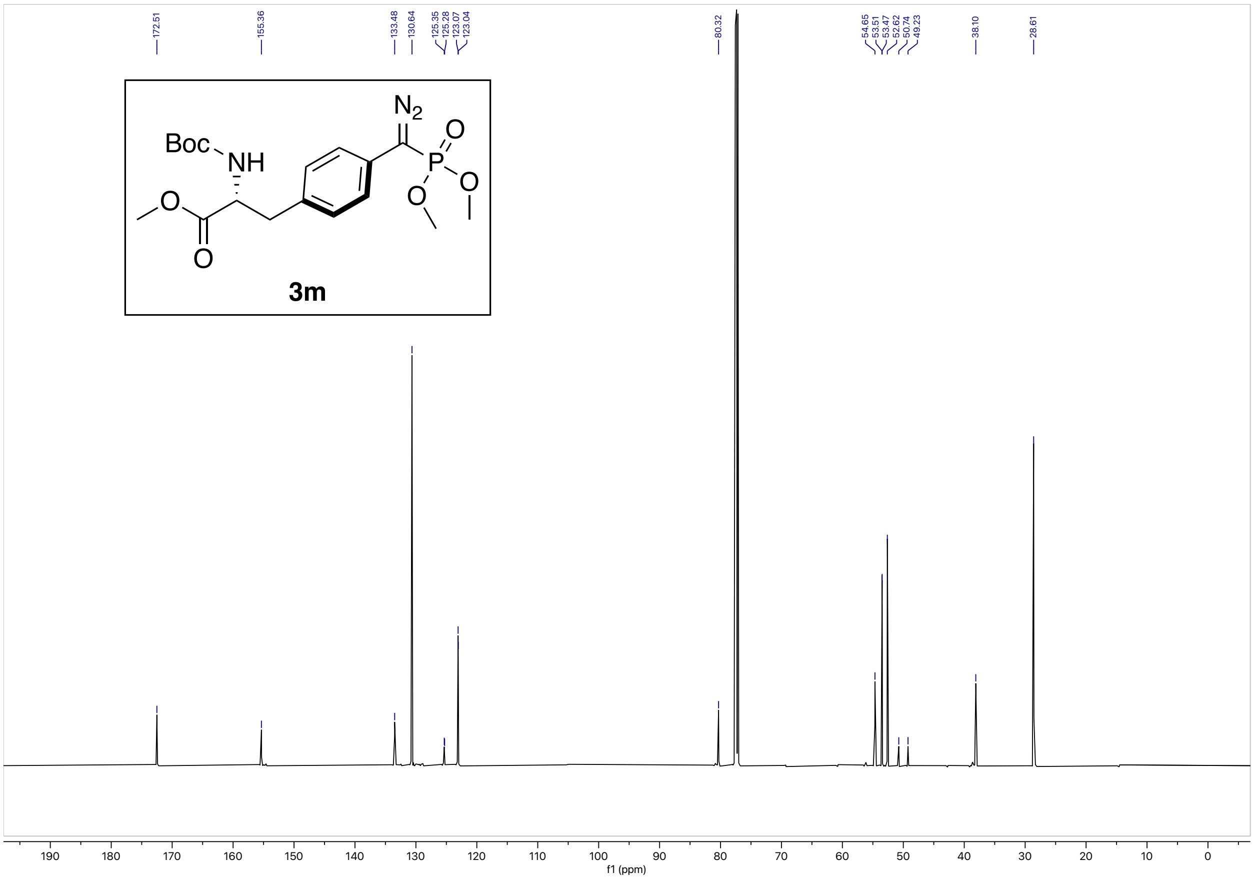

$^{13}C\{^{1}H\}$ NMR spectrum (151 MHz, Chloroform-*d*) (t, 77.36 ppm) of **3m.**

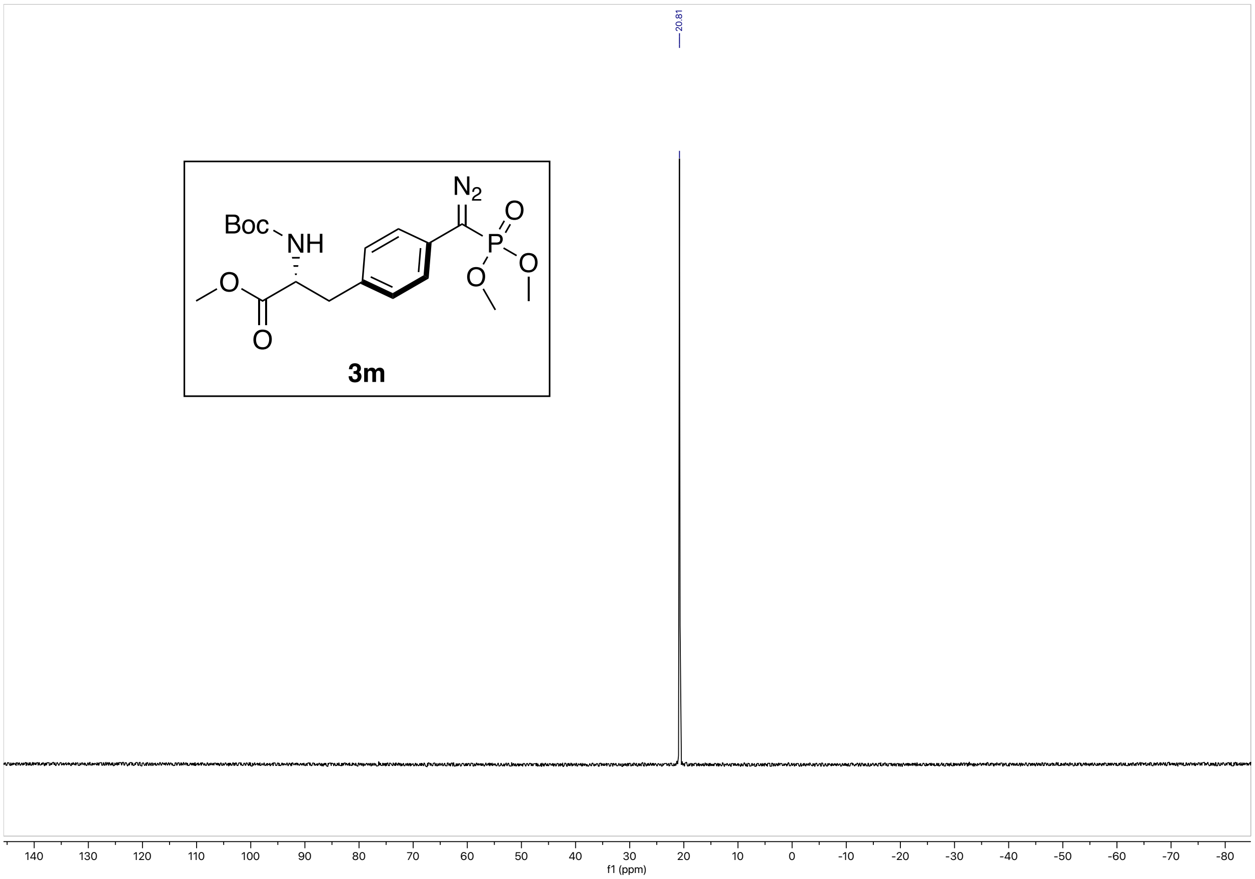

^{31}P NMR spectrum (162 MHz, Chloroform-d) of **3m.**

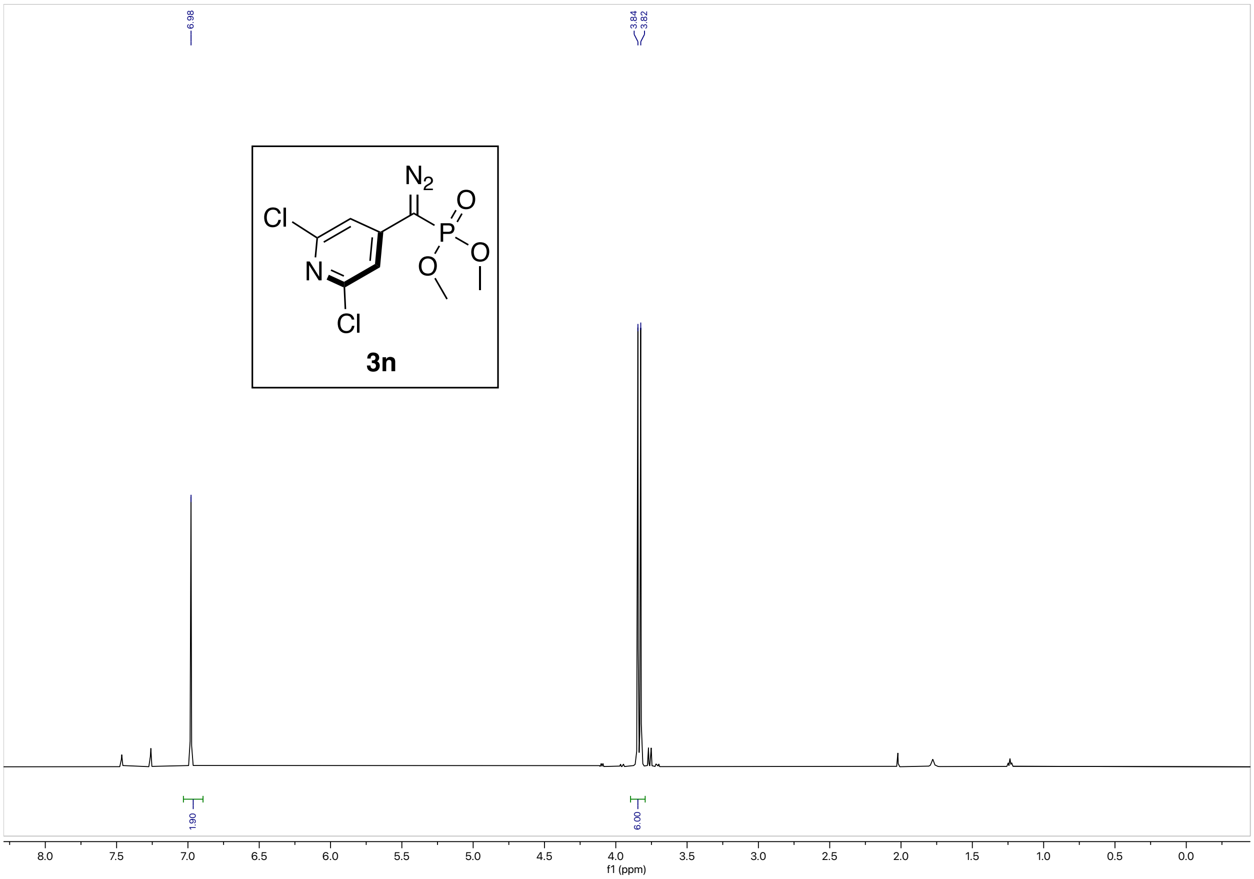

^{1}H NMR spectrum (600 MHz, Chloroform-*d*) (s, 7.26 ppm) of **3n.**

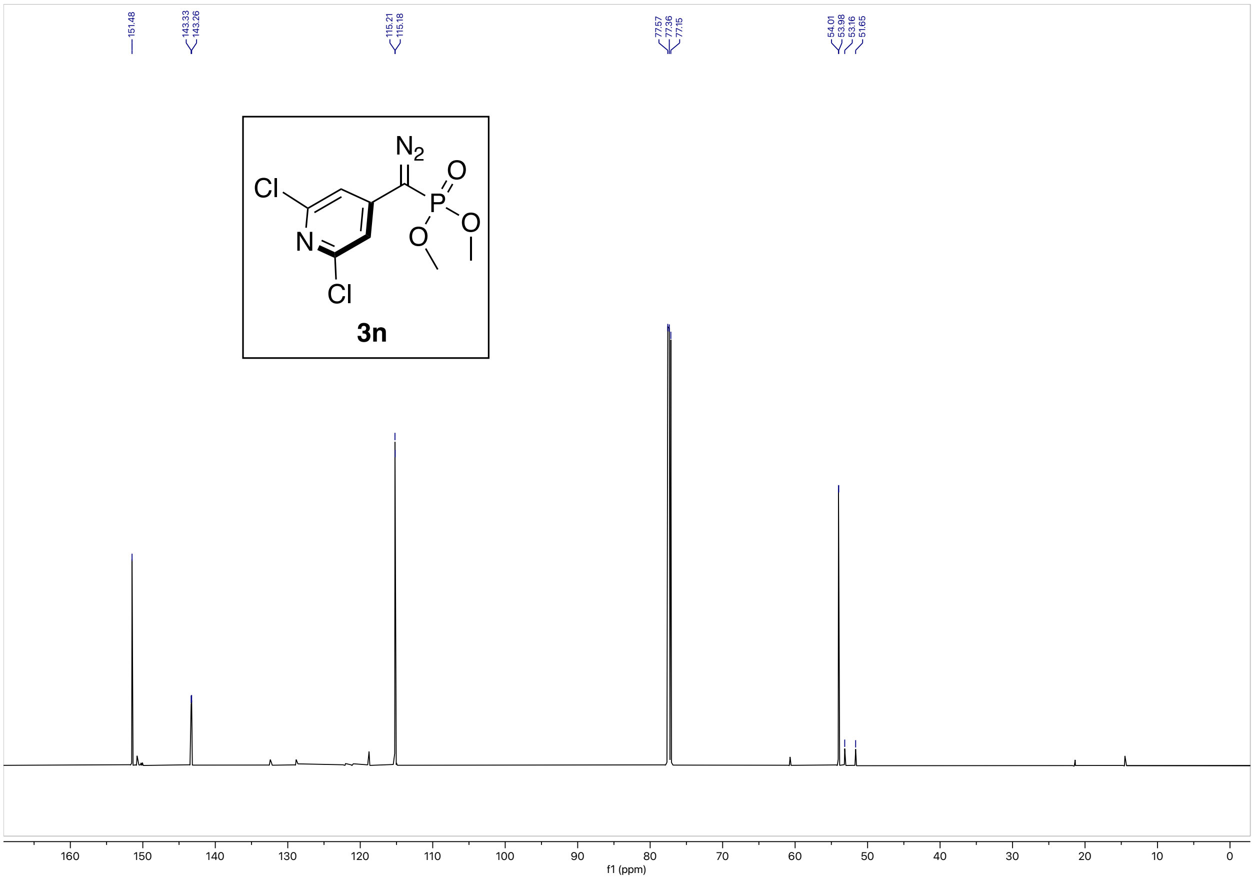

$^{13}C\{^{1}H\}$ NMR spectrum (151 MHz, Chloroform-*d*) (t, 77.36 ppm) of **3n.**

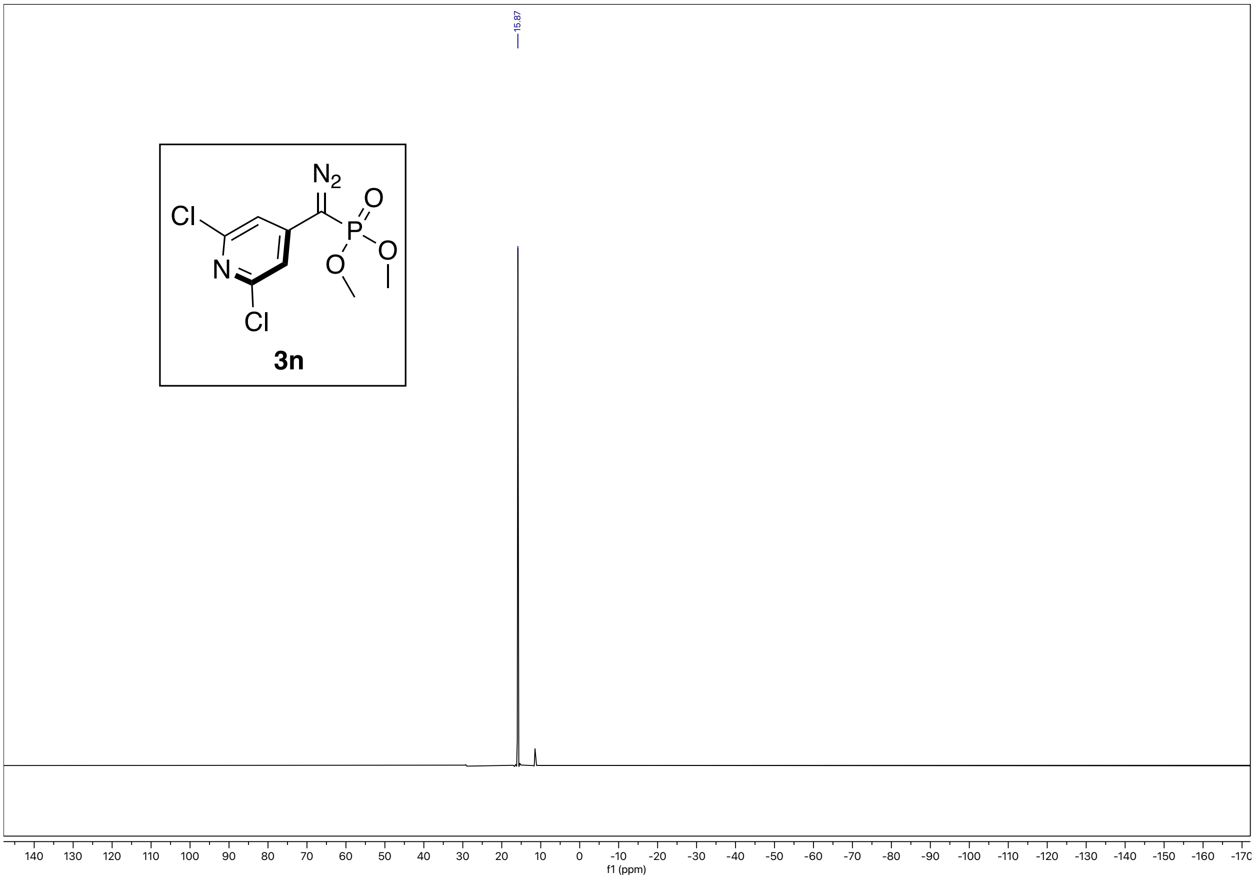

^{31}P NMR spectrum (243 MHz, Chloroform-d) of **3n.**

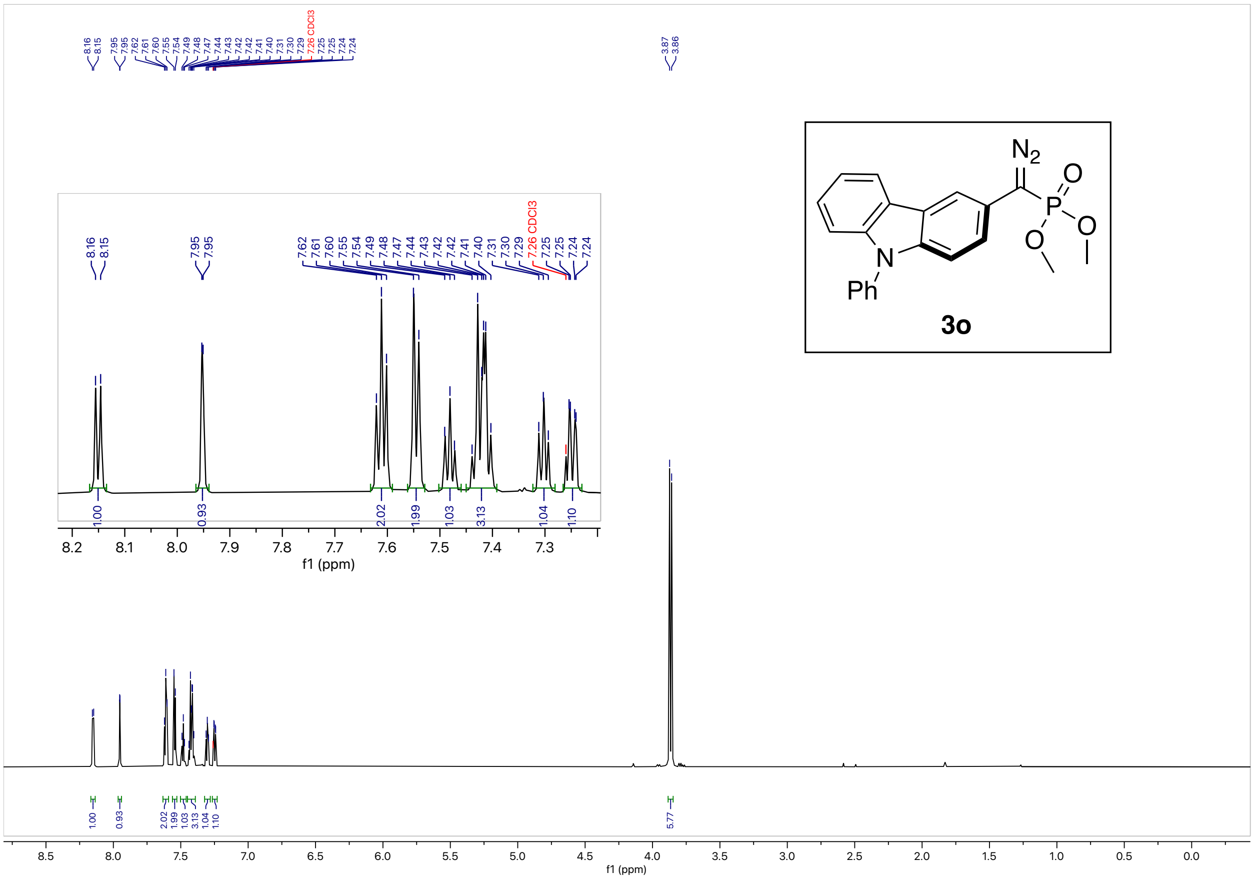

^{1}H NMR spectrum (800 MHz, Chloroform-*d*) (s, 7.26 ppm) of **3o.**

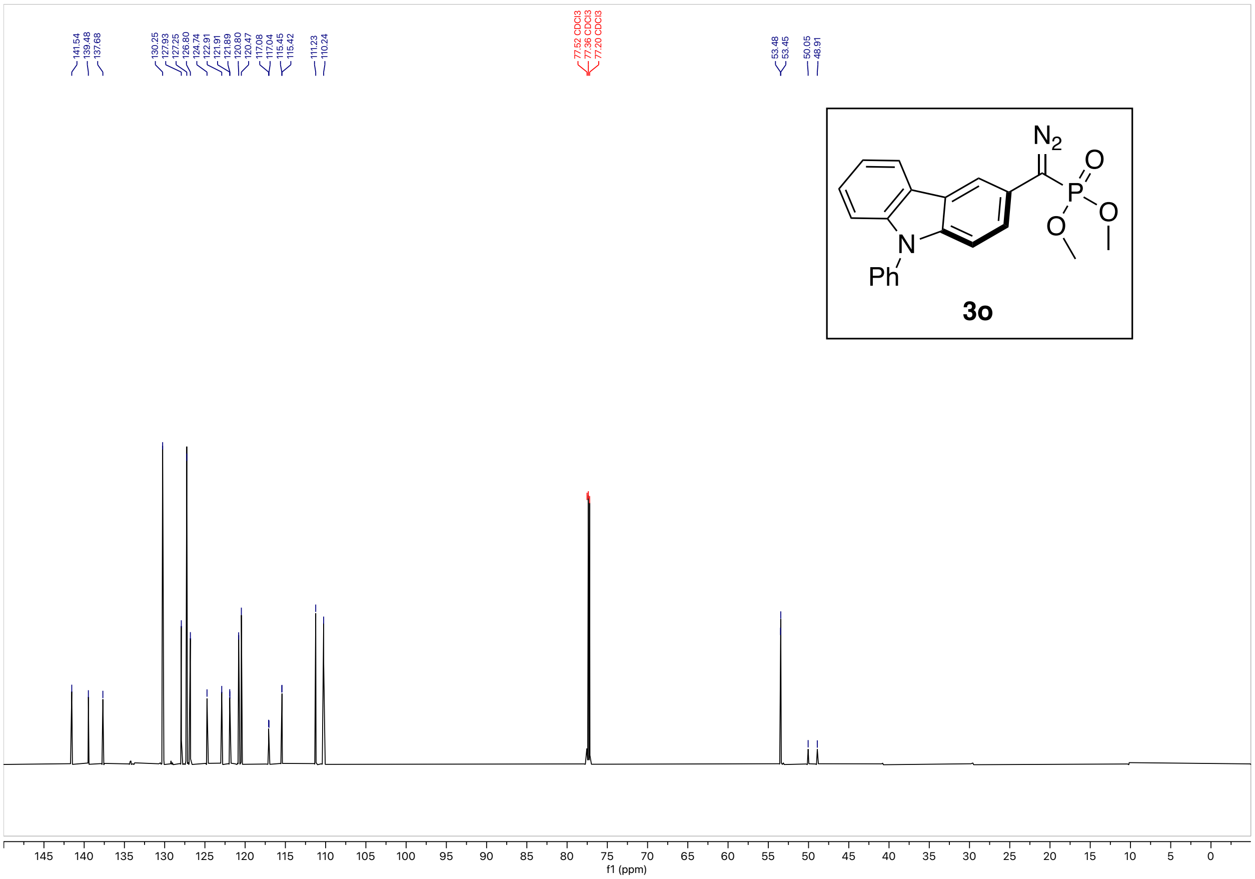

$^{13}C\{^{1}H\}$ NMR spectrum (201 MHz, Chloroform-*d*) (t, 77.36 ppm) of **3o.**

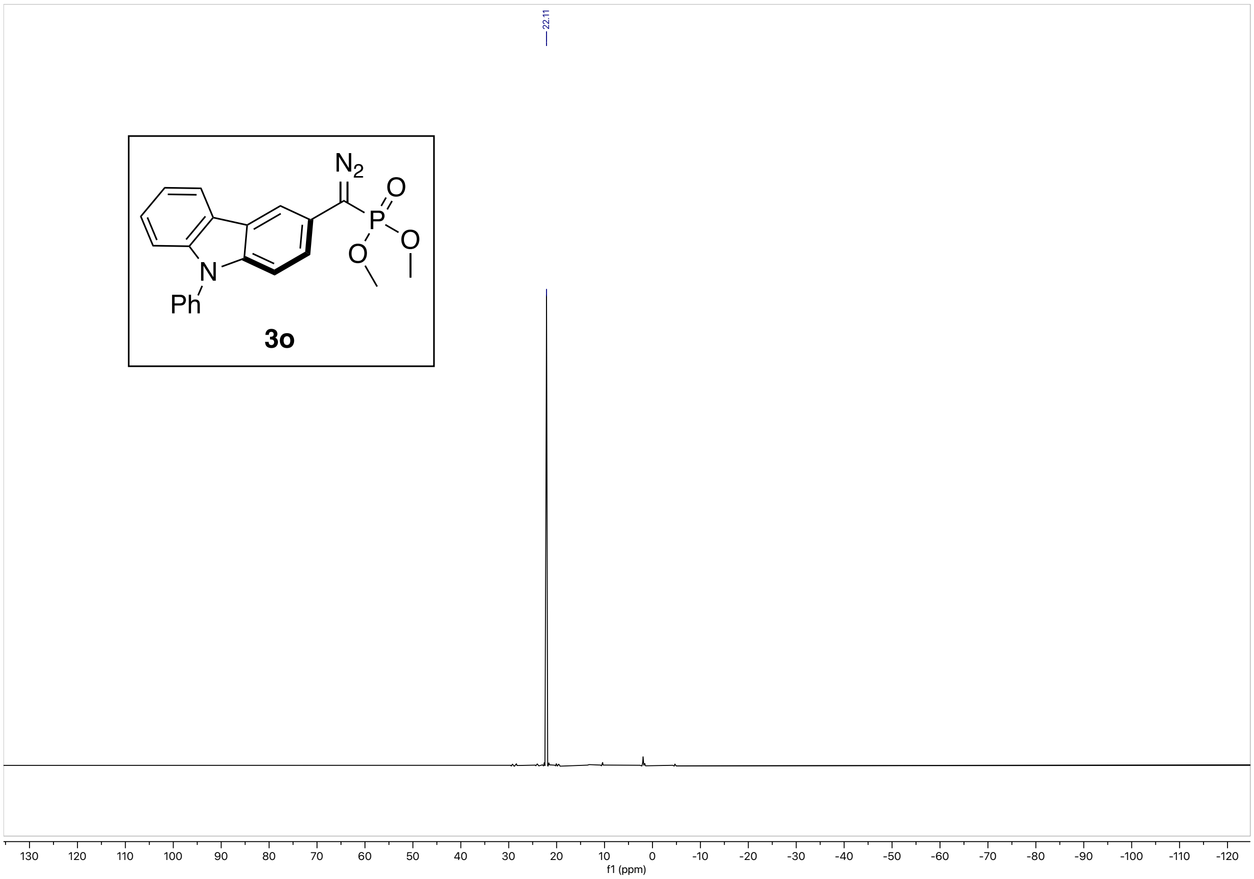

^{31}P NMR spectrum (243 MHz, Chloroform-d) of **3o.**

7. Cyclopropanation ^{1}H NMR, ^{13}C{^{1}H} NMR, ^{19}F NMR, and ^{31}P NMR Spectroscopic Data.

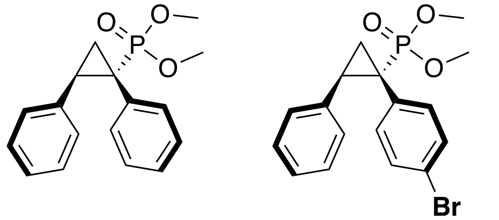

6b **6c**

The NMR spectra for these compounds have been reported in previous publications.[1, 2]

(1) Davies, H. M. L.; Lee, G. H. Enantioselective Synthesis of Cyclopropylphosphonates Containing Quaternary Stereocenters Using a D_2-Symmetric Chiral Catalyst Rh_2(*S*-biTISP)$_2$. *Org. Lett.* **2004**, 6, 2117-2120.

(2) Reddy, R. P.; Lee, G. H.; Davies, H. M. L. Dirhodium Tetracarboxylate Derived from Adamantylglycine as a Chiral Catalyst for Carbenoid Reactions. *Org. Lett.* **2006**, 8, 3437-3440.

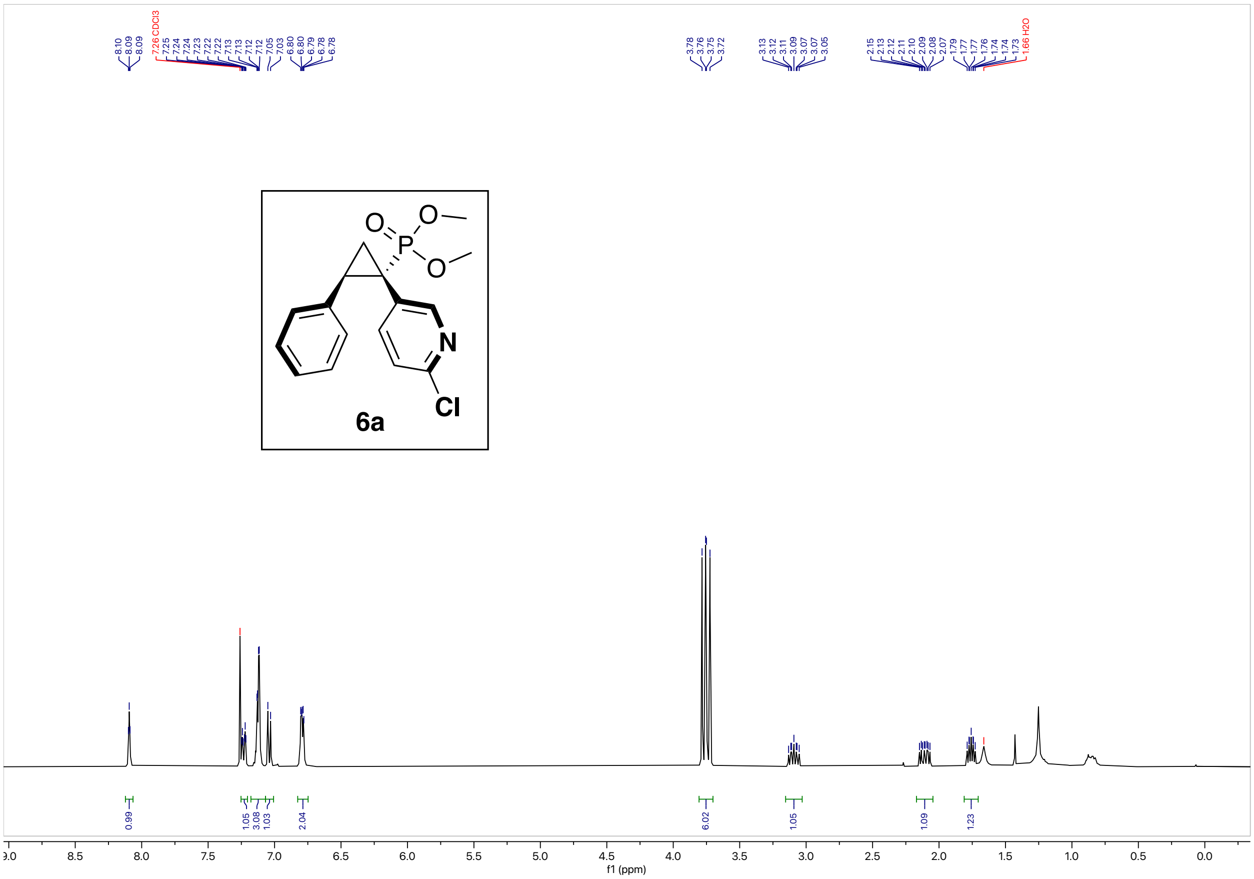

^{1}H NMR spectrum (600 MHz, Chloroform-*d*) (s, 7.26 ppm) of **6a.**

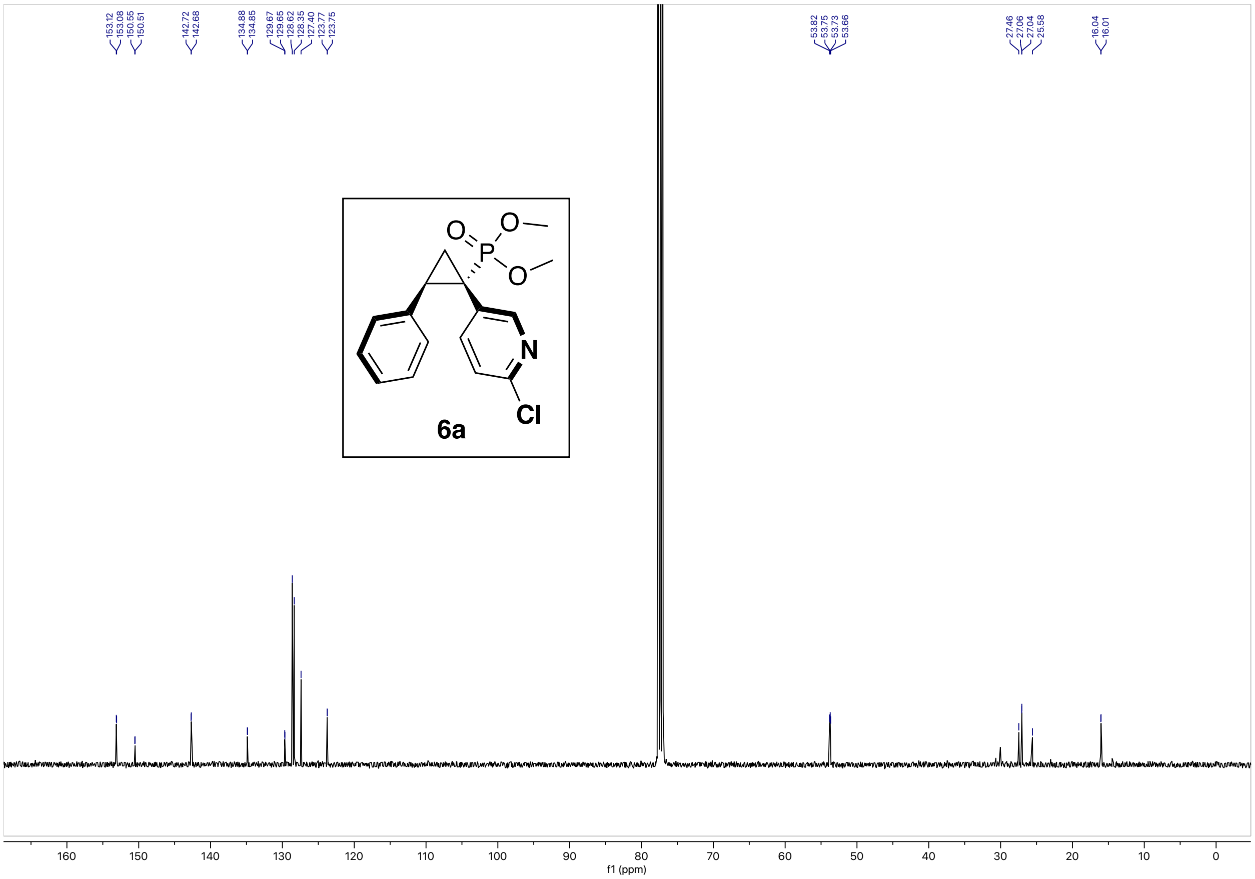

$^{13}C\{^{1}H\}$ NMR spectrum (151 MHz, Chloroform-*d*) (t, 77.36 ppm) of **6a.**

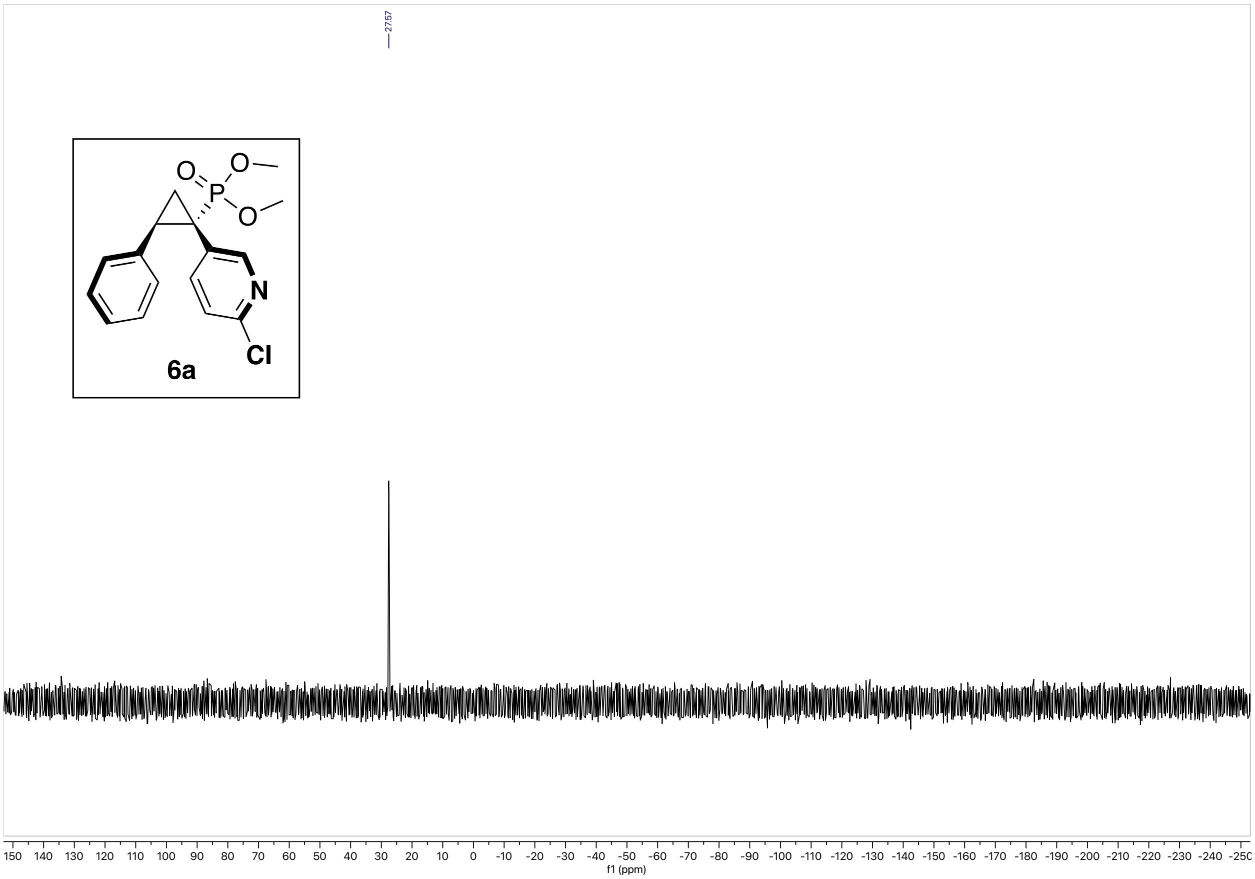

^{31}P NMR spectrum (243 MHz, Chloroform-d) of **6a.**

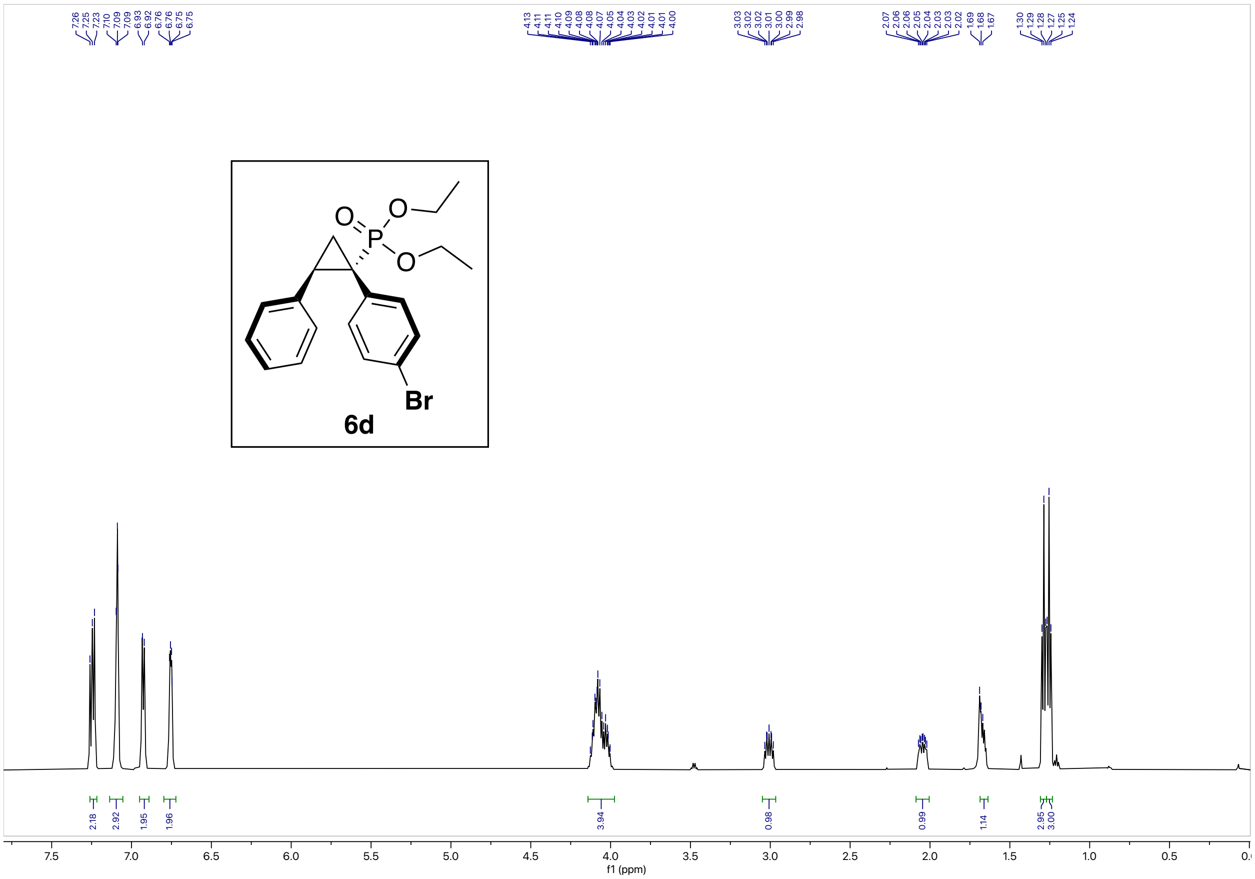

^{1}H NMR spectrum (600 MHz, Chloroform-*d*) (s, 7.26 ppm) of **6d.**

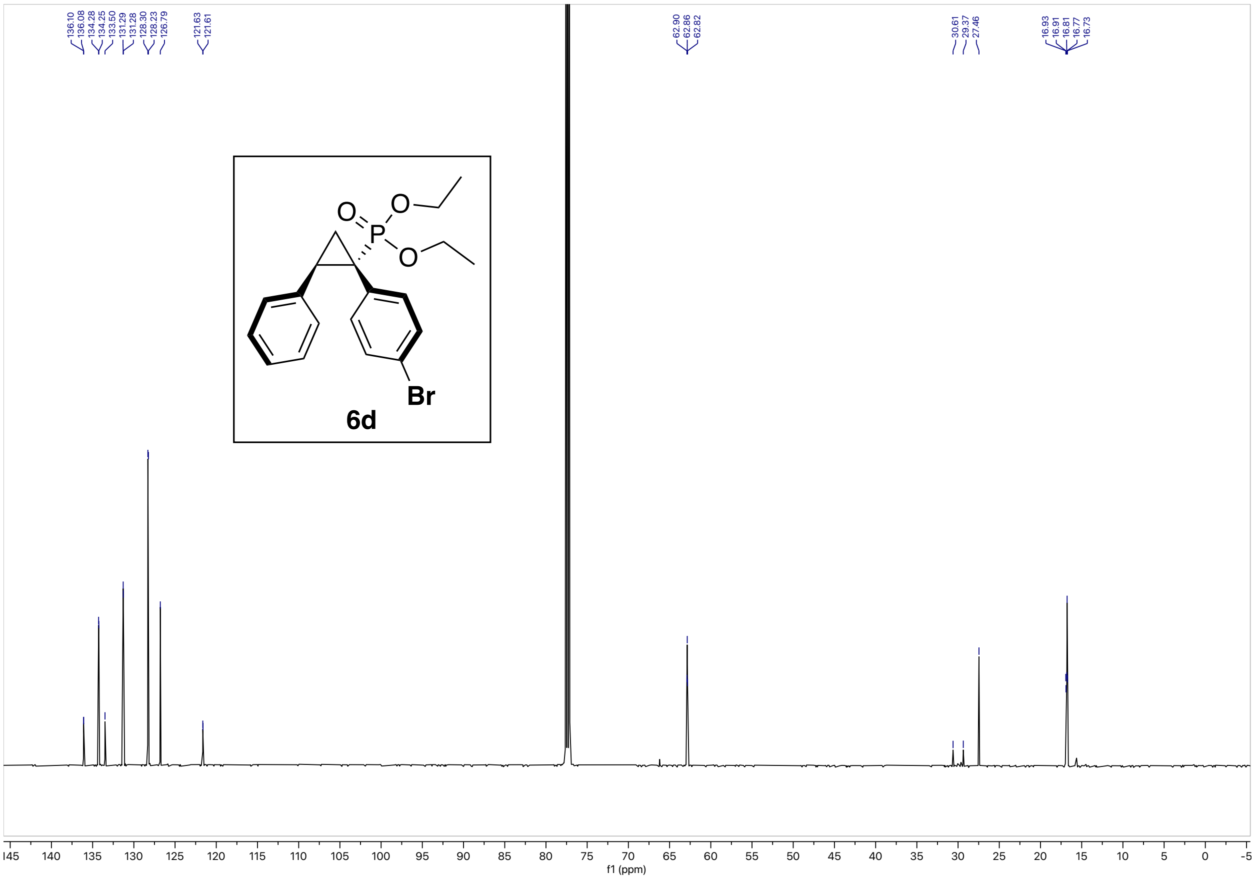

$^{13}C\{^{1}H\}$ NMR spectrum (151 MHz, Chloroform-*d*) (t, 77.36 ppm) of **6d.**

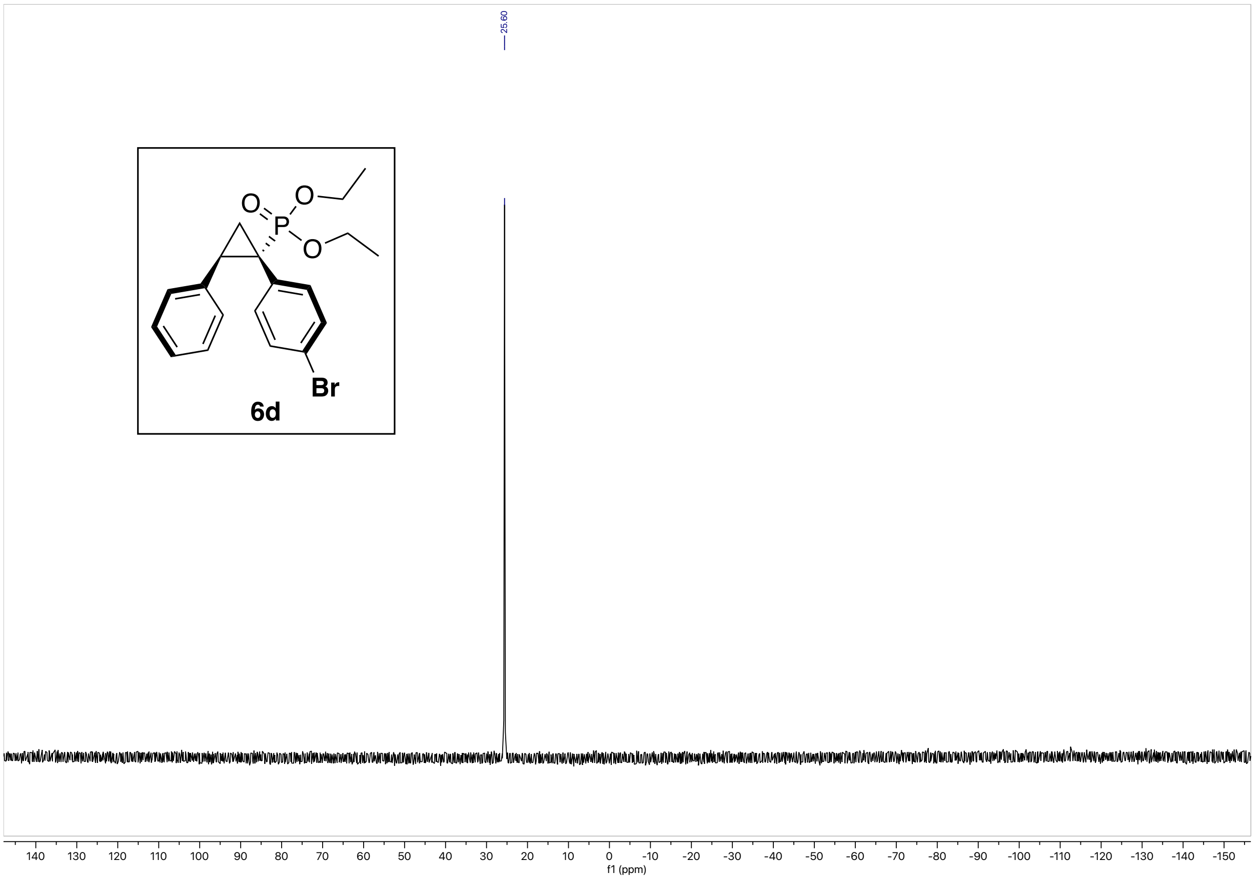

^{31}P NMR spectrum (243 MHz, Chloroform-d) of **6d.**

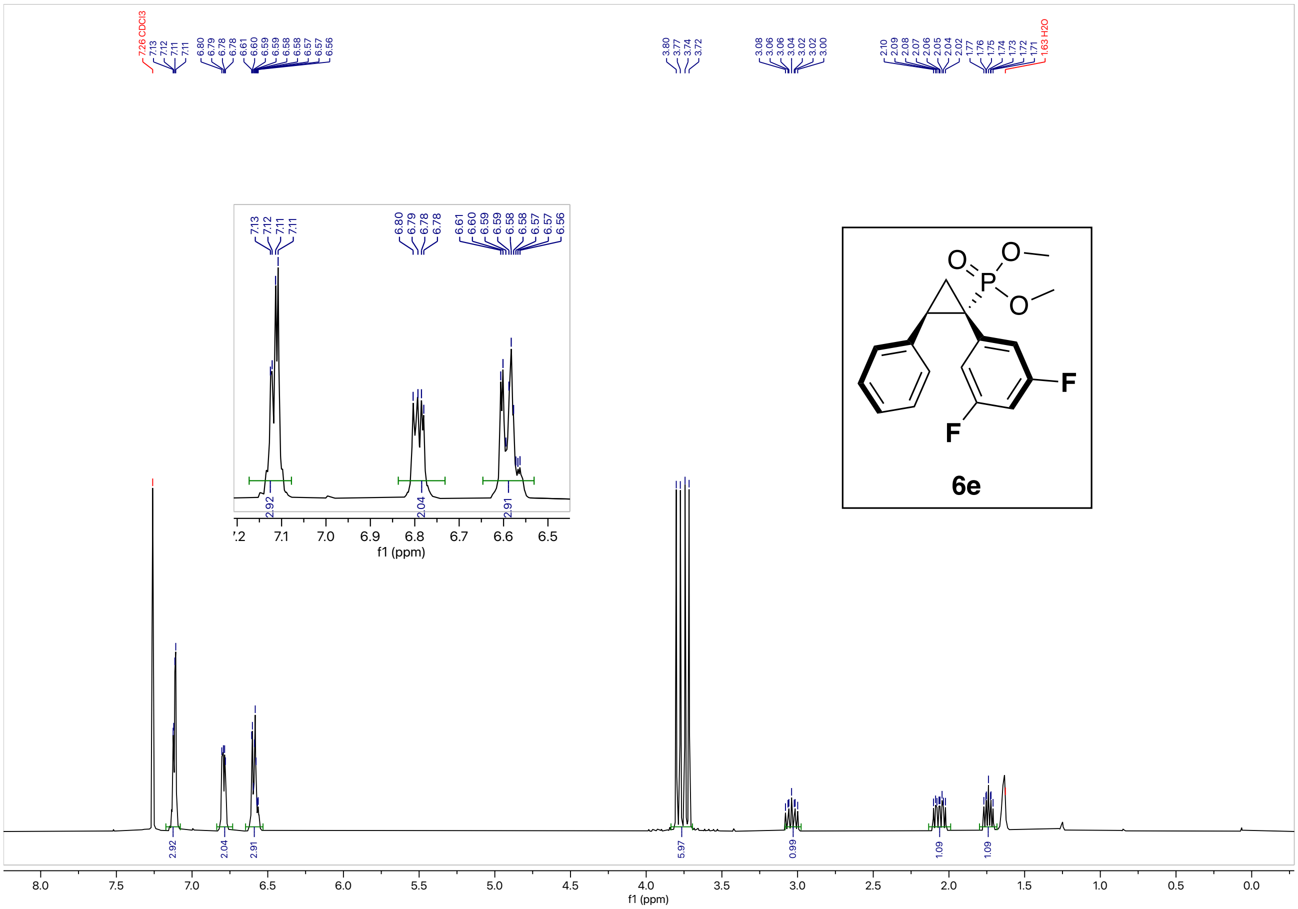

^{1}H NMR spectrum (400 MHz, Chloroform-*d*) (s, 7.26 ppm) of **6e.**

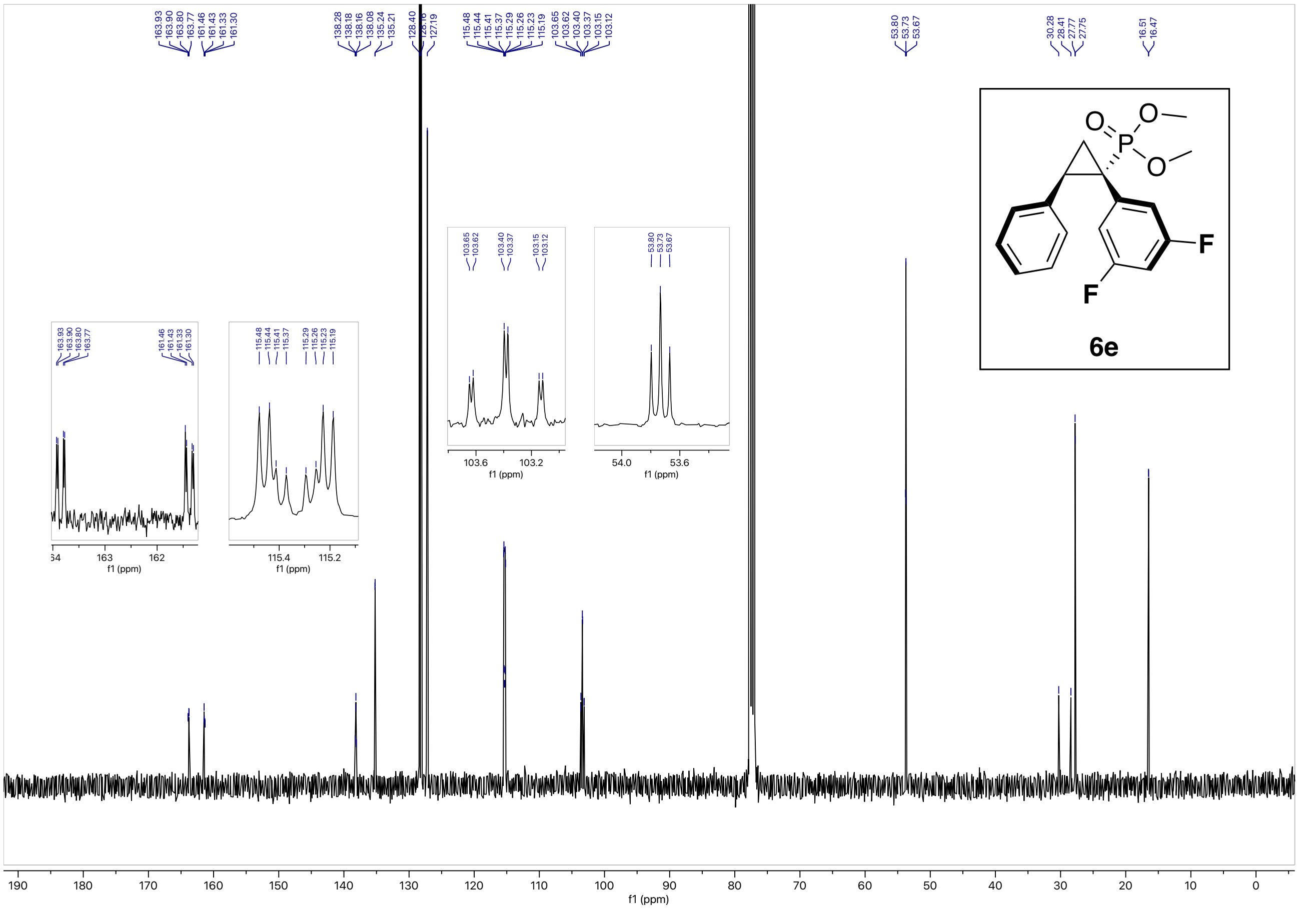

$^{13}C\{^1H\}$ NMR spectrum (101 MHz, Chloroform-*d*) (t, 77.36 ppm) of **6e.**

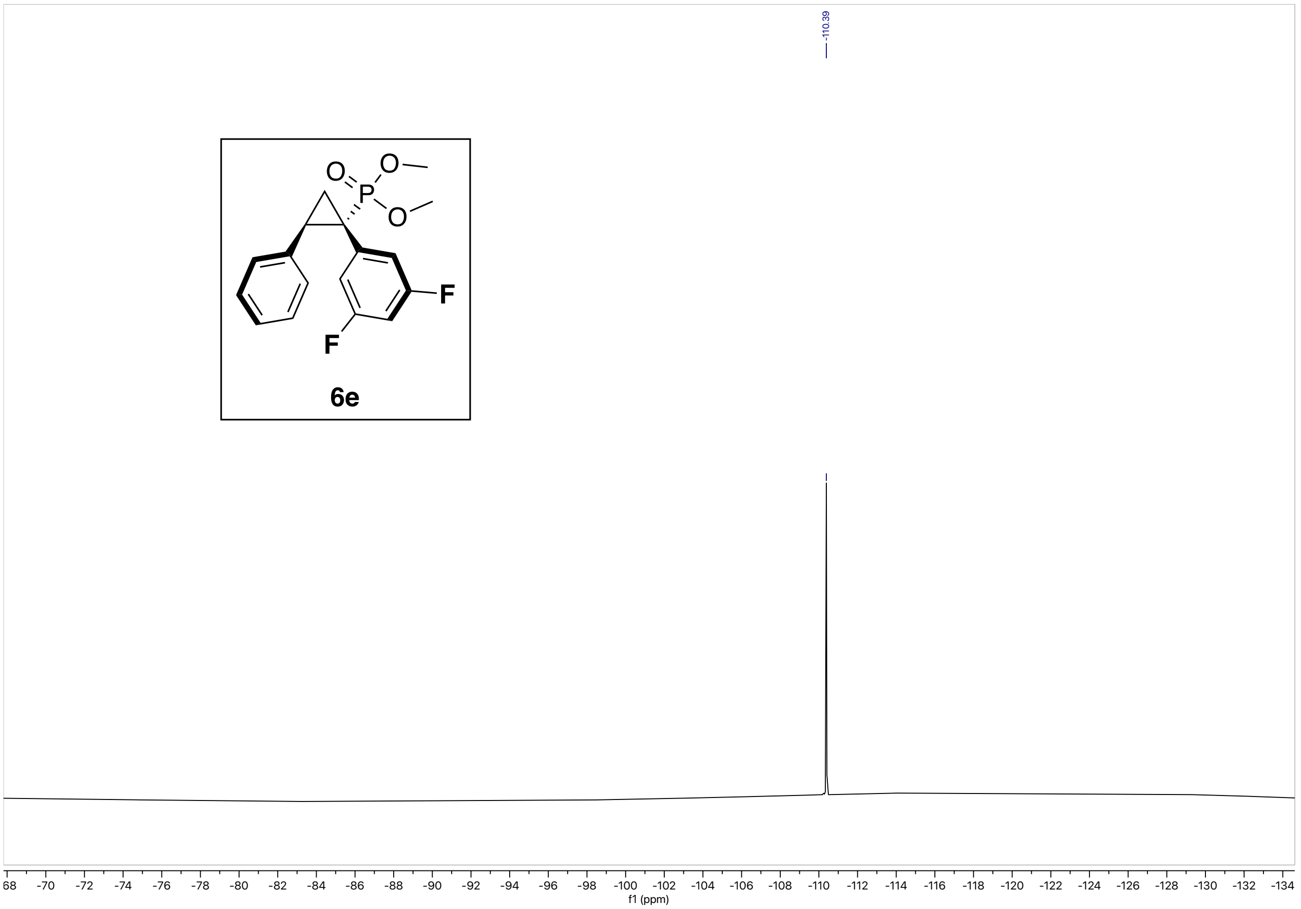

^{19}F NMR spectrum (565 MHz, Chloroform-d) of **6e.**

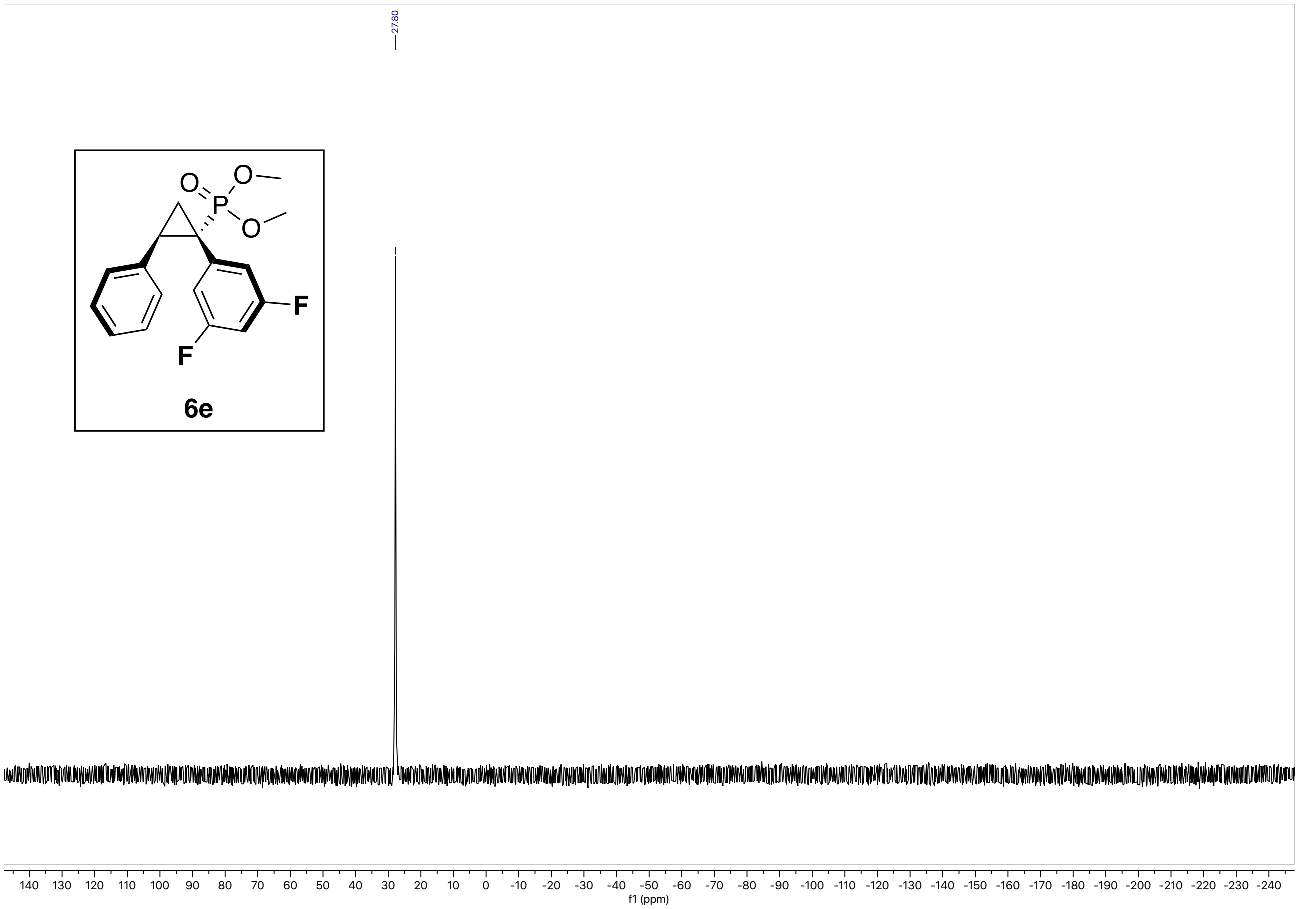

^{31}P NMR spectrum (243 MHz, Chloroform-d) of **6e.**

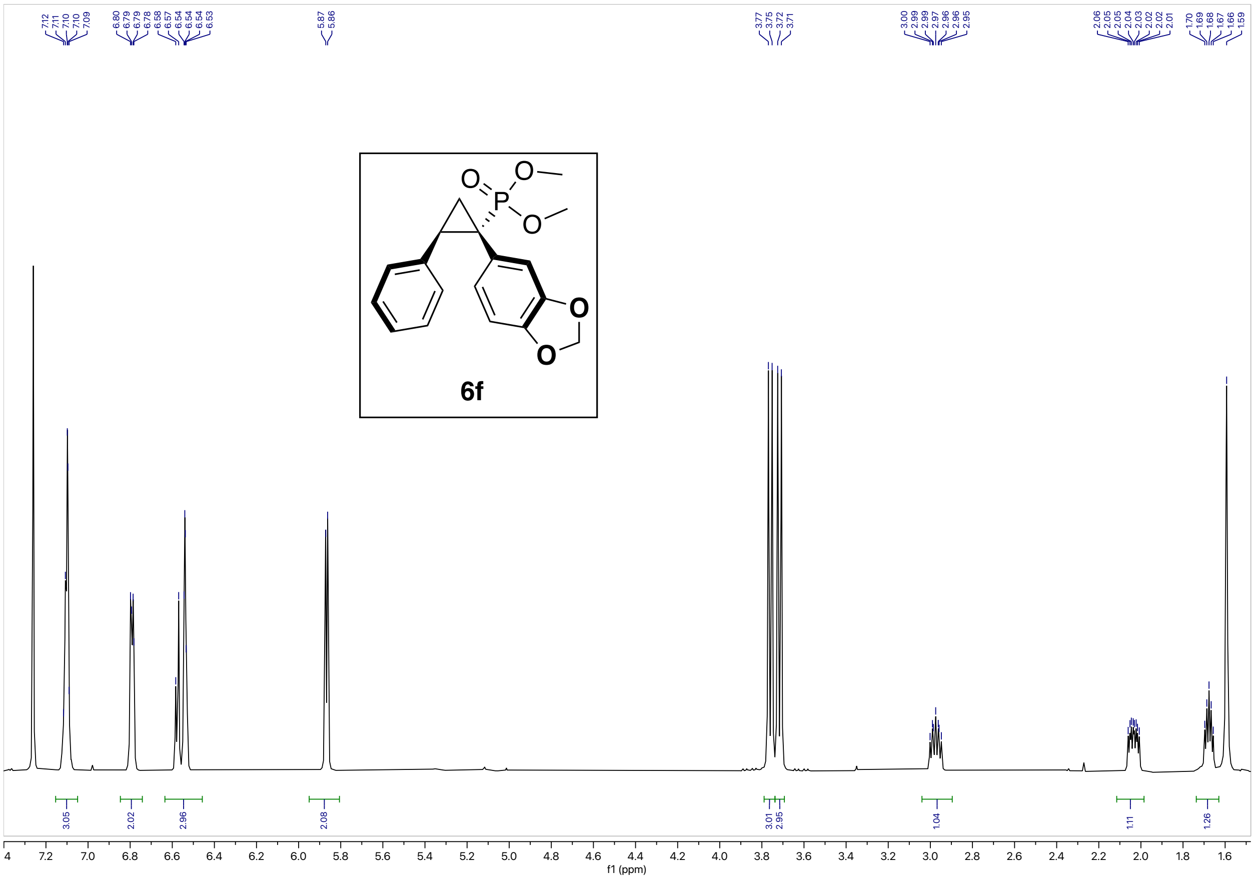

^{1}H NMR spectrum (600 MHz, Chloroform-*d*) (s, 7.26 ppm) of **6f.**

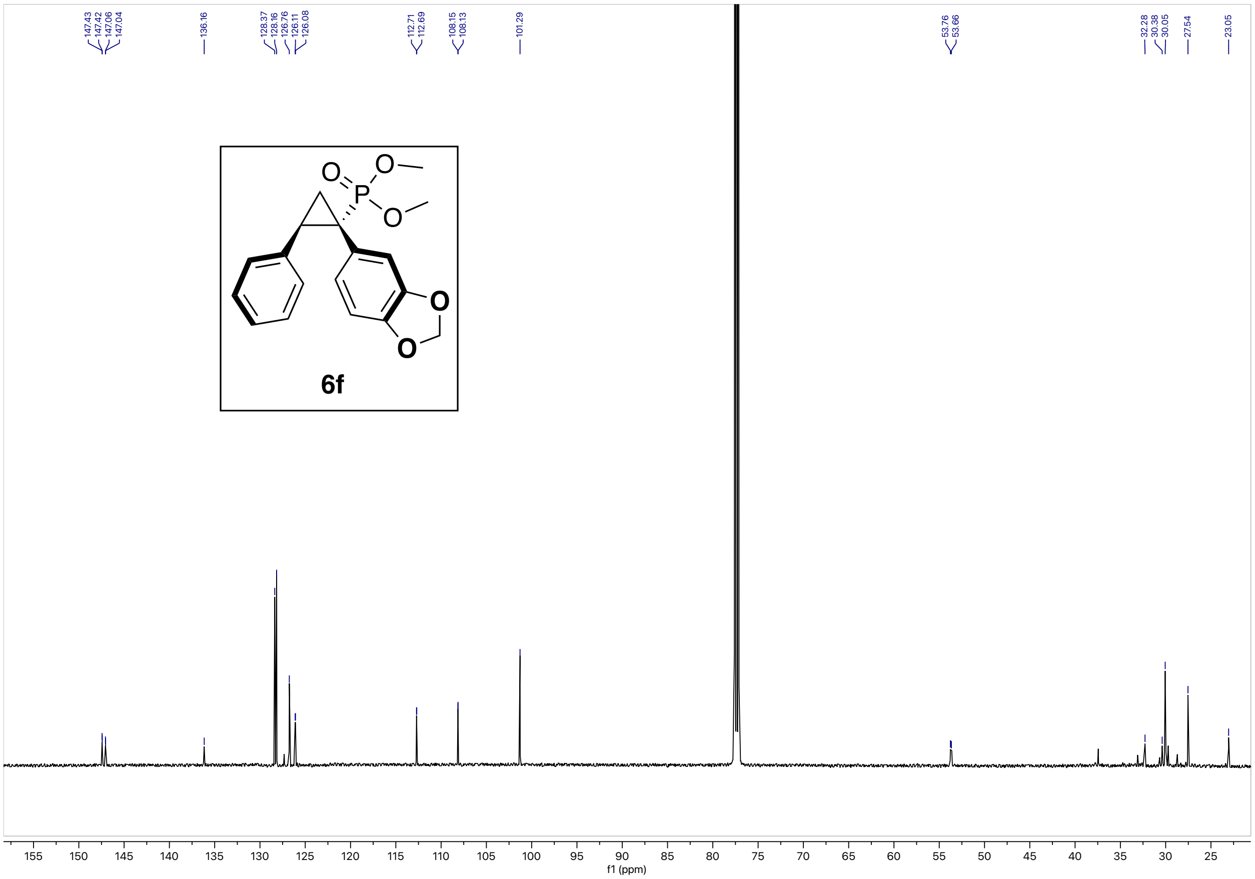

$^{13}C\{^{1}H\}$ NMR spectrum (151 MHz, Chloroform-*d*) (t, 77.36 ppm) of **6f.**

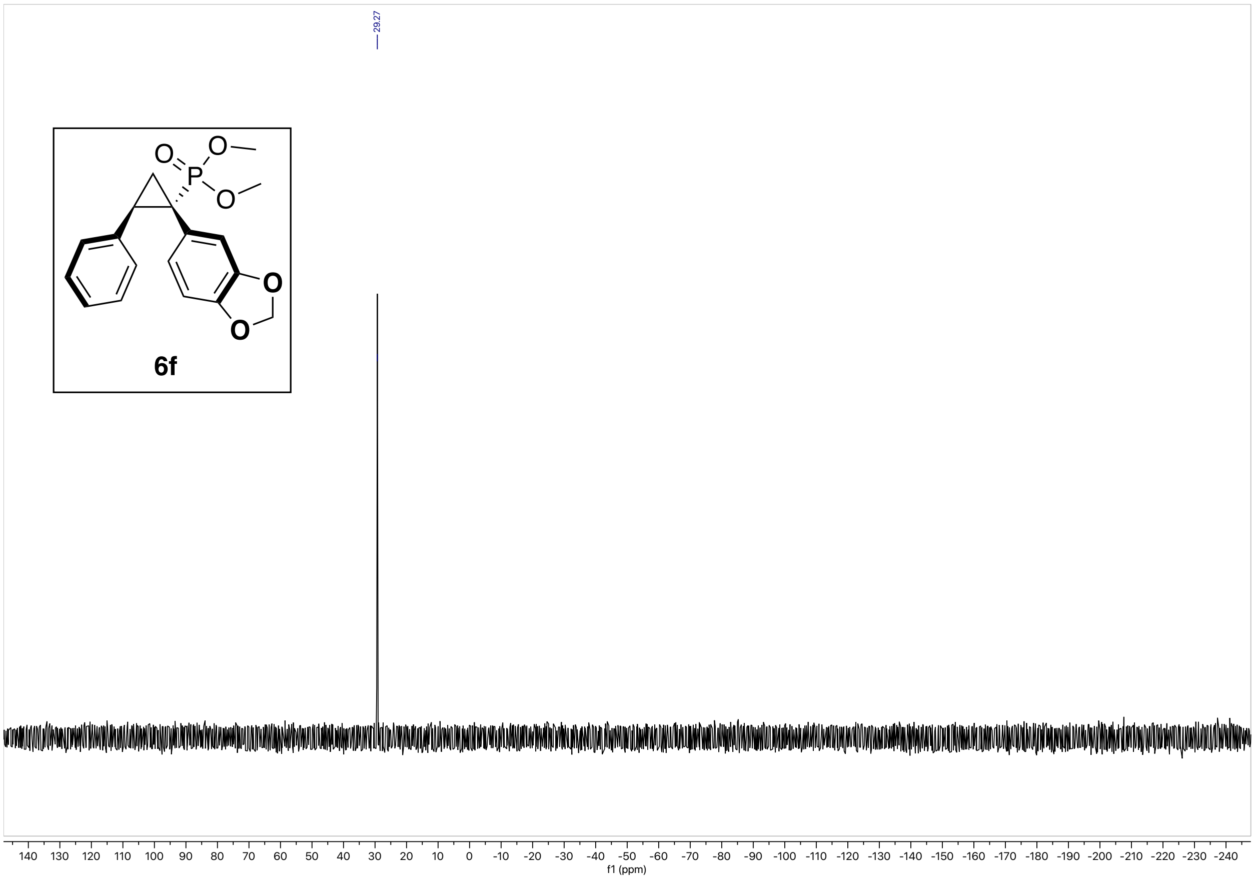

^{31}P NMR spectrum (243 MHz, Chloroform-d) of **6f.**

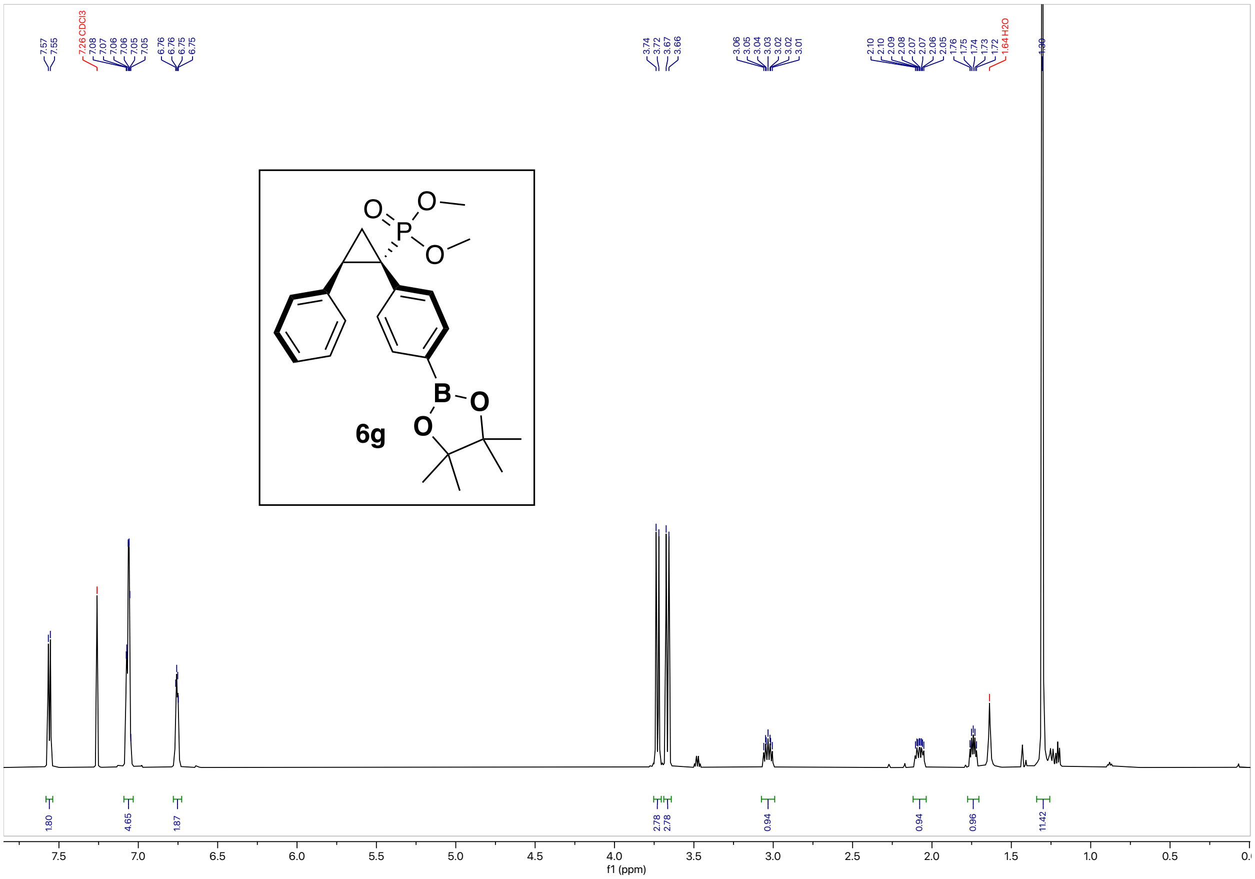

^{1}H NMR spectrum (600 MHz, Chloroform-*d*) (s, 7.26 ppm) of **6g.**

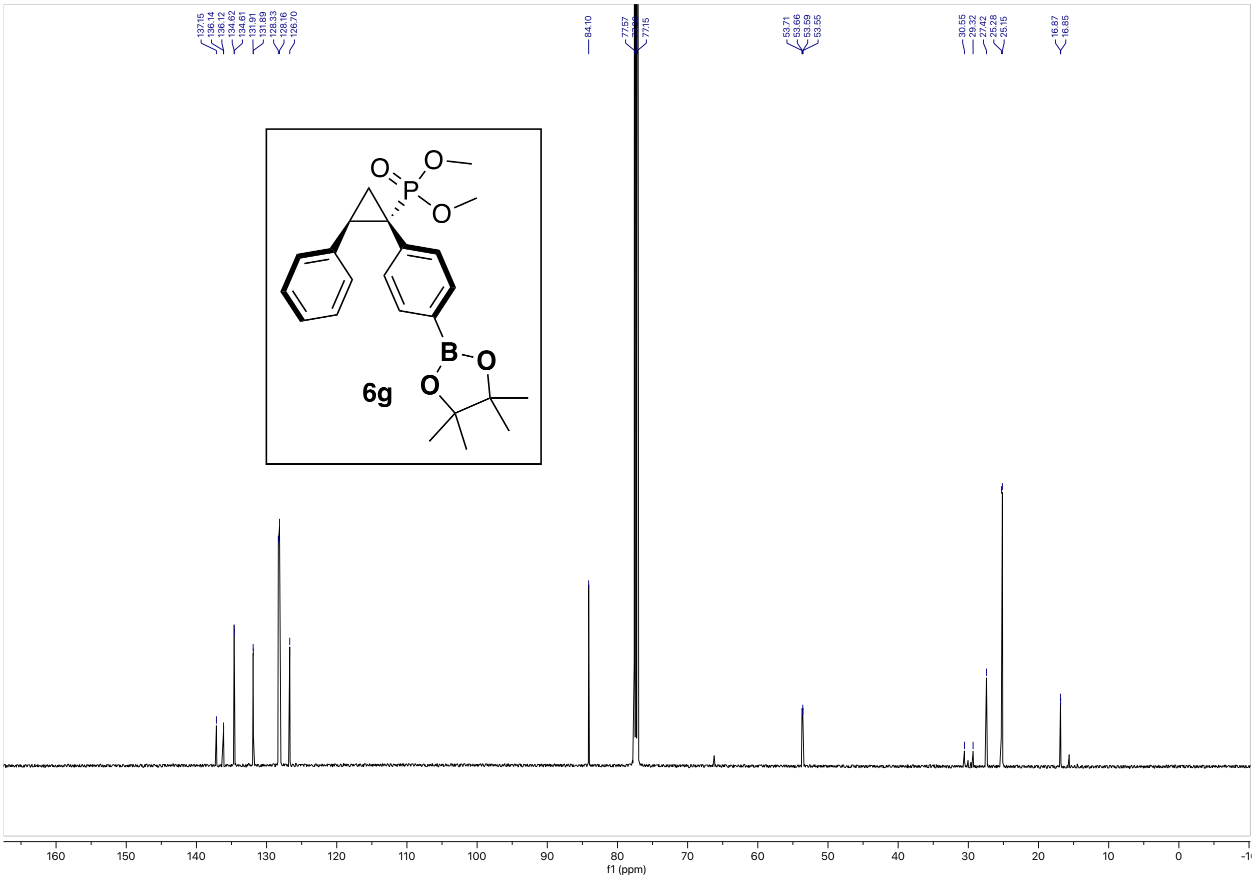

$^{13}C\{^{1}H\}$ NMR spectrum (151 MHz, Chloroform-*d*) (t, 77.36 ppm) of **6g.**

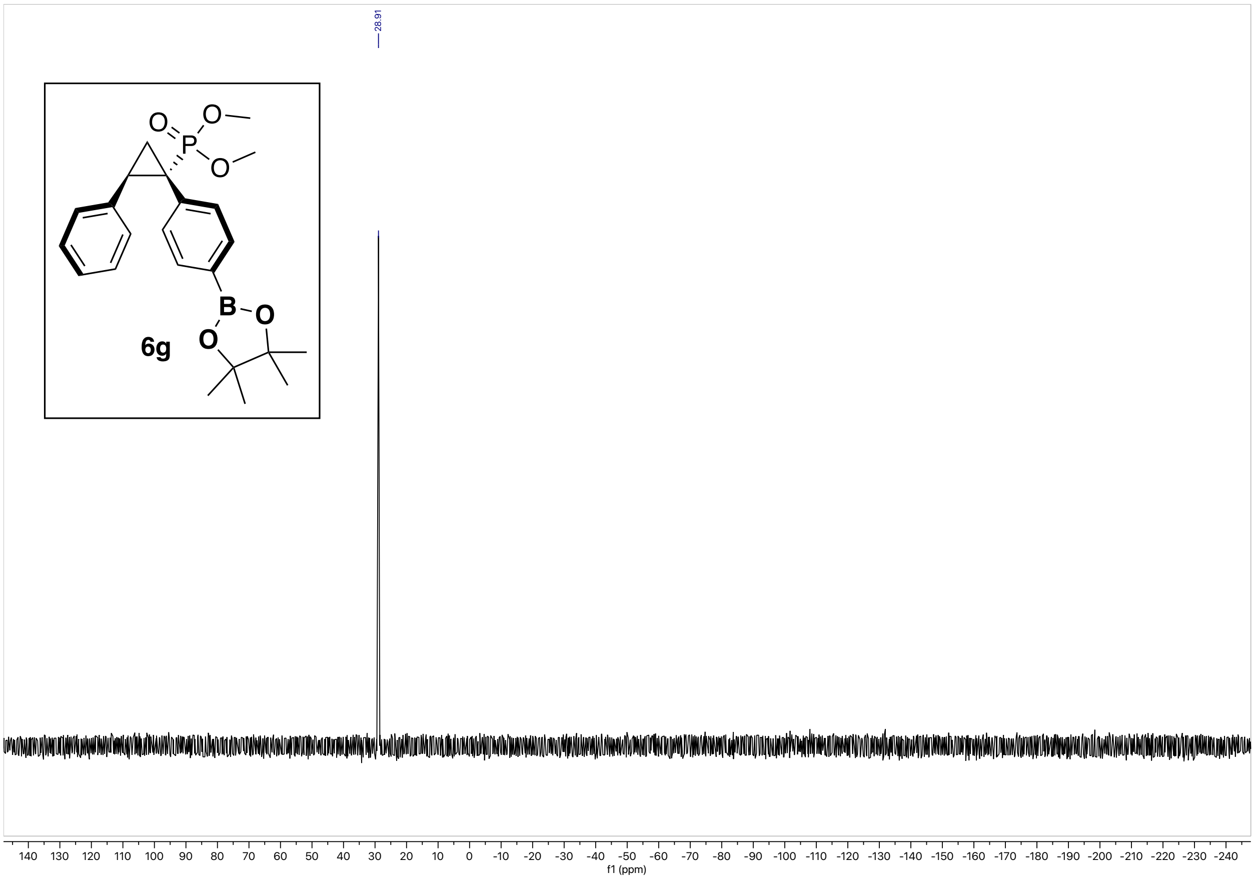

^{31}P NMR spectrum (243 MHz, Chloroform-d) of **6g.**

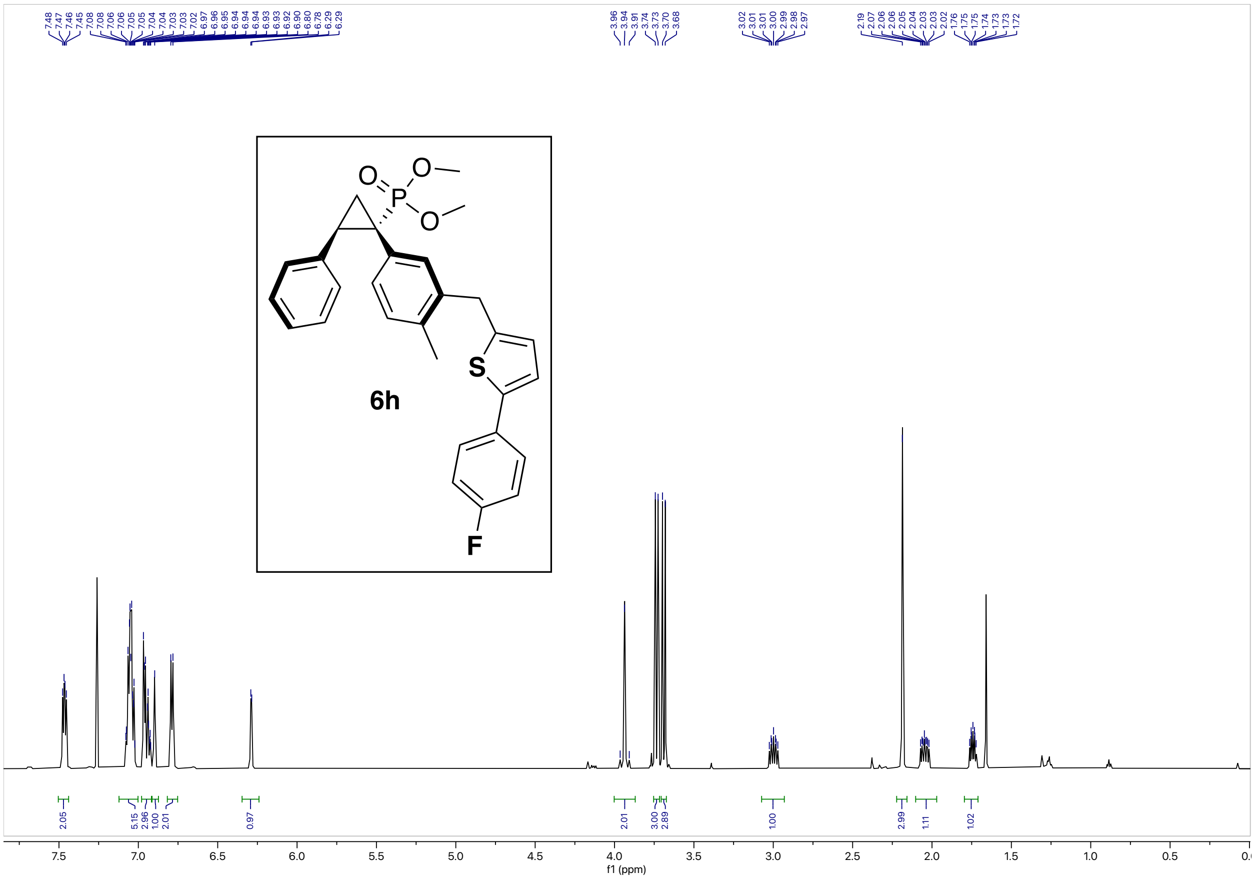

^{1}H NMR spectrum (600 MHz, Chloroform-*d*) (s, 7.26 ppm) of **6h.**

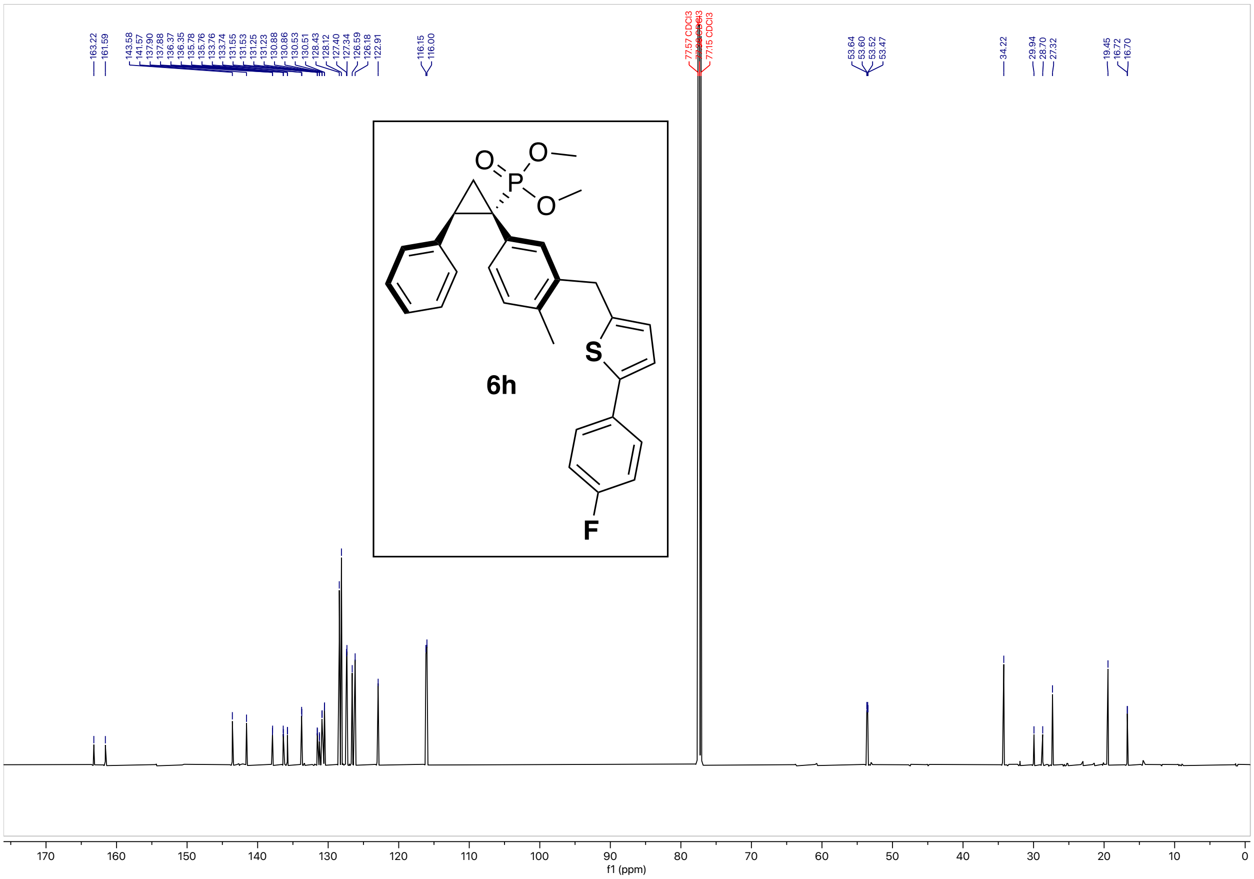

$^{13}C\{^{1}H\}$ NMR spectrum (151 MHz, Chloroform-*d*) (t, 77.36 ppm) of **6h.**

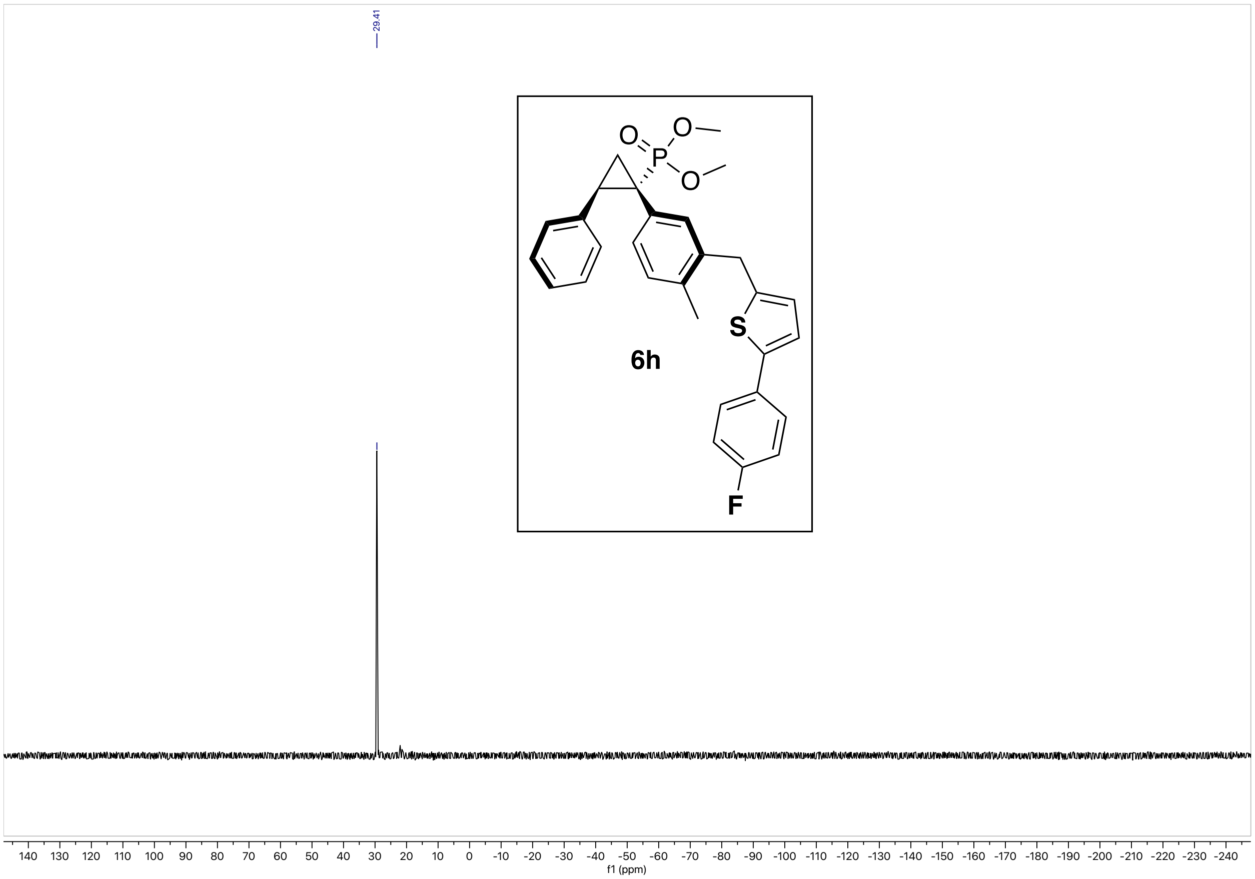

^{31}P NMR spectrum (243 MHz, Chloroform-d) of **6h.**

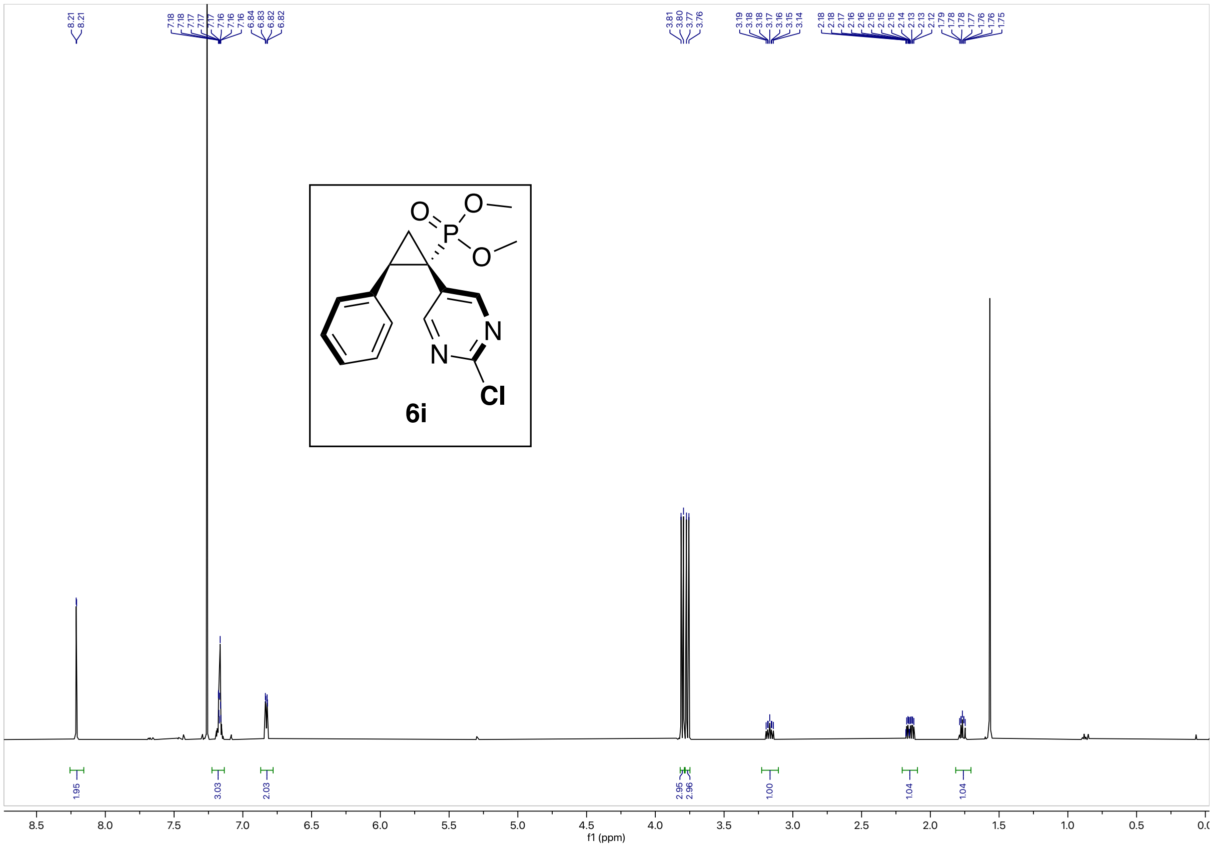

^{1}H NMR spectrum (600 MHz, Chloroform-*d*) (s, 7.26 ppm) of **6i.**

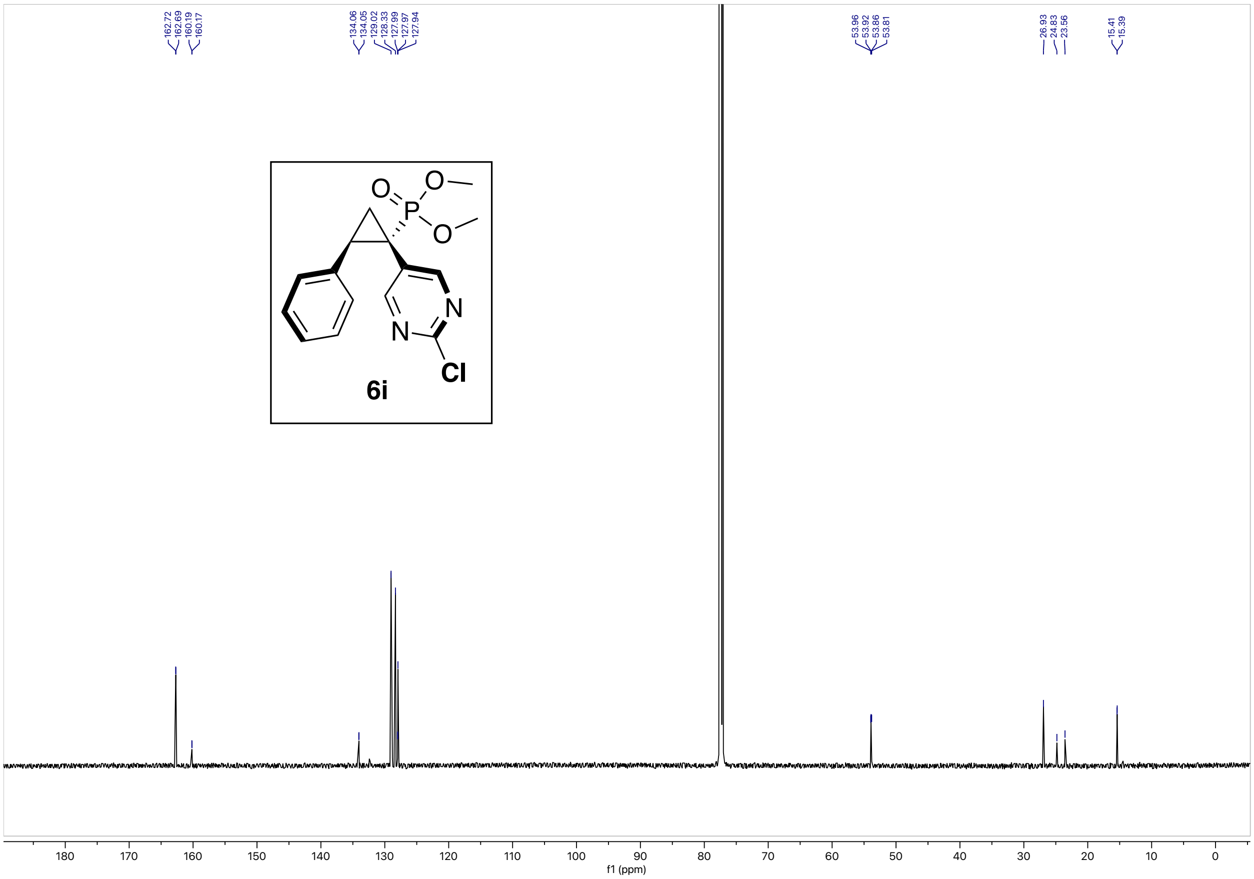

$^{13}C\{^1H\}$ NMR spectrum (151 MHz, Chloroform-*d*) (t, 77.36 ppm) of **6i.**

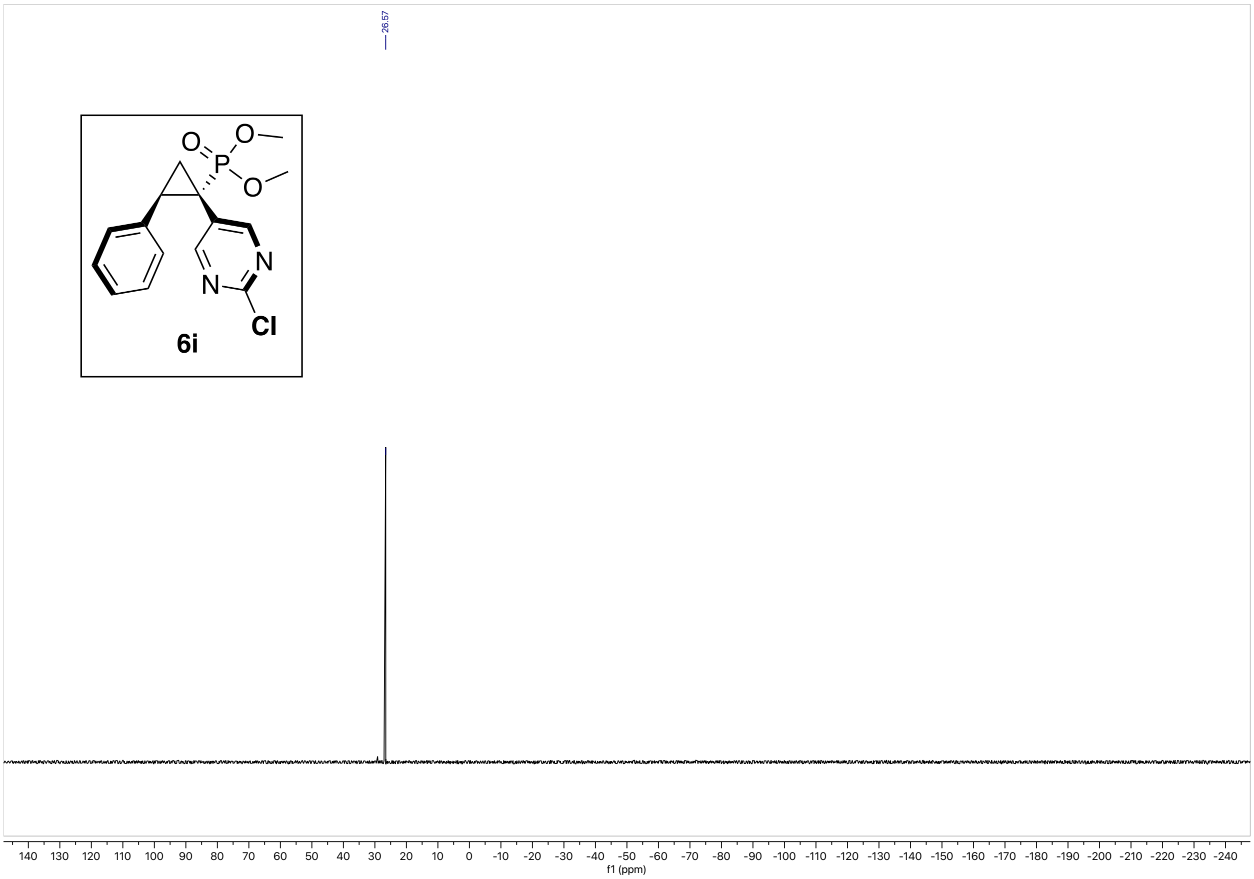

^{31}P NMR spectrum (243 MHz, Chloroform-d) of **6i.**

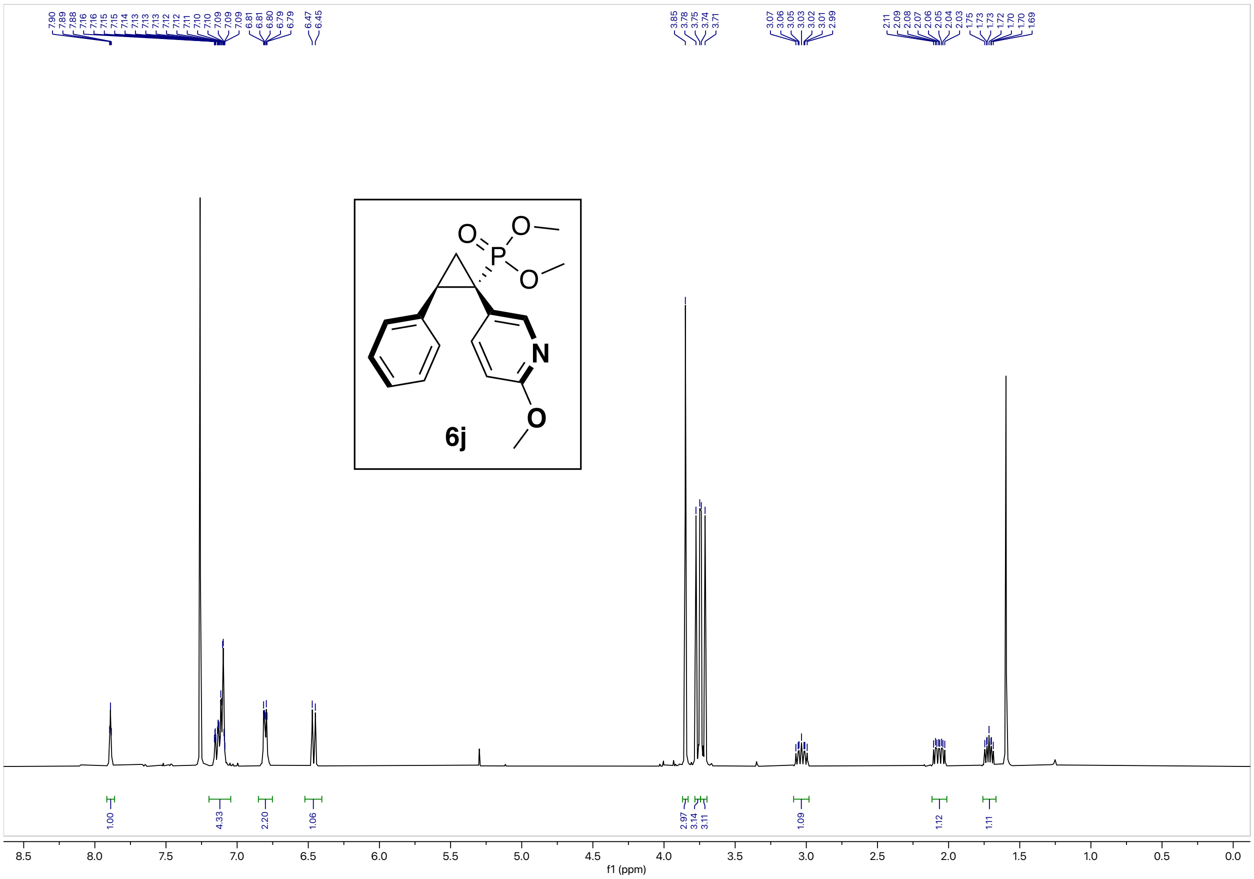

^{1}H NMR spectrum (400 MHz, Chloroform-*d*) (s, 7.26 ppm) of **6j.**

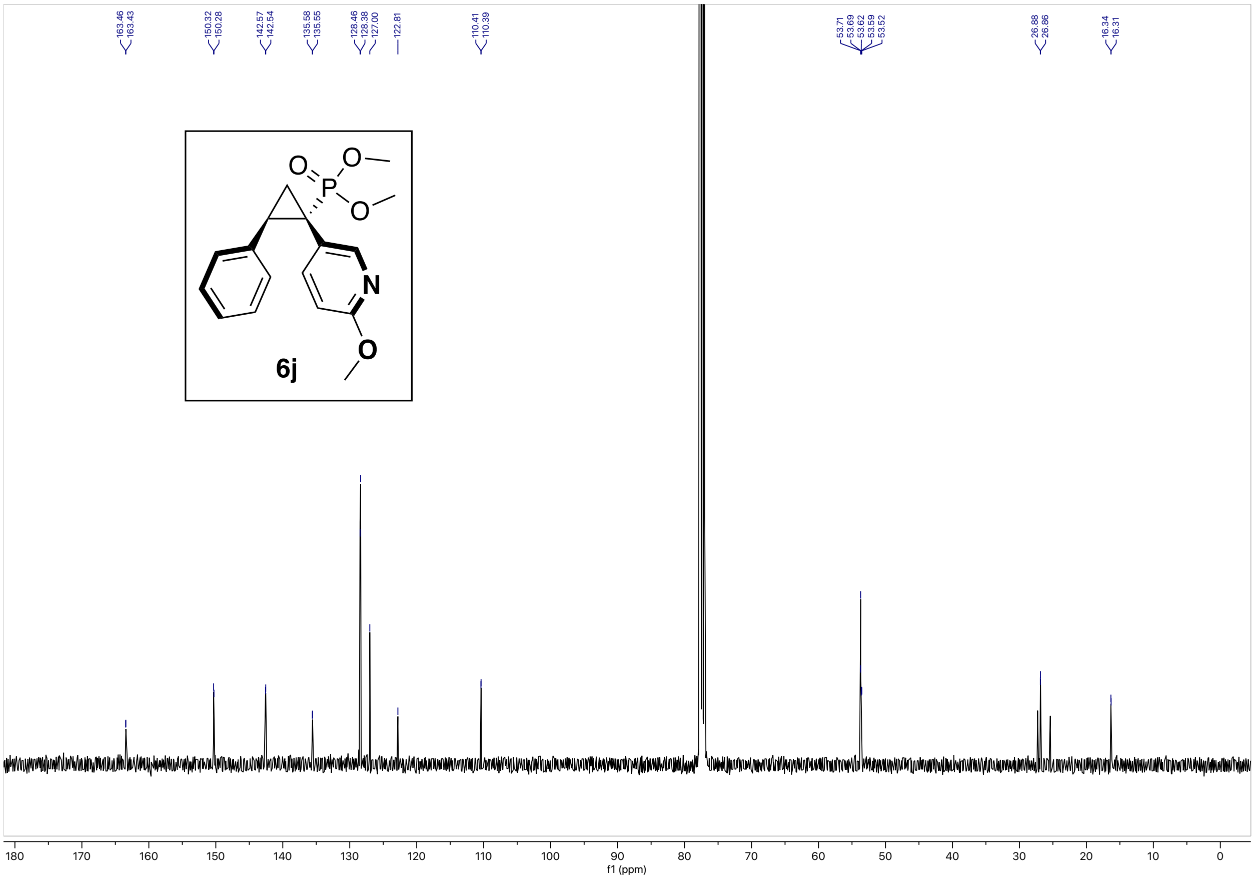

$^{13}C\{^{1}H\}$ NMR spectrum (101 MHz, Chloroform-*d*) (t, 77.36 ppm) of **6j.**

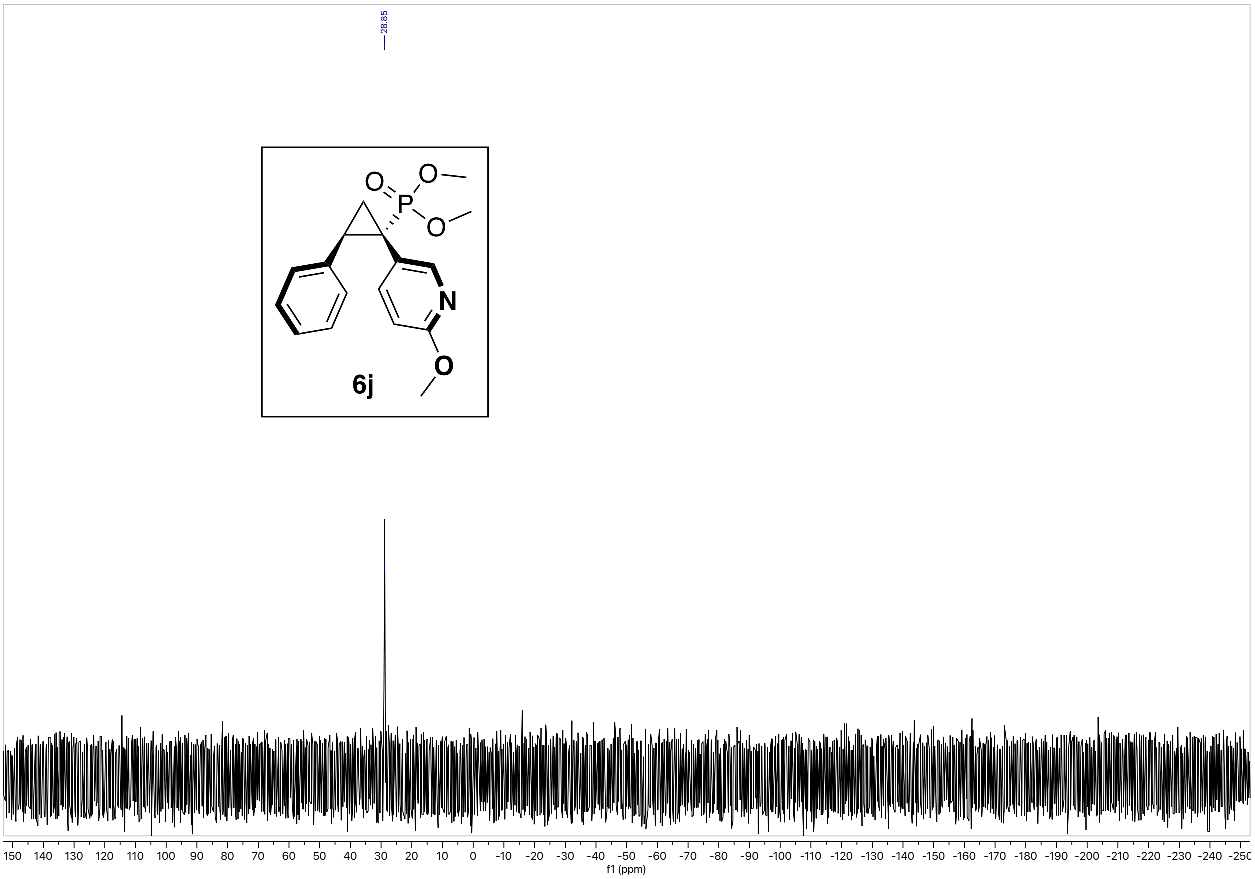

^{31}P NMR spectrum (162 MHz, Chloroform-d) of **6j.**

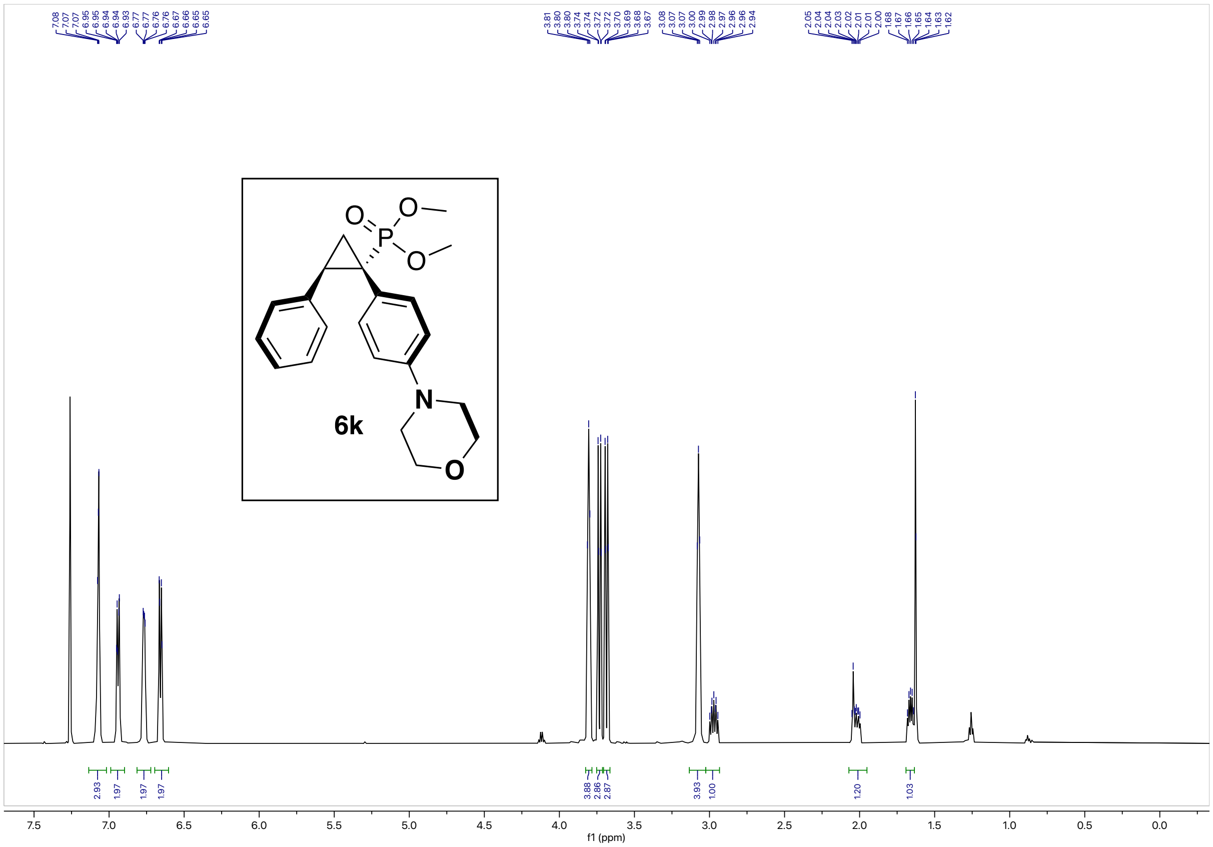

^{1}H NMR spectrum (600 MHz, Chloroform-*d*) (s, 7.26 ppm) of **6k.**

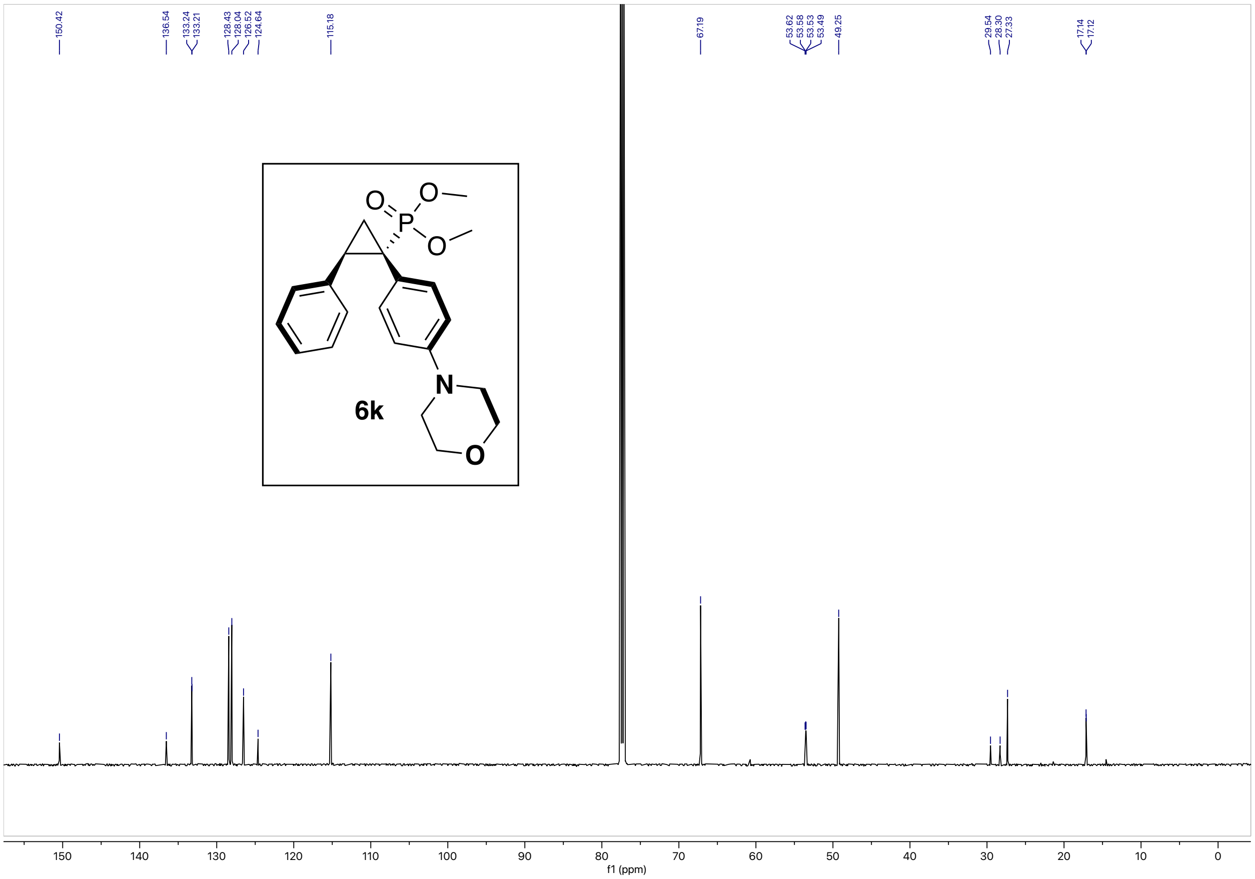

$^{13}C\{^1H\}$ NMR spectrum (151 MHz, Chloroform-*d*) (t, 77.36 ppm) of **6k.**

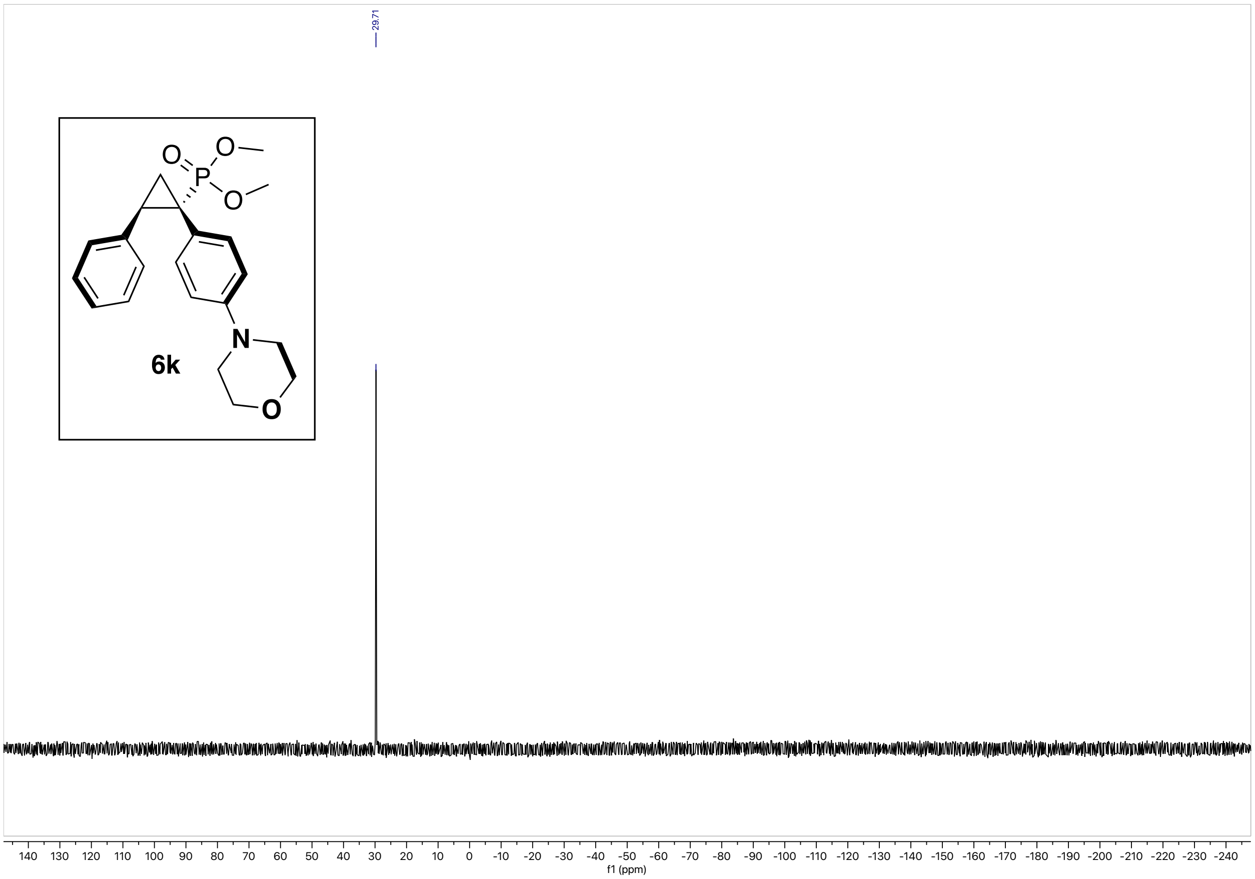

^{31}P NMR spectrum (243 MHz, Chloroform-d) of **6k.**

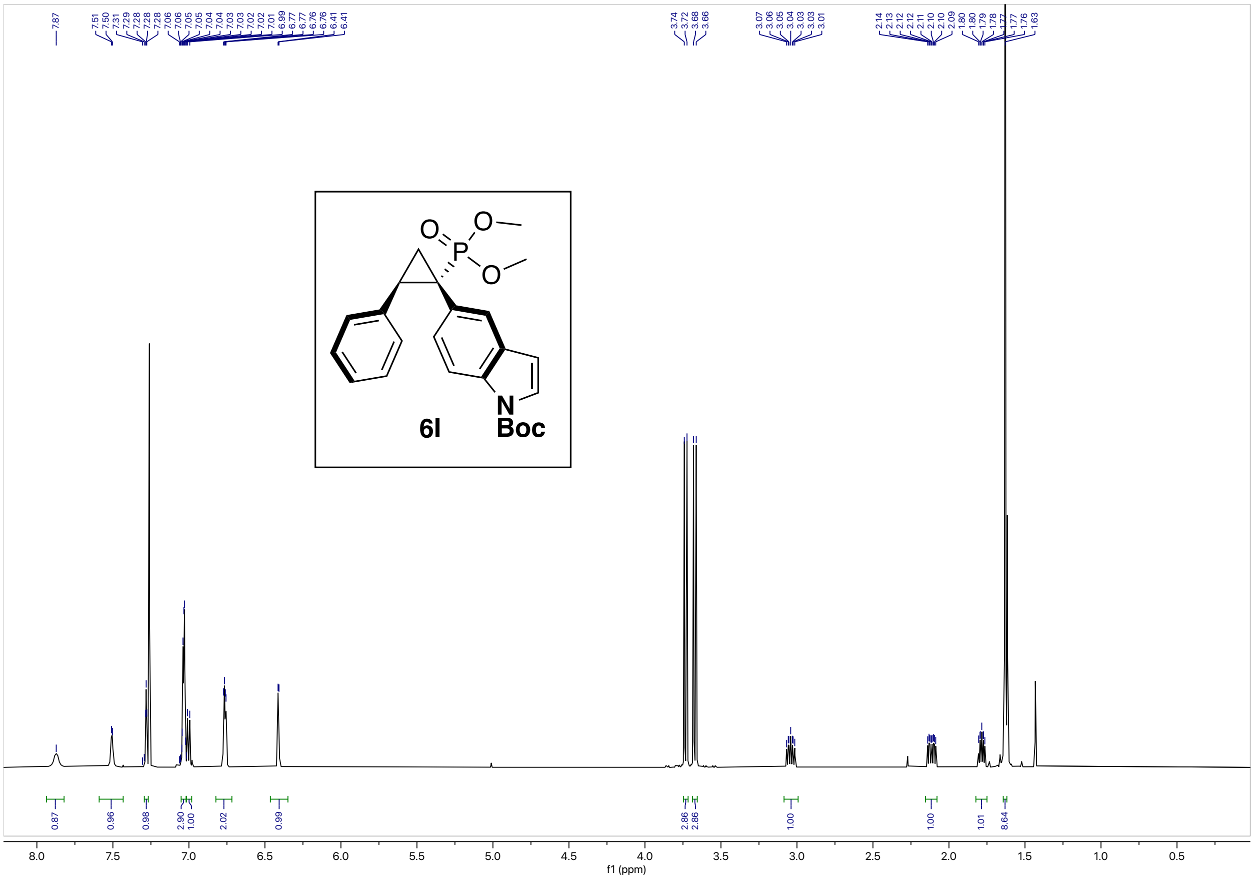

^{1}H NMR spectrum (600 MHz, Chloroform-*d*) (s, 7.26 ppm) of **6l.**

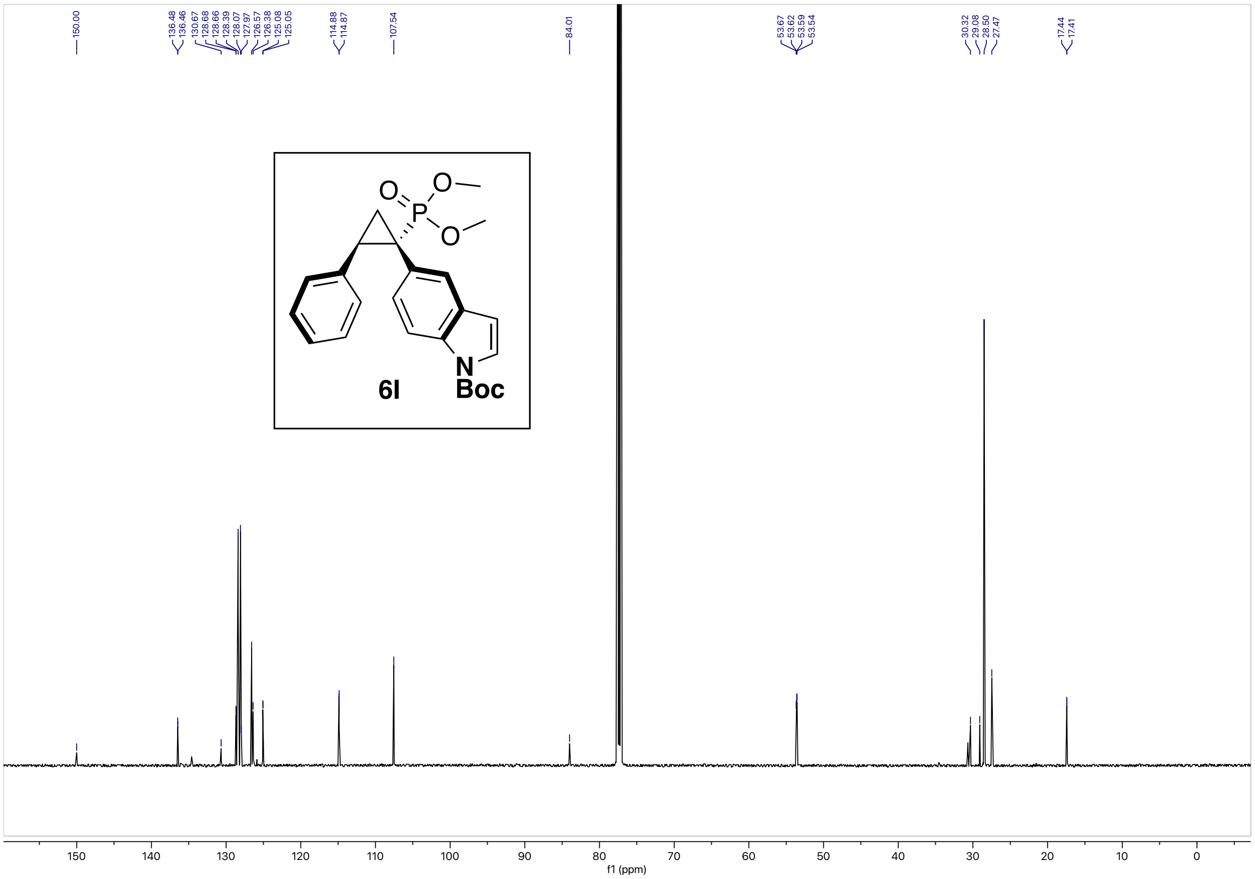

$^{13}C\{^{1}H\}$ NMR spectrum (151 MHz, Chloroform-*d*) (t, 77.36 ppm) of **6l.**

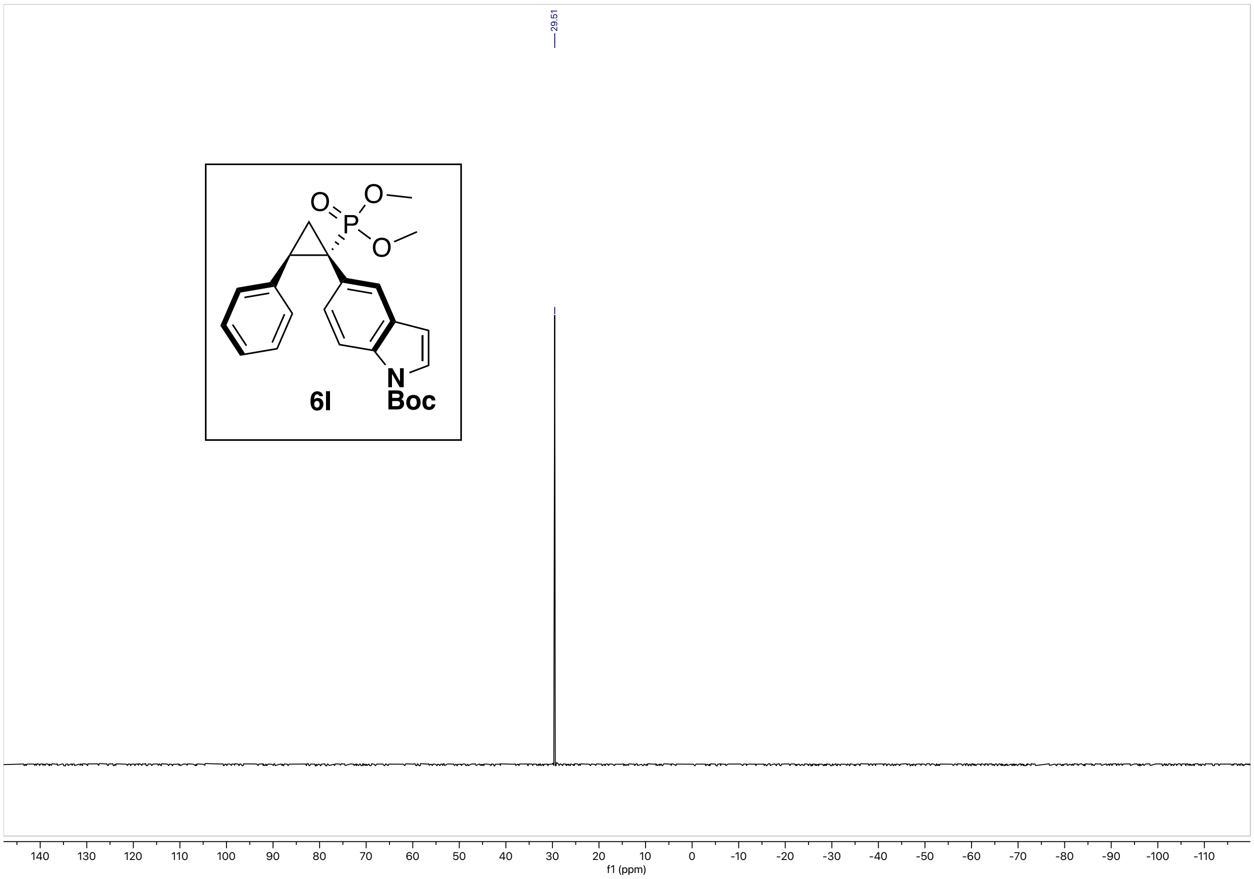

^{31}P NMR spectrum (243 MHz, Chloroform-d) of **6l.**

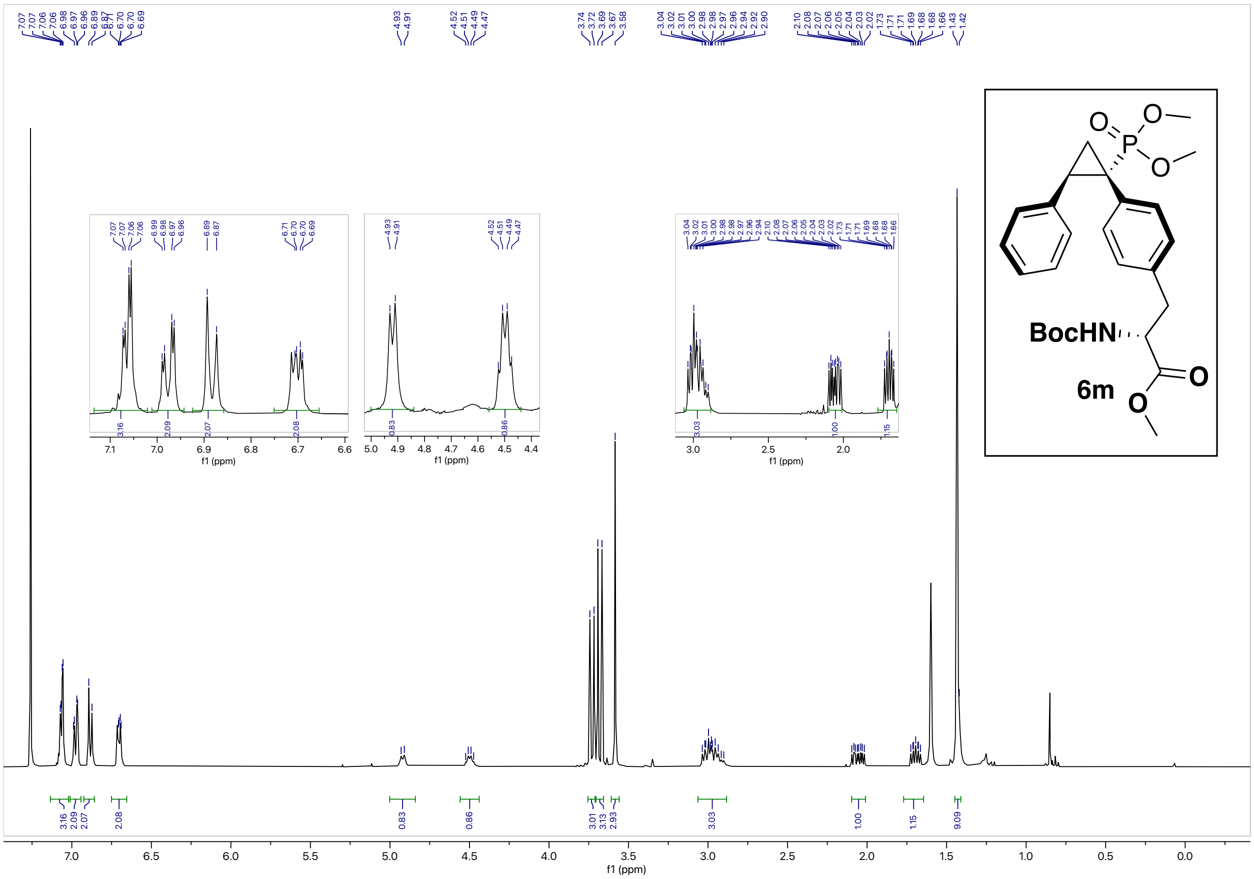

^{1}H NMR spectrum (400 MHz, Chloroform-*d*) (s, 7.26 ppm) of **6m.**

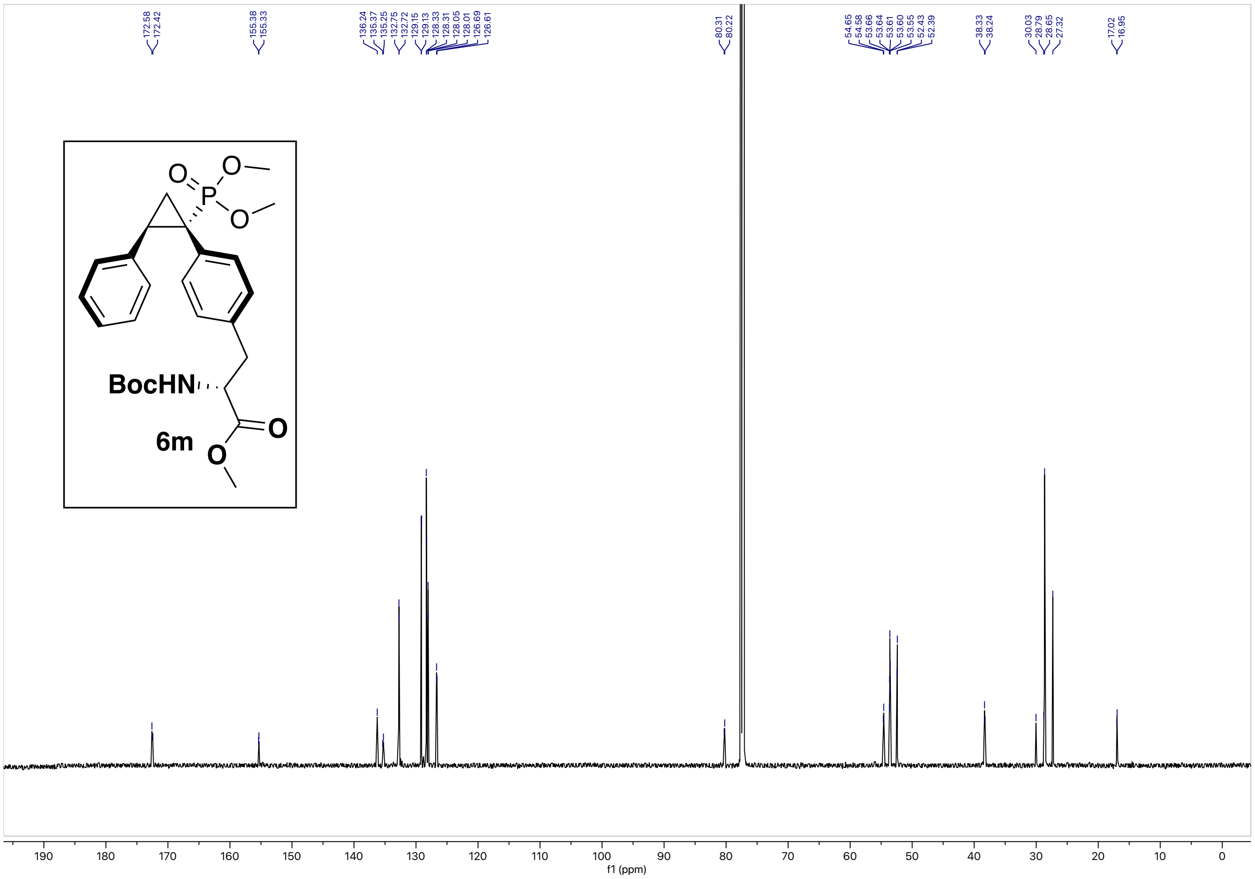

$^{13}C\{^{1}H\}$ NMR spectrum (151 MHz, Chloroform-*d*) (t, 77.36 ppm) of **6m.**

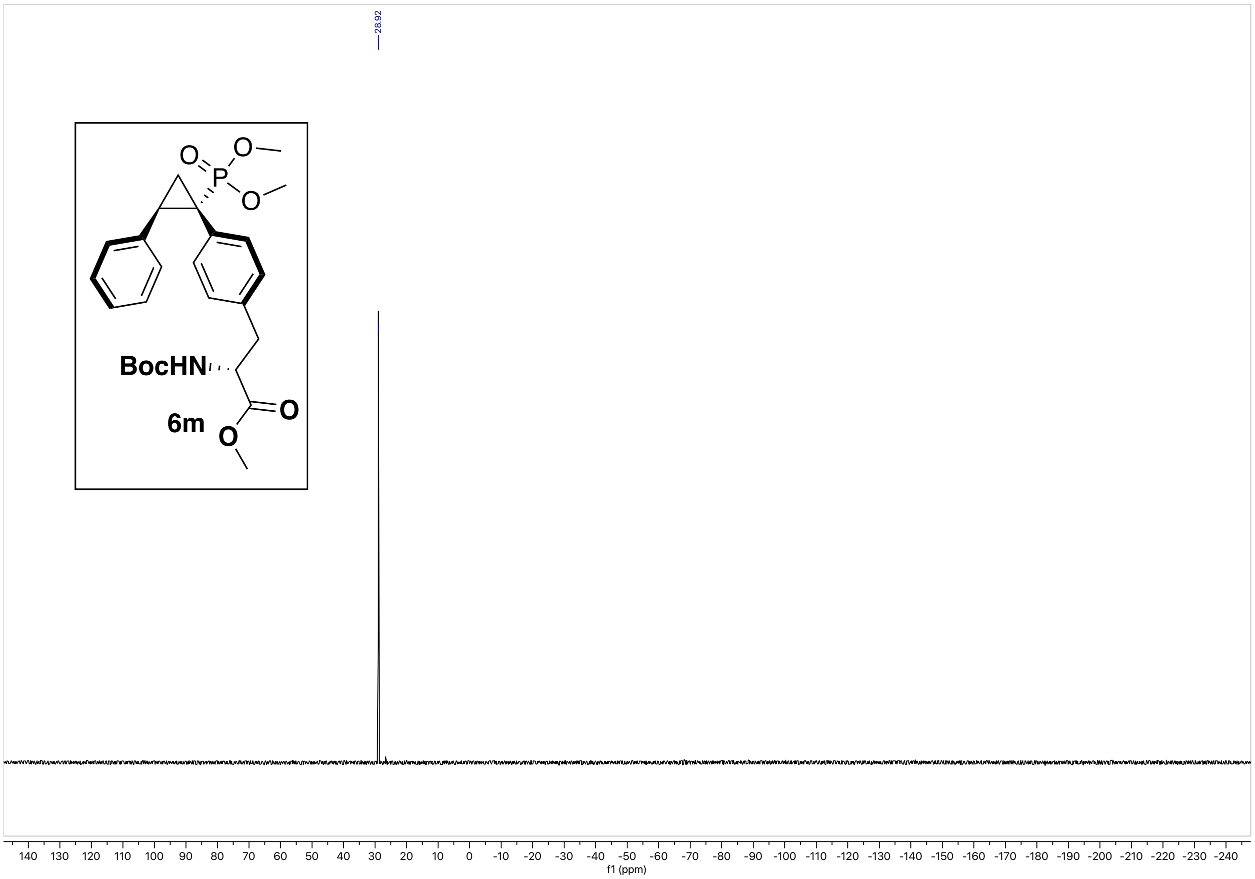

^{31}P NMR spectrum (243 MHz, Chloroform-d) of **6m.**

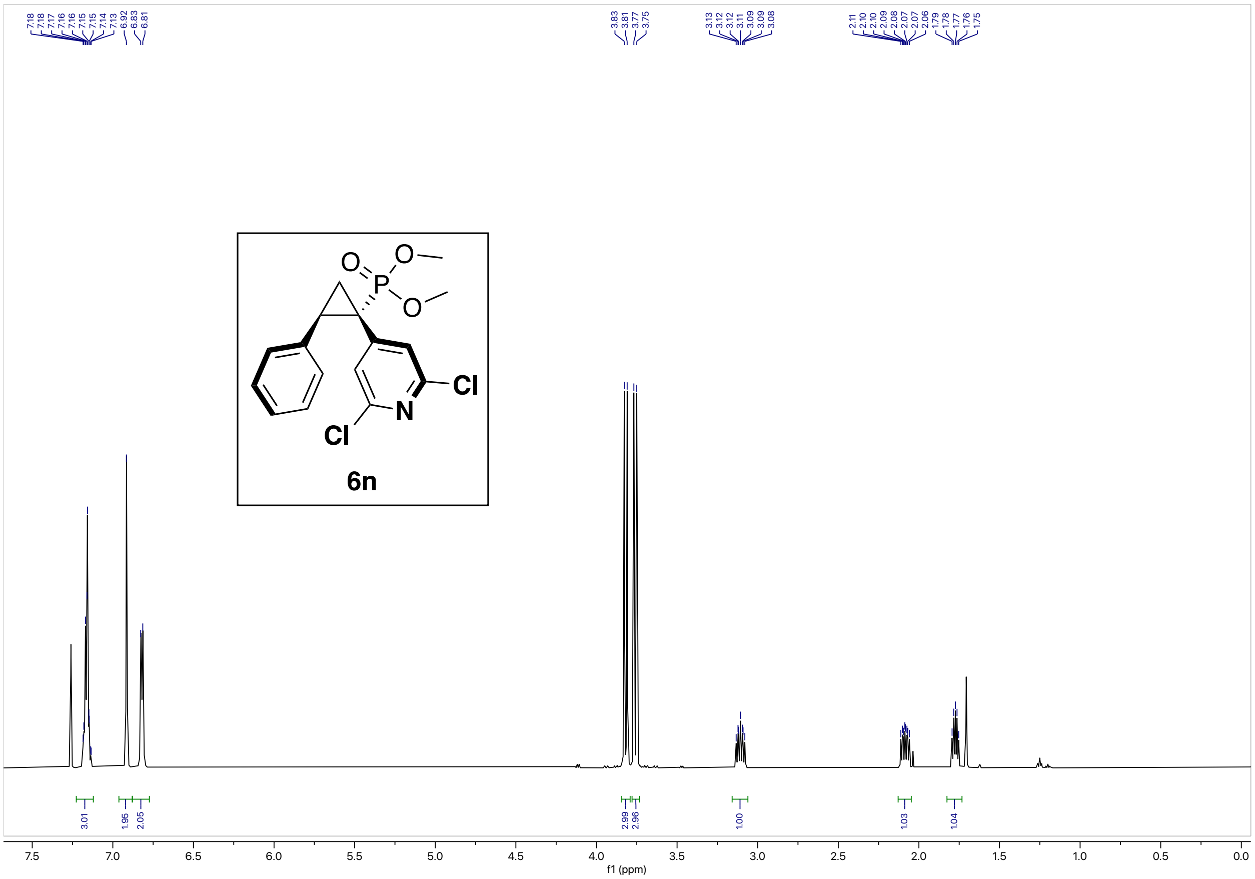

^{1}H NMR spectrum (600 MHz, Chloroform-*d*) (s, 7.26 ppm) of **6n.**

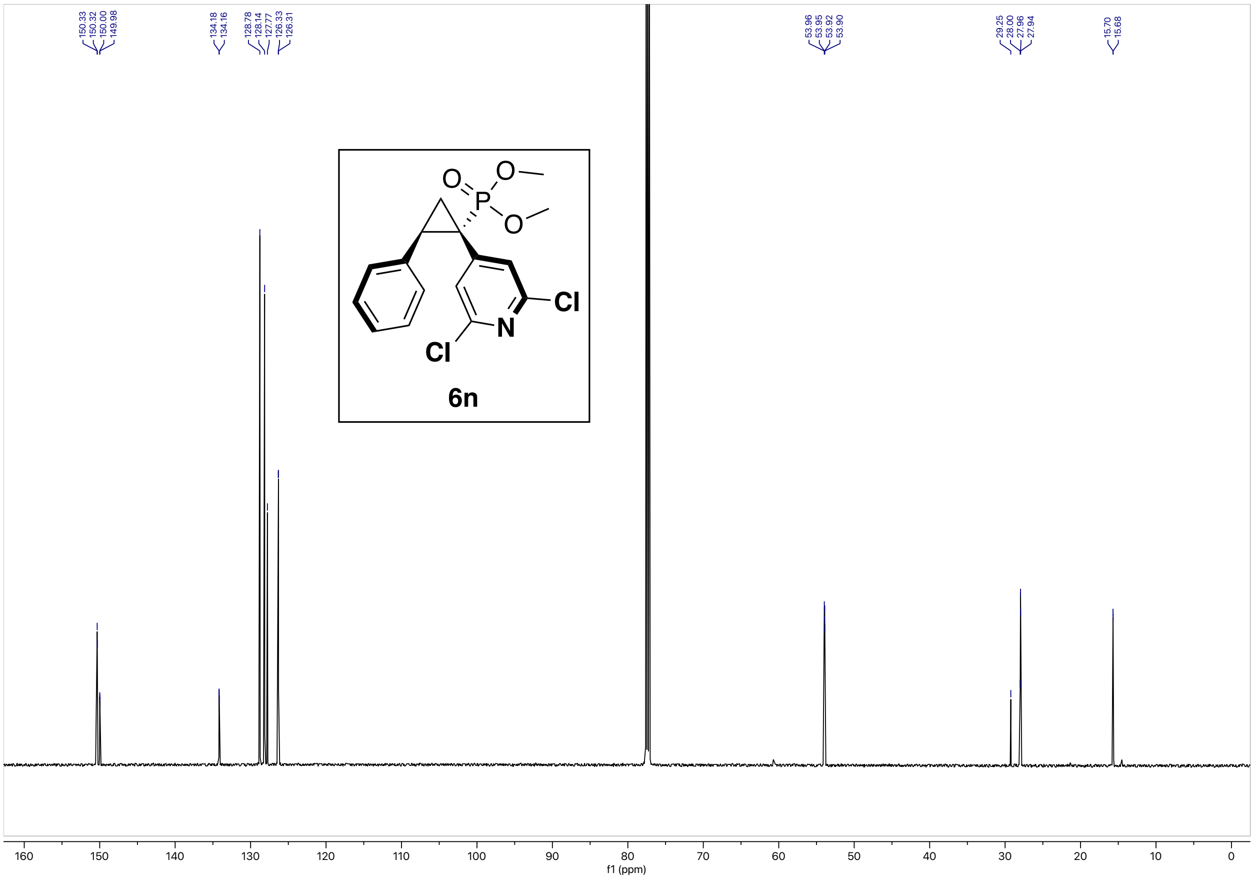

$^{13}C\{^1H\}$ NMR spectrum (151 MHz, Chloroform-*d*) (t, 77.36 ppm) of **6n.**

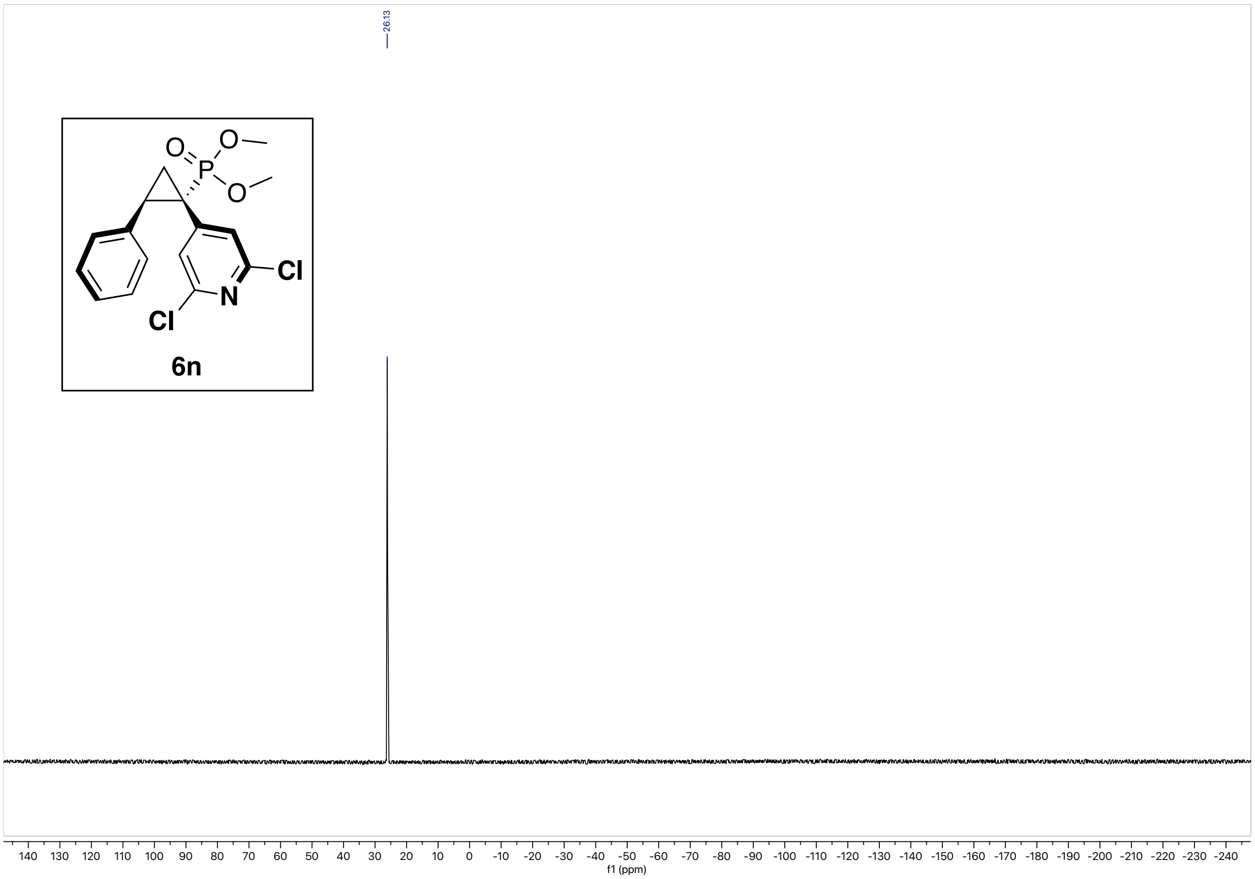

^{31}P NMR spectrum (243 MHz, Chloroform-d) of **6n.**

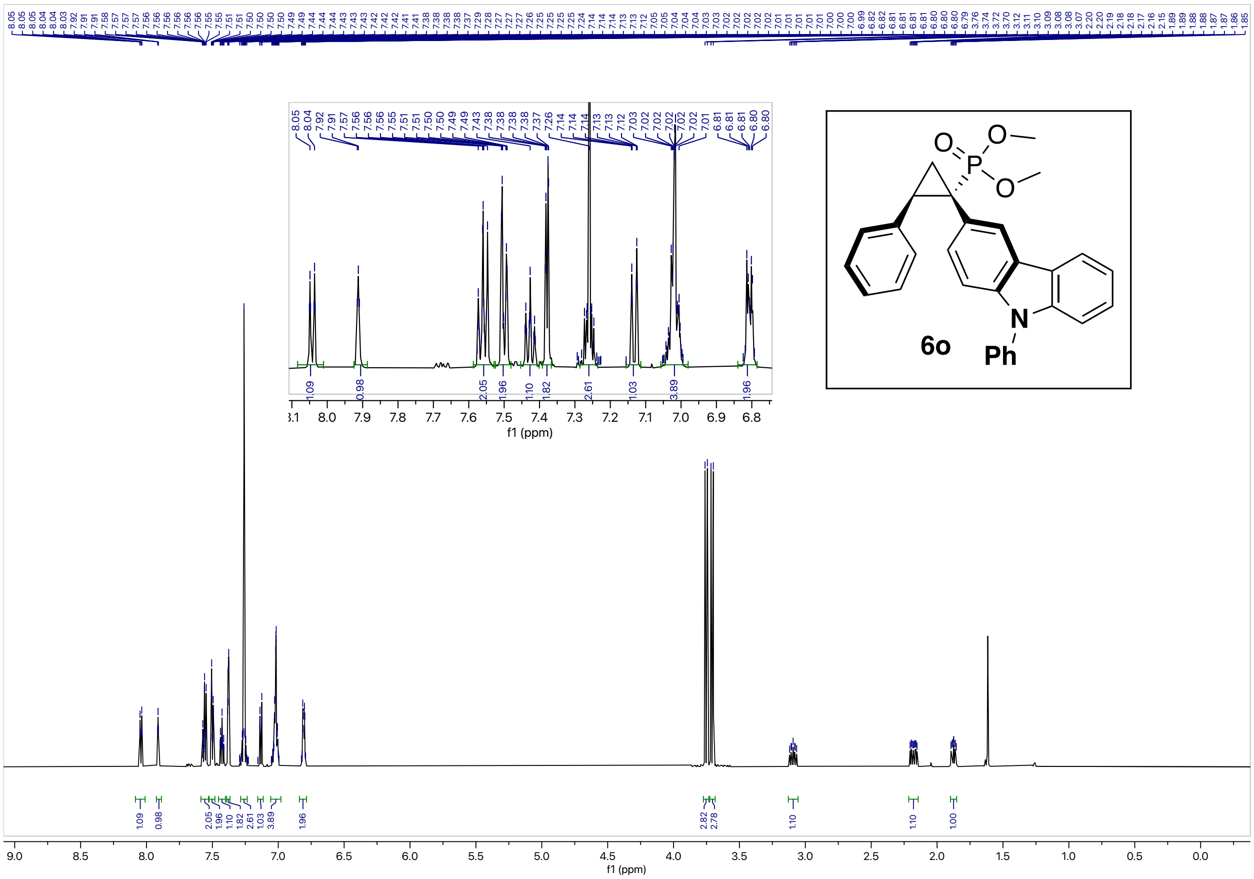

^{1}H NMR spectrum (600 MHz, Chloroform-*d*) (s, 7.26 ppm) of **6o.**

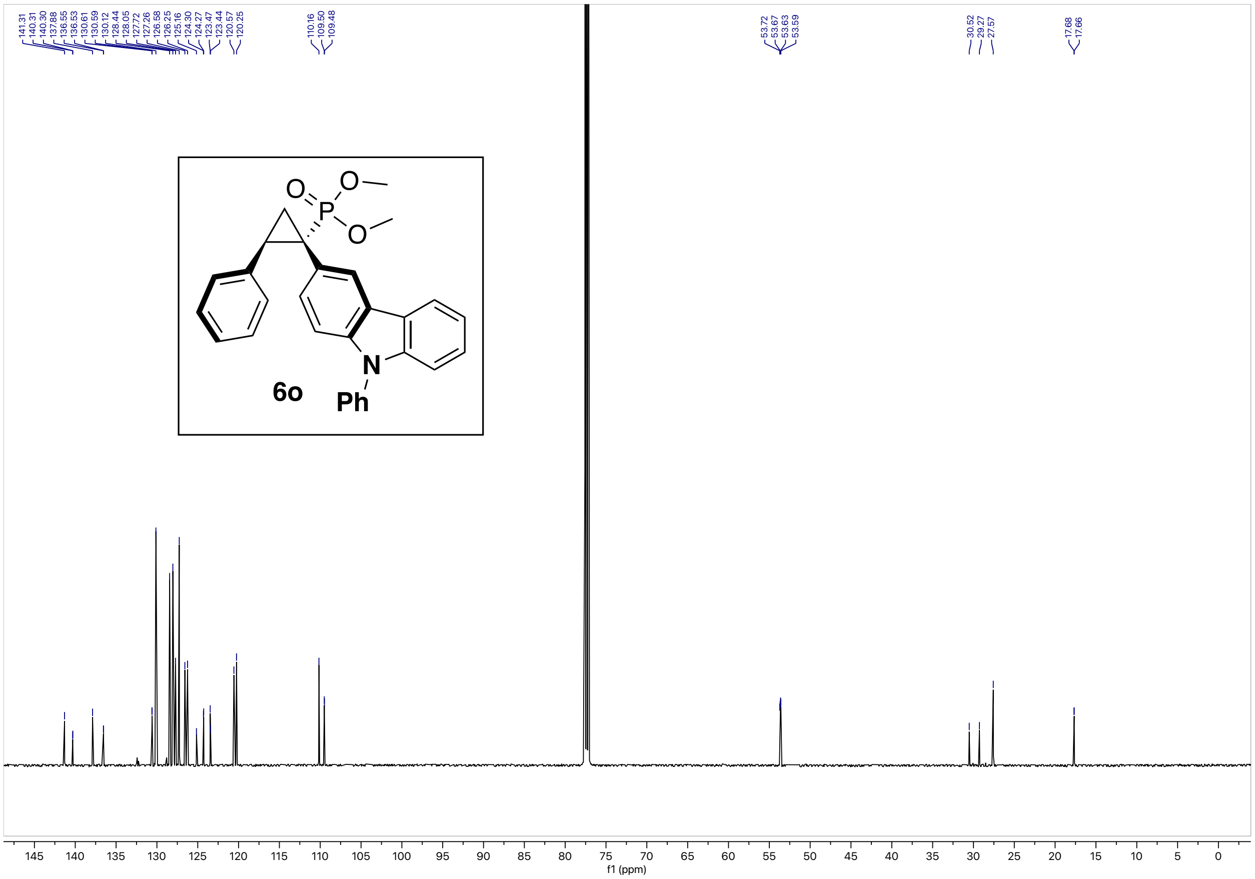

$^{13}C\{^{1}H\}$ NMR spectrum (151 MHz, Chloroform-*d*) (t, 77.36 ppm) of **6o.**

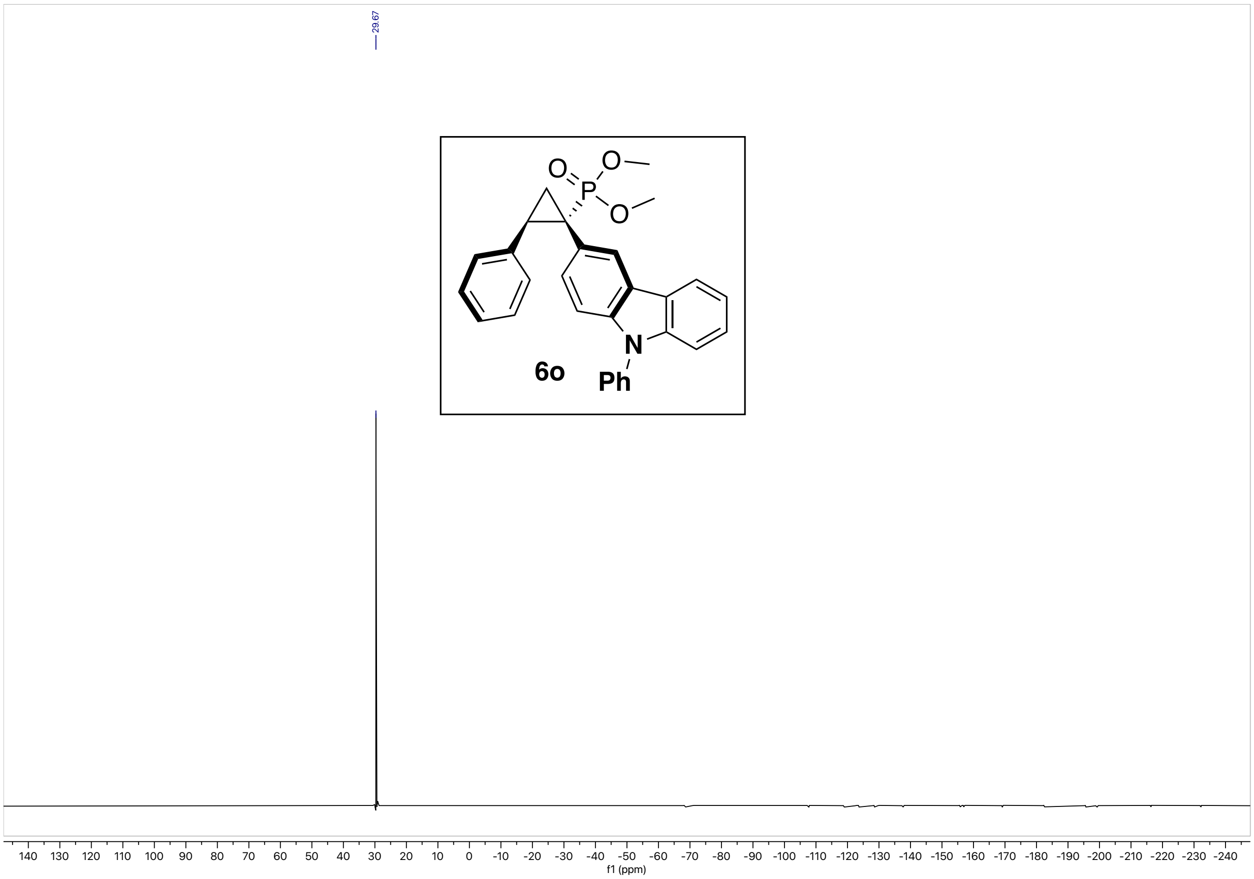

^{31}P NMR spectrum (243 MHz, Chloroform-d) of **6o.**

8. Enantioselectivity Determination by HPLC or SFC

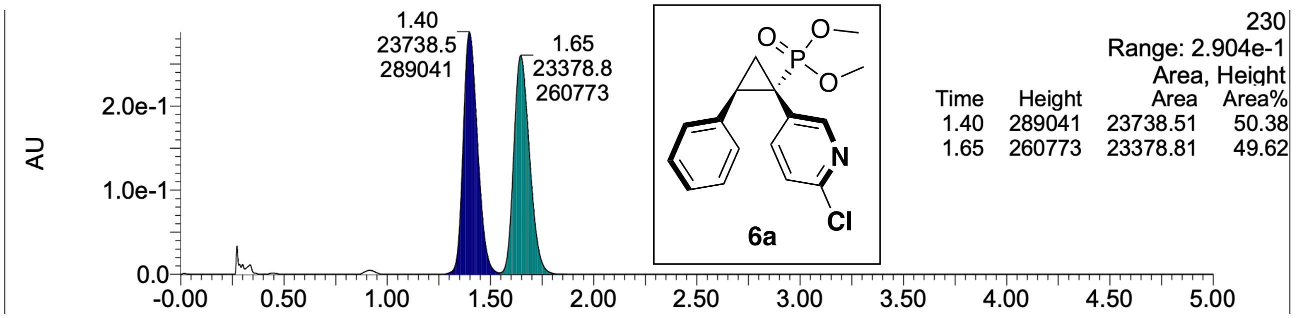

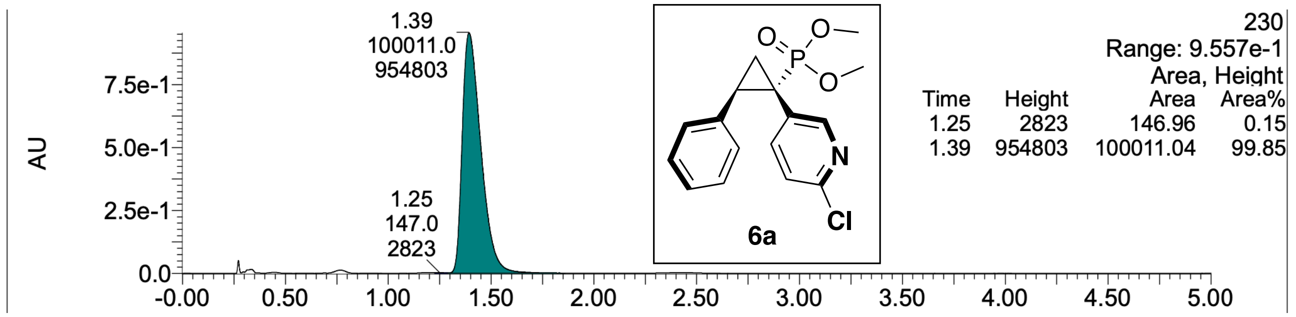

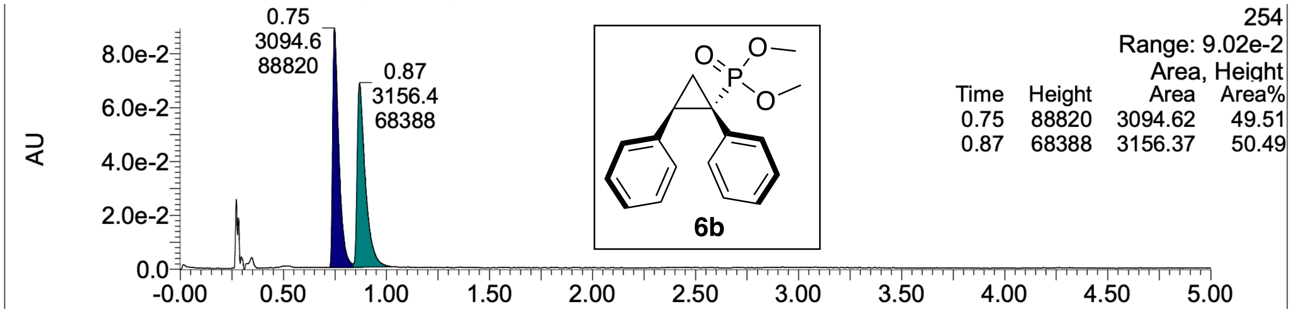

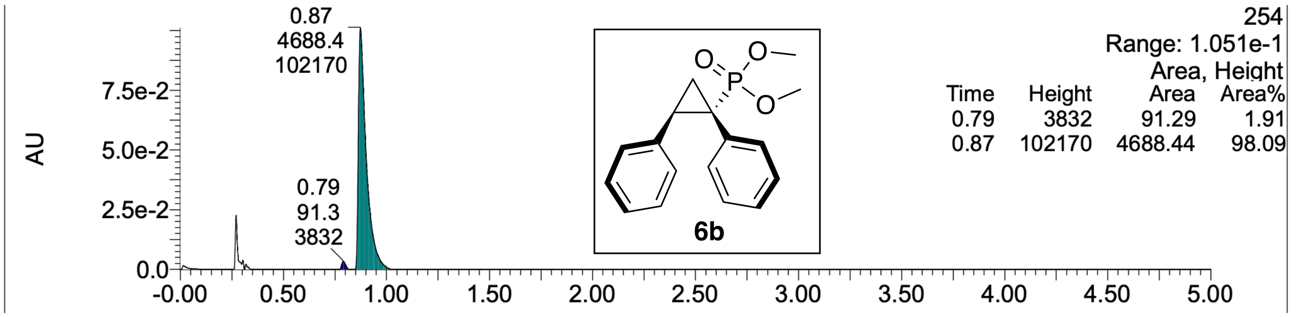

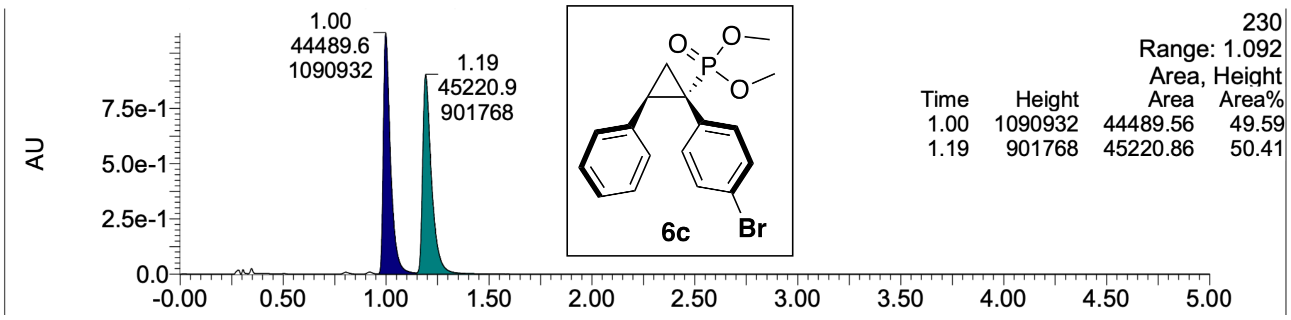
1.00
44489.6
1090932
1.19
45220.9
901768
6c
Br
230
Range: 1.092
Area, Height
Time Height Area Area%
1.00 1090932 44489.56 49.59
1.19 901768 45220.86 50.41
AU

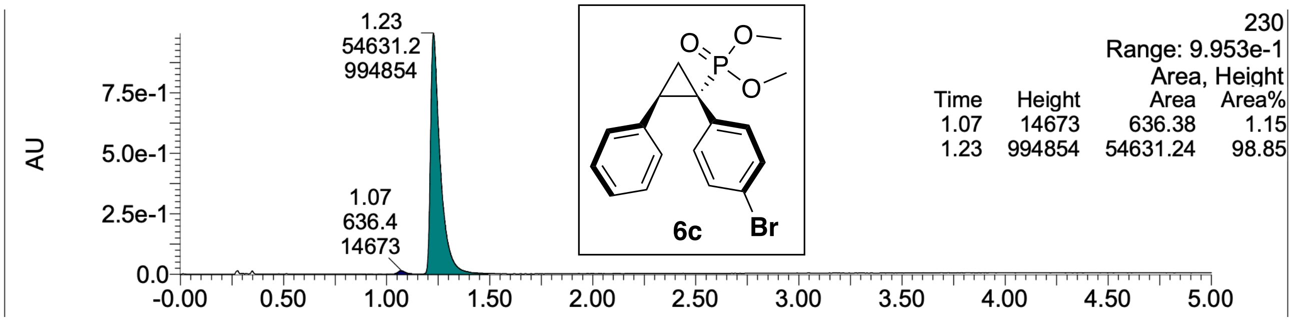
1.23
54631.2
994854
1.07
636.4
14673
6c
Br
230
Range: 9.953e-1
Area, Height
Time Height Area Area%
1.07 14673 636.38 1.15
1.23 994854 54631.24 98.85
AU

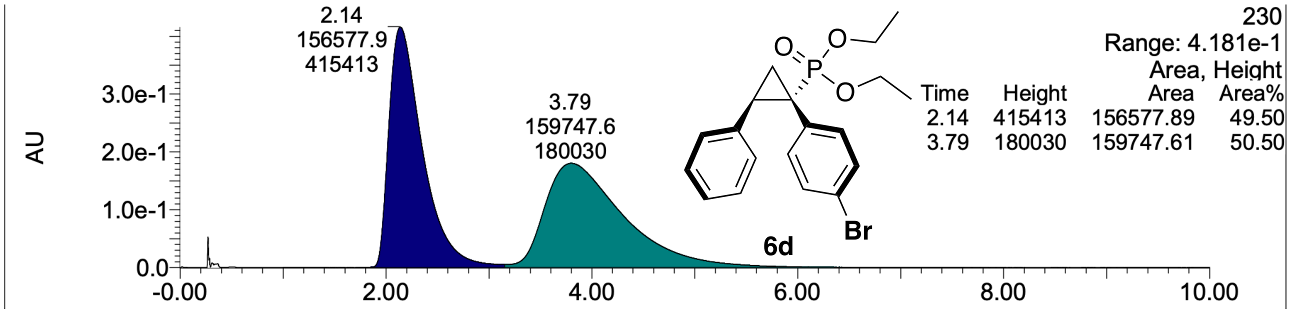
2.14
156577.9
415413
3.79
159747.6
180030
6d
Br
230
Range: 4.181e-1
Area, Height
Time Height Area Area%
2.14 415413 156577.89 49.50
3.79 180030 159747.61 50.50
AU

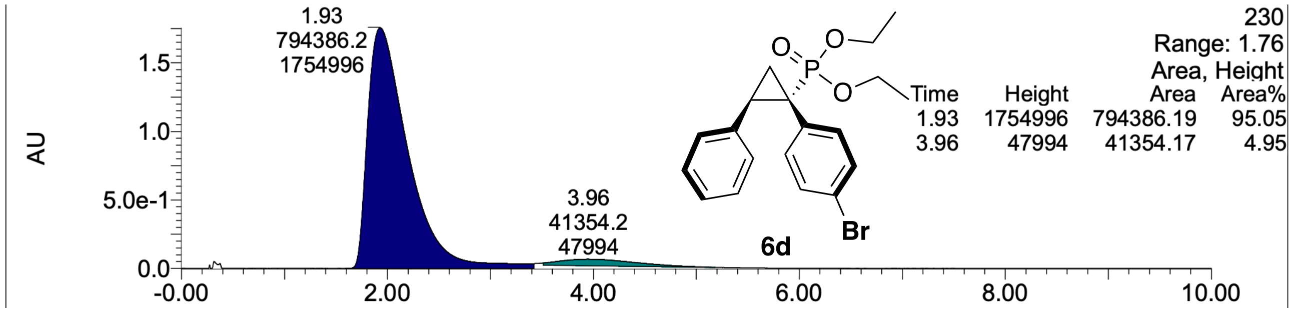
1.93
794386.2
1754996
3.96
41354.2
47994
6d
Br
230
Range: 1.76
Area, Height
Time Height Area Area%
1.93 1754996 794386.19 95.05
3.96 47994 41354.17 4.95
AU

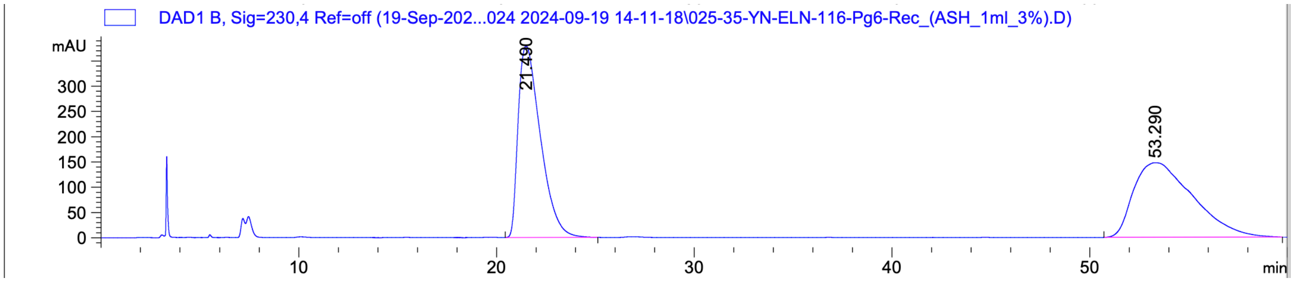

Signal 2: DAD1 B, Sig=230,4 Ref=off

Peak #	RetTime [min]	Type	Width [min]	Area [mAU*s]	Height [mAU]	Area %
1	21.490	BB	0.9190	2.96888e4	378.76147	49.0181
2	53.290	BB	2.4409	3.08782e4	147.73773	50.9819

Totals : 6.05670e4 526.49921

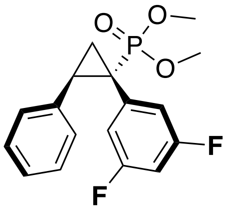

6e

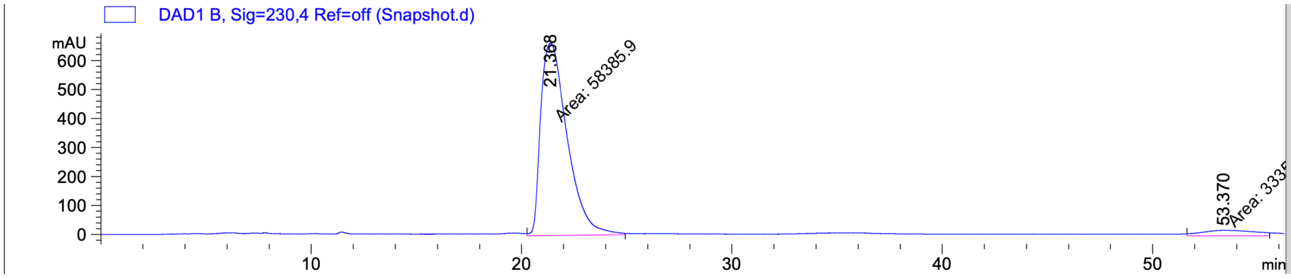

Signal 2: DAD1 B, Sig=230,4 Ref=off

Peak #	RetTime [min]	Type	Width [min]	Area [mAU*s]	Height [mAU]	Area %
1	21.368	MM	1.4662	5.83859e4	663.68878	94.5962
2	53.370	MM	2.9732	3335.29956	18.69671	5.4038

Totals : 6.17212e4 682.38549

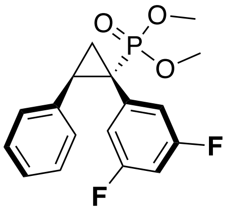

6e

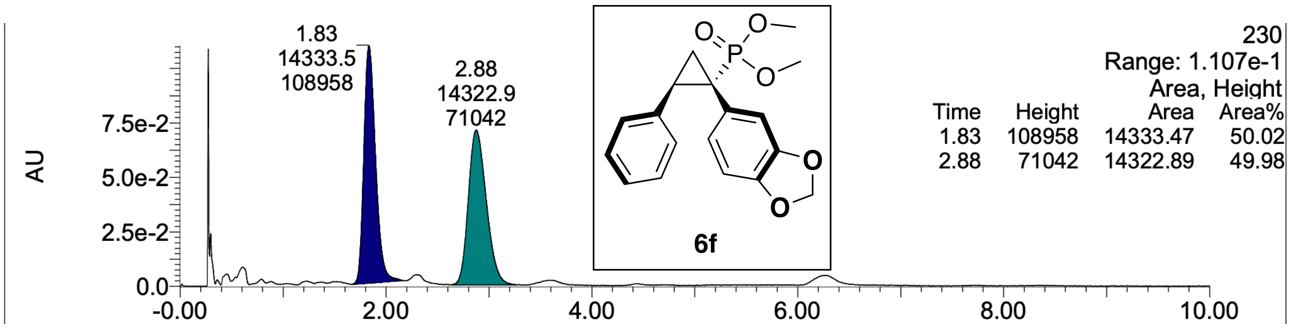
AU
7.5e-2
5.0e-2
2.5e-2
0.0
-0.00
2.00
4.00
6.00
8.00
10.00
1.83
14333.5
108958
2.88
14322.9
71042
6f
230
Range: 1.107e-1
Area, Height
Time Height Area Area%
1.83 108958 14333.47 50.02
2.88 71042 14322.89 49.98

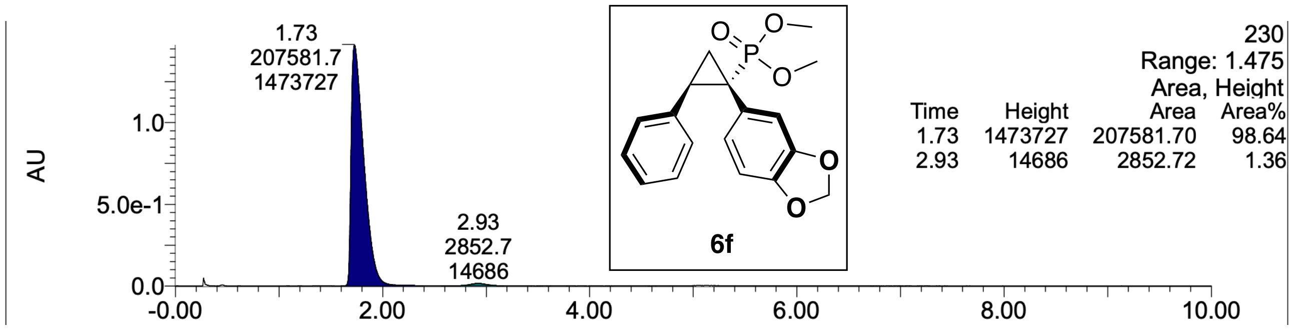
AU
1.0
5.0e-1
0.0
-0.00
2.00
4.00
6.00
8.00
10.00
1.73
207581.7
1473727
2.93
2852.7
14686
6f
230
Range: 1.475
Area, Height
Time Height Area Area%
1.73 1473727 207581.70 98.64
2.93 14686 2852.72 1.36

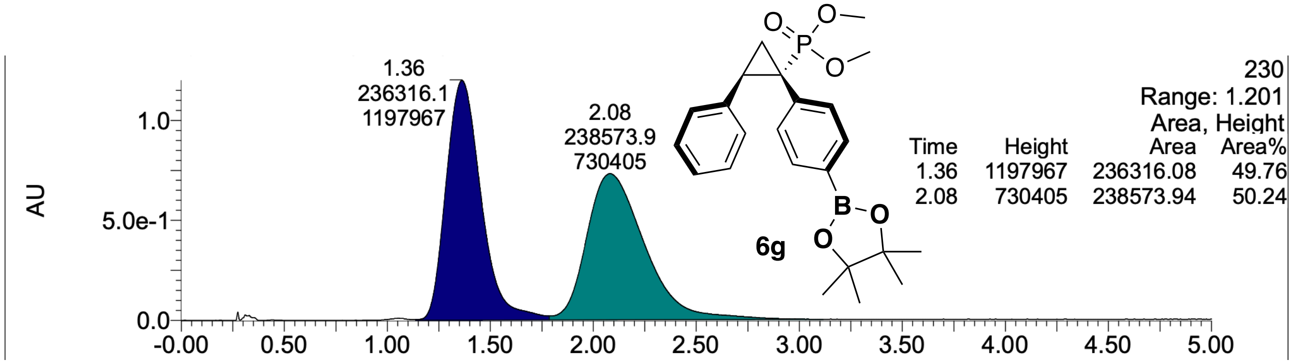
AU
1.0
5.0e-1
0.0
-0.00
0.50
1.00
1.50
2.00
2.50
3.00
3.50
4.00
4.50
5.00
1.36
236316.1
1197967
2.08
238573.9
730405
6g
230
Range: 1.201
Area, Height
Time Height Area Area%
1.36 1197967 236316.08 49.76
2.08 730405 238573.94 50.24

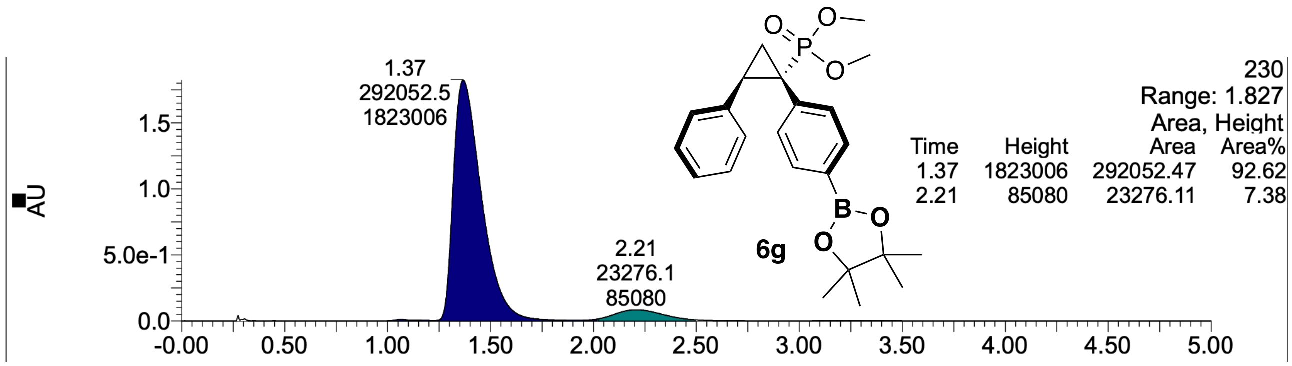
AU
1.5
1.0
5.0e-1
0.0
-0.00
0.50
1.00
1.50
2.00
2.50
3.00
3.50
4.00
4.50
5.00
1.37
292052.5
1823006
2.21
23276.1
85080
6g
230
Range: 1.827
Area, Height
Time Height Area Area%
1.37 1823006 292052.47 92.62
2.21 85080 23276.11 7.38

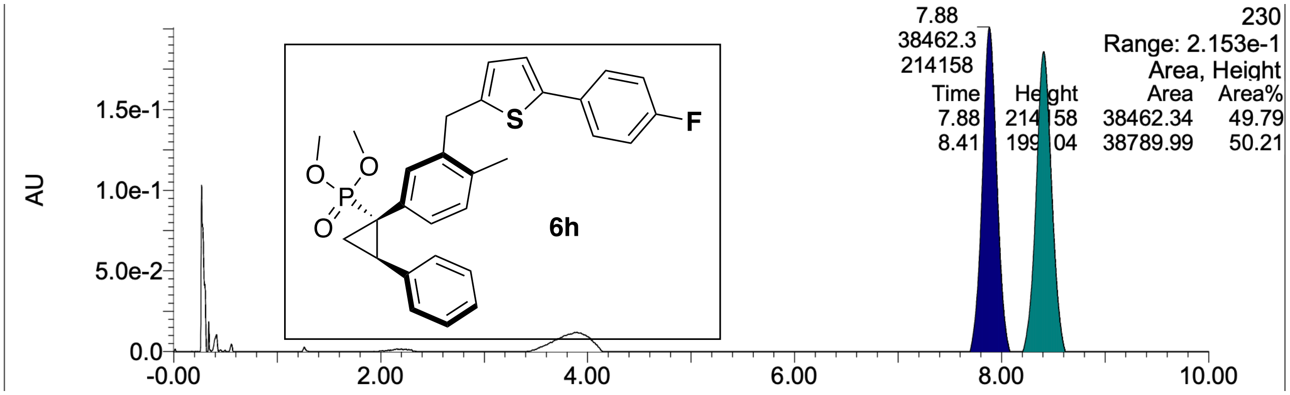

7.88
38462.3
214158
230
Range: 2.153e-1
Area, Height
Time Height Area Area%
7.88 38462.34 49.79
8.41 38789.99 50.21
6h
AU

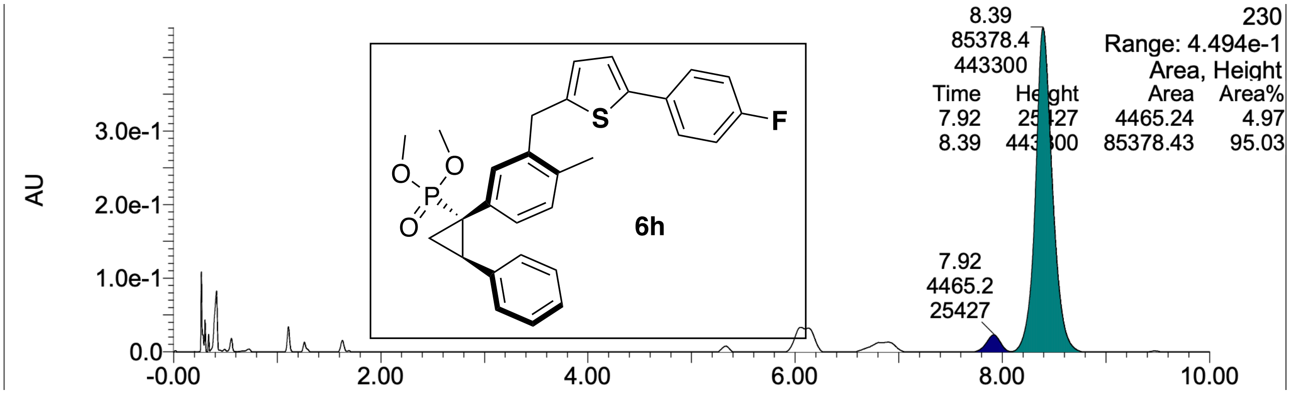

8.39
85378.4
443300
230
Range: 4.494e-1
Area, Height
Time Height Area Area%
7.92 4465.24 4.97
8.39 85378.43 95.03
7.92
4465.2
25427
6h
AU

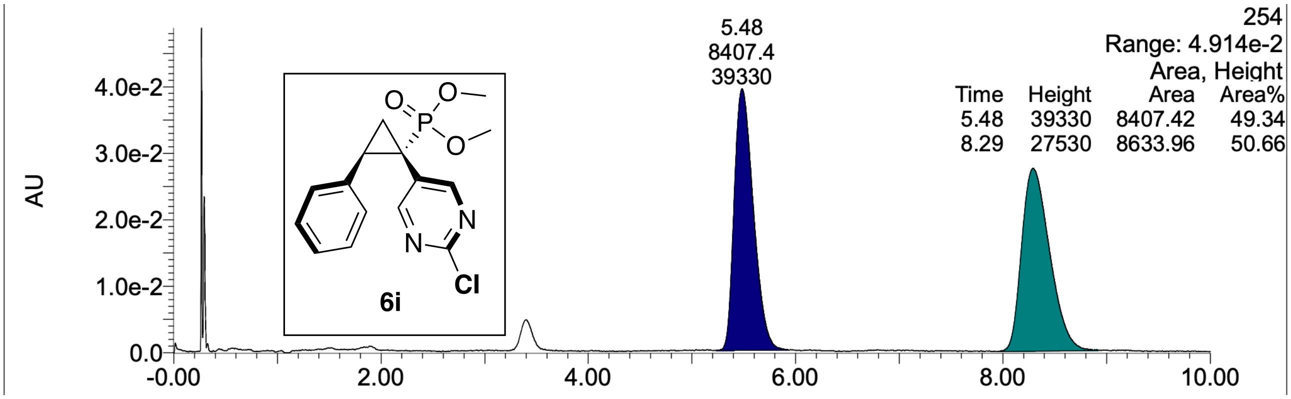

5.48
8407.4
39330
254
Range: 4.914e-2
Area, Height
Time Height Area Area%
5.48 39330 8407.42 49.34
8.29 27530 8633.96 50.66
6i
AU

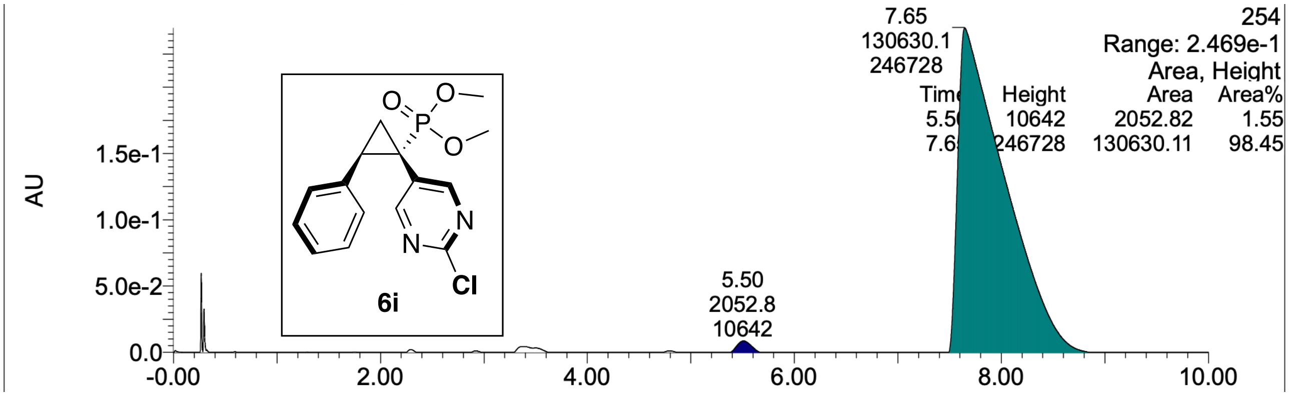

7.65
130630.1
246728
254
Range: 2.469e-1
Area, Height
Height Area Area%
10642 2052.82 1.55
246728 130630.11 98.45
5.50
2052.8
10642
6i
AU

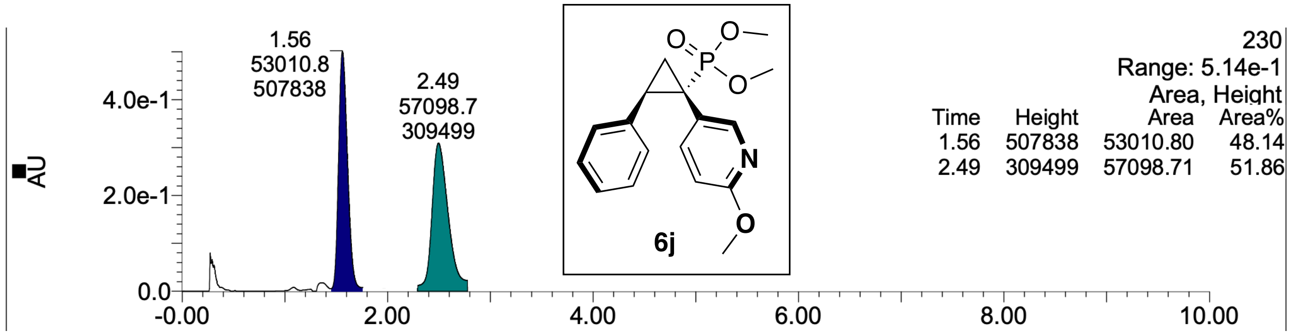
1.56
53010.8
507838
2.49
57098.7
309499
6j
230
Range: 5.14e-1
Area, Height
Time Height Area Area%
1.56 507838 53010.80 48.14
2.49 309499 57098.71 51.86
AU
4.0e-1
2.0e-1
0.0
-0.00 2.00 4.00 6.00 8.00 10.00

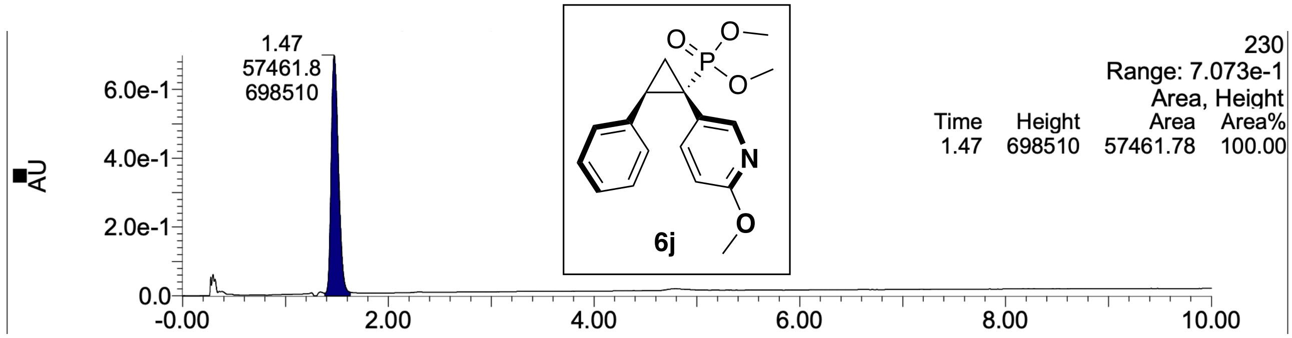
1.47
57461.8
698510
6j
230
Range: 7.073e-1
Area, Height
Time Height Area Area%
1.47 698510 57461.78 100.00
AU
6.0e-1
4.0e-1
2.0e-1
0.0
-0.00 2.00 4.00 6.00 8.00 10.00

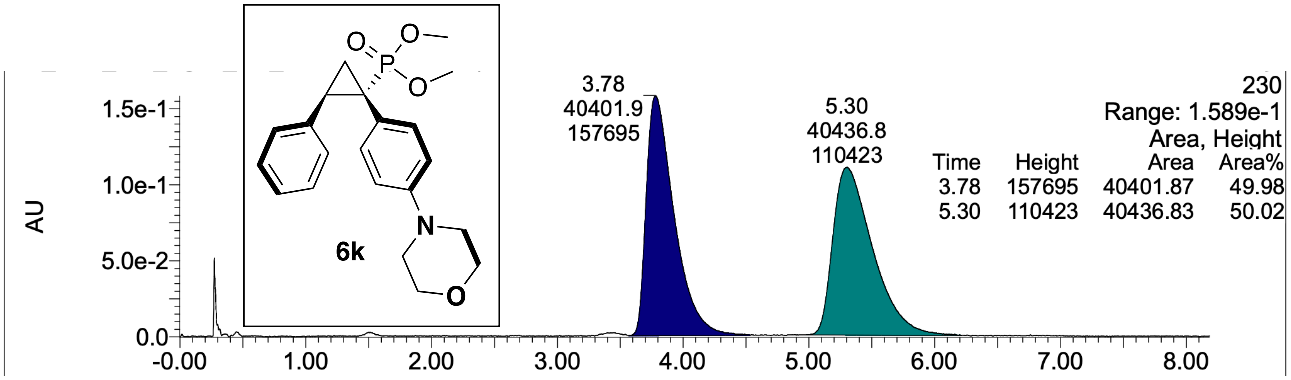
6k
3.78
40401.9
157695
5.30
40436.8
110423
230
Range: 1.589e-1
Area, Height
Time Height Area Area%
3.78 157695 40401.87 49.98
5.30 110423 40436.83 50.02
AU
1.5e-1
1.0e-1
5.0e-2
0.0
-0.00 1.00 2.00 3.00 4.00 5.00 6.00 7.00 8.00

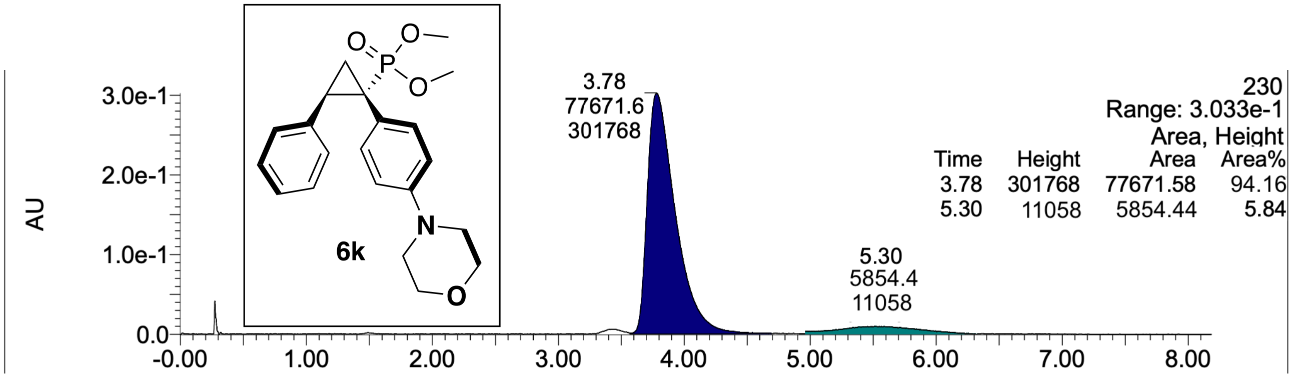
6k
3.78
77671.6
301768
5.30
5854.4
11058
230
Range: 3.033e-1
Area, Height
Time Height Area Area%
3.78 301768 77671.58 94.16
5.30 11058 5854.44 5.84
AU
3.0e-1
2.0e-1
1.0e-1
0.0
-0.00 1.00 2.00 3.00 4.00 5.00 6.00 7.00 8.00

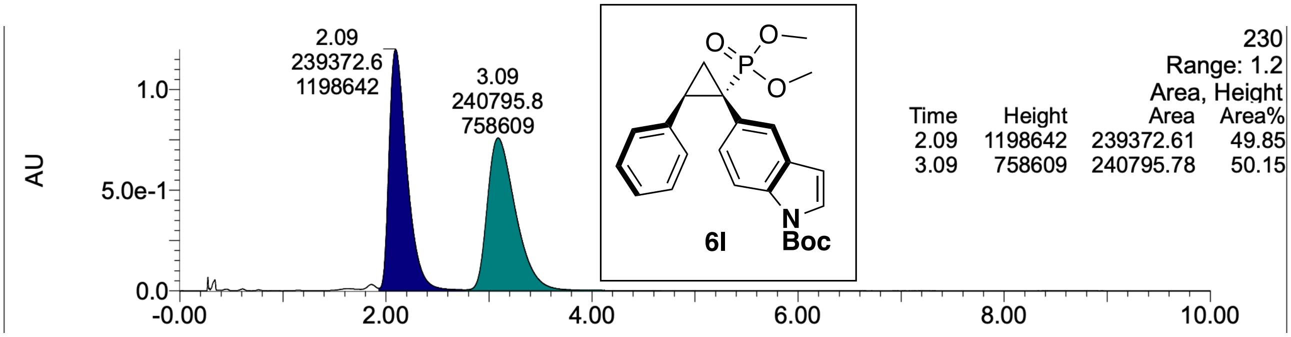

Time	Height	Area	Area%
2.09	1198642	239372.61	49.85
3.09	758609	240795.78	50.15

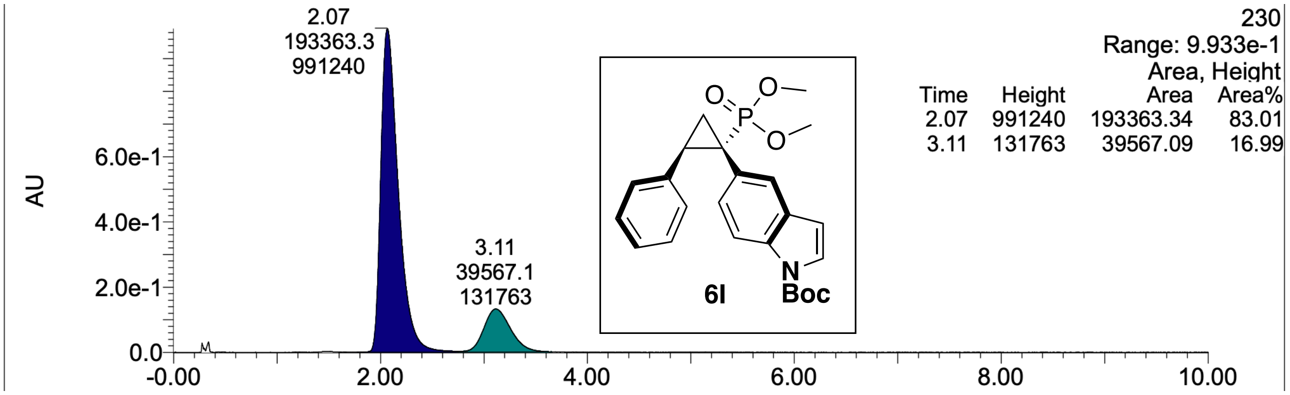

Time	Height	Area	Area%
2.07	991240	193363.34	83.01
3.11	131763	39567.09	16.99

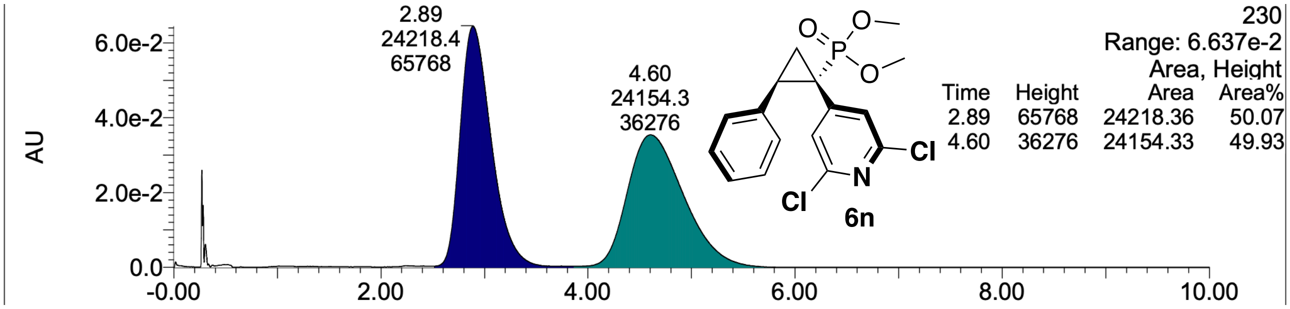

Time	Height	Area	Area%
2.89	65768	24218.36	50.07
4.60	36276	24154.33	49.93

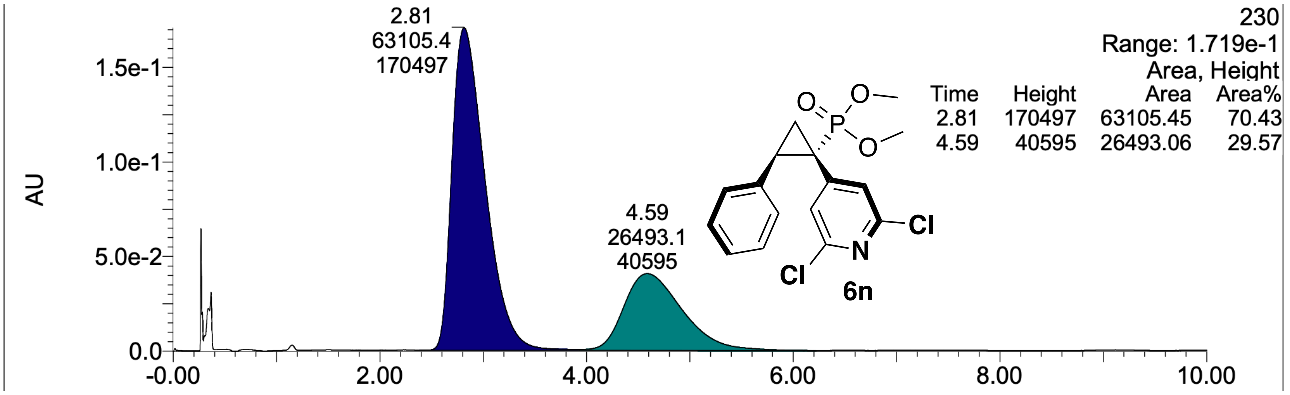

Time	Height	Area	Area%
2.81	170497	63105.45	70.43
4.59	40595	26493.06	29.57

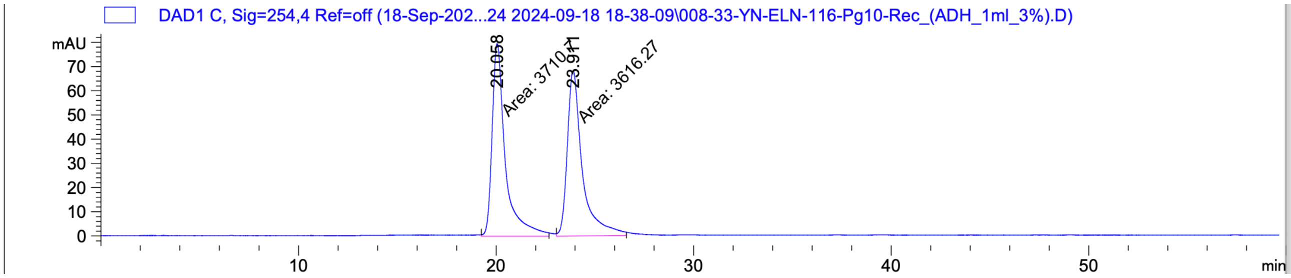

Signal 3: DAD1 C, Sig=254,4 Ref=off

Peak #	RetTime [min]	Type	Width [min]	Area [mAU*s]	Height [mAU]	Area %
1	20.058	MM	0.7771	3710.70142	79.58295	50.6444
2	23.911	MM	0.8804	3616.27075	68.45638	49.3556

Totals : 7326.97217 148.03933

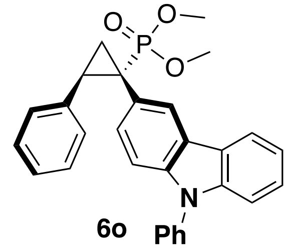

6o

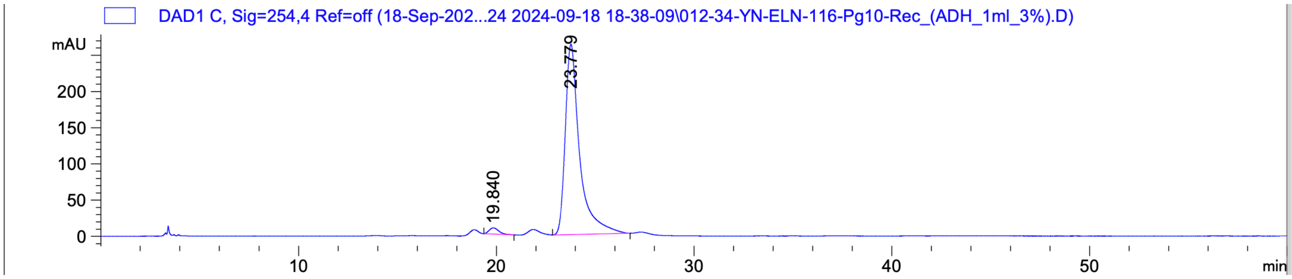

Signal 3: DAD1 C, Sig=254,4 Ref=off

Peak #	RetTime [min]	Type	Width [min]	Area [mAU*s]	Height [mAU]	Area %
1	19.840	BB	0.3942	286.00339	8.52900	2.1111
2	23.779	BB	0.6542	1.32616e4	262.60242	97.8889

Totals : 1.35476e4 271.13142

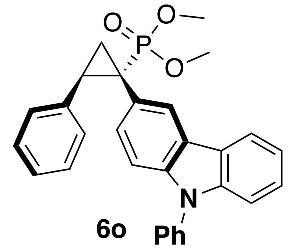

6o

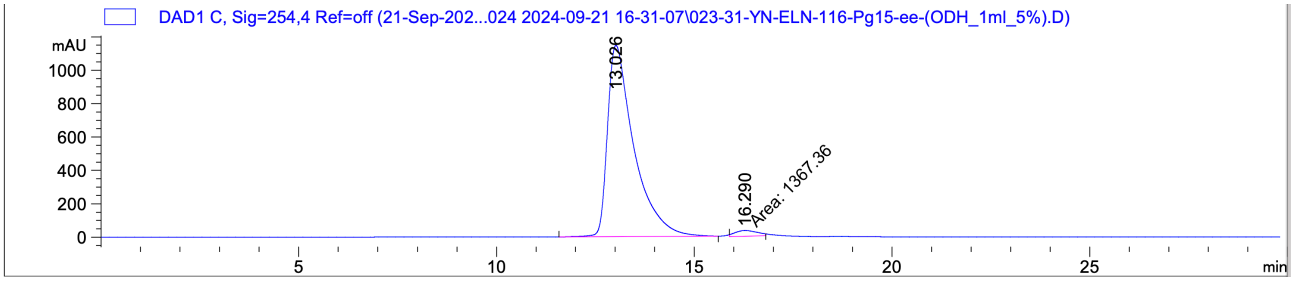

Signal 3: DAD1 C, Sig=254,4 Ref=off

Peak #	RetTime [min]	Type	Width [min]	Area [mAU*s]	Height [mAU]	Area %
1	13.026	BB	0.5564	5.31636e4	1145.58081	97.4925
2	16.290	MM	0.4673	1367.35583	34.50927	2.5075

Totals : 5.45309e4 1180.09008

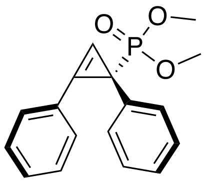

8a

9. X-Ray Crystallographic Data for Compound 6a, 6c, 6g, and 6j. Submitted by: Yasir Naeem Solved by: John Bacsa

Compound 6a (CCDC 2415244)

R_1=3.70%

Crystal Data and Experimental

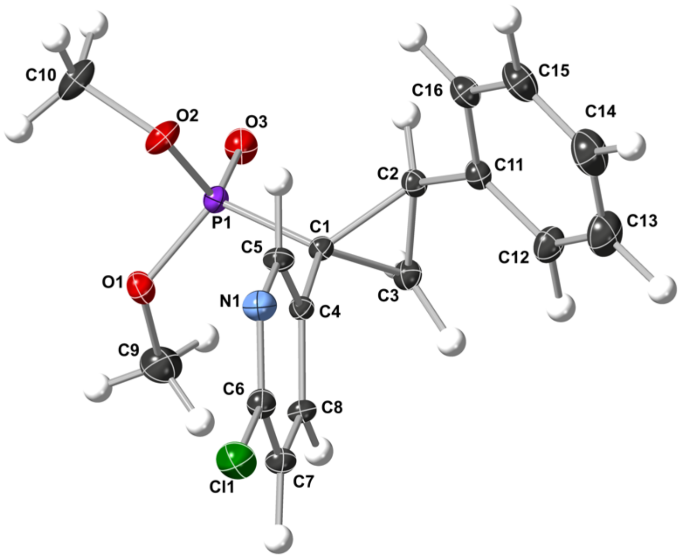

Experimental. Single colorless needle-shaped crystals of **6a** were recrystallised from ethyl acetate by slow evaporation. A suitable crystal with dimensions 0.18 × 0.13 × 0.04 mm³ was selected and mounted on a loop with paratone on a XtaLAB Synergy, Dualflex, HyPix diffractometer. The crystal was kept at a steady T = 100.0(1) K during data collection. The structure was solved with the ShelXT 2018/2 (Sheldrick, 2018) solution program and by using Olex2 1.5-alpha (Dolomanov et al., 2009) as the graphical interface. The model was refined with olex2.refine 1.5-alpha (Bourhis et al., 2015) using full matrix least squares minimisation on F^2.

Crystal Data. $C_{16}H_{17}ClNO_3P$, M_r = 337.745, monoclinic, $P2_1$ (No. 4), a = 8.7380(3) Å, b = 6.4981(2) Å, c = 14.2928(4) Å, β = 98.610(3)°, α = γ = 90°, V = 802.40(4) Å³, T = 100.01(10) K, Z = 2, Z' = 1, μ(Mo K_α) = 0.349, 33455 reflections measured, 8032 unique (R_{int} = 0.0552) which were used in all calculations. The final wR_2 was 0.0496 (all data) and R_1 was 0.0370 (I≥2 σ(I)).

Compound	6a
Formula	$C_{16}H_{17}ClNO_3P$
$D_{calc.}$/ g cm^{-3}	1.398
μ/mm^{-1}	0.349
Formula Weight	337.745
Colour	colourless
Shape	needle-shaped
Size/mm³	0.18×0.13×0.04
T/K	100.01(10)
Crystal System	monoclinic
Flack Parameter	0.01(3)
Hooft Parameter	0.01(3)
Space Group	$P2_1$
a/Å	8.7380(3)
b/Å	6.4981(2)
c/Å	14.2928(4)
α/°	90
β/°	98.610(3)
γ/°	90
V/Å³	802.40(4)
Z	2
Z'	1
Wavelength/Å	0.71073
Radiation type	Mo K_α
Θ_{min}/°	3.44
Θ_{max}/°	37.85
Measured Refl's.	33455
Indep't Refl's	8032
Refl's I≥2 σ(I)	6873
R_{int}	0.0552
Parameters	402
Restraints	367
Largest Peak	0.3701
Deepest Hole	-0.3091
GooF	1.0125
wR_2 (all data)	0.0496
wR_2	0.0473
R_1 (all data)	0.0500
R_1	0.0370

Structure Quality Indicators

Reflections:

d min (MoKα) 2Θ=75.7°	I/σ(I)	Rint m=4.15	Full 50.5° 95% to 75.7°
0.58	18.3	5.52%	98.4

Refinement:

Shift	Max Peak	Min Peak	GooF	Hooft
0.001	0.4	-0.3	1.012	.01(3)

A colourless needle-shaped-shaped crystal with dimensions 0.18 × 0.13 × 0.04 mm^3 was mounted on a loop with paratone. Data were collected using a XtaLAB Synergy, Dualflex, HyPix diffractometer operating at T = 100.01(10) K.

Data were measured using ω scans with Mo K_α radiation. The diffraction pattern was indexed and the total number of runs and images was based on the strategy calculation from the program CrysAlisPro system (CCD 43.128a 64-bit (release 20-06-2024)). The maximum resolution that was achieved was Θ = 37.85° (0.58 Å). The unit cell was refined using CrysAlisPro 1.171.43.121a (Rigaku OD, 2024) on 6897 reflections, 21% of the observed reflections.

Data reduction, scaling and absorption corrections were performed using CrysAlisPro 1.171.43.121a (Rigaku OD, 2024). The final completeness is 99.37 % out to 37.85° in Θ. A numerical absorption correction based on gaussian integration over a multifaceted crystal model was performed using CrysAlisPro 1.171.42.74a (Rigaku Oxford Diffraction, 2022). An empirical absorption correction using spherical harmonics, implemented in SCALE3 ABSPACK scaling algorithm was also applied. The absorption coefficient μ of this material is 0.349 mm^{-1} at this wavelength (λ = 0.71073Å) and the minimum and maximum transmissions are 0.806 and 1.000.

The structure was solved and the space group $P2_1$ (# 4) determined by the ShelXT 2018/2 (Sheldrick, 2018) structure solution program and refined by full matrix least squares minimisation on $\boldsymbol{F}^2$ using version of olex2.refine 1.5-alpha (Bourhis et al., 2015). Hydrogen atom positions were located from the electron densities and freely refined using Hirshfeld scattering factors. Refinement was by using NoSpherA2, an implementation of non-spherical atom-form-factors (F. Kleemiss, H. Puschmann, O. Dolomanov, S.Grabowsky - https://doi.org/10.1039/D0SC05526C – 2020). NoSpherA2 implementation of HAR makes use of tailor-made aspherical atomic form factors calculated from a Hirshfeld-partitioned electron density (ED) not from spherical-atom form factors. The ED was calculated from a Gaussian basis set single determinant SCF wavefunction from DFT using selected functionals for a fragment of this crystal. This fragment was embedded in an electrostatic crystal field by employing cluster charges. The following options were used: SOFTWARE: ORCA 5.0 PARTITIONING: NoSpherA2 INT ACCURACY: Normal METHOD: PBE BASIS SET: def2-TZVP CHARGE: 0 MULTIPLICITY: 1 SOLVATION: Ethanol DATE: 2024-09-23_11-01-10

There is a single formula unit in the asymmetric unit, which is represented by the reported sum formula. In other words: Z is 2 and Z' is 1. The moiety formula is C16 H17 Cl N O3 P.

The Flack parameter was refined to 0.01(3). Determination of absolute structure using Bayesian statistics on Bijvoet differences using the Olex2 results in 0.01(3). The chiral atoms in this structure are: C1(S), C2(R). Note: The Flack parameter is used to determine chirality of the crystal studied, the value should be near 0, a value of 1 means that the stereochemistry is wrong, and the model should be inverted. A value of 0.5 means that the crystal consists of a racemic mixture of the two enantiomers.

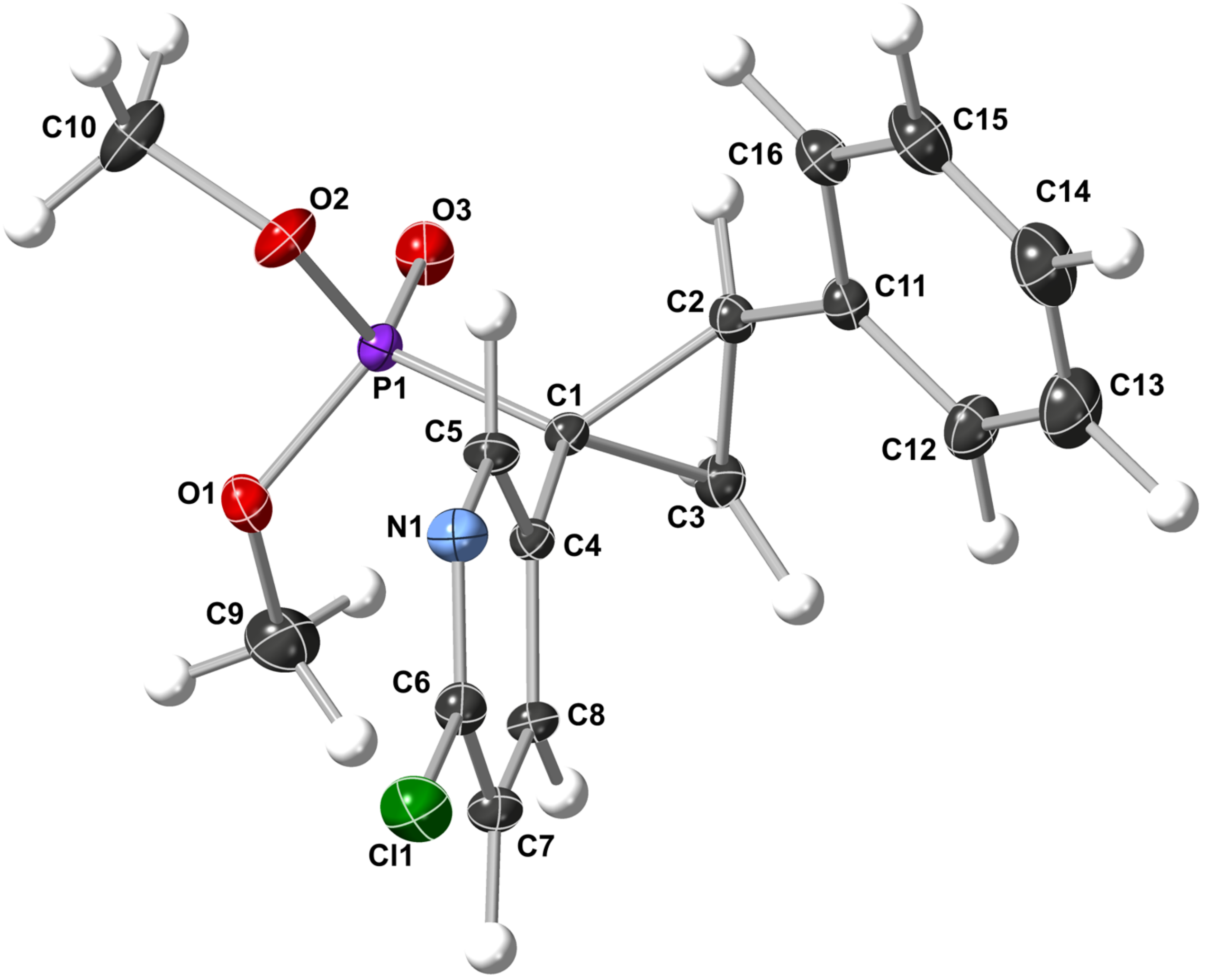

Figure 1 Thermal ellipsoidal representation (50% probability for all atoms, excluding hydrogens) of the molecular structure in the crystal. The chiral atoms in this structure are: C1(S), C2(R).

Data Plots: Diffraction Data

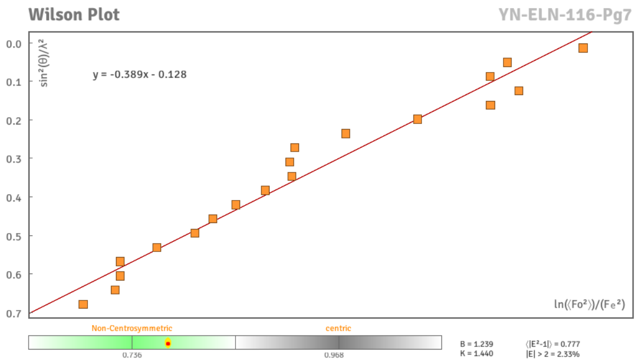

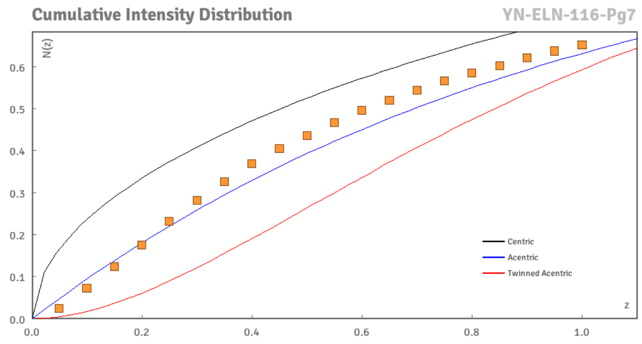

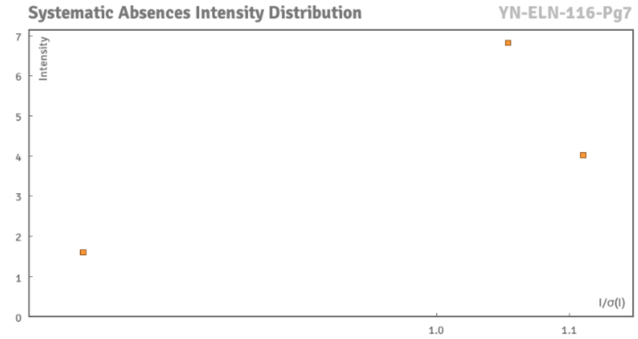

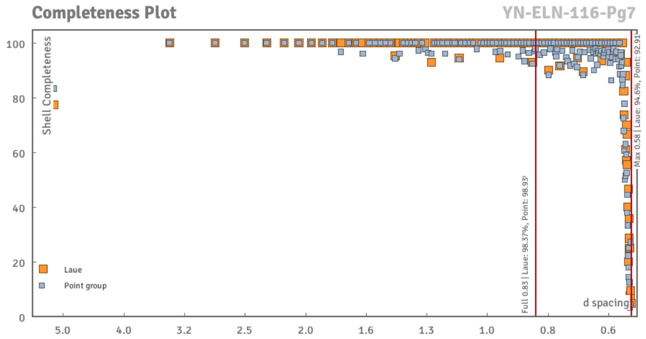

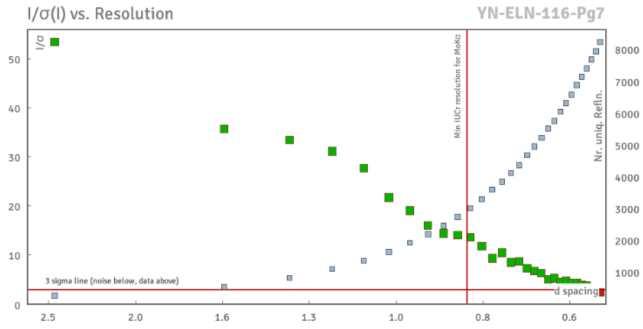

Data Plots: Refinement and Data

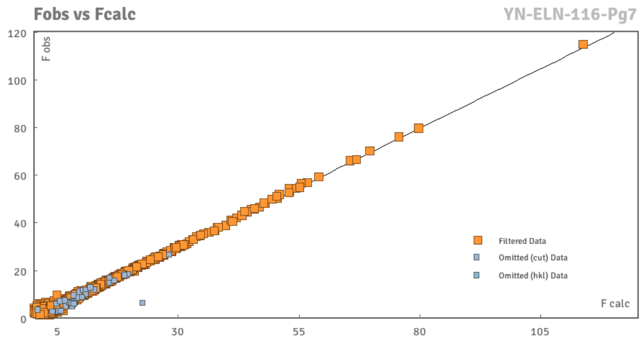

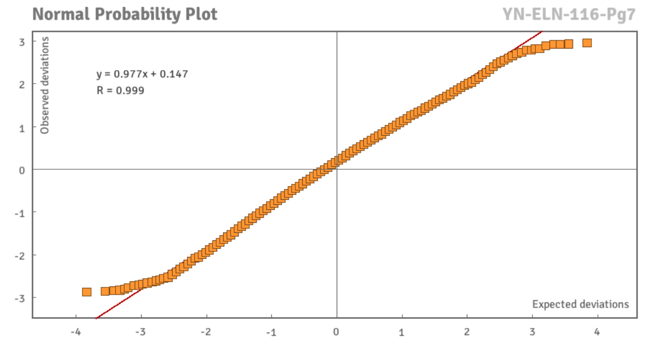

Reflection Statistics

Total reflections (after filtering)	33306	Unique reflections	8032
Completeness	0.929	Mean I/σ	13.77
hkl_{max} collected	(14, 10, 24)	hkl_{min} collected	(-14, -11, -23)
hkl_{max} used	(14, 10, 24)	hkl_{min} used	(-14, -11, 0)
Lim d_{max} collected	100.0	Lim d_{min} collected	0.36
d_{max} used	5.92	d_{min} used	0.58
Friedel pairs	6612	Friedel pairs merged	0
Inconsistent equivalents	0	R_{int}	0.0551
R_{sigma}	0.0546	Intensity transformed	0
Omitted reflections	152	Omitted by user (OMIT hkl)	10
Multiplicity	(4069, 4916, 3473, 1403, 494, 117, 33, 12, 3)	Maximum multiplicity	14
Removed systematic absences	3	Filtered off (Shel/OMIT)	0

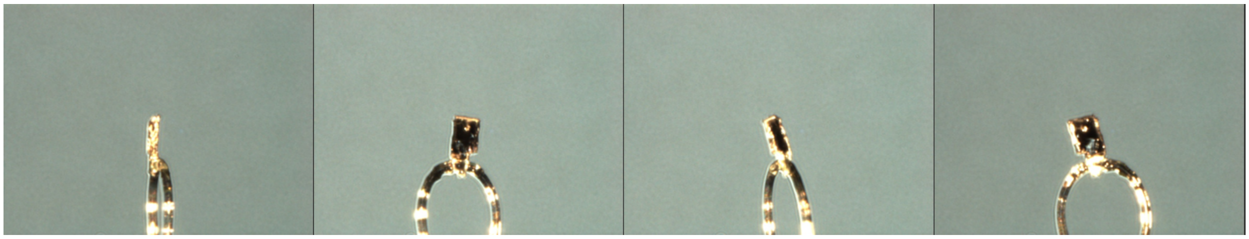

Table 1: Fractional Atomic Coordinates (×10⁴) and Equivalent Isotropic Displacement Parameters (Å²×10³) for **6a**. U_{eq} is defined as 1/3 of the trace of the orthogonalised U_{ij}.

Atom	x	y	z	U_{eq}
C1	2909.6(8)	3958.4(14)	7660.4(5)	11.82(12)
C2	1737.3(9)	4012.6(15)	6738.3(5)	14.34(14)
C3	2596.1(11)	2074.0(13)	7032.0(7)	16.34(17)
C4	4414.5(9)	5074.0(13)	7703.8(6)	11.88(15)
C5	4415.0(10)	7222.8(13)	7671.7(6)	14.86(16)
C6	7031.1(11)	7378.8(14)	7798.4(6)	16.42(17)
C7	7192.6(10)	5246.6(14)	7862.1(7)	17.00(17)
C8	5845.7(9)	4080.1(15)	7804.3(6)	14.32(15)
C9	3902.6(16)	1089.2(18)	9591.1(9)	32.3(3)
C10	1154.4(15)	6789.0(17)	9769.9(9)	28.9(2)
C11	2074.7(10)	5329.8(13)	5940.3(6)	15.60(16)
C12	3041.6(13)	4716.8(17)	5298.9(8)	23.8(2)
C13	3345.0(14)	6038.1(18)	4582.5(8)	32.9(2)
C14	2694.5(15)	7996.9(18)	4503.3(8)	32.8(2)
C15	1711.5(13)	8613.9(15)	5130.7(7)	26.4(2)
C16	1393.1(11)	7282.0(14)	5840.1(7)	19.53(18)
Cl1	8655.8(5)	8925.7(5)	7843.2(3)	25.91(13)
N1	5692.5(9)	8370.3(11)	7710.0(6)	17.37(15)
O1	3375.0(8)	3186.6(9)	9541.1(5)	19.57(14)
O2	1922.0(8)	6243.5(9)	8973.7(5)	20.48(14)
O3	637.4(8)	2691.2(10)	8632.8(5)	20.99(14)
P1	2069.0(5)	3904.5(6)	8722.5(3)	12.67(11)

Table 2: Anisotropic Displacement Parameters (×10⁴) for **6a.** The anisotropic displacement factor exponent takes the form: $-2\pi^2[h^2a^{*2} \times U_{11} + ... + 2hka^* \times b^* \times U_{12}]$

Atom	U_{11}	U_{22}	U_{33}	U_{23}	U_{13}	U_{12}
C1	11.5(3)	11.3(3)	12.9(3)	-0.6(3)	2.9(2)	-0.7(4)
C2	13.3(3)	16.0(3)	13.5(3)	-1.6(3)	1.4(2)	-0.2(3)
H2	13(4)	40(7)	41(7)	-1(2)	7(2)	10(6)
C3	19.0(4)	12.1(4)	17.7(4)	-1.6(3)	2.2(3)	-2.3(3)
H3a	37(7)	24(5)	45(8)	-10(3)	10(4)	4(3)
H3b	24(5)	27(6)	28(7)	-5(3)	9(3)	-8(4)
C4	10.6(3)	12.2(4)	12.9(4)	-0.5(3)	1.9(3)	-0.5(3)
C5	11.7(4)	12.5(4)	20.4(4)	-0.1(3)	2.2(3)	-0.1(3)
H5	16(4)	18(5)	84(11)	1(2)	13(3)	11(5)
C6	13.0(4)	19.8(4)	17.0(4)	-3.8(3)	3.7(3)	-1.0(3)
C7	11.6(4)	20.5(4)	19.3(4)	0.3(3)	3.7(3)	-0.6(3)
H7	14(4)	38(7)	73(11)	8(2)	3(3)	0(5)
C8	12.1(3)	14.2(4)	17.0(3)	2.1(3)	3.4(3)	-0.3(4)
H8	41(8)	13(4)	57(10)	3(2)	-1(6)	0(2)

Atom	U_{11}	U_{22}	U_{33}	U_{23}	U_{13}	U_{12}
C9	37.2(7)	32.4(6)	27.9(6)	12.1(5)	6.8(5)	14.4(5)
H9a	68(7)	79(11)	83(9)	19(5)	43(4)	11(6)
H9b	141(13)	71(10)	33(4)	39(6)	4(3)	22(3)
H9c	59(7)	44(7)	98(11)	4(3)	-11(4)	6(4)
C10	34.4(6)	26.9(6)	29.4(6)	-0.2(4)	18.3(5)	-8.1(4)
H10a	83(10)	33(4)	79(10)	2(2)	38(7)	-12(2)
H10b	73(9)	110(11)	35(5)	35(5)	16(3)	-1(4)
H10c	44(4)	67(8)	66(10)	-12(3)	23(3)	-12(6)
C11	15.4(4)	17.9(4)	13.2(3)	-2.6(3)	1.3(2)	0.4(2)
C12	25.8(5)	28.6(5)	18.5(5)	-0.1(4)	8.8(4)	-0.2(4)
H12	34(8)	32(4)	33(8)	2(3)	8(5)	-2(3)
C13	37.3(6)	43.4(6)	20.5(5)	-3.7(5)	12.7(4)	4.9(5)
H13	64(9)	72(10)	44(8)	4(6)	36(4)	2(5)
C14	37.3(6)	40.2(6)	20.6(5)	-6.7(5)	3.7(4)	10.7(4)
H14	72(10)	64(7)	44(7)	-4(6)	21(5)	28(4)
C15	29.6(5)	26.2(6)	21.0(4)	-3.5(4)	-3.5(3)	8.7(4)
H15	56(9)	32(4)	54(10)	8(3)	15(6)	20(3)
C16	20.3(4)	20.2(4)	17.1(4)	0.1(3)	-0.2(3)	3.0(3)
H16	35(7)	33(7)	32(6)	10(4)	12(3)	6(4)
Cl1	17.1(2)	28.9(3)	32.1(3)	-9.1(3)	4.7(2)	0.8(3)
N1	15.5(3)	13.6(4)	23.2(4)	-3.0(2)	3.4(3)	0.4(3)
O1	20.2(3)	22.9(3)	14.9(3)	-0.7(3)	0.3(2)	1.7(2)
O2	26.2(4)	14.2(3)	24.1(3)	1.4(3)	13.7(3)	-0.7(3)
O3	16.6(3)	26.1(4)	21.2(3)	-8.7(3)	5.7(3)	-0.8(3)
P1	13.4(2)	12.8(2)	12.4(2)	-1.3(2)	3.75(17)	0.7(2)

Table 3: Bond Lengths in Å for **6a**.

Atom	Atom	Length/Å
C1	C2	1.5437(10)
C1	C3	1.5191(12)
C1	C4	1.4947(11)
C1	P1	1.7830(8)
C2	H2	1.065(10)
C2	C3	1.4939(12)
C2	C11	1.4903(12)
C3	H3a	1.056(11)
C3	H3b	1.124(12)
C4	C5	1.3970(11)
C4	C8	1.3957(11)
C5	H5	1.063(11)
C5	N1	1.3367(11)
C6	C7	1.3943(12)
C6	Cl1	1.7328(10)
C6	N1	1.3247(12)
C7	H7	1.059(12)
C7	C8	1.3919(12)
C8	H8	1.056(11)
C9	H9a	1.043(15)
C9	H9b	0.990(14)
C9	H9c	1.020(15)
C9	O1	1.4372(12)
C10	H10a	1.075(13)
C10	H10b	1.050(15)
C10	H10c	1.088(15)
C10	O2	1.4488(12)
C11	C12	1.3946(14)
C11	C16	1.3996(12)
C12	H12	1.076(12)
C12	C13	1.3918(15)
C13	H13	1.112(14)
C13	C14	1.3917(16)
C14	H14	1.099(12)
C14	C15	1.3904(17)
C15	H15	1.078(13)
C15	C16	1.3925(14)
C16	H16	1.064(12)
O1	P1	1.5776(7)
O2	P1	1.5714(8)
O3	P1	1.4679(8)

Table 4: Bond Angles in ° for **6a**.

Atom	Atom	Atom	Angle/°
C3	C1	C2	58.38(6)
C4	C1	C2	119.44(7)
C4	C1	C3	119.68(7)
P1	C1	C2	114.96(5)
P1	C1	C3	115.26(6)
P1	C1	C4	116.50(6)
H2	C2	C1	115.4(6)
C3	C2	C1	59.98(6)
C3	C2	H2	115.7(7)
C11	C2	C1	118.98(7)
C11	C2	H2	113.3(6)
C11	C2	C3	123.42(8)
C2	C3	C1	61.63(5)
H3a	C3	C1	115.9(7)
H3a	C3	C2	115.8(7)
H3b	C3	C1	115.8(6)
H3b	C3	C2	118.8(6)
H3b	C3	H3a	117.1(9)
C5	C4	C1	119.21(8)
C8	C4	C1	123.30(8)
C8	C4	C5	117.46(8)
H5	C5	C4	119.0(6)
N1	C5	C4	124.10(8)
N1	C5	H5	116.9(6)
Cl1	C6	C7	119.98(7)
N1	C6	C7	124.67(8)
N1	C6	Cl1	115.35(7)
H7	C7	C6	119.8(7)
C8	C7	C6	117.44(8)
C8	C7	H7	122.7(7)
C7	C8	C4	119.39(9)
H8	C8	C4	119.8(7)
H8	C8	C7	120.8(7)
H9b	C9	H9a	112.4(13)
H9c	C9	H9a	107.4(13)
H9c	C9	H9b	109.4(14)
O1	C9	H9a	109.6(8)
O1	C9	H9b	108.8(8)
O1	C9	H9c	109.3(8)
H10b	C10	H10a	108.2(13)
H10c	C10	H10a	104.9(12)
H10c	C10	H10b	115.5(12)
O2	C10	H10a	107.0(8)
O2	C10	H10b	108.9(8)
O2	C10	H10c	111.9(7)
C12	C11	C2	123.21(8)
C16	C11	C2	117.97(8)
C16	C11	C12	118.81(9)
H12	C12	C11	119.4(7)
C13	C12	C11	120.55(10)
C13	C12	H12	120.1(7)
H13	C13	C12	119.8(7)
C14	C13	C12	120.24(11)
C14	C13	H13	120.0(7)
H14	C14	C13	120.0(9)
C15	C14	C13	119.70(10)
C15	C14	H14	120.3(9)
H15	C15	C14	120.0(7)
C16	C15	C14	120.03(10)
C16	C15	H15	119.9(8)
C15	C16	C11	120.63(10)
H16	C16	C11	119.7(6)
H16	C16	C15	119.7(6)
C6	N1	C5	116.91(8)
P1	O1	C9	120.55(7)
P1	O2	C10	118.65(6)
O1	P1	C1	107.03(4)
O2	P1	C1	103.52(4)
O2	P1	O1	101.03(4)
O3	P1	C1	112.98(4)
O3	P1	O1	114.73(4)
O3	P1	O2	116.21(5)

Table 5: Torsion Angles in ° for **6a**.

Atom	Atom	Atom	Atom	Angle/˚
C1	C2	C11	C12	82.11(10)
C1	C2	C11	C16	-97.33(8)
C1	C3	C2	C11	106.81(6)
C1	C4	C5	N1	179.67(8)
C1	C4	C8	C7	-178.40(8)
C1	P1	O1	C9	-71.75(7)
C1	P1	O2	C10	175.01(7)
C2	C11	C12	C13	-178.28(9)
C2	C11	C16	C15	177.51(8)
C4	C5	N1	C6	-1.06(11)
C4	C8	C7	C6	-1.13(9)
C5	N1	C6	C7	-0.63(10)
C5	N1	C6	Cl1	179.90(7)
C9	O1	P1	O2	-179.73(8)
C9	O1	P1	O3	54.45(9)
C10	O2	P1	O1	-74.28(8)
C10	O2	P1	O3	50.54(9)
C11	C12	C13	C14	0.51(13)
C11	C16	C15	C14	1.08(11)
C12	C13	C14	C15	-1.41(13)
C13	C14	C15	C16	0.61(13)

Table 6: Hydrogen Fractional Atomic Coordinates (×10[4]) and Equivalent Isotropic Displacement Parameters ($Å^2 \times 10^3$) for **6a.** U_{eq} is defined as 1/3 of the trace of the orthogonalised U_{ij}.

Atom	x	y	z	U_{eq}
H2	550(12)	4034(19)	6831(8)	31(3)
H3a	1943(14)	894(16)	7291(9)	35(3)
H3b	3565(14)	1590(18)	6645(8)	26(3)
H5	3341(14)	8019(16)	7593(10)	39(4)
H7	8311(14)	4579(18)	7955(10)	42(4)
H8	5897(15)	2457(17)	7837(10)	38(4)
H9a	4827(19)	930(20)	9212(12)	73(5)
H9b	4180(20)	700(20)	10265(11)	82(5)
H9c	3034(18)	160(20)	9280(13)	69(5)
H10a	1071(19)	8440(20)	9778(11)	63(4)
H10b	1849(18)	6320(30)	10397(10)	72(5)
H10c	-38(18)	6250(20)	9677(10)	58(4)
H12	3550(15)	3205(18)	5360(9)	33(3)
H13	4103(18)	5520(20)	4069(11)	57(4)
H14	2980(17)	9060(20)	3957(10)	59(4)
H15	1223(16)	10141(19)	5082(10)	47(4)
H16	635(15)	7760(17)	6316(9)	32(3)

Compound 6c

(CCDC 2414497 for 6c)

Submitted by: **Yasir Naeem**

Solved by: **John Bacsa**

R_1=1.58%

Crystal Data and Experimental

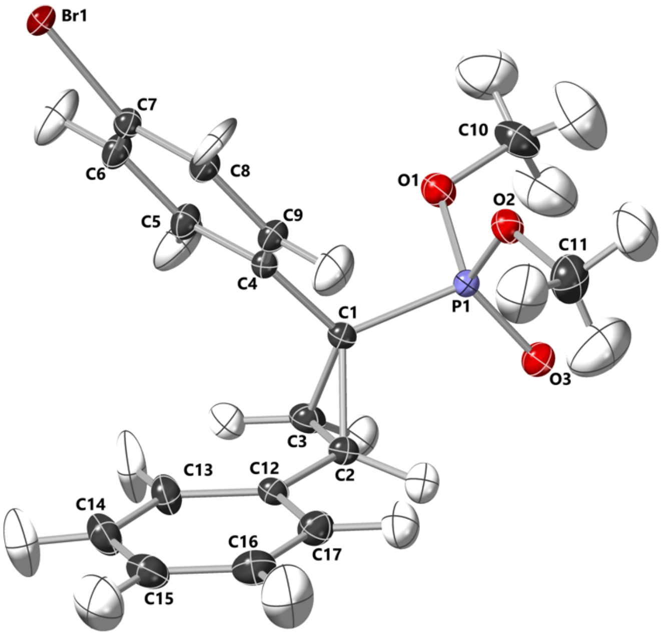

Experimental. A single colorless crystal of **6c** with dimensions 0.25 × 0.18 × 0.05 mm³ was selected and mounted on a loop with paratone on a XtaLAB Synergy, Dualflex, HyPix diffractometer. The crystal was kept at a steady T = 100(1) K during data collection. The structure was solved with the ShelXT (Sheldrick, 2015) solution program using iterative methods and by using Olex2 1.5-alpha (Dolomanov et al., 2009) as the graphical interface. The model was refined with olex2.refine 1.5-alpha (Bourhis et al., 2015) using full matrix least squares minimisation on $\boldsymbol{F}^2$.

Crystal Data. $C_{17}H_{18}BrO_3P$, M_r = 381.208, orthorhombic, $P2_12_12_1$ (No. 19), a = 6.5281(2) Å, b = 9.2356(3) Å, c = 27.8895(7) Å, $\alpha = \beta = \gamma = 90°$, V = 1681.48(9) Å³, T = 158(80) K, Z = 4, Z' = 1, μ(Cu K_α) = 4.313, 29021 reflections measured, 3452 unique (R_{int} = 0.0535) which were used in all calculations. The final wR_2 was 0.0347 (all data) and R_1 was 0.0158 (I≥2 σ(I)).

**Compound**	**6c**
Formula	$C_{17}H_{18}BrO_3P$
$D_{calc.}$/ g cm^{-3}	1.506
μ/mm^{-1}	4.313
Formula Weight	381.208
Color	colorless
Shape	block-shaped
Size/mm³	0.25×0.18×0.05
T/K	158(80)
Crystal System	orthorhombic
Flack Parameter	-0.032(6)
Hooft Parameter	-0.032(6)
Space Group	$P2_12_12_1$
a/Å	6.5281(2)
b/Å	9.2356(3)
c/Å	27.8895(7)
α/°	90
β/°	90
γ/°	90
V/Å³	1681.48(9)
Z	4
Z'	1
Wavelength/Å	1.54184
Radiation type	Cu K_α
Θ_{min}/°	3.17
Θ_{max}/°	78.22
Measured Refl's.	29021
Indep't Refl's	3452
Refl's I≥2 σ(I)	3415
R_{int}	0.0535
Parameters	411
Restraints	309
Largest Peak	0.2098
Deepest Hole	-0.1553
GooF	1.1007
wR_2 (all data)	0.0347
wR_2	0.0345
R_1 (all data)	0.0161
R_1	0.0158

Structure Quality Indicators

**Reflections:**	d min (CuKα) 2Θ=156.4° 0.79	I/σ(I) 48.7	Rint m=8.39 5.35%	Full 135.4° 95% to 156.4° 98.7	
**Refinement:**	Shift -0.001	Max Peak 0.2	Min Peak -0.2	GooF 1.101	Hooft -.032(6)

A colorless block-shaped-shaped crystal with dimensions 0.25 × 0.18 × 0.05 mm^3 was mounted on a loop with paratone. Data were collected using a XtaLAB Synergy, Dualflex, HyPix diffractometer operating at T = 158(80) K.

Data were measured using ω scans with Cu K_α radiation. The diffraction pattern was indexed and the total number of runs and images was based on the strategy calculation from the program CrysAlisPro system (CCD 43.128a 64-bit (release 20-06-2024)). The maximum resolution that was achieved was Θ = 78.22° (0.79 Å).

The unit cell was refined using CrysAlisPro 1.171.43.121a (Rigaku OD, 2024) on 23439 reflections, 81% of the observed reflections.

Data reduction, scaling and absorption corrections were performed using CrysAlisPro 1.171.43.121a (Rigaku OD, 2024). The final completeness is 99.78 % out to 78.22° in Θ. A numerical absorption correction based on gaussian integration over a multifaceted crystal model was performed using CrysAlisPro 1.171.42.74a (Rigaku Oxford Diffraction, 2022). An empirical absorption correction using spherical harmonics, implemented in SCALE3 ABSPACK scaling algorithm was also used. The absorption coefficient μ of this material is 4.313 mm^{-1} at this wavelength (λ = 1.54184Å) and the minimum and maximum transmissions are 0.354 and 1.000.

The structure was solved and the space group $P2_12_12_1$ (# 19) determined by the ShelXT (Sheldrick, 2015) structure solution program using using iterative methods and refined by full matrix least squares minimisation on $\boldsymbol{F}^2$ using version of olex2.refine 1.5-alpha (Bourhis et al., 2015). All atoms were refined anisotropically. Hydrogen atom positions were located from the electron densities and freely refined using Hirshfeld scattering factors. Refinement was by using NoSpherA2, an implementation of non-spherical atom-form-factors (F. Kleemiss, H. Puschmann, O. Dolomanov, S.Grabowsky - https://doi.org/10.1039/D0SC05526C – 2020). NoSpherA2 implementation of HAR makes use of tailor-made aspherical atomic form factors calculated from a Hirshfeld-partitioned electron density (ED) not from spherical-atom form factors. The ED was calculated from a Gaussian basis set single determinant SCF wavefunction from DFT using selected functionals for a fragment of this crystal. This fragment was embedded in an electrostatic crystal field by employing cluster charges. The following options were used: SOFTWARE: ORCA PARTITIONING: NoSpherA2 INT ACCURACY: Normal METHOD: PBE BASIS SET: def2-TZVP CHARGE: 0 MULTIPLICITY: 1 SOLVATION: Ethanol DATE: 2024-09-15_11-56-33

There is a single formula unit in the asymmetric unit, which is represented by the reported sum formula. In other words: Z is 4 and Z' is 1. The moiety formula is C17 H18 Br O3 P.

The Flack parameter was refined to -0.032(6). Determination of absolute structure using Bayesian statistics on Bijvoet differences using the Olex2 results in -0.032(6). The chiral atoms in this structure are: C1(S), C2(R). Note: The Flack parameter is used to determine chirality of the crystal studied, the value should be near 0, a value of 1 means that the stereochemistry is wrong, and the model should be inverted. A value of 0.5 means that the crystal consists of a racemic mixture of the two enantiomers.

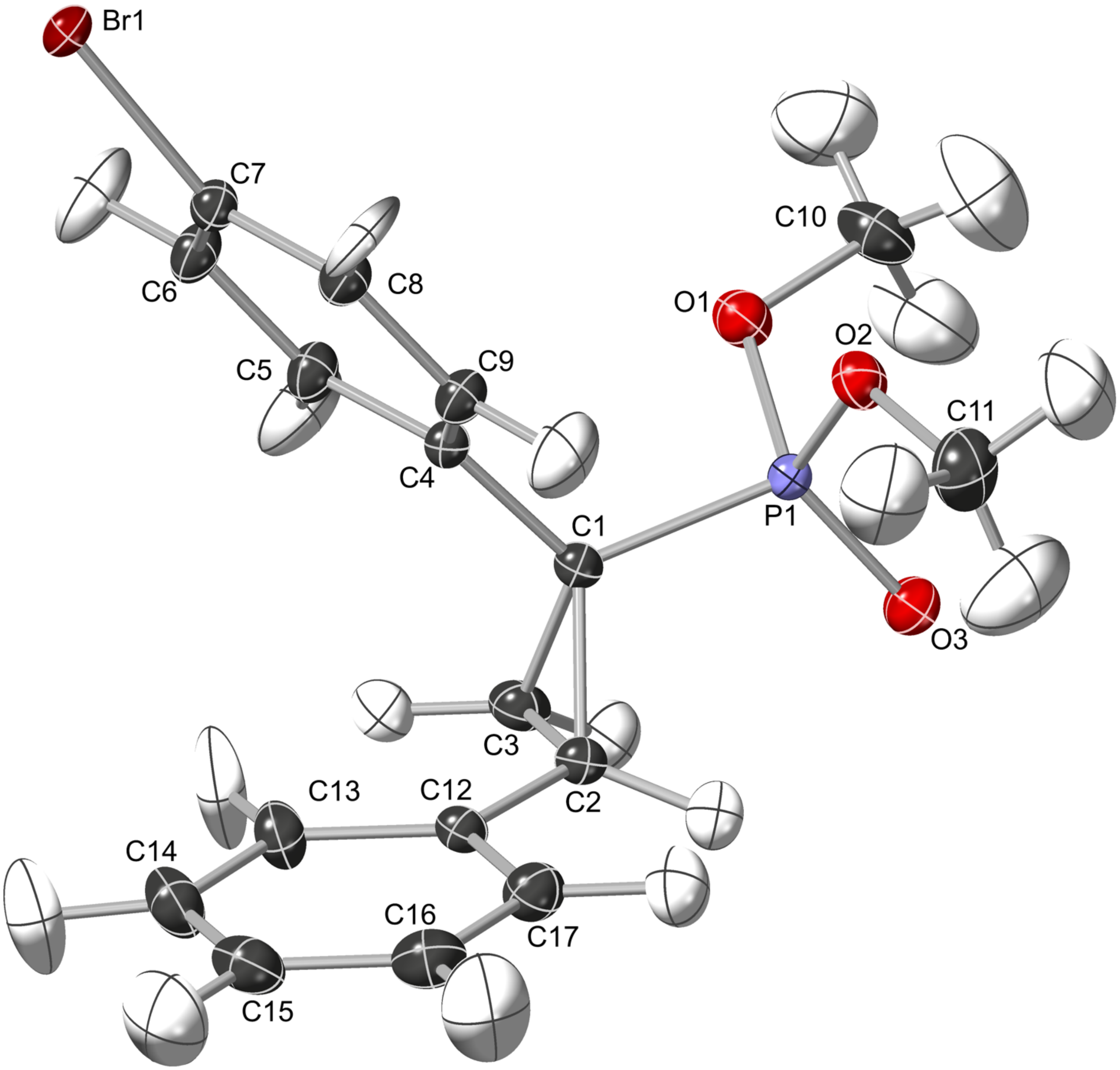

Figure 2 Thermal ellipsoidal representation (50% probability for all atoms, including hydrogens) of the molecular structure in the crystal. The chiral atoms in this structure are: C1(S), C2(R).

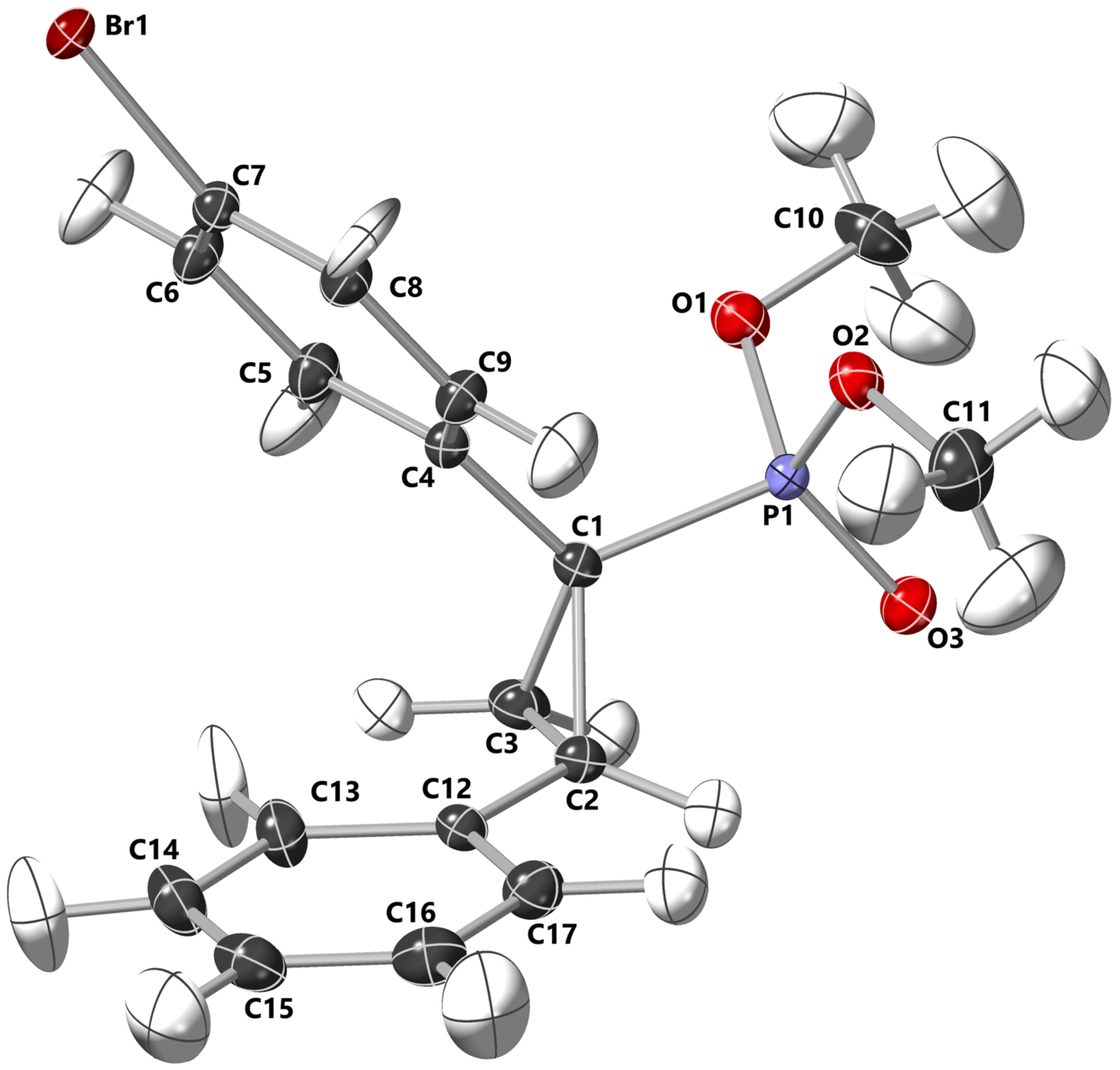

Figure 3 Thermal ellipsoidal representation (50% probability for all atoms, including hydrogens) of the molecular structure in the crystal. The chiral atoms in this structure are: C1(S), C2(R).

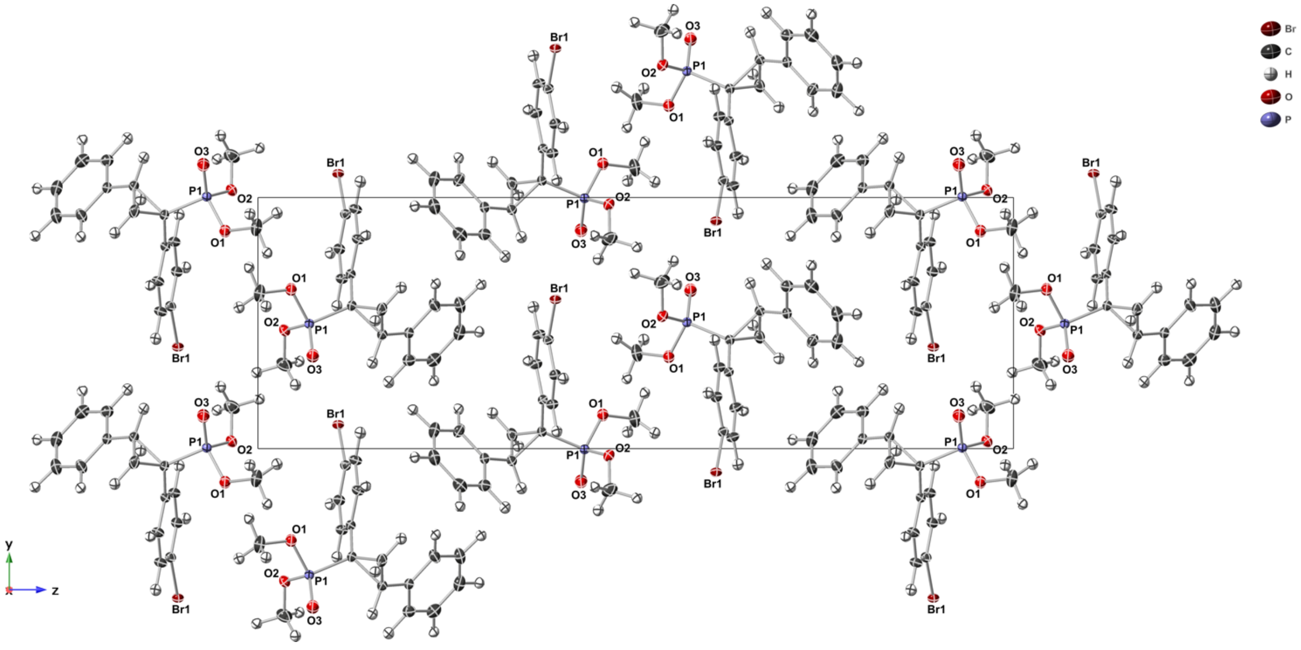

Figure 4 Molecular packing in the crystal structure

Data Plots: Diffraction Data

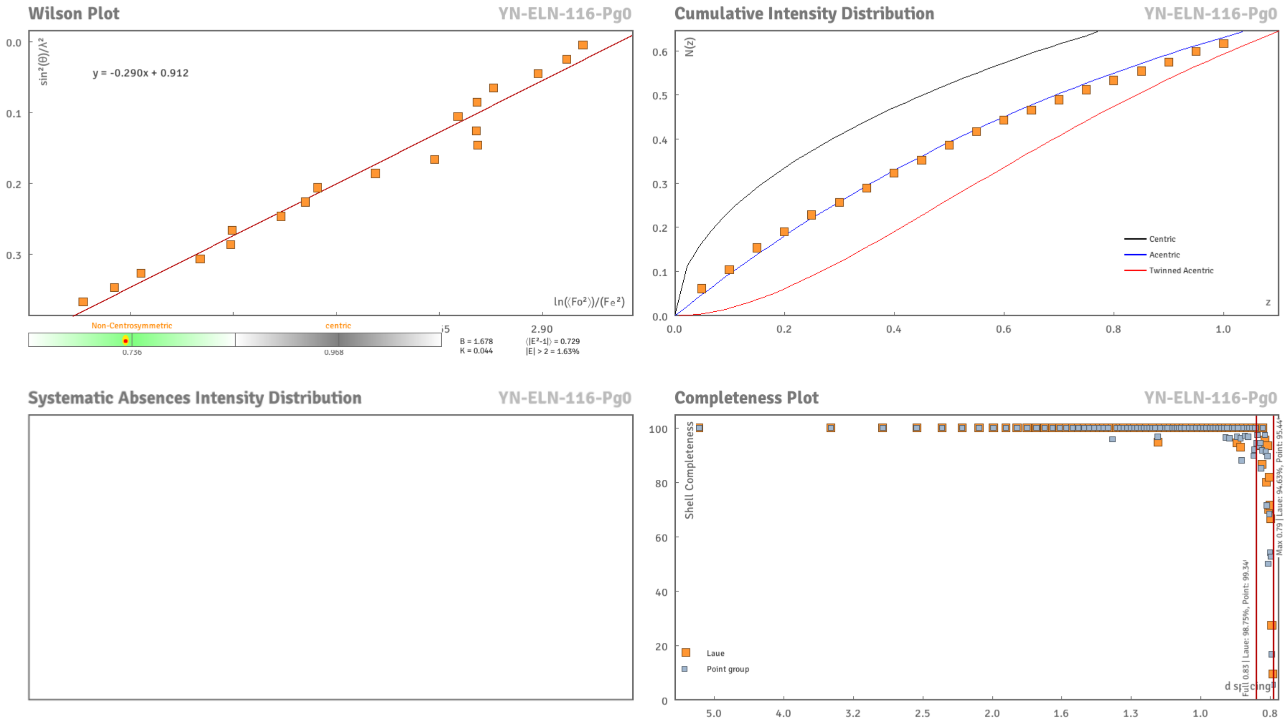

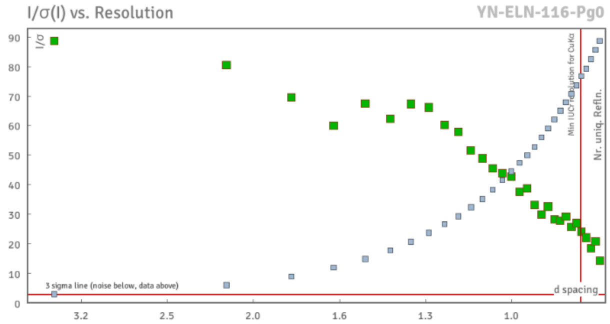

Data Plots: Refinement and Data

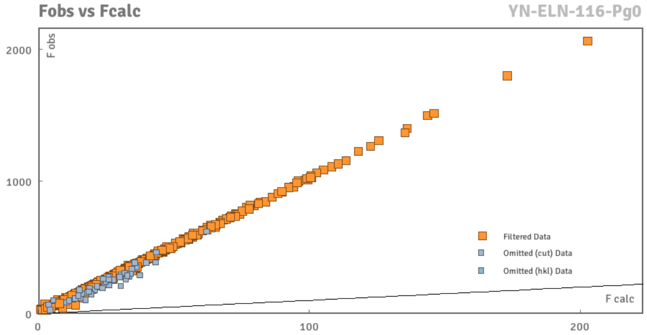

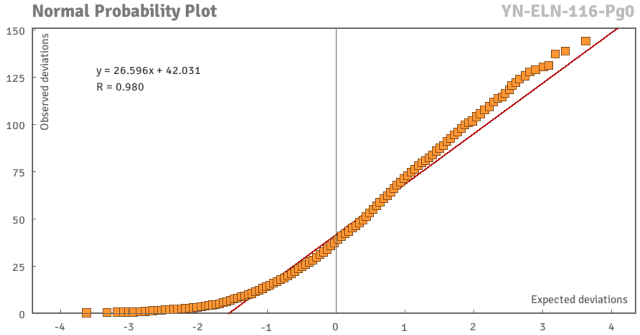

Reflection Statistics

Total reflections (after filtering)	28615	Unique reflections	3447
Completeness	0.953	Mean I/σ	45.24
hkl_{max} collected	(8, 11, 35)	hkl_{min} collected	(-8, -11, -27)
hkl_{max} used	(8, 11, 35)	hkl_{min} used	(-8, 0, 0)
Lim d_{max} collected	100.0	Lim d_{min} collected	0.77
d_{max} used	13.94	d_{min} used	0.79
Friedel pairs	2450	Friedel pairs merged	0
Inconsistent equivalents	19	R_{int}	0.0459
R_{sigma}	0.0181	Intensity transformed	0
Omitted reflections	185	Omitted by user (OMIT hkl)	0
Multiplicity	(2925, 1928, 1216, 983, 689, 426, 321, 254, 131, 82, 48, 54, 33, 21, 13, 2, 2)	Maximum multiplicity	26
Removed systematic absences	0	Filtered off (Shel/OMIT)	0

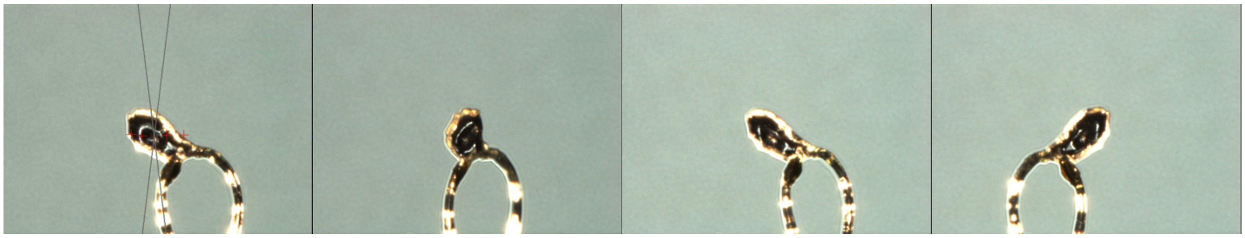

Table 7: Fractional Atomic Coordinates (×10^4) and Equivalent Isotropic Displacement Parameters (Å^2×10^3) for **6c**. U_{eq} is defined as 1/3 of the trace of the orthogonalised U_{ij}.

Atom	x	y	z	U_{eq}
Br1	564.7(5)	-945.6(3)	6062.12(11)	18.07(15)
C1	6362(2)	4317.1(14)	6241.9(4)	16.5(3)
C2	6153(2)	5472.0(15)	6642.4(5)	19.1(3)
C3	7948(2)	4448.7(16)	6638.3(5)	21.7(3)
C4	4933(2)	3046.3(14)	6229.7(4)	15.7(3)
C5	5647(2)	1651.0(15)	6323.3(4)	20.0(3)
C6	4345(2)	457.4(15)	6287.3(5)	20.6(3)
C7	2309(2)	670.4(14)	6158.0(4)	16.2(3)
C8	1558(2)	2053.5(15)	6071.4(5)	18.5(3)
C9	2875(2)	3235.2(14)	6107.4(5)	17.9(3)
C10	9452(3)	3811(2)	5017.6(5)	32.0(4)
C11	4176(3)	6649.6(18)	5347.2(6)	33.9(4)
C12	4468(2)	5391.8(14)	6999.8(4)	18.5(3)
C13	4308(3)	4286.7(16)	7339.2(5)	25.0(3)
C14	2706(3)	4277.0(19)	7669.6(5)	29.2(3)
C15	1235(2)	5363.1(19)	7666.4(5)	28.9(3)
C16	1377(3)	6463.1(18)	7327.9(5)	28.3(3)
C17	2983(2)	6472.5(16)	6999.7(5)	24.3(3)
O1	8175.9(16)	3660.7(11)	5436.5(3)	22.6(2)
O2	5231.7(16)	5283.4(11)	5353.5(3)	21.9(2)
O3	8523.5(17)	6303.7(12)	5723.9(3)	29.2(3)
P1	7182.9(14)	5031.2(10)	5678.0(3)	15.5(2)

Table 8: Anisotropic Displacement Parameters (×10[4]) for **6c**. The anisotropic displacement factor exponent takes the form: $-2\pi^2[h^2a^{*2} \times U_{11} + \ldots + 2hka^* \times b^* \times U_{12}]$

Atom	U_{11}	U_{22}	U_{33}	U_{23}	U_{13}	U_{12}
Br1	20.7(2)	12.8(2)	20.7(2)	-4.36(19)	-0.19(18)	0.38(16)
C1	19.8(6)	16.1(7)	13.7(5)	-1.9(5)	-1.9(5)	-0.1(5)
C2	24.1(8)	18.3(6)	15.0(6)	-3.9(5)	0.2(5)	-1.6(5)
H2	46(11)	24(5)	27(9)	-6(3)	4(6)	3(3)
C3	22.7(7)	25.6(8)	16.7(6)	-3.2(6)	-4.1(6)	-2.5(5)
H3a	25(5)	30(9)	41(9)	-4(3)	0(3)	1(6)
H3b	45(10)	30(6)	22(7)	-6(5)	-6(5)	2(3)
C4	19.6(7)	13.2(6)	14.3(5)	-0.9(5)	-1.5(4)	1.1(4)
C5	19.9(7)	16.4(6)	23.6(6)	-0.1(6)	-3.4(6)	4.1(5)
H5	24(4)	19(9)	76(14)	1(3)	-18(3)	4(7)
C6	21.8(7)	13.9(6)	26.1(6)	-1.3(6)	-4.3(6)	4.0(5)
H6	30(10)	16(5)	77(14)	0(3)	-12(7)	10(3)
C7	16.8(6)	15.0(7)	16.9(5)	-1.8(5)	-0.6(5)	1.5(4)
C8	17.8(6)	16.1(6)	21.5(6)	-0.8(5)	-2.1(6)	1.3(6)
H8	22(4)	8(8)	69(12)	-4(3)	-18(3)	3(6)
C9	17.9(6)	14.2(6)	21.7(6)	-0.2(5)	-1.4(5)	1.1(5)
H9	45(10)	21(5)	53(12)	9(3)	-5(7)	7(3)
C10	32.8(8)	44.6(10)	18.6(6)	0.8(9)	4.1(7)	-5.4(7)
H10a	51(6)	101(14)	46(11)	-25(4)	4(4)	-10(6)
H10b	70(12)	96(13)	46(8)	13(6)	-4(4)	18(5)
H10c	74(14)	58(5)	58(11)	5(3)	18(7)	-16(3)
C11	37.1(10)	27.3(8)	37.4(8)	12.8(8)	4.4(8)	7.7(7)
H11a	50(6)	46(12)	64(11)	11(4)	19(4)	3(6)
H11b	74(14)	48(11)	43(5)	24(7)	1(3)	13(3)
H11c	68(11)	39(8)	95(13)	6(4)	-15(5)	-5(4)
C12	24.3(7)	17.6(6)	13.7(5)	-0.6(6)	-3.1(6)	-0.7(4)
C13	29.2(8)	26.2(8)	19.7(6)	1.6(7)	1.7(6)	6.7(5)

Atom	U_{11}	U_{22}	U_{33}	U_{23}	U_{13}	U_{12}
H13	54(9)	52(9)	48(11)	27(5)	26(7)	35(7)
C14	31.4(8)	37.4(10)	18.8(6)	-3.1(7)	1.3(6)	6.0(6)
H14	41(11)	61(9)	41(8)	12(7)	15(6)	28(5)
C15	29.6(8)	37.0(9)	20.2(6)	-2.8(7)	1.0(6)	-5.3(6)
H15	49(8)	60(13)	44(9)	2(6)	23(4)	2(7)
C16	27.5(8)	27.4(8)	30.2(7)	1.0(7)	0.3(6)	-7.1(6)
H16	51(10)	50(9)	62(13)	25(5)	17(7)	9(7)
C17	27.7(8)	20.4(7)	24.9(6)	1.6(6)	-1.7(6)	-0.8(5)
H17	53(12)	31(7)	33(8)	4(6)	0(6)	8(4)
O1	26.2(5)	22.7(5)	18.8(4)	3.9(4)	0.1(4)	-0.1(4)
O2	26.4(6)	21.0(5)	18.3(4)	3.2(4)	-3.0(4)	2.5(4)
O3	38.4(6)	26.4(6)	22.7(5)	-16.8(5)	1.0(5)	0.3(4)
P1	20.4(5)	13.0(5)	13.0(5)	-3.7(5)	-0.1(4)	0.2(4)

Table 9: Bond Lengths in Å for **6c**.

Atom	Atom	Length/Å
Br1	C7	1.8961(13)
C1	C2	1.5504(18)
C1	C3	1.5195(18)
C1	C4	1.4993(18)
C1	P1	1.7876(15)
C2	H2	1.105(18)
C2	C3	1.505(2)
C2	C12	1.4867(19)
C3	H3a	1.093(18)
C3	H3b	1.071(17)
C4	C5	1.3949(18)
C4	C9	1.3971(19)
C5	H5	1.077(17)
C5	C6	1.395(2)
C6	H6	1.094(16)
C6	C7	1.391(2)
C7	C8	1.3893(18)
C8	H8	1.081(16)
C8	C9	1.3931(19)
C9	H9	1.076(17)
C10	H10a	1.08(2)

Atom	Atom	Length/Å
C10	H10b	1.04(2)
C10	H10c	1.04(2)
C10	O1	1.4415(17)
C11	H11a	1.11(2)
C11	H11b	1.080(19)
C11	H11c	1.04(2)
C11	O2	1.4378(19)
C12	C13	1.3959(18)
C12	C17	1.391(2)
C13	H13	1.093(19)
C13	C14	1.394(2)
C14	H14	1.090(18)
C14	C15	1.389(2)
C15	H15	1.093(18)
C15	C16	1.390(2)
C16	H16	1.07(2)
C16	C17	1.392(2)
C17	H17	1.109(17)
O1	P1	1.5736(13)
O2	P1	1.5798(13)
O3	P1	1.4708(13)

Table 10: Bond Angles in ° for **6c**.

Atom	Atom	Atom	Angle/°
C3	C1	C2	58.71(9)
C4	C1	C2	120.02(11)
C4	C1	C3	120.18(11)
P1	C1	C2	113.97(10)
P1	C1	C3	113.98(10)
P1	C1	C4	117.09(9)
H2	C2	C1	112.7(9)
C3	C2	C1	59.62(9)
C3	C2	H2	115.0(10)
C12	C2	C1	120.92(12)

Atom	Atom	Atom	Angle/°
C12	C2	H2	114.3(10)
C12	C2	C3	123.35(12)
C2	C3	C1	61.67(9)
H3a	C3	C1	118.6(9)
H3a	C3	C2	116.9(9)
H3b	C3	C1	116.0(9)
H3b	C3	C2	118.0(10)
H3b	C3	H3a	115.2(14)
C5	C4	C1	120.76(12)
C9	C4	C1	120.39(12)

Atom	Atom	Atom	Angle/˚
C9	C4	C5	118.82(13)
H5	C5	C4	119.2(10)
C6	C5	C4	120.87(13)
C6	C5	H5	119.9(10)
H6	C6	C5	119.7(9)
C7	C6	C5	119.26(12)
C7	C6	H6	121.0(9)
C6	C7	Br1	119.93(10)
C8	C7	Br1	119.17(10)
C8	C7	C6	120.80(12)
H8	C8	C7	120.7(8)
C9	C8	C7	119.35(12)
C9	C8	H8	119.8(8)
C8	C9	C4	120.89(13)
H9	C9	C4	118.6(10)
H9	C9	C8	120.5(10)
H10b	C10	H10a	110.2(18)
H10c	C10	H10a	107.9(19)
H10c	C10	H10b	107.2(18)
O1	C10	H10a	110.7(11)
O1	C10	H10b	111.1(13)
O1	C10	H10c	109.6(12)
H11b	C11	H11a	108.6(16)
H11c	C11	H11a	111.5(17)
H11c	C11	H11b	106.8(17)
O2	C11	H11a	108.8(11)
O2	C11	H11b	110.5(10)

Atom	Atom	Atom	Angle/˚
O2	C11	H11c	110.7(12)
C13	C12	C2	123.11(14)
C17	C12	C2	118.67(12)
C17	C12	C13	118.21(13)
H13	C13	C12	119.5(9)
C14	C13	C12	120.59(15)
C14	C13	H13	119.9(9)
H14	C14	C13	120.5(10)
C15	C14	C13	120.67(14)
C15	C14	H14	118.7(10)
H15	C15	C14	120.2(11)
C16	C15	C14	119.09(15)
C16	C15	H15	120.7(11)
H16	C16	C15	119.2(11)
C17	C16	C15	120.10(16)
C17	C16	H16	120.7(11)
C16	C17	C12	121.35(14)
H17	C17	C12	119.0(11)
H17	C17	C16	119.6(11)
P1	O1	C10	120.48(10)
P1	O2	C11	121.52(11)
O1	P1	C1	101.73(7)
O2	P1	C1	108.46(7)
O2	P1	O1	101.86(7)
O3	P1	C1	113.37(7)
O3	P1	O1	115.78(8)
O3	P1	O2	114.32(8)

Table 11: Torsion Angles in ˚ for **6c**.

Atom	Atom	Atom	Atom	Angle/˚
Br1	C7	C6	C5	175.48(10)
Br1	C7	C8	C9	-175.33(10)
C1	C2	C12	C13	66.32(15)
C1	C2	C12	C17	-114.15(14)
C1	C3	C2	C12	109.10(10)
C1	C4	C5	C6	-176.56(11)
C1	C4	C9	C8	176.75(12)
C1	P1	O1	C10	-166.00(11)
C1	P1	O2	C11	91.79(11)
C2	C12	C13	C14	179.22(13)
C2	C12	C17	C16	-179.49(13)
C4	C5	C6	C7	-0.26(15)
C4	C9	C8	C7	-0.10(15)
C5	C6	C7	C8	-0.88(15)
C6	C7	C8	C9	1.05(14)
C10	O1	P1	O2	82.04(12)
C10	O1	P1	O3	-42.62(13)
C11	O2	P1	O1	-161.40(11)
C11	O2	P1	O3	-35.77(12)
C12	C13	C14	C15	0.23(17)
C12	C17	C16	C15	0.28(17)
C13	C14	C15	C16	0.11(18)
C14	C15	C16	C17	-0.36(17)

Table 12: Hydrogen Fractional Atomic Coordinates (×10[4]) and Equivalent Isotropic Displacement Parameters (Å[2]×10[3]) for **6c**. U_{eq} is defined as 1/3 of the trace of the orthogonalised U_{ij}.

Atom	x	y	z	U_{eq}
H2	6490(30)	6580(20)	6514(6)	32(4)
H3a	9440(30)	4910(19)	6551(6)	32(4)
H3b	7970(30)	3590(19)	6896(6)	32(4)
H5	7220(30)	1500(19)	6425(7)	40(5)
H6	4940(30)	-630(18)	6354(7)	41(5)
H8	-10(30)	2214(17)	5957(7)	33(5)
H9	2320(30)	4315(18)	6044(7)	40(5)
H10a	10870(30)	4340(30)	5107(7)	66(6)
H10b	8700(40)	4380(30)	4748(8)	70(6)
H10c	9800(40)	2790(20)	4881(7)	63(6)
H11a	2710(30)	6530(20)	5544(8)	53(5)
H11b	3840(40)	6970(20)	4983(7)	55(5)
H11c	5090(40)	7460(20)	5496(8)	67(6)
H13	5470(30)	3430(20)	7348(7)	51(6)
H14	2650(30)	3460(20)	7952(7)	48(6)
H15	-20(30)	5350(20)	7926(7)	51(6)
H16	250(40)	7300(20)	7326(7)	55(6)
H17	3080(30)	7350(20)	6729(6)	39(5)

Compound 6g

(CCDC 2415082)

***R*$_1$=1.08%**

Submitted by: **Yasir Naeem, Davies Group**

Solved by: **John Bacsa**

Crystal Data and Experimental

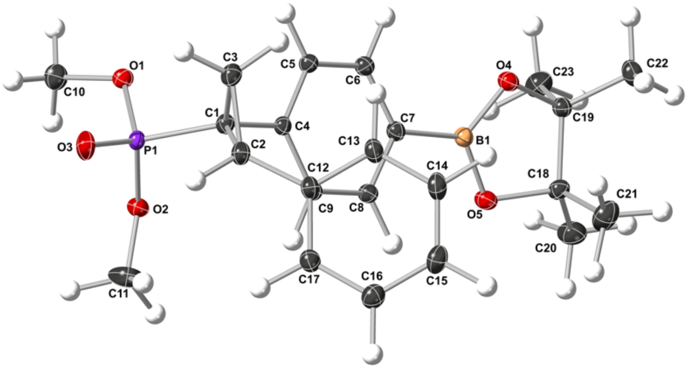

Experimental. Single colorless needle-shaped crystals of **6g** were crystallised from ethyl acetate by slow evaporation. A suitable crystal with dimensions 0.18 × 0.14 × 0.10 mm^3 was selected and mounted on a loop with paratone on a XtaLAB Synergy, Dualflex, HyPix diffractometer. The crystal was kept at a steady T = 100.0(1) K during data collection. The structure was solved with the ShelXT 2018/2 (Sheldrick, 2018) solution program and by using Olex2 1.5-alpha (Dolomanov et al., 2009) as the graphical interface. The model was refined with olex2.refine 1.5-alpha (Bourhis et al., 2015) using full matrix least squares minimisation on $\boldsymbol{F^2}$.

Crystal Data. $C_{23}H_{30}BO_5P$, M_r = 428.290, orthorhombic, $P2_12_12_1$ (No. 19), a = 6.64621(3) Å, b = 13.47483(7) Å, c = 25.47104(11) Å, $\alpha = \beta = \gamma$ = 90°, V = 2281.100(18) Å^3, T = 100.00(10) K, Z = 4, Z' = 1, μ(Cu K$_\alpha$) = 1.320, 68237 reflections measured, 4751 unique (R_{int} = 0.0403) which were used in all calculations. The final wR_2 was 0.0242 (all data) and R_1 was 0.0108 (I≥2 σ(I)).

**Compound**	**6g**
Formula	$C_{23}H_{30}BO_5P$
$D_{calc.}$/ g cm^{-3}	1.247
μ/mm^{-1}	1.320
Formula Weight	428.290
Colour	colourless
Shape	needle-shaped
Size/mm^3	0.18×0.14×0.10
T/K	100.00(10)
Crystal System	orthorhombic
Flack Parameter	0.004(4)
Hooft Parameter	0.004(4)
Space Group	$P2_12_12_1$
a/Å	6.64621(3)
b/Å	13.47483(7)
c/Å	25.47104(11)
α/°	90
β/°	90
γ/°	90
V/Å^3	2281.100(18)
Z	4
Z'	1
Wavelength/Å	1.54184
Radiation type	Cu K$_\alpha$
Θ_{min}/°	3.47
Θ_{max}/°	77.23
Measured Refl's.	68237
Indep't Refl's	4751
Refl's I≥2 σ(I)	4662
R_{int}	0.0403
Parameters	566
Restraints	387
Largest Peak	0.0916
Deepest Hole	-0.0722
GooF	1.1258
wR_2 (all data)	0.0242
wR_2	0.0241
R_1 (all data)	0.0112
R_1	0.0108

Structure Quality Indicators

**Reflections:**	d min (CuKα) 2Θ=154.5° 0.79	I/σ(I) 67.3	Rint m=14.24 4.03%	Full 135.4° 98% to 154.5° 98.6
**Refinement:**	Shift 0.000	Max Peak 0.1	Min Peak -0.1	GooF 1.126	Hooft .004(4)

A colourless needle-shaped-shaped crystal with dimensions 0.18 × 0.14 × 0.10 mm³ was mounted on a loop with paratone. Data were collected using a XtaLAB Synergy, Dualflex, HyPix diffractometer operating at T = 100.00(10) K.

Data were measured using ω scans with Cu K_α radiation. The diffraction pattern was indexed and the total number of runs and images was based on the strategy calculation from the program CrysAlisPro system (CCD 43.128a 64-bit (release 20-06-2024)). The maximum resolution that was achieved was Θ = 77.23° (0.79 Å).

The unit cell was refined using CrysAlisPro 1.171.43.121a (Rigaku OD, 2024) on 52224 reflections, 77% of the observed reflections. Data reduction, scaling and absorption corrections were performed using CrysAlisPro 1.171.43.121a (Rigaku OD, 2024). The final completeness is 99.50 % out to 77.23° in Θ. A numerical absorption correction based on gaussian integration over a multifaceted crystal model was performed using CrysAlisPro 1.171.42.74a (Rigaku Oxford Diffraction, 2022). An empirical absorption correction using spherical harmonics, implemented in SCALE3 ABSPACK scaling algorithm was also applied. The absorption coefficient μ of this material is 1.320 mm^{-1} at this wavelength (λ = 1.54184Å) and the minimum and maximum transmissions are 0.825 and 1.000.

The structure was solved and the space group $P2_12_12_1$ (# 19) determined by the ShelXT 2018/2 (Sheldrick, 2018) structure solution program and refined by full matrix least squares minimisation on $\boldsymbol{F}^2$ using version of olex2.refine 1.5-alpha (Bourhis et al., 2015). Hydrogen atom positions were located from the electron densities and freely refined using Hirshfeld scattering factors. Refinement was by using NoSpherA2, an implementation of non-spherical atom-form-factors (F. Kleemiss, H. Puschmann, O. Dolomanov, S.Grabowsky - https://doi.org/10.1039/D0SC05526C – 2020). NoSpherA2 implementation of HAR makes use of tailor-made aspherical atomic form factors calculated from a Hirshfeld-partitioned electron density (ED) not from spherical-atom form factors. The ED was calculated from a Gaussian basis set single determinant SCF wavefunction from DFT using selected functionals for a fragment of this crystal. This fragment was embedded in an electrostatic crystal field by employing cluster charges. The following options were used: SOFTWARE: ORCA 5.0 PARTITIONING: NoSpherA2 INT ACCURACY: Normal METHOD: PBE0 BASIS SET: def2-TZVP CHARGE: 0 MULTIPLICITY: 1 SOLVATION: Acetone DATE: 2024-09-22_18-05-39

There is a single formula unit in the asymmetric unit, which is represented by the reported sum formula. In other words: Z is 4 and Z' is 1. The moiety formula is C23 H30 B O5 P.

The Flack parameter was refined to 0.004(4). Determination of absolute structure using Bayesian statistics on Bijvoet differences using the Olex2 results in 0.004(4). The chiral atoms in this structure are: C1(S), C2(R). Note: The Flack parameter is used to determine chirality of the crystal studied, the value should be near 0, a value of 1 means that the stereochemistry is wrong, and the model should be inverted. A value of 0.5 means that the crystal consists of a racemic mixture of the two enantiomers.

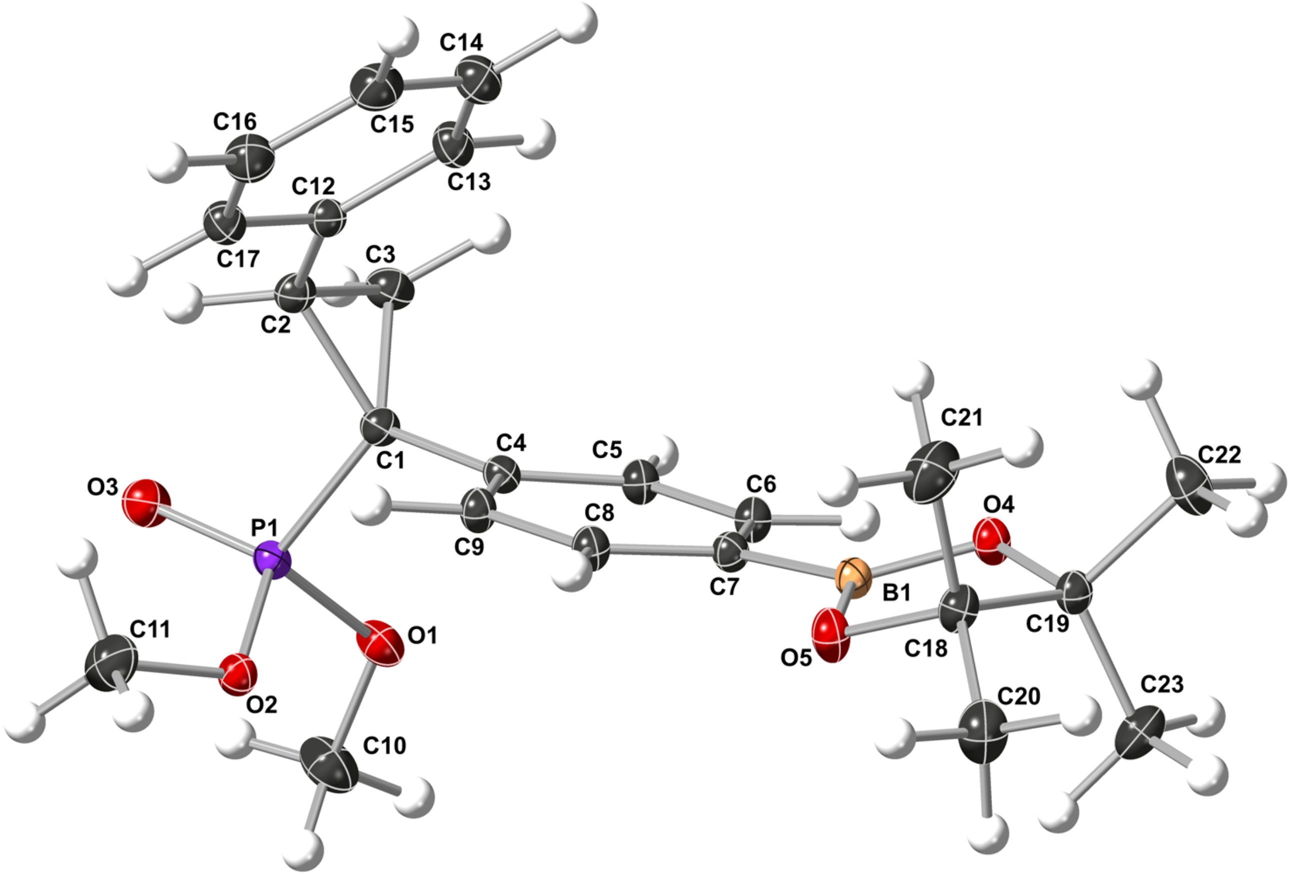

Figure 5 Thermal ellipsoidal representation (50% probability for all atoms, including hydrogens) of the molecular structure in the crystal. The chiral atoms in this structure are: C1(S), C2(R).

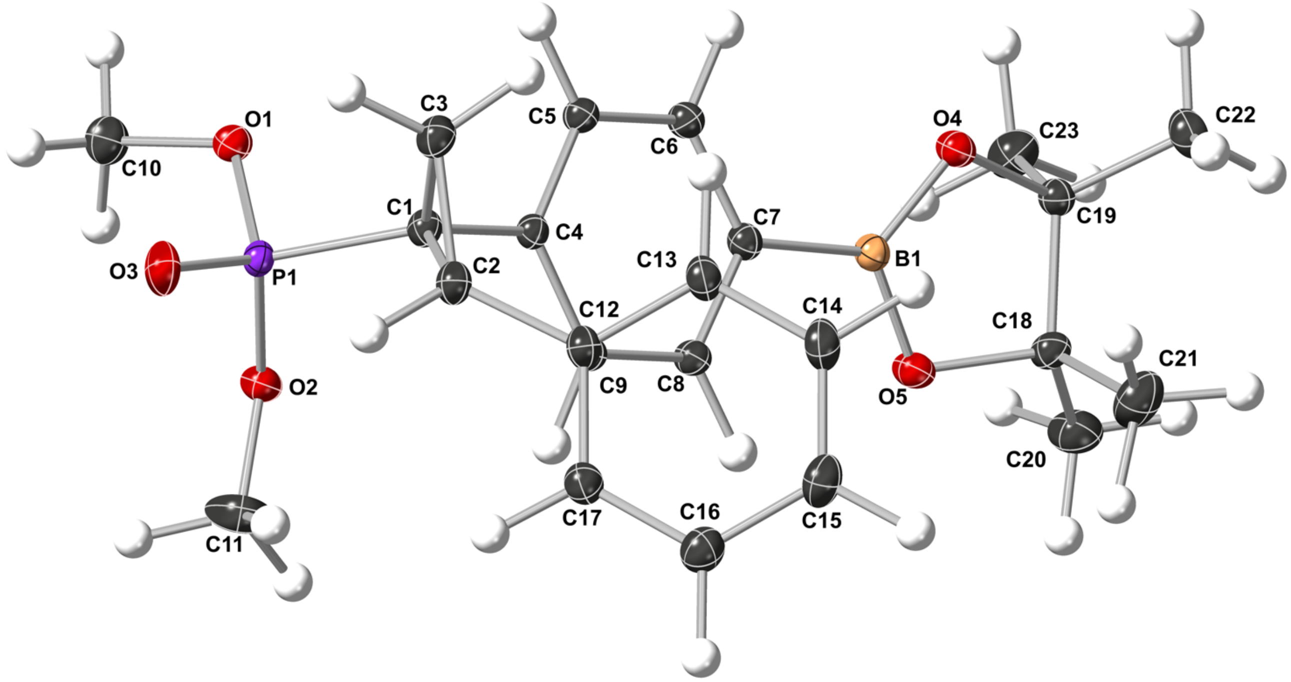

Figure 6 Thermal ellipsoidal representation (50% probability for all atoms, including hydrogens) of the molecular structure in the crystal from a different perspective. The chiral atoms in this structure are: C1(S), C2(R).

Data Plots: Diffraction Data

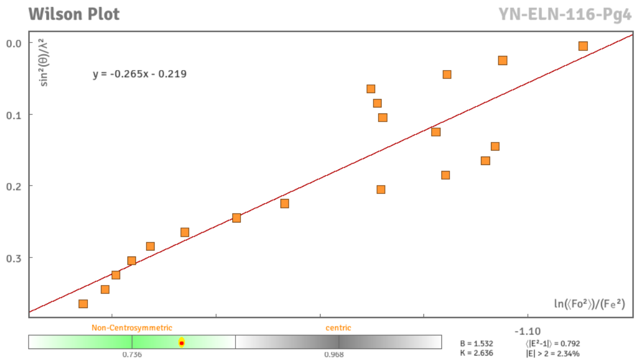

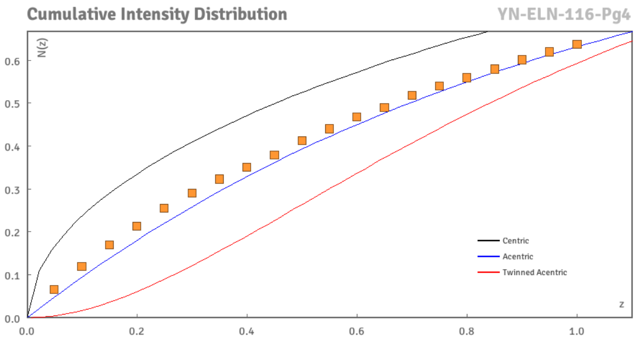

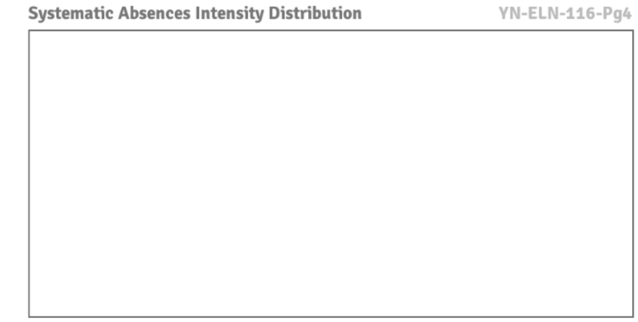

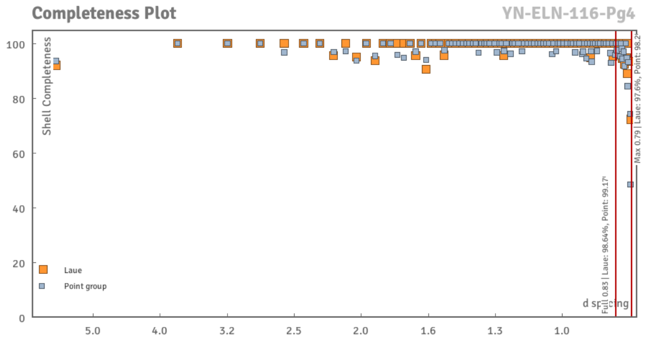

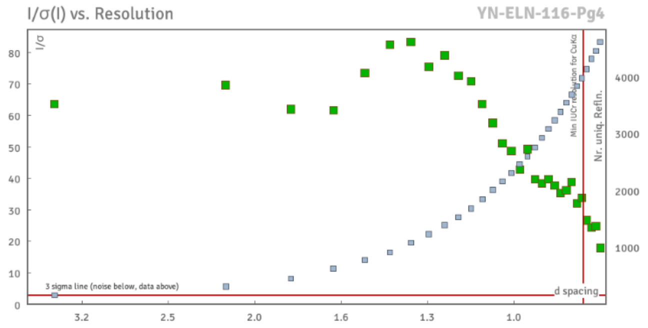

Data Plots: Refinement and Data

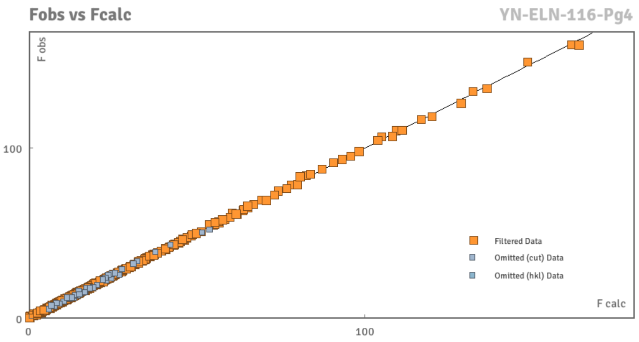

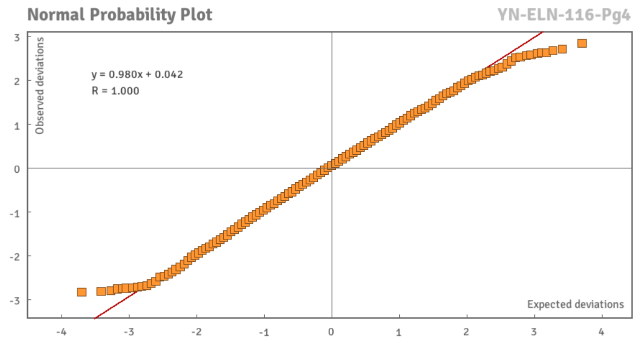

Reflection Statistics

Total reflections (after filtering)	67648	Unique reflections	4751
Completeness	0.982	Mean I/σ	51.11
hkl_{max} collected	(8, 16, 32)	hkl_{min} collected	(-8, -17, -31)
hkl_{max} used	(8, 17, 32)	hkl_{min} used	(-8, 0, 0)
Lim d_{max} collected	100.0	Lim d_{min} collected	0.77
d_{max} used	12.74	d_{min} used	0.79
Friedel pairs	6117	Friedel pairs merged	0
Inconsistent equivalents	0	R_{int}	0.0402
R_{sigma}	0.0149	Intensity transformed	0
Omitted reflections	589	Omitted by user (OMIT hkl)	8
Multiplicity	(3365, 2687, 2025, 1479, 982, 1040, 796, 686, 567, 504, 342, 250, 169, 160, 79, 62, 47, 33, 15, 5)	Maximum multiplicity	37
Removed systematic absences	0	Filtered off (Shel/OMIT)	0

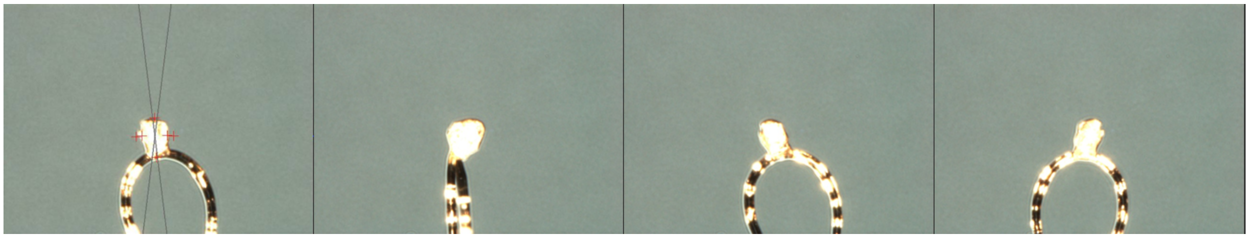

Table 13: Fractional Atomic Coordinates (×10[4]) and Equivalent Isotropic Displacement Parameters (Å[2]×10[3]) for **6g.** U_{eq} is defined as 1/3 of the trace of the orthogonalised U_{ij}.

Atom	x	y	z	U_{eq}
B1	3814.6(8)	2266.0(4)	6881.6(2)	14.78(10)
C1	1281.9(7)	5711.6(4)	5633.10(17)	14.47(9)
C2	1694.1(8)	5632.5(4)	5035.51(18)	16.27(9)
C3	-419.0(8)	5577.6(4)	5239.86(19)	18.49(10)
C4	1897.6(7)	4872.4(3)	5983.53(17)	13.21(9)
C5	489.4(8)	4348.4(4)	6283.25(19)	15.40(9)
C6	1079.1(8)	3537.4(4)	6587.18(19)	15.89(10)
C7	3096.3(7)	3232.1(4)	6602.05(17)	14.61(10)
C8	4504.5(8)	3782.2(4)	6313.57(19)	15.87(10)
C9	3920.4(7)	4588.9(4)	6009.14(19)	15.55(10)
C10	46.1(10)	7833.2(5)	6690.9(3)	30.27(13)
C11	5381.5(9)	7363.4(6)	5842.8(3)	32.22(13)
C12	2806.2(8)	4770.2(4)	4814.65(18)	15.36(10)
C13	2047.1(8)	3805.6(4)	4810.4(2)	19.46(11)
C14	3188.7(9)	3024.8(4)	4609.93(19)	23.22(11)
C15	5107.4(9)	3200.3(4)	4410.3(2)	23.70(11)
C16	5864.3(9)	4163.1(4)	4406.8(2)	22.75(11)
C17	4722.3(8)	4943.1(4)	4605.49(19)	18.69(10)
C18	5947.4(7)	1021.7(4)	7107.00(19)	17.42(10)
C19	3782.2(7)	785.1(4)	7308.77(19)	15.61(10)
C20	7591.4(9)	875.0(5)	7512.1(3)	28.67(13)
C21	6484.4(11)	489.7(5)	6599.5(2)	31.19(13)
C22	3020.4(9)	-245.4(4)	7174.1(3)	27.77(13)

Atom	x	y	z	U_{eq}
C23	3504.5(9)	984.2(4)	7891.0(2)	24.53(11)
O1	134.6(5)	6946.1(3)	6376.96(14)	19.76(8)
O2	3693.5(5)	7025.7(3)	6142.42(12)	16.64(7)
O3	1131.0(6)	7714.5(3)	5486.53(15)	22.65(8)
O4	2558.3(5)	1513.4(3)	7030.05(14)	17.35(8)
O5	5800.1(5)	2072.6(3)	6979.01(14)	20.19(8)
P1	1546.8(5)	6942.0(3)	5879.47(13)	13.25(10)

Table 14: Anisotropic Displacement Parameters (×10[4]) for **6g.** The anisotropic displacement factor exponent takes the form: $-2\pi^2[h^2a^{*2} \times U_{11} + ... + 2hka^* \times b^* \times U_{12}]$

Atom	U_{11}	U_{22}	U_{33}	U_{23}	U_{13}	U_{12}
B1	14.6(3)	13.4(2)	16.3(2)	0.0(2)	-1.86(19)	2.37(19)
C1	14.7(2)	13.1(2)	15.6(2)	1.57(18)	-2.49(18)	0.90(17)
C2	19.7(2)	13.9(2)	15.2(2)	1.95(19)	-2.48(19)	1.11(18)
H2	35(4)	16(3)	29(4)	1(2)	2(3)	3.2(19)
C3	16.6(2)	17.8(3)	21.1(2)	3.1(2)	-5.38(19)	-1.0(2)
H3a	24(4)	24(3)	34(4)	-2.6(18)	-8(3)	1(2)
H3b	30(4)	29(3)	45(4)	13(2)	-7(3)	0(2)
C4	12.9(2)	11.7(2)	15.1(2)	0.74(17)	-1.36(17)	0.29(16)
C5	13.4(2)	14.4(2)	18.4(2)	1.08(18)	0.60(18)	2.09(18)
H5	23(4)	31(4)	46(4)	5(3)	5(3)	5(4)
C6	15.1(2)	14.7(2)	17.8(2)	-0.02(19)	1.44(19)	2.74(18)
H6	31(4)	32(4)	35(4)	1(3)	10(3)	10(3)
C7	14.9(2)	13.1(2)	15.8(2)	0.03(18)	-1.48(18)	2.05(17)
C8	13.0(2)	15.0(2)	19.6(2)	0.64(19)	-1.47(19)	3.97(18)
H8	20(4)	36(4)	46(4)	5(3)	-3(3)	14(4)
C9	13.5(2)	14.7(2)	18.4(2)	0.31(19)	-1.09(19)	4.24(18)
H9	20(4)	28(4)	42(4)	1(3)	3(3)	13(3)
C10	29.3(3)	23.0(3)	38.5(3)	-2.5(2)	9.6(3)	-13.5(3)
H10a	54(4)	52(6)	80(6)	-2(3)	-14(2)	-27(4)
H10b	94(7)	35(4)	74(5)	12(3)	2(4)	-2(2)
H10c	78(5)	56(6)	79(5)	-23(4)	47(3)	-33(4)
C11	20.9(3)	50.6(4)	25.3(3)	-11.5(3)	3.5(2)	4.8(3)
H11a	45(5)	95(7)	31(3)	-5(4)	5(2)	-2(2)
H11b	30(4)	160(9)	66(6)	13(3)	8(3)	39(4)
H11c	111(8)	62(4)	80(6)	-29(2)	34(5)	-3(2)
C12	19.5(2)	12.5(2)	14.1(2)	0.45(18)	-1.58(18)	0.71(17)
C13	23.7(3)	13.3(2)	21.4(2)	-0.9(2)	1.8(2)	-0.52(19)
H13	33(3)	28(4)	49(5)	-3(2)	15(2)	2(3)
C14	31.2(3)	14.0(2)	24.5(2)	0.6(2)	2.0(2)	-2.66(19)
H14	54(5)	18(3)	55(5)	-10(2)	11(4)	-8(2)
C15	30.6(3)	19.3(3)	21.2(2)	5.8(2)	2.1(2)	-3.9(2)
H15	51(5)	27(4)	49(5)	16(2)	6(3)	-9(2)
C16	23.8(3)	22.5(3)	21.9(2)	1.9(2)	3.3(2)	-2.5(2)
H16	32(3)	47(5)	56(5)	2(2)	19(2)	3(4)
C17	20.2(3)	16.6(3)	19.2(2)	-1.0(2)	0.89(19)	-0.78(19)
H17	38(4)	23(3)	44(4)	-10.6(19)	11(3)	-2(2)
C18	15.2(2)	18.3(2)	18.8(2)	2.70(18)	1.52(19)	4.22(19)
C19	15.4(2)	14.2(2)	17.2(2)	0.18(18)	-0.21(18)	2.98(18)
C20	17.7(3)	31.4(3)	37.0(3)	1.3(2)	-5.9(2)	12.5(3)
H20a	40(5)	56(5)	49(4)	5(3)	-6(3)	-6(2)
H20b	49(5)	32(3)	49(5)	4(2)	-13(4)	19(2)
H20c	20(3)	72(6)	71(5)	-2(2)	-1(2)	32(4)

Atom	U_{11}	U_{22}	U_{33}	U_{23}	U_{13}	U_{12}
C21	37.5(3)	30.4(3)	25.7(3)	10.4(3)	12.0(3)	1.8(2)
H21a	66(5)	72(6)	24(4)	20(3)	0(2)	-5(3)
H21b	50(4)	62(6)	56(5)	2(3)	27(3)	9(4)
H21c	78(6)	27(3)	48(5)	17(2)	15(4)	0(2)
C22	26.8(3)	15.8(3)	40.6(4)	-2.6(2)	-2.4(3)	2.9(2)
H22a	62(6)	57(6)	39(3)	-5(4)	-6(2)	-3(2)
H22b	49(5)	22(4)	73(5)	6(2)	-11(3)	9(3)
H22c	39(4)	38(5)	82(6)	-10(2)	14(2)	2(4)
C23	26.2(3)	29.5(3)	17.9(2)	5.3(2)	3.3(2)	5.5(2)
H23a	30(3)	64(6)	44(5)	5(2)	16(2)	5(4)
H23b	59(5)	41(4)	31(4)	-8(2)	6(3)	-5(2)
H23c	44(5)	52(5)	46(4)	14(3)	1(3)	21(3)
O1	15.96(17)	17.95(17)	25.35(18)	-1.37(14)	3.73(14)	-6.14(15)
O2	14.89(16)	18.14(17)	16.90(16)	-2.11(14)	-0.40(13)	1.83(13)
O3	29.8(2)	13.78(17)	24.38(18)	4.24(15)	-7.03(16)	2.28(15)
O4	14.08(16)	16.17(18)	21.81(18)	-1.01(13)	-2.07(13)	4.81(14)
O5	14.33(16)	18.55(18)	27.69(18)	-1.11(14)	-1.17(14)	7.54(14)
P1	13.49(19)	10.9(2)	15.38(18)	1.52(15)	-1.38(16)	-0.00(16)

Table 15: Bond Lengths in Å for **6g**.

Atom	Atom	Length/Å
B1	C7	1.5587(7)
B1	O4	1.3670(6)
B1	O5	1.3678(6)
C1	C2	1.5502(6)
C1	C3	1.5211(7)
C1	C4	1.4976(6)
C1	P1	1.7815(6)
C2	H2	1.082(6)
C2	C3	1.4996(7)
C2	C12	1.4876(7)
C3	H3a	1.084(7)
C3	H3b	1.073(7)
C4	C5	1.3990(7)
C4	C9	1.3991(7)
C5	H5	1.084(7)
C5	C6	1.3954(7)
C6	H6	1.075(7)
C6	C7	1.4029(7)
C7	C8	1.4019(7)
C8	H8	1.080(7)
C8	C9	1.3905(7)
C9	H9	1.092(7)
C10	H10a	1.090(9)
C10	H10b	1.077(9)
C10	H10c	1.066(8)
C10	O1	1.4394(6)
C11	H11a	1.051(8)
C11	H11b	1.066(10)
C11	H11c	1.104(10)
C11	O2	1.4311(6)
C12	C13	1.3942(7)
C12	C17	1.4000(7)
C13	H13	1.087(7)
C13	C14	1.3941(7)
C14	H14	1.084(7)
C14	C15	1.3931(8)
C15	H15	1.084(7)
C15	C16	1.3915(8)
C16	H16	1.109(7)
C16	C17	1.3917(8)
C17	H17	1.072(7)
C18	C19	1.5610(7)
C18	C20	1.5158(7)
C18	C21	1.5207(7)
C18	O5	1.4563(6)
C19	C22	1.5172(7)
C19	C23	1.5184(7)
C19	O4	1.4590(6)
C20	H20a	1.106(9)
C20	H20b	1.102(7)
C20	H20c	1.081(8)
C21	H21a	1.101(8)
C21	H21b	1.097(8)
C21	H21c	1.098(8)
C22	H22a	1.104(8)
C22	H22b	1.082(7)
C22	H22c	1.085(8)
C23	H23a	1.103(7)
C23	H23b	1.094(8)
C23	H23c	1.088(7)
O1	P1	1.5769(5)
O2	P1	1.5801(5)
O3	P1	1.4703(5)

Table 16: Bond Angles in ° for **6g.**

Atom	Atom	Atom	Angle/°
O4	B1	C7	123.98(4)
O5	B1	C7	122.51(4)
O5	B1	O4	113.44(4)
C3	C1	C2	58.44(3)
C4	C1	C2	119.01(4)
C4	C1	C3	120.39(4)
P1	C1	C2	113.10(3)
P1	C1	C3	114.57(4)
P1	C1	C4	117.76(3)
H2	C2	C1	112.3(3)
C3	C2	C1	59.81(3)
C3	C2	H2	115.0(4)
C12	C2	C1	120.85(4)
C12	C2	H2	114.0(3)
C12	C2	C3	123.92(4)
C2	C3	C1	61.75(3)
H3a	C3	C1	114.9(3)
H3a	C3	C2	117.4(4)
H3b	C3	C1	117.3(4)
H3b	C3	C2	117.2(4)
H3b	C3	H3a	116.8(5)
C5	C4	C1	121.57(4)
C9	C4	C1	119.77(4)
C9	C4	C5	118.66(4)
H5	C5	C4	118.3(4)
C6	C5	C4	120.67(5)
C6	C5	H5	121.1(4)
H6	C6	C5	119.1(4)
C7	C6	C5	120.87(5)
C7	C6	H6	120.0(4)
C6	C7	B1	123.36(4)
C8	C7	B1	118.46(4)
C8	C7	C6	117.96(4)
H8	C8	C7	119.2(4)
C9	C8	C7	121.28(5)
C9	C8	H8	119.5(4)
C8	C9	C4	120.51(5)
H9	C9	C4	119.4(3)
H9	C9	C8	120.0(3)
H10b	C10	H10a	108.2(8)
H10c	C10	H10a	109.8(7)
H10c	C10	H10b	111.6(8)
O1	C10	H10a	110.0(5)
O1	C10	H10b	110.8(5)
O1	C10	H10c	106.4(4)
H11b	C11	H11a	110.0(7)
H11c	C11	H11a	107.7(8)
H11c	C11	H11b	108.4(9)
O2	C11	H11a	113.9(5)
O2	C11	H11b	107.2(5)
O2	C11	H11c	109.5(6)
C13	C12	C2	123.46(5)
C17	C12	C2	117.77(4)
C17	C12	C13	118.77(5)
H13	C13	C12	120.6(4)
C14	C13	C12	120.63(5)
C14	C13	H13	118.7(4)
H14	C14	C13	119.3(4)
C15	C14	C13	120.25(5)
C15	C14	H14	120.4(4)
H15	C15	C14	119.3(4)
C16	C15	C14	119.44(5)
C16	C15	H15	121.3(4)
H16	C16	C15	120.0(4)
C17	C16	C15	120.31(5)
C17	C16	H16	119.7(4)
C16	C17	C12	120.58(5)
H17	C17	C12	117.4(4)
H17	C17	C16	122.0(4)
C20	C18	C19	114.44(4)
C21	C18	C19	113.57(5)
C21	C18	C20	110.36(5)
O5	C18	C19	102.15(4)
O5	C18	C20	109.13(4)
O5	C18	C21	106.49(4)
C22	C19	C18	114.85(4)
C23	C19	C18	113.42(4)
C23	C19	C22	110.00(4)
O4	C19	C18	102.49(4)
O4	C19	C22	108.63(4)
O4	C19	C23	106.78(4)
H20a	C20	C18	111.1(4)
H20b	C20	C18	110.4(4)
H20b	C20	H20a	110.4(6)
H20c	C20	C18	109.1(4)
H20c	C20	H20a	107.6(7)
H20c	C20	H20b	108.2(6)
H21a	C21	C18	112.4(4)
H21b	C21	C18	109.4(5)
H21b	C21	H21a	107.8(6)
H21c	C21	C18	111.0(4)
H21c	C21	H21a	107.6(7)
H21c	C21	H21b	108.5(7)
H22a	C22	C19	109.1(5)
H22b	C22	C19	109.9(4)
H22b	C22	H22a	110.8(7)
H22c	C22	C19	109.6(4)
H22c	C22	H22a	110.2(7)
H22c	C22	H22b	107.2(6)
H23a	C23	C19	109.4(4)
H23b	C23	C19	110.9(4)
H23b	C23	H23a	109.8(6)
H23c	C23	C19	110.9(4)
H23c	C23	H23a	109.1(6)
H23c	C23	H23b	106.8(6)
P1	O1	C10	118.27(4)

Atom	Atom	Atom	Angle/°
P1	O2	C11	120.30(4)
C19	O4	B1	107.04(4)
C18	O5	B1	106.90(4)
O1	P1	C1	103.15(3)
O2	P1	C1	107.76(3)

Atom	Atom	Atom	Angle/°
O2	P1	O1	101.34(2)
O3	P1	C1	113.61(3)
O3	P1	O1	115.64(3)
O3	P1	O2	114.06(3)

Table 17: Torsion Angles in ° for **6g**.

Atom	Atom	Atom	Atom	Angle/°
B1	C7	C6	C5	-172.89(5)
B1	C7	C8	C9	172.96(4)
B1	O4	C19	C18	-21.39(4)
B1	O4	C19	C22	-143.33(4)
B1	O4	C19	C23	98.08(4)
B1	O5	C18	C19	-24.39(4)
B1	O5	C18	C20	-145.90(4)
B1	O5	C18	C21	94.98(5)
C1	C2	C12	C13	67.43(5)
C1	C2	C12	C17	-111.96(5)
C1	C3	C2	C12	108.89(3)
C1	C4	C5	C6	176.79(4)
C1	C4	C9	C8	-177.00(4)
C1	P1	O1	C10	177.51(4)
C1	P1	O2	C11	92.21(4)
C2	C12	C13	C14	-178.28(5)
C2	C12	C17	C16	178.12(4)
C4	C5	C6	C7	0.39(5)
C4	C9	C8	C7	0.00(6)
C5	C6	C7	C8	1.64(6)
C6	C7	C8	C9	-1.84(5)
C10	O1	P1	O2	66.02(4)
C10	O1	P1	O3	-57.86(5)
C11	O2	P1	O1	-159.87(5)
C11	O2	P1	O3	-34.92(5)
C12	C13	C14	C15	-0.08(6)
C12	C17	C16	C15	0.45(6)
C13	C14	C15	C16	-0.78(6)
C14	C15	C16	C17	0.59(6)

Table 18: Hydrogen Fractional Atomic Coordinates (×10^4) and Equivalent Isotropic Displacement Parameters (Å^2×10^3) for **6g.** U_{eq} is defined as 1/3 of the trace of the orthogonalised U_{ij}.

Atom	x	y	z	U_{eq}
H2	2110(10)	6338(5)	4865(3)	26.8(16)
H3a	-1133(10)	4854(5)	5247(3)	27.2(16)
H3b	-1377(11)	6205(5)	5168(3)	34.5(17)
H5	-1073(10)	4577(5)	6265(3)	33.2(17)
H6	-45(11)	3129(5)	6801(3)	32.4(16)
H8	6061(10)	3552(5)	6314(3)	34.0(18)
H9	5035(10)	4991(5)	5777(3)	30.1(17)
H10a	1478(14)	7949(7)	6890(4)	62(2)
H10b	-254(17)	8472(7)	6449(4)	68(3)
H10c	-1114(15)	7717(6)	6973(4)	71(3)

Atom	x	y	z	U_{eq}
H11a	5282(13)	7202(7)	5440(3)	57(2)
H11b	6690(13)	7025(10)	6005(4)	85(3)
H11c	5528(19)	8175(8)	5884(4)	84(3)
H13	563(11)	3646(5)	4969(3)	36.6(18)
H14	2562(13)	2282(5)	4608(3)	42(2)
H15	5976(12)	2584(5)	4257(3)	42(2)
H16	7374(11)	4313(6)	4239(3)	45(2)
H17	5248(11)	5695(5)	4598(3)	34.9(17)
H20a	7323(12)	1340(6)	7863(3)	48(2)
H20b	7701(13)	87(6)	7624(3)	43(2)
H20c	9016(11)	1098(7)	7345(3)	55(2)
H21a	5325(14)	584(7)	6295(3)	54(2)
H21b	7901(13)	789(7)	6445(3)	56(2)
H21c	6676(15)	-310(6)	6666(3)	51(2)
H22a	2983(13)	-330(7)	6743(3)	53(2)
H22b	3985(12)	-800(6)	7351(3)	48(2)
H22c	1526(13)	-348(6)	7336(4)	53(2)
H23a	1891(11)	942(6)	7990(3)	46(2)
H23b	4092(12)	1715(6)	7997(3)	44(2)
H23c	4325(11)	444(6)	8126(3)	47(2)

Compound 6j

(CCDC 2414498)

Submitted by: **Yasir Naeem, Davies Group**

Solved by: **John Bacsa**

***R_1*=4.15%**

Crystal Data and Experimental

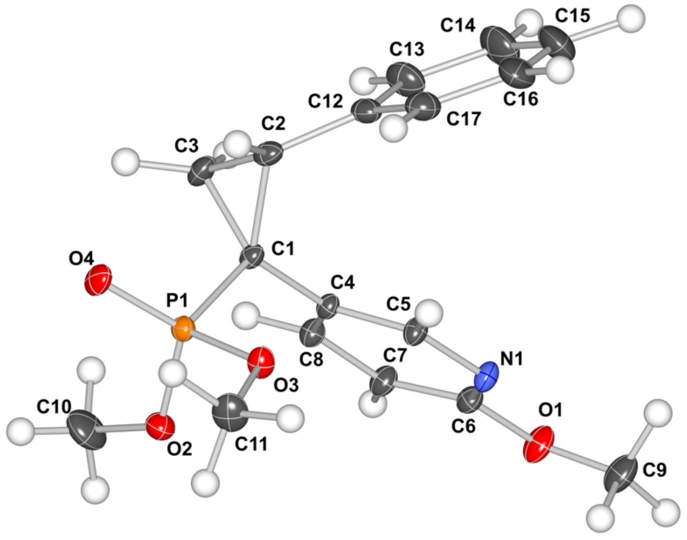

Experimental. Single colourless needle-shaped crystals of **6j** were chosen from the sample as supplied. A suitable crystal with dimensions 0.26 × 0.09 × 0.06 mm³ was selected and mounted on a loop with paratone on a XtaLAB Synergy, Dualflex, HyPix diffractometer. The crystal was kept at a steady T = 100.1(1) K during data collection. The structure was solved with the ShelXT (Sheldrick, 2015) solution program and by using Olex2 1.5-alpha (Dolomanov et al., 2009) as the graphical interface. The model was refined with olex2.refine 1.5-alpha (Bourhis et al., 2015) using full matrix least squares minimisation on F^2. **Crystal Data.** $C_{17}H_{20}NO_4P$, M_r = 333.326, monoclinic, $P2_1$ (No. 4), a = 8.9875(3) Å, b = 6.5712(2) Å, c = 14.3241(4) Å, β = 97.404(3)°, $\alpha = \gamma$ = 90°, V = 838.90(5) Å³, T = 100.1(1) K, Z = 2, Z' = 1, μ(Mo K_α) = 0.183, 22146 reflections measured, 8047 unique (R_{int} = 0.0450) which were used in all calculations. The final wR_2 was 0.0593 (all data) and R_1 was 0.0415 (I≥2 σ(I)).

**Compound**	**6j**
Formula	$C_{17}H_{20}NO_4P$
$D_{calc.}$/ g cm⁻³	1.320
μ/mm⁻¹	0.183
Formula Weight	333.326
Colour	colourless
Shape	needle-shaped
Size/mm³	0.26×0.09×0.06
T/K	100.1(1)
Crystal System	monoclinic
Flack Parameter	0.01(4)
Hooft Parameter	0.01(4)
Space Group	$P2_1$
a/Å	8.9875(3)
b/Å	6.5712(2)
c/Å	14.3241(4)
α/°	90
β/°	97.404(3)
γ/°	90
V/Å³	838.90(5)
Z	2
Z'	1
Wavelength/Å	0.71073
Radiation type	Mo K_α
Θ_{min}/°	2.85
Θ_{max}/°	38.13
Measured Refl's.	22146
Indep't Refl's	8047
Refl's I≥2 σ(I)	6702
R_{int}	0.0450
Parameters	455
Restraints	379
Largest Peak	0.3291
Deepest Hole	-0.2761
GooF	0.9675
wR_2 (all data)	0.0593
wR_2	0.0563
R_1 (all data)	0.0542
R_1	0.0415

Structure Quality Indicators

**Reflections:**	d min (MoKα) 2Θ=76.3° 0.58	I/σ(I) 14.8	Rint m=2.73 4.50%	Full 50.5° 92% to 76.3° 98.6	
**Refinement:**	Shift 0.001	Max Peak 0.3	Min Peak -0.3	GooF 0.968	Hooft .01(4)

A colourless needle-shaped-shaped crystal with dimensions 0.26 × 0.09 × 0.06 mm³ was mounted on a loop with paratone. Data were collected using a XtaLAB Synergy, Dualflex, HyPix diffractometer equipped with an Oxford Cryosystems low-temperature device operating at T = 100.1(1) K.

Data were measured using ω scans with Mo K_α radiation. The diffraction pattern was indexed and the total number of runs and images was based on the strategy calculation from the program CrysAlisPro system (CCD 43.128a 64-bit (release 20-06-2024)). The maximum resolution that was achieved was Θ = 38.13° (0.83 Å). The unit cell was refined using CrysAlisPro 1.171.44.57a (Rigaku OD, 2024) on 10099 reflections, 46% of the observed reflections.

Data reduction, scaling and absorption corrections were performed using CrysAlisPro 1.171.44.57a (Rigaku OD, 2024). The final completeness is 98.61 % out to 38.13° in Θ. A numerical absorption correction based on gaussian integration over a multifaceted crystal model was performed using CrysAlisPro 1.171.42.74a (Rigaku Oxford Diffraction, 2022). An empirical absorption correction using spherical harmonics, implemented in SCALE3 ABSPACK scaling algorithm was also applied. The absorption coefficient μ of this material is 0.183 mm^{-1} at this wavelength (λ = 0.71073Å) and the minimum and maximum transmissions are 0.816 and 1.000.

The structure was solved and the space group $P2_1$ (# 4) determined by the ShelXT (Sheldrick, 2015) structure solution program using using dual methods and refined by full matrix least squares minimisation on $\boldsymbol{F}^2$ using version of olex2.refine 1.5-alpha (Bourhis et al., 2015). Hydrogen atom positions were located from the electron densities and freely refined using Hirshfeld scattering factors. Refinement was by using NoSpherA2, an implementation of non-spherical atom-form-factors (F. Kleemiss, H. Puschmann, O. Dolomanov, S.Grabowsky - https://doi.org/10.1039/D0SC05526C – 2020). NoSpherA2 implementation of HAR makes use of tailor-made aspherical atomic form factors calculated from a Hirshfeld-partitioned electron density (ED) not from spherical-atom form factors. The ED was calculated from a Gaussian basis set single determinant SCF wavefunction from DFT using selected functionals for a fragment of this crystal. This fragment was embedded in an electrostatic crystal field by employing cluster charges. The following options were used: SOFTWARE: ORCA 5.0 PARTITIONING: NoSpherA2 INT ACCURACY: Normal METHOD: PBE BASIS SET: def2-SVP CHARGE: 0 MULTIPLICITY: 1 SOLVATION: Ethanol DATE: 2025-01-04_17-56-54

There is a single formula unit in the asymmetric unit, which is represented by the reported sum formula. In other words: Z is 2 and Z' is 1. The moiety formula is C17 H20 N O4 P.

The Flack parameter was refined to 0.01(4). Determination of absolute structure using Bayesian statistics on Bijvoet differences using the Olex2 results in 0.01(4). The chiral atoms in this structure are: C1(S), C2(R). Note: The Flack parameter is used to determine chirality of the crystal studied, the value should be near 0, a value of 1 means that the stereochemistry is wrong, and the model should be inverted. A value of 0.5 means that the crystal consists of a racemic mixture of the two enantiomers.

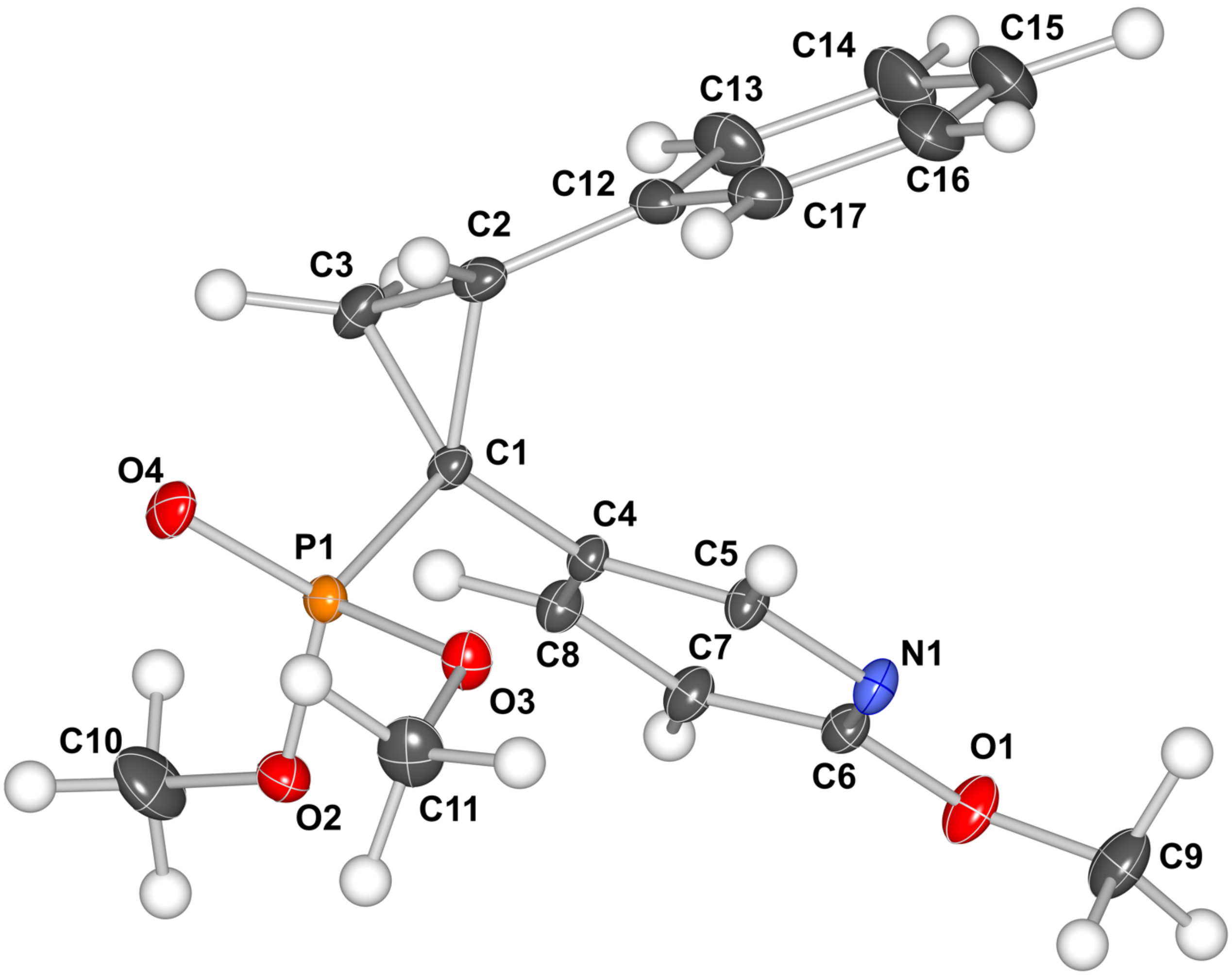

Figure 7 Thermal ellipsoidal representation (50% probability for all atoms, excluding hydrogens) of the molecular structure in the crystal. The chiral atoms in this structure are: C1(S), C2(R).

Data Plots: Diffraction Data

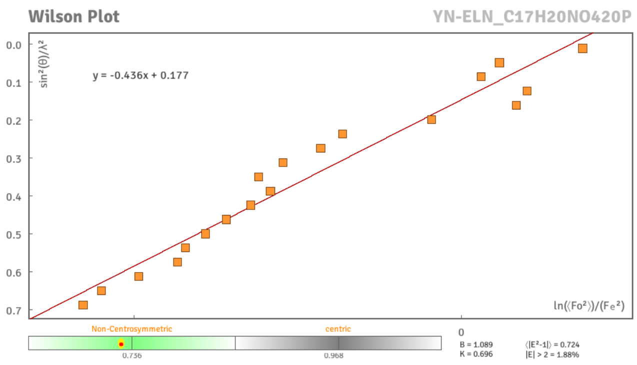

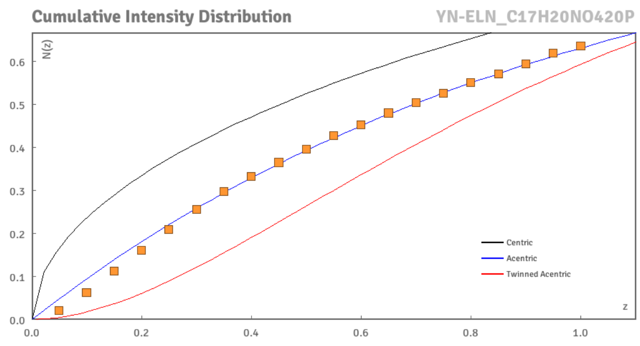

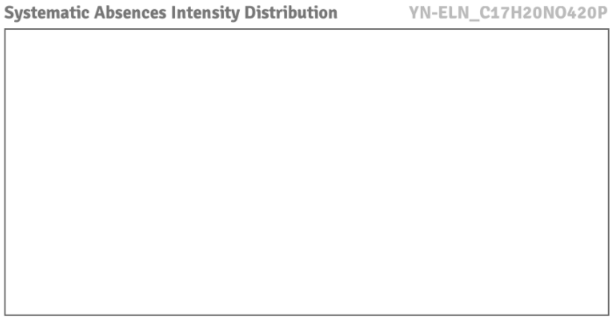

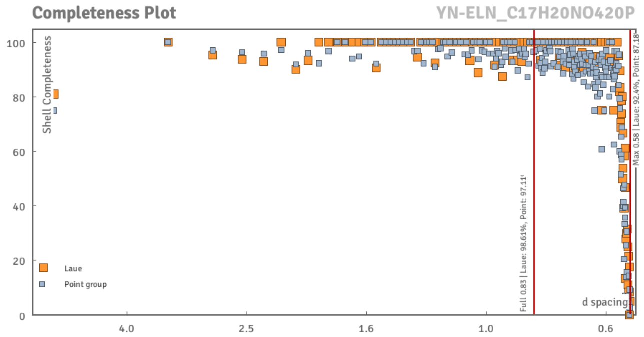

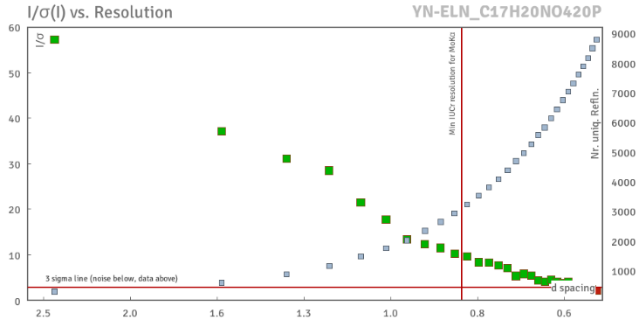

Data Plots: Refinement and Data

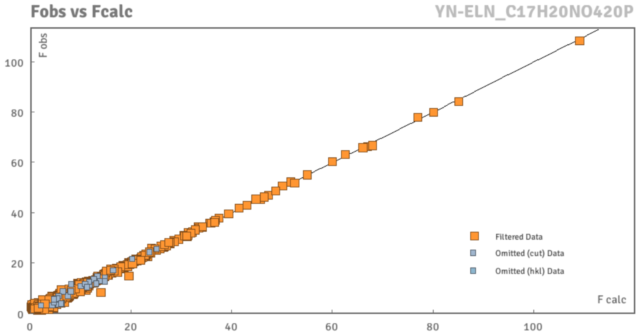

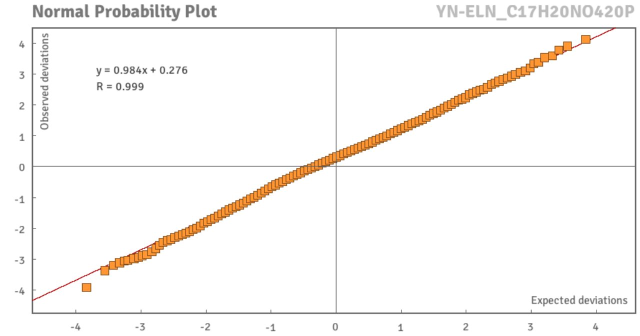

Reflection Statistics

Total reflections (after filtering)	22040	Unique reflections	8061
Completeness	0.874	Mean I/σ	12.29
hkl$_{max}$ collected	(14, 10, 24)	hkl$_{min}$ collected	(-14, -11, -24)
hkl$_{max}$ used	(14, 10, 24)	hkl$_{min}$ used	(-14, -11, 0)
Lim d$_{max}$ collected	100.0	Lim d$_{min}$ collected	0.36
d$_{max}$ used	7.15	d$_{min}$ used	0.58
Friedel pairs	4534	Friedel pairs merged	0
Inconsistent equivalents	2	R$_{int}$	0.0451
R$_{sigma}$	0.0677	Intensity transformed	0
Omitted reflections	0	Omitted by user (OMIT hkl)	106
Multiplicity	(5766, 4138, 1545, 555, 177, 37, 18, 2)	Maximum multiplicity	12
Removed systematic absences	0	Filtered off (Shel/OMIT)	0

There are no images if the crystal on the diffractometer, but the inclusion of these images has been requested from the GUI. Please unitck the relevant box if you don't have these images!

Table 19: Fractional Atomic Coordinates (×10^4) and Equivalent Isotropic Displacement Parameters (Å^2×10^3) for **6j**. U_{eq} is defined as 1/3 of the trace of the orthogonalised U_{ij}.

Atom	x	y	z	U_{eq}
P1	7907.7(6)	3536.2(6)	1242.4(3)	13.53(13)
O4	9260.1(9)	2289.2(11)	1229.0(5)	19.58(16)
O3	8104.3(8)	5899.6(10)	1141.6(4)	17.54(15)
O1	1453.5(14)	6821.9(17)	2293.3(9)	22.55(16)
O2	6624.7(8)	3089.0(10)	402.2(4)	19.60(16)
N1	4022.3(10)	7214.9(12)	2385.3(5)	15.80(16)
C7	2885.6(12)	3900.5(14)	2175.8(6)	16.58(19)
C16	8318(3)	7970(3)	4821.2(13)	32.1(3)
C14	6561(3)	5569(3)	5297.1(14)	37.6(3)
C10	6149.2(16)	1048.6(17)	194.4(8)	33.6(3)
C6	2828.1(11)	6013.1(15)	2288.2(6)	15.05(17)
C12	7931(2)	4755(3)	4003.4(12)	18.77(18)
C9	1346(2)	8933(3)	2291.3(14)	27.0(2)
C1	7095.5(10)	3333.1(15)	2311.2(5)	13.08(16)
C11	8932.1(14)	6664.6(17)	416.5(7)	26.0(2)
C2	8263.6(10)	3453.6(17)	3200.4(5)	16.40(17)
C13	6890(2)	4224(3)	4603.5(12)	28.1(3)
C3	7506.2(12)	1484.9(15)	2924.0(7)	18.46(19)
C15	7260(3)	7455(3)	5406.7(14)	38.5(3)
C4	5568.9(11)	4232.3(13)	2308.7(6)	12.08(16)
C17	8654(2)	6635(3)	4125.8(13)	23.6(2)
C5	5369.4(11)	6318.7(14)	2390.8(6)	14.58(17)
C8	4279.4(11)	3011.3(14)	2196.7(6)	14.44(17)

Table 20: Anisotropic Displacement Parameters (×10^4) for **6j**. The anisotropic displacement factor exponent takes the form: $-2\pi^2[h^2a^{*2} \times U_{11} + ... +2hka^* \times b^* \times U_{12}]$

Atom	U_{11}	U_{22}	U_{33}	U_{23}	U_{13}	U_{12}
P1	12.2(3)	15.4(3)	13.3(2)	2.1(3)	2.93(18)	-0.4(2)
O4	16.0(4)	23.1(4)	20.9(3)	7.9(3)	6.9(3)	1.6(3)
O3	18.9(4)	15.5(3)	19.5(3)	-0.4(3)	7.0(3)	0.6(2)
O1	12.7(4)	21.1(4)	34.5(4)	4.1(3)	5.4(3)	1.6(3)
O2	19.1(4)	22.4(4)	16.6(3)	2.2(3)	-0.3(3)	-2.6(2)
N1	13.6(4)	12.5(4)	21.3(4)	3.2(3)	2.7(3)	0.3(3)
C7	11.5(5)	16.3(5)	22.4(4)	1.5(3)	3.9(3)	1.6(3)
H7	19(3)	30(5)	53(8)	-6.3(17)	11(2)	-12(3)
C16	37.8(7)	33.9(7)	21.9(5)	4.5(5)	-6.5(4)	-6.4(4)
H16	89(10)	49(4)	53(8)	-22(3)	21(5)	-22(3)
C14	40.2(8)	52.9(8)	21.9(5)	4.9(6)	12.5(5)	-6.1(5)
H14	78(8)	86(8)	48(7)	-19(4)	42(4)	-21(4)
C10	40.3(8)	31.6(6)	27.6(6)	-9.6(5)	-0.4(5)	-10.5(4)
H10a	67(7)	50(5)	41(3)	-9(3)	5.4(19)	0.6(17)
H10b	55(3)	51(6)	67(5)	-13.0(17)	-16.5(17)	-7(3)
H10c	60(5)	44(5)	58(5)	-6(2)	15(2)	-16(2)
C6	11.4(4)	16.2(4)	17.7(4)	2.4(3)	2.7(3)	1.3(3)
C12	16.1(4)	25.6(4)	14.2(4)	4.0(3)	0.0(3)	0.9(3)
C9	17.9(6)	25.1(6)	38.5(6)	7.3(4)	5.3(5)	4.0(4)

Atom	U_{11}	U_{22}	U_{33}	U_{23}	U_{13}	U_{12}
H9a	34(6)	38(6)	50(3)	6(2)	12(2)	8.4(19)
H9b	23(2)	36(6)	71(7)	10.9(13)	8.3(13)	6(3)
H9c	29(5)	33(5)	46(3)	8(2)	2.5(17)	-0.4(17)
C1	11.2(4)	13.9(4)	14.6(3)	2.8(3)	3.2(3)	2.2(3)
C11	29.9(7)	25.1(6)	25.4(5)	-3.7(5)	12.4(4)	4.1(4)
H11a	45(5)	47(6)	30(3)	-10(3)	9.7(15)	4.5(16)
H11b	43(6)	28(2)	55(6)	-6.4(13)	18(3)	1.2(13)
H11c	36(3)	37(5)	56(6)	0.9(17)	15.9(17)	3(3)
C2	12.3(4)	21.7(4)	15.3(3)	4.0(4)	2.0(3)	3.5(3)
H2	13.9(17)	34(5)	21(4)	0.1(11)	4.4(10)	-2(2)
C13	27.6(6)	39.2(7)	19.0(5)	-0.7(5)	8.5(4)	0.1(4)
H13	45(8)	45(3)	32(6)	-10(2)	26(4)	-9(2)
C3	17.8(5)	16.2(4)	22.2(4)	5.5(4)	5.6(4)	5.4(3)
H3a	25(5)	21(3)	28(5)	8.9(15)	8(2)	3.8(16)
H3b	22(4)	28(5)	29(5)	4(2)	10(2)	10(2)
C15	47.7(9)	48.4(8)	18.8(5)	8.3(6)	2.2(5)	-10.6(5)
H15	83(10)	61(5)	43(6)	-2(3)	26(4)	-24(3)
C4	9.8(4)	12.3(4)	14.6(4)	1.7(3)	3.4(3)	0.9(3)
C17	22.9(5)	27.3(4)	19.6(4)	0.8(3)	-1.3(4)	0.7(3)
H17	60(7)	41(7)	52(6)	-18(3)	30(3)	-14(3)
C5	11.1(4)	12.5(4)	20.1(4)	0.7(3)	1.9(3)	-0.9(3)
H5	16(3)	20(4)	53(8)	-3.8(16)	3(2)	-1(3)
C8	11.8(4)	13.2(4)	18.7(4)	-0.1(3)	3.3(3)	0.4(3)
H8	14(6)	14(2)	59(8)	-0.4(11)	11(4)	-3.7(12)

Table 21: Bond Lengths in Å for **6j**.

Atom	Atom	Length/Å
P1	O4	1.4681(8)
P1	O3	1.5718(8)
P1	O2	1.5832(8)
P1	C1	1.7839(9)
O3	C11	1.4436(11)
O1	C6	1.3458(15)
O1	C9	1.3909(18)
O2	C10	1.4270(12)
N1	C6	1.3253(13)
N1	C5	1.3454(12)
C7	H7	1.090(13)
C7	C6	1.3993(13)
C7	C8	1.3791(13)
C16	H16	1.081(15)
C16	C15	1.388(3)
C16	C17	1.390(2)
C14	H14	1.063(13)
C14	C13	1.389(3)
C14	C15	1.389(3)
C10	H10a	1.022(7)
C10	H10b	1.022(7)
C10	H10c	1.022(7)
C12	C2	1.4940(19)

Atom	Atom	Length/Å
C12	C13	1.394(2)
C12	C17	1.397(2)
C9	H9a	1.060(7)
C9	H9b	1.060(7)
C9	H9c	1.060(7)
C1	C2	1.5448(12)
C1	C3	1.5160(13)
C1	C4	1.4935(13)
C11	H11a	1.081(7)
C11	H11b	1.081(7)
C11	H11c	1.081(7)
C2	H2	1.043(11)
C2	C3	1.4914(15)
C13	H13	1.060(13)
C3	H3a	1.081(11)
C3	H3b	1.091(11)
C15	H15	1.090(12)
C4	C5	1.3896(12)
C4	C8	1.4017(13)
C17	H17	1.066(12)
C5	H5	1.066(12)
C8	H8	1.069(11)

Table 22: Bond Angles in ° for **6j**.

Atom	Atom	Atom	Angle/°
O3	P1	O4	116.57(5)
O2	P1	O4	114.18(5)
O2	P1	O3	101.13(5)
C1	P1	O4	113.60(5)
C1	P1	O3	102.47(5)
C1	P1	O2	107.46(5)
C11	O3	P1	118.98(6)
C9	O1	C6	117.23(13)
C10	O2	P1	120.14(7)
C5	N1	C6	117.11(8)
C6	C7	H7	121.15(6)
C8	C7	H7	121.15(5)
C8	C7	C6	117.69(9)
C15	C16	H16	119.73(13)
C17	C16	H16	119.73(14)
C17	C16	C15	120.5(2)
C13	C14	H14	119.55(14)
C15	C14	H14	119.55(13)
C15	C14	C13	120.9(2)
H10a	C10	O2	109.5
H10b	C10	O2	109.5
H10b	C10	H10a	109.5
H10c	C10	O2	109.5
H10c	C10	H10a	109.5
H10c	C10	H10b	109.5
N1	C6	O1	119.72(10)
C7	C6	O1	116.10(10)
C7	C6	N1	124.18(9)
C13	C12	C2	123.25(16)
C17	C12	C2	117.83(16)
C17	C12	C13	118.85(17)
H9a	C9	O1	109.5
H9b	C9	O1	109.5
H9b	C9	H9a	109.5
H9c	C9	O1	109.5
H9c	C9	H9a	109.5
H9c	C9	H9b	109.5
C2	C1	P1	113.21(6)
C3	C1	P1	117.44(7)
C3	C1	C2	58.31(6)
C4	C1	P1	116.47(6)
C4	C1	C2	120.54(7)
C4	C1	C3	118.26(7)
H11a	C11	O3	109.5
H11b	C11	O3	109.5
H11b	C11	H11a	109.5
H11c	C11	O3	109.5
H11c	C11	H11a	109.5
H11c	C11	H11b	109.5
C1	C2	C12	118.82(10)
H2	C2	C12	113.3(6)
H2	C2	C1	111.0(6)
C3	C2	C12	124.83(11)
C3	C2	C1	59.88(6)
C3	C2	H2	117.3(7)
C12	C13	C14	120.2(2)
H13	C13	C14	119.89(14)
H13	C13	C12	119.89(11)
C2	C3	C1	61.81(6)
H3a	C3	C1	118.0(6)
H3a	C3	C2	117.2(7)
H3b	C3	C1	113.4(6)
H3b	C3	C2	118.4(6)
H3b	C3	H3a	116.7(9)
C14	C15	C16	119.0(2)
H15	C15	C16	120.52(13)
H15	C15	C14	120.52(13)
C5	C4	C1	121.25(9)
C8	C4	C1	121.30(8)
C8	C4	C5	117.43(9)
C12	C17	C16	120.5(2)
H17	C17	C16	119.74(14)
H17	C17	C12	119.74(11)
C4	C5	N1	123.86(9)
H5	C5	N1	117.0(6)
H5	C5	C4	119.1(6)
C4	C8	C7	119.69(8)
H8	C8	C7	120.15(5)
H8	C8	C4	120.15(5)

Table 23: Torsion Angles in ° for **6j**.

Atom	Atom	Atom	Atom	Angle/°
P1	C1	C2	C12	135.31(10)
P1	C1	C2	C3	-108.94(10)
P1	C1	C3	C2	101.61(10)
P1	C1	C4	C5	-77.87(8)
P1	C1	C4	C8	100.30(7)
O1	C6	N1	C5	178.91(10)
O1	C6	C7	C8	-178.01(9)
N1	C6	C7	C8	2.08(10)
N1	C5	C4	C1	179.45(8)

Atom	Atom	Atom	Atom	Angle/°
N1	C5	C4	C8	1.21(10)
C7	C8	C4	C1	-178.48(7)
C7	C8	C4	C5	-0.24(10)
C16	C15	C14	C13	-1.1(2)
C16	C17	C12	C2	175.76(16)
C16	C17	C12	C13	-1.3(2)
C14	C13	C12	C2	-175.64(17)
C14	C13	C12	C17	1.3(2)
C12	C2	C1	C3	-115.75(13)
C12	C2	C1	C4	-9.38(13)
C12	C2	C3	C1	105.98(14)

Table 24: Hydrogen Fractional Atomic Coordinates (×10^4) and Equivalent Isotropic Displacement Parameters (Å^2×10^3) for **6j**. U_{eq} is defined as 1/3 of the trace of the orthogonalised U_{ij}.

Atom	x	y	z	U_{eq}
H7	1864(12)	2989(11)	2075.2(13)	33(3)
H16	8885(9)	9420(20)	4906.9(18)	63(5)
H14	5765(11)	5148(6)	5750(6)	67(5)
H10a	6065.3(17)	289(6)	808(4)	53(3)
H10b	5128(7)	1062.6(17)	-212(3)	60(3)
H10c	6914(6)	334(6)	-162(3)	53(3)
H9a	1826(4)	9527(5)	1710(4)	40(3)
H9b	202(8)	9365(4)	2236.8(15)	43(3)
H9c	1928(4)	9518(5)	2925(4)	36(3)
H11a	8287(4)	6426(2)	-265(4)	40(3)
H11b	9140(2)	8273(10)	524.3(10)	41(3)
H11c	9989(7)	5867(5)	447.6(7)	42(3)
H2	9341(13)	3668(19)	3021(7)	23(2)
H13	6346(8)	2790(19)	4530.2(16)	39(4)
H3a	8178(14)	312(17)	2654(8)	24(2)
H3b	6603(14)	976(17)	3309(8)	26(3)
H15	6982(4)	8514(13)	5944(6)	61(4)
H17	9471(10)	7050(6)	3682(6)	49(4)
H5	6328(14)	7289(17)	2462(9)	30(3)
H8	4375.9(15)	1399(17)	2127.9(9)	29(3)

10. Citations for X-ray Crystallography

[1] CrysAlisPro (Rigaku, V1.171.42.74a, 2022)

[2] L.J. Bourhis and O.V. Dolomanov and R.J. Gildea and J.A.K. Howard and H. Puschmann, The Anatomy of a Comprehensive Constrained, Restrained, Refinement Program for the Modern Computing Environment - Olex2 Disected, *Acta Cryst. A*, (2015), **A71**, 59-71.

[3] O.V. Dolomanov and L.J. Bourhis and R.J. Gildea and J.A.K. Howard and H. Puschmann, Olex2: A complete structure solution, refinement and analysis program, *J. Appl. Cryst.*, (2009), **42**, 339-341.

[4] Sheldrick, G.M., ShelXT-Integrated space-group and crystal-structure determination, *Acta Cryst.*, (2015), **A71**, 3-8.

[5] CrysAlisPro (Rigaku, V1.171.41.108a, 2021)

[6] O.V. Dolomanov and L.J. Bourhis and R.J. Gildea and J.A.K. Howard and H. Puschmann, Olex2: A complete structure solution, refinement and analysis program, *J. Appl. Cryst.*, (2009), **42**, 339-341.

[7] Sheldrick, G.M., Crystal structure refinement with ShelXL, *Acta Cryst.*, (2015), **C71**, 3-8.

[8] CrysAlisPro (Rigaku, V1.171.41.116a, 2021)

[9] CrysAlisPro (ROD), Rigaku Oxford Diffraction, Poland (?).

[10] CrysAlisPro Software System, Rigaku Oxford Diffraction, (2024).
